# RNA-seq analysis of the key long noncoding RNAs and mRNAs related to cognitive impairment after cardiac arrest and cardiopulmonary resuscitation

**DOI:** 10.18632/aging.103495

**Published:** 2020-07-21

**Authors:** Chan Chen, Changliang Liu, Zhendong Niu, Ming Li, Yuhan Zhang, Rui Gao, Hai Chen, Qiao Wang, Shu Zhang, Ronghua Zhou, Lu Gan, Zheng Zhang, Tao Zhu, Hai Yu, Jin Liu

**Affiliations:** 1 Department of Anesthesiology, Laboratory of Anesthesia and Critical Care Medicine, Translational Neuroscience Center, West China Hospital, Sichuan University and The Research Units of West China, Chinese Academy of Medical Sciences, Chengdu, Sichuan, China; 2 Department of Emergency Medicine, West China Hospital, Sichuan University, Chengdu, Sichuan, China

**Keywords:** long noncoding RNA, cardiac arrest, cardiopulmonary resuscitation, RNA sequencing, signal pathway

## Abstract

Cardiac arrest (CA) is the leading cause of death around the world. Survivors after CA and cardiopulmonary resuscitation (CPR) develop moderate to severe cognitive impairment up to 60% at 3 months. Accumulating evidence demonstrated that long non-coding RNAs (lncRNAs) played a pivotal role in ischemic brain injury. This study aimed to identify potential key lncRNAs associated with early cognitive deficits after CA/CPR. LncRNA and mRNA expression profiles of the hippocampus in CA/CPR or sham group were analyzed via high-throughput RNA sequencing, which exhibited 1920 lncRNAs and 1162 mRNAs were differentially expressed. These differentially expressed genes were confirmed to be primarily associated with inflammatory or apoptotic signaling pathways through GO and KEGG pathway enrichment analysis and coding-noncoding co-expression network analysis. Among which, five key pairs of lncRNA-mRNA were further analyzed by qRT-PCR and western blot. We found that the lncRNANONMMUT113601.1 and mRNA *Shc1*, an inflammation and apoptosis-associated gene, exhibited the most significant changes in hippocampus of CA/CPR mice. Furthermore, we found that the correlations between this lncRNA and mRNA mainly happened in neurons of hippocampus by *in situ* hybridization. These results suggested that the critical pairs of lncRNA-mRNA may act as essential regulators in early cognitive deficits after resuscitation.

## INTRODUCTION

Cardiac arrest (CA) is a common clinical emergency and a leading cause of death [1]. Although the continuous improvement of cardiopulmonary resuscitation (CPR) strategy contributes to enhancing the rate of patients' restoration of spontaneous circulation (ROSC) up to 50%, the survival rate of CA patients is still not more than 20% [2]. Notably, up to 60% of the survivors undergoing CA/CPR developed moderate to severe cognitive impairment at 3 months, and 40%-50% had permanent cognitive impairment, which severely decreased the quality of life in patients and increased the burden on society [3, 4]. Previously, numerous attempts were made to improve neurologic outcomes of patients after successful resuscitation [5]. For example, organ-specific support and targeted hypothermia therapy are recognized as efficient approaches to improve the survival and neurologic outcomes of CA/CPR patients [6, 7]. However, the exact molecular mechanisms of CA/CPR induced cerebral injury, especially cognitive impairment, are still lacking.

Long noncoding RNA (lncRNA), a novel RNA molecule, which is defined as a length greater than 200 nucleotides, has been shown to regulate gene expression potently at the levels of transcription and translation [8]. By linking to microRNA or serving as a scaffold, decoy, tether, or guide for interacting with proteins, lncRNA can enhance or inhibit the transcription of the neighboring or distant target gene [9]. LncRNA-related deregulation may play a substantial role in various diseases, such as cardiovascular diseases [10], cancers [11], as well as neurodegeneration diseases [12]. Besides, recent studies suggested that some lncRNAs could regulate neuronal apoptosis and function as anti-inflammatory factors [13, 14]. Of note, a growing number of studies pointed out that lncRNAs could participate in the pathogenesis of cerebral ischemia/reperfusion(I/R) injury [15, 16].

Nevertheless, it remains unknown whether lncRNAs engage in cognitive deficits of the survivors undergoing CA/CPR. This study aimed to identify potential vital lncRNAs and target mRNAs as new therapeutic targets of CA/CPR induced cognitive impairment. Our findings will lay a foundation for further understanding of the molecular mechanisms of lncRNAs in CA/CPR induced cognitive impairment.

## RESULTS

### Mice following CA/CPR exhibited severely neurological and cognitive dysfunction

**Figure 1:**
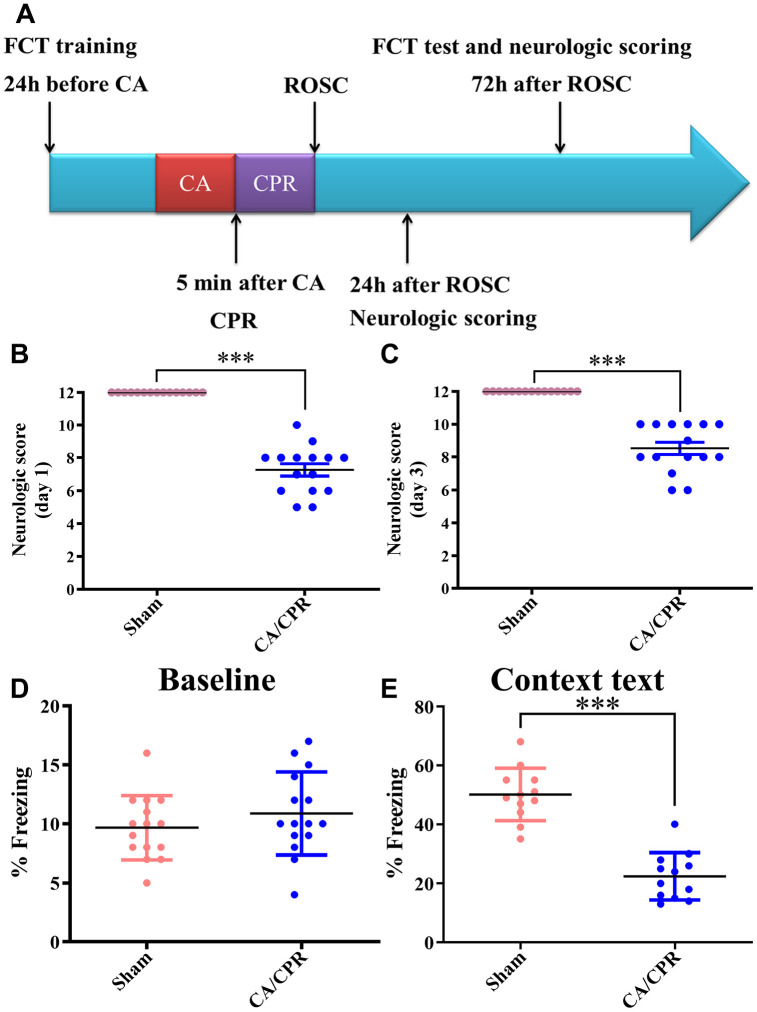
**Neurological function was severely impaired in mice after CA/CPR.** (**A**). Scheme of the experimental design. (**B, C**). Day 1 (B) and day 3 (**C**) average neurologic scores after ROSC. (**D**). Baseline freezing levels in the two groups. (**E**). Freezing levels of context text on day 3 after resuscitation. n=15 per group. ^***^P < 0.001. FCT, fear-conditioning test; CA/CPR, cardiac arrest/cardiopulmonary resuscitation; ROSC, restoration of spontaneous circulation.

As shown in Figure 1A, a scheme of the experimental design is described. Neurological function was assessed in mice from six aspects: movement/activity, respiratory model, consciousness, coordination, righting reflex, and corneal reflex. On day 1 and day 3 after CA/CPR, the neurologic score was remarkably decreased in comparison with the sham procedure (*P* < 0.001, Figure 1B, 1C), indicating severe neurological impairment. In the fear conditioning test, the baseline freezing level between the two groups was little difference (*P* > 0.05, Figure 1D). However, compared with the sham group, contextual memory was significantly decreased in the CA/CPR group, as demonstrated by the shorter average freezing time (*P* < 0.001, Figure 1E). These data confirmed that CA/CPR-induced systemic I/R caused severe brain damage and cognitive deficits in mice.

### Survival rates in sham and CA/CPR groups

**Figure 2:**
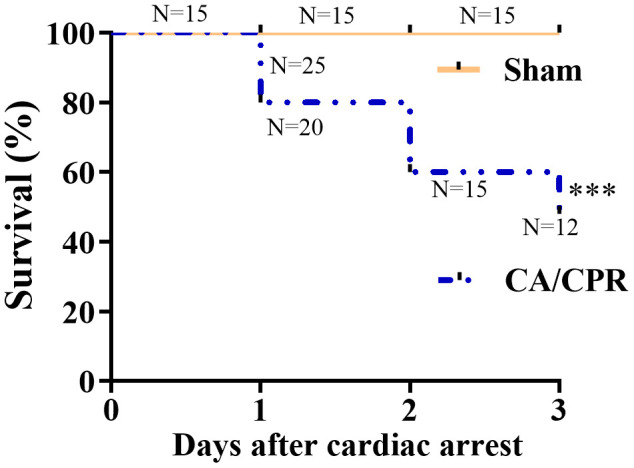
**Survival rates within 3 days in the CA/CPR and sham groups.** The observation period was 72 h after ROSC. (n = 15 for sham group, n = 25 for CA/CPR group). ^***^P < 0.001. ROSC, restoration of spontaneous circulation.

As shown in Figure 2, all 15 mice survived in the sham group on day 3 after sham procedures. Besides, 20, 15 and 12 of 25 mice survived in the CA/CPR group on day 1, 2 and 3 after CA/CPR procedures, respectively. The survival rates were 80%, 60%, and 48%, respectively, which was similar to previously published literature [17, 18]. In our settings, the mice received five minutes of CA, and subsequent CPR performance exhibited a remarkable abnormality in cognitive function (Figure 1E).

### Overview of lncRNA and mRNA expression profiles

**Figure 3:**
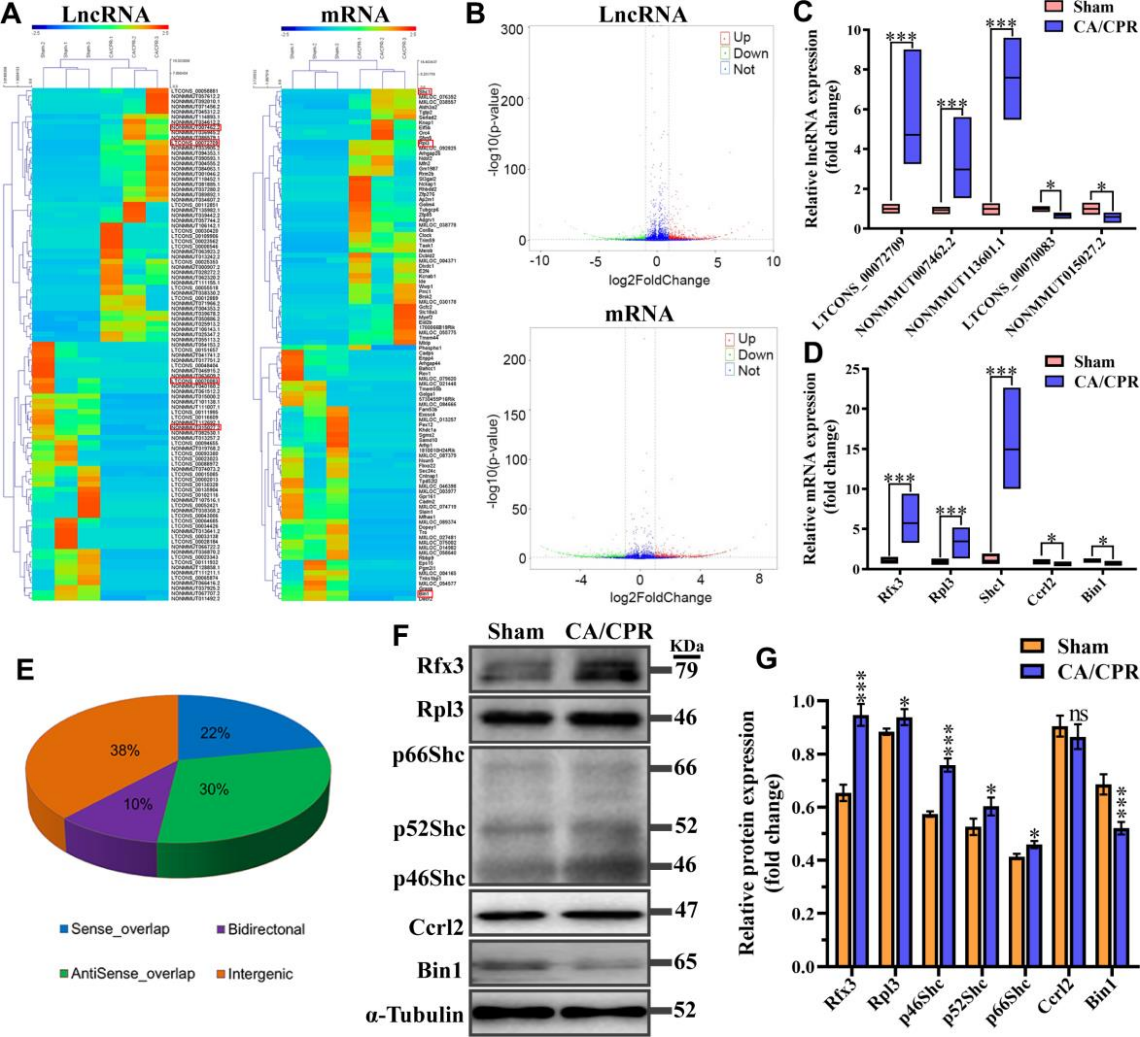
**Expression profiles for lncRNA, mRNA, and encoding protein.** (**A**) Hierarchical clustering of the expression data for lncRNA and mRNA. Each column represents one sample; rows indicate the 100 lncRNAs or mRNAs (50 overexpressed and 50 underexpressed in CA/CPR group). The relative expression of lncRNAs and mRNAs is displayed according to the color scale. Green and red denote downregulation and upregulation, respectively. (**B**) Volcano plots of the lncRNA and mRNA expression level. Up-regulated and down-regulated genes are indicated in red and green dots, respectively. Blue dots denote the same between the two groups. (**C**, **D**) Five pairs of dysregulated lncRNA (**C**) and mRNA (**D**) were validated through qRT-PCR in 10 pairs of CA/CPR and sham samples. (**E**) Subgroup analysis for lncRNAs with differentially expressed depending on genes location and the association with neighboring protein-encoding genes. ^*^P < 0.05, ^***^P < 0.001. (**F**) Expression levels of mRNA encoded proteins were detected by western blot assay in the hippocampus of CA/CPR (right line) and sham (left line) models. (**G**) Quantitative analysis of expression levels of proteins using gray analysis of ImageJ. ^*^P < 0.05, ^**^P < 0.01, ^***^P < 0.001.

To identify novel therapeutic targets for CA/CPR induced cognitive impairment, we applied high-throughput RNA-seq analysis to profile lncRNAs and mRNAs with differentially expressed in the hippocampus of the two groups. Our results showed that in comparison with the sham group, 1920 lncRNAs were differentially expressed in the CA/CPR group, among which 719 were down-regulated, and 1201 were up-regulated; 1162 mRNAs were differentially expressed, among which 480 were down-regulated, and 682 were up-regulated. All of the differentially expressed mRNAs and lncRNAs were shown in the Supplementary Table 1. One hundred lncRNAs and mRNAs with the most significant difference were extracted for clustering analysis between the two groups (Figure 3A). The volcano plots were constructed to reveal systematic variations for lncRNAs and mRNAs expression between CA/CPR and sham groups (Figure 3B). Among these lncRNAs and mRNAs, five pairs of lncRNAs and mRNAs were chosen for further validation by qRT-PCR in an additional 10 pairs of hippocampal tissues from the two groups. The expression levels of lncRNA LTCONS_00072709, NONMMUT113601.1, and NONMMUT007462.2 were up-regulated, while NONMMUT015027.2 and LTCONS_00070083 were down-regulated (Figure 3C). For mRNAs levels, Ccrl2 and Bin1 were down-regulated, while Rfx3, Shc1, and Rpl3 were up-regulated (Figure 3D). The results were consistent between the RNA-seq analysis and qRT-PCR analysis, suggesting the reliability of our RNA-seq data. In addition, structurally different RNAs exhibit diverse mechanisms that lead to different regulatory outcomes. We classified the differentially expressed lncRNAs into four categories (bidirectional, intergenic, antisense, and sense overlap) (Figure 3E). Our data indicated that among these lncRNAs, the intergenic lncRNA category accounted for about 40%, many of which were conserved and functional across mammalian species [19]. The lncRNA and mRNA raw data can be accessed at NCBI SRA (BioProject ID: PRJNA517595).

Considering the expression levels of mRNAs were inconsistent with those of protein result from post-transcriptional regulations. Therefore, the expression of corresponding proteins was validated through western blot assay. Results exhibited that the protein levels of Rfx3, Rpl3 and Shc1 were significantly increased, and that of Bin1 was decreased, which was consistent with the expression levels of mRNA detected by qPCR (Figure 3F, 3G). However, there was no significant change in the protein level of Ccrl2 between sham and CA/CPR mice, which might result from the effects of post-transcriptional regulation.

### Correlation analysis of lncRNA and mRNA in neuron cells

**Figure 4:**
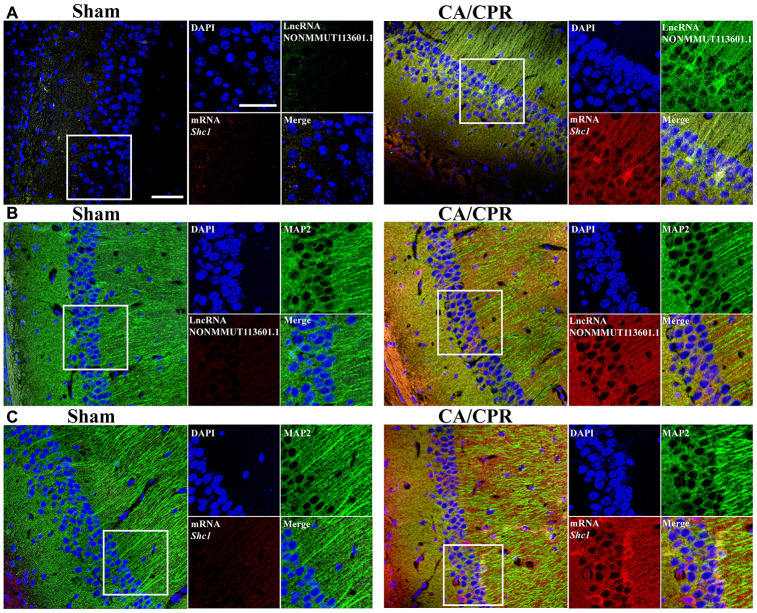
***In situ* hybridization of lncRNA and mRNA in neuron cells.** (**A**) Biotin-labeled lncRNA and digoxigenin-labeled mRNA probes are shown in green and red, respectively. LncRNA and mRNA are co-expressed in CA1 of the hippocampus. (**B**, **C**) The colocation effect of LncRNA (**B**) or mRNA (**C**) with MAP-2 labeled neuron cells indicated these correlations mainly happened in neuron cells of the hippocampus. LncRNA and mRNA were labeled by red fluorescent probes, and he neuron cells were marked using anti-MAP2 antibody and Alexa 488 conjugated anti-rabbit IgG. Scale bar: 50 μm.

According to the investigations of the top five paires of lncRNA and mRNA by qRT-PCR and western bolt assay, we found that lncRNA NONMMUT113601.1 and its corresponding mRNA *Shc1* exhibited the most significant changes and the highest expression levels. Thus, we chose the pair of lncRNA and mRNA of Shc1 to demonstrate the correlations between lncRNA and mRNA, and the specific cell type that it happened in by *in situ* hybridization. As shown in Figure 4., the increased fluorescent signal in CA/CPR group compared with the sham group demonstrated the upregulated *Shc1* expression level (Figure 4A–4C). Meanwhile, the colocation effect of lncRNA NONMMUT113601.1 and *Shc1* indicated the interaction between lncRNA and mRNA of *Shc1* in the molecular regulatory process (Figure 4A). In addition, the increased fluorescent signals of lncRNA and mRNA were observed in anti-MAP2 labeled neuron cells, which demonstrated this correlation occurred mainly in neuron cells (Figure 4B, 4C).

### GO and KEGG pathway analysis

**Figure 5:**
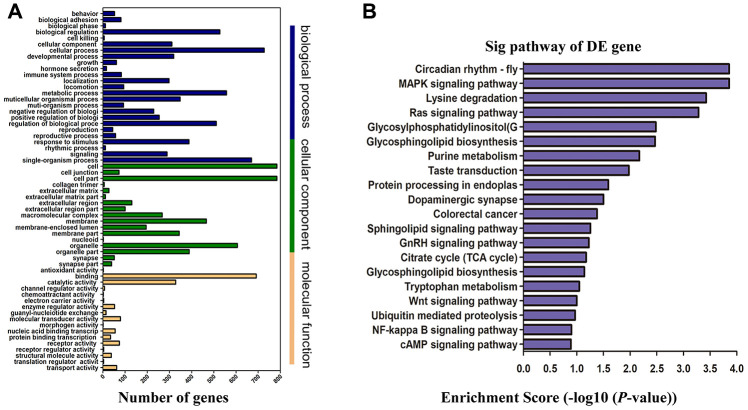
**Functional enrichment analysis of genes with differentially expressed.** (**A**) GO enrichment analysis of lncRNA target genes based on cellular component, biological process, and molecular function. X-axis displays a number of differentially expressed genes; Y-axis indicates GO terms. All GO terms are divided into three ontologies: orange is for molecular function, green is for cellular component, and blue is for biological process. (**B**) Top 20 enrichment scores of KEGG pathway enrichment analysis. The *P*-value indicates the significance of the pathway term correlated to the conditions. The smaller the *P*-value, the more significant the pathway is. The recommended *P*-value cut-off is 0.05. GO, gene ontology; KEGG, Kyoto Encyclopedia of Genes and Genomes.

Annotation analysis of Gene Ontology (GO) was conducted for screened differentially expressed genes targeted by lncRNAs. The results showed that only a few of the differentially expressed mRNAs were related to molecular function, while most of them were associated with mainly cellular components and biological processes (Figure 5A). By targeting differentially expressed mRNAs, the Kyoto Encyclopedia of Genes and Genomes (KEGG) pathway enrichment analysis revealed that they were mainly implicated in GnRH, Wnt, Ras, protein processing, NF-κB and MAPK signaling pathway (20 pathways with the highest enrichment scores were shown in Figure 5B). According to the results from GO and Pathway Enrichment Analysis, we found a large amount of the differentially expressed genes were primarily linked to the signal transduction process of inflammation and apoptosis, which may contribute to the development of cognitive impairment after ROSC.

### Neuroinflammation and neuronal apoptosis were dramatically elevated in the hippocampal tissue post-CA/CPR

**Figure 6:**
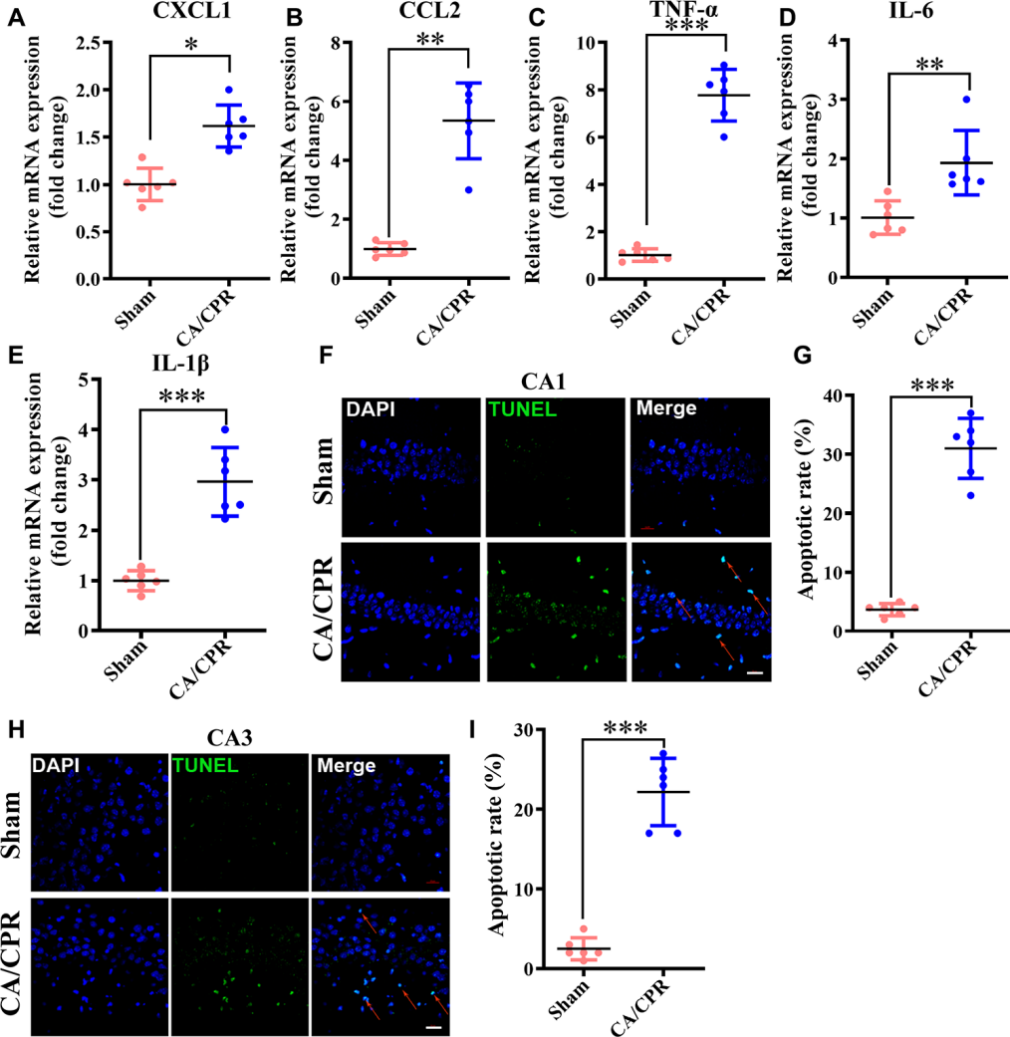
**Neuroinflammation and neuronal apoptosis were markedly augmented in the hippocampus following CA/CPR. Hippocampus was collected at 4h and 72h after resuscitation for the detection of neuronal apoptosis and inflammatory cytokines, respectively.** (**A**–**E**). The levels of CXCL1 (**A**), CCL2 (**B**), TNF-α (**C**), IL-6 (**D**), and IL-1β mRNA (**E**). (**F**). Representative images for TUNEL detection in the hippocampal CA1 subarea. (**G**). The percentage of TUNEL-positive cells in the CA1 region. (**H**). Representative images for TUNEL detection in the hippocampal CA3 subarea. (**I**). The percentage of TUNEL-positive cells in the CA3 region. The cells pointed by the red arrows represent typical TUNEL-positive cells. n = 6 per group. ^**^P < 0.01, ^***^P < 0.001. CXCL1, chemokine (C-X-C motif) ligand 1; CCL2, chemokine (C-C motif) ligand 2; TNF-α, tumor necrosis factor α; IL-1β, interleukin-1β; IL-6, interleukin-6.

The results of functional enrichment analysis suggested that neuroinflammation and neuronal apoptosis may be the main signal transduction pathways leading to cognitive impairment after CA/CPR. To further validate the results, we measured *in vivo* the important factors involved in two items. Our data showed that in comparison with the sham group, the levels of inflammatory chemokine (C-X-C motif) ligand 1 (CXCL1) and chemokine (C-C motif) ligand 2 (CCL2) were substantially enhanced in the CA/CPR group (^**^P < 0.01, ^***^P < 0.001, Figure 6A, 6B). Also, the expression of pro-inflammatory cytokines, including IL-6, TNF-α and IL-1β, presented a similar result (^**^P < 0.01, ^***^P < 0.001, Figure 6C–6E). Moreover, in the early stage of ROSC, the ratio of apoptotic neurons in the hippocampus of the CA/CPR group was markedly enhanced in comparison with the sham group via TUNEL staining (*P* < 0.01, Figure 6F–6I). These findings demonstrated that neuroinflammation and neuronal apoptosis were substantially elevated in the hippocampus after ROSC.

### LncRNA/mRNA co-expression networks

**Figure 7:**
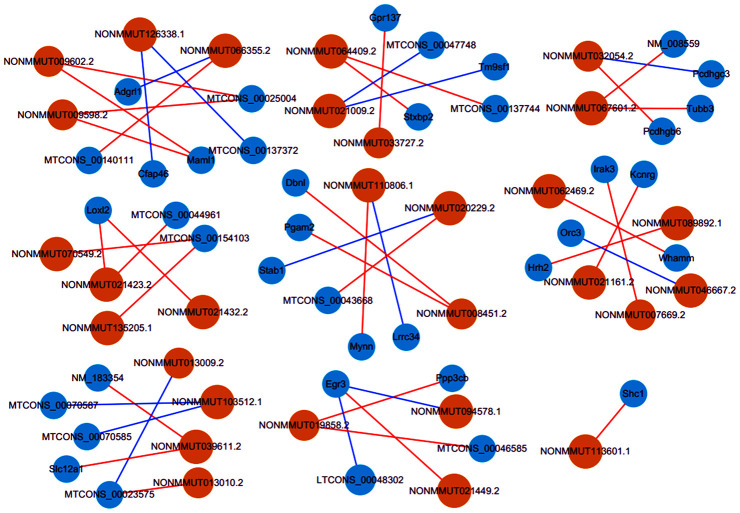
**Co-expression networks of thirty lncRNAs with related mRNAs.** Via rigorous screening progress (PCC > 0.90 or < -0.90, *P* < 0.01), the pairs for co-expressed lncRNA-mRNA were screened. Blue circles represent mRNAs, and orange circles denote lncRNAs. Blue lines show a negative correlation, and red lines indicate a positive correlation. PCC, Pearson correlation coefficient.

Until now, we predict lncRNAs functions based primarily on their co-expression with corresponding coding genes. Accordingly, by a rigorous screening (PCC > 0.90 or < -0.90, and *P* < 0.01), we constructed the co-expression networks for the lncRNA-mRNA with differentially expressed (Figure 7). It was found that lncRNA NONMMUT032054.2 correlated positively with Pcdhgb6 level, while negatively with Pcdhgc3 expression. LncRNA NONMMUT110806.1 was positively related to Mynn level, while negatively related to Lrrc34 expression. LncRNA NONMMUT113601.1 showed a positive correlation to the Shc1 level. Interestingly, co-expressed mRNAs were mainly related to cellular apoptosis, inflammation, or cerebral I/R injury pathways.

### Prediction of the potential key lncRNA-mRNA pairs implicated in cognitive deficits after CA/CPR

The function of lncRNAs is mainly realized by cis-or trans-acting on target genes to date. Combined with lncRNAs-mRNAs co-expression networks, potential targets of lncRNAs were predicted in *cis* (*cis*-prediction) and *trans* (*trans*-prediction). If the distance within 10 kb from each other, lncRNA-mRNA pairs are identified as *cis*-prediction, while trans-prediction screens lncRNA-mRNA pairs by the method of calculating combined energy (When its value is less than 30, it is considered *trans*). Our data showed that lncRNA NONMMUT113601.1 might regulate the expression of *Shc1,* an inflammation, and apoptosis-associated protein, in *cis*. LncRNA NONMMUT041306.2 and NONMMUT115748.1 could regulate gene expression through binding to CCAAT/enhancer-binding proteins (C/EBPs), as a family of inflammation-regulated transcription factors, in *trans*. Previous studies have demonstrated that *Shc1* upregulation in the brain played a vital role in cerebral ischemia-reperfusion injury [20, 21]. Additionally, emerging researches showed that C/EBPs were associated with cognitive impairment in AD and brain injury [22, 23]. Consequently, combined with previous reports, our findings indicated that these lncRNAs-target molecules might play an essential role in cognitive impairment of mice after ROSC.

## DISCUSSION

The current study aimed to explore which lncRNAs may be implicated in the pathogenesis of cognitive impairment after CA/CPR. We established a CA/CPR mouse model as previously reported [17, 18, 24], and identified significantly deregulated lncRNAs and mRNAs (fold change ≥ 2, p < 0.05) in the hippocampal samples. Our results showed that 1162 mRNAs and 1920 lncRNAs were significantly differentially expressed between the two groups. Pathway enrichment analysis of the genes with differential expression suggested that neuronal apoptosis and inflammation may correlate closely with cognitive impairment following CA/CPR. Furthermore, we demonstrated that CA/CPR-induced neuroinflammation and neuronal apoptosis in the hippocampus were significantly increased. Importantly, to the best of our knowledge, we were the first to identify the expression profiles of lncRNA and mRNA in the post-cardiac arrest mouse hippocampus and singled out several key pairs of lncRNA-mRNA, which may play a pivotal role in cognitive impairment in mice after successful resuscitation.

In this study, we demonstrated that mice surviving after 5 minutes of CA displayed remarkable abnormalities in the contextual fear-conditioning test and neurologic score (Figure 1). The survival rate on day 3 after CA/CPR was 48%, which was quantitatively in good agreement with earlier reports [17, 18]. It was indicated that the animal model of CA/CPR in our study was reliable. Then, we applied high-throughput RNA-seq analyses to identify the expression profile of lncRNA and mRNA from the CA/CPR and sham mice in the hippocampal tissue. Previously, Liu *et al*. have investigated lncRNA and mRNA expression profiles in the cerebral cortex from a CA/CPR rat model using the microarray [25]. However, it is widely recognized that the hippocampus is a brain region vital for cognitive functions, such as spatial and episodic memory and learning [26]. Besides, the hippocampus is also considered to be one of the most sensitive areas of neuronal damage after CA or cerebral ischemia [27]. Therefore, we focused on the hippocampus of mice to identify key molecules and related lncRNAs involved in cognitive deficits after CA/CPR.

Recently, increasing evidence has shown that lncRNAs were related to the change of cognitive function [28, 29], but the possible underlying mechanisms remain to be elucidated. As a regulatory factor, lncRNA was proved to be closely associated with cellular inflammation, excessive reactive oxygen species (ROS) and apoptosis [13, 30, 31]. More importantly, plenty of studies suggested that reducing neuronal apoptosis or inflammation could improve cognitive function after CA/CPR [5, 18, 32]. In the context of cognitive deficits after CA/CPR, we found that several inflammation and apoptosis-related genes had abnormal expression levels, which may be potentially targeted by lncRNAs. Moreover, we further demonstrated that *in vivo* neuronal apoptosis and inflammatory cytokines were dramatically enhanced in the CA/CPR mice, consistent with previous researches [18, 32]. Therefore, it could be hypothesized that lncRNAs dysregulation may contribute to cognitive dysfunction following ischemic brain insult by regulating neuronal inflammation or apoptosis.

Nevertheless, it is still unknown which lncRNAs participate in the development of poor neurological outcomes after ROSC. Unfortunately, the functions for most lncRNAs are still not clear to date. Generally, we predict a lncRNA function according to its closely related coding genes. Combining bioinformatics analysis with literature validation, we focused on a potential key inflammation and apoptosis-associated gene *Shc1*, which can encode three proteins, namely p46Shc, p52Shc, and p66Shc [33]. Previous reports showed that p66Shc knockout could prolong the survival time of the mice treated with paraquat [34]. Also, in the mouse model of transient middle cerebral artery occlusion (MCAO), p66Shc knockout could significantly maintain the integrity of the blood-brain barrier, reduce the area of cerebral infarction after I/R, alleviate neurological dysfunction and improve survival rate [20, 21]. Notably, the genetic ablation of the p66Shc adaptor could improve mitochondrial function and reverse cognitive deficits in transgenic mice with Alzheimer's disease (AD) [35]. Intriguing, we identified that in our study, Shc1 expression was also significantly up-regulated following CA/CPR with a remarkable neuroinflammatory response and hippocampal neuronal apoptosis. We, therefore, inferred that Shc1 might be a potential key therapeutic target against cognitive impairment after CA/CPR. Additionally, emerging studies have demonstrated that C/EBPs were significantly elevated in the brain of patients with AD, which could regulate many key elements of energy metabolism and a variety of inflammatory mediators [36]. Also, accumulating evidence showed that C/EBPs upregulation was involved in cognitive impairment in AD and brain injury [22, 23]. Similarly, in our RNA-seq data, we also found that some C/EBPs were up-regulated. These findings suggested that C/EBPs may exert an important role in cognitive impairment after CA/CPR. In our CNC network analysis, the expression of lncRNA NONMMUT113601.1 correlated positively to Shc1 expression and could regulate it in *cis*. LncRNA NONMMUT041306.2 and NONMMUT115748.1 could mediate gene expression by binding to the transcription factors C/EBPs in *trans*. These data suggested that these potential key lncRNAs in our study may regulate the corresponding target genes expression participating in the formation and development of cognitive impairment after CA/CPR.

Several limitations of the present research must be acknowledged. Firstly, we only screened differentially expressed genes on day 3 after successful resuscitation in mice with cognitive impairment when we detected marked learning and memory dysfunction. It is admitted that we could not determine whether these genes expression would change over a longer period. Secondly, the relatively small sample size was used in the study. Our conclusions are required to be further verified in a future study with larger sample size. Thirdly, we only explored the changes of lncRNAs and mRNAs in the whole hippocampus after CA/CPR Further experiments need to clarify the changes of RNAs in different subregions of the hippocampus (the CA1, CA3, and dentate gyrus regions). Finally, the roles of lncRNAs and target mRNAs axis in the pathogenesis of cognitive deficits after CA/CPR are primarily based on bioinformatics analysis and prediction, therefore whether these genes are the authentic triggers for cognitive impairment after CA/CPR needs further experimental verification.

In conclusion, through high-throughput RNA-seq, we have identified the differentially expressed lncRNAs and mRNAs in the hippocampus following CA/CPR. Further, the correlated pathways and functions of genes with differentially expressed were assessed by KEGG and GO analyses. The lncRNA-mRNA expression networks were established to analyze and predict lncRNAs functions. In the end, we singled out several key pairs of lncRNA and target molecule via bioinformatics analysis and literature validation and speculated that they might mediate cognitive deficits after CA/CPR. However, further researches are needed to validate the correlation between lncRNAs and target molecules and whether the lncRNAs and target molecules axis act as a vital role in cognitive deficits formation and development after CA/CPR.

## MATERIALS AND METHODS

### Animals

Aged 2-3 months C57BL/6J male mice (weight, 22–25 g) were obtained from Dashuo Company, Sichuan Province (Chengdu, China). Mice were raised under standardized conditions with a 12-h light-dark cycle, and allowed free access to food and water. Our protocol approved by the Animal Care and Use Committee of Sichuan University was in agreement with the National Institute of Health Guidelines for the Care and Use of Laboratory Animals. Mice were randomly divided into 2 groups: Sham (n=15) and CA/CPR group (n=25). Investigators dealing with the animals knew the grouping and collected samples, and other researchers blinded to the specific treatment were responsible for analyzing data.

### CA/CPR procedure

CA/CPR in mice was performed according to previously reported with minor modifications [18, 37, 38]. Briefly, mice were anesthetized with the administration of a mixture of 100 μg/g ketamine and 10 μg/g xylazine (i.p. injection), then intubated with a 22-gauge intubation cannula and connected to the ventilator (Harvard Bioscience, USA). The parameters of mechanical ventilation were as follows: respiratory frequency of 130 beats per minute (bpm), a fraction of inspired oxygen (FiO_2_) of 40%, and tidal volume of 10 μl/g. Throughout the whole procedure, an electrocardiogram (ECG) needle probe was monitored. The right external jugular vein was inserted into a heparinized micro-polyethylene catheter (PE10) for drug administration under a microscope. For blood pressure measurement, another heparinized PE10 was introduced into the right femoral artery of mice.

Potassium chloride (0.08 mg/g) was administered through the PE10 catheter to induce CA. Meanwhile, the endotracheal tube was disconnected. CA was determined by the appearance of asystole on the electrocardiogram and loss of arterial pressure. After 5 minutes of CA, CPR was initiated with the right index finger at a frequency of 350–400 bpm. Meanwhile, the ventilation system was reconstructed with FiO_2_ of 1.0, respiratory rate of 150 bpm, and 0.4 ug/g epinephrine was injected into the PE10. ROSC was defined as a mean arterial pressure >40 mm Hg for at least 1 minute as well as the recovery of sinus rhythm in ECG. During the procedure, core body temperature was maintained at 37±0.5ºC using a heating blanket and heating lamp. If spontaneous circulation could not be restored within 2.5 min, CPR was abandoned. Twenty minutes after ROSC, the ventilation frequency decreased to 130 bpm and FiO_2_ to 0.4. At 1 hour following ROSC, the PE10 catheter was removed, and then the incision was sutured. At 2 hours after ROSC, mechanical ventilation was broken off. Finally, 0.2% ropivacaine 50 μl was subcutaneously administered to mitigate post-operative pain. The animals were returned to their original cages maintained at 35 °C with a heated water blanket, free access to water, and received soft food for three days after CPR. For the sham group, mice were only subjected to anesthesia, mechanical ventilation, the PE10 catheter insertion, skin incision as well as postoperative analgesia.

### Neurological function score

Based on the previously published literature [24, 39], neurological scores were assessed according to the following six categories: level of consciousness, movement, respiration model, coordination, corneal reflex, and righting reflex. In each item, mice could receive different scores, including 0, 1, or 2, with a maximum score of 12-point determined by conditions detailed in Table 1. Investigators who performed the neurologic score were blinded to mice grouping.

**Table 1:** Neurological function scoring system used in this study.

**Category**	**Scoring**
Level of consciousness	0 = reaction to pinching of tail
	1 = Poor response to tail pinch
	2 = Normal response to tail pinch
Respiration pattern	0 = Irregular breathing pattern
	1 = Decreased breathing frequency, normal pattern
	2 = Normal breathing frequency and pattern
Corneal reflex	0 = No blinking
	1 = Sluggish blinking
	2 = Normal blinking
Righting reflex	0 = No turning attempts
	1 = Sluggish turning
	2 = Turns over spontaneously and quickly
coordination	0 = No movement
	1 = Moderate ataxia
	2 = Normal coordination
Movement/activity	0 = No spontaneous movement
	1 = Sluggish movement
	2 = Normal movement

### Fear-conditioning (FCT) test

The FCT test was a classic approach applied to assess the memory and learning ability of rodents. According to the previously described protocol, conditioned fear training was first performed on mice one day before the CA/CPR procedure [40]. The mouse was put into a quiet behavioral test room for 30 minutes and then transported into a conditioning chamber (Ugo Basile, Italy). Subsequently, the mouse was given 100 s to explore the chamber, followed by a 2-Hz pulsating tone (75 dB, 3,600 Hz) for 20 s. Before the end of the sound, a foot shock (0.75 mA for 2 s) was given. This process was repeated once, and mice were moved out 30 s later. The conditional fear training time was 274 s in total. On day 3 after CA/CPR, the contextual test was performed. As before, the mouse was placed into the same training room for 30 minutes and then transferred to the original conditioning chamber for 274 s, where the environment remained unchanged, but the cue tone or the foot shock was not given. We evaluated the cognitive function in mice by calculating the percentage of freezing time during the 274 s.

### Quantitative real-time PCR (qRT-PCR)

As previously described, qRT-PCR was conducted for quantification of cytokines mRNA expression and validation of the selected lncRNAs and mRNAs in the hippocampal tissue [25, 41]. The hippocampus of the mouse was collected on day 3 after CA/CPR procedure. With TRIzol reagent (Invitrogen, United States), the total hippocampal RNA was extracted, followed by reversely transcribed utilizing a PrimeScript RT reagent Kit with gDNA Eraser (PerfectReal Time) (Bio-Rad, USA) to synthesize cDNA in accordance with the manufacturer’s instructions. The Qubit® Assay Kit (Life Technologies, United States) and Nano 6000 Assay Kit (Agilent Technologies, United States) were used to measure the concentration of total RNA and assess RNA integrity, respectively. qRT-PCR was conducted through a Mastercycler ep realplex RT-PCR system (Eppendorf, NY). 18S mRNA was used as a reference. The primer pairs for this study were displayed in Table 2.

**Table 2:** The primers used for qRT-PCR analysis in the study.

	**Forward(5'-3')**	**Reverse(5'-3')**
LTCONS_00072709	CTGCCTCCTTATCTAGGTTATGGT	GTGTAGCCTTTTTAGTCCGAGC
NONMMUT007462.2	GTCTACACCAGAGGTCAGAGTC	GCAGAGCAGCTAAATGCACC
NONMMUT015027.2	AGAGATCTGCCTGCCTCTCC	CCCATCTAGGTTGGGCATGG
LTCONS_00070083	AGCCATGAGCAACCATCCTC	TACACTGAGCCCAGAAGTGC
NONMMUT113601.1	ATCTTTTCTTTAGCCGCATACTCC	GCTTCCTGACCCTCATACTTCACT
Ccrl2	CCGGGAGAGGGAGAGATGAA	CCATCGGAGGCTGTCCTTG
Bin1	GTGAAGCAACCTCCAGCTCT	TTCTTCACACTCGGGAAGGC
Rp13	ATATAGCCTGCACTGGCTCCT	TGGTTACCCCTTTGTACCCT
Rfx3	CGCCATAGTCACCGTAGTCC	CCATTGCAGATGGCTGTTGAG
Shc1	TAGTGAGGCCGGAAGTGAGT	TTGAAGCGCAACTCAAAGGC
TNF-α	CTGTGAAGGGAATGGGTGTT	CAGGGAAGAATCTGGAAAGGTC
IL-1β	TGCCACCTTTTGACAGTGATG	CATCTCGGAGCCTGTAGTGC
IL-6	TGAGAAAAGAGTTGTGCAATGG	GGAGAGCATTGGAAATTGGGG
CCL2	CCCCAAGAAGGAATGGGTCC	GTGCTGAAGACCTTAGGGCA
CXCL1	GCACCCAAACCGAAGTCA	AAGCCAGCGTTCACCAGA
18S	TTGACTCAACACGGGAAACC	AGACAAATCGCTCCACCAAC

### Western blot

Protein expression corresponding to the selected mRNA was further analyzed by western blot assay in hippocampal tissue. The hippocampus of the mouse was collected on day 3 after CA/CPR procedure and frozen by liquid nitrogen. The samples were suspended into brain lysis buffer containing 50 mM Tris (pH 8.0), 150 mM NaCl, and protease inhibitors and further sonicated for 30 s at 10% amplitude (ultrasonic cell crusher, Ningbo) to dissolve the total proteins sufficiently. The total proteins in the supernatant were collected by being centrifugated at 16000 g for 20 min. Protein concentrations of these supernatants were determined using BCA assay. Then the proteins were separated in polyacrylamide gels and further transferred to poly(vinylidene difluoride) membrane (0.45 mm, Millipore, Bedford, MA, USA). The membranes were blocked with 5% nonfat milk (BD Biosciences) in Tris-buffered saline with 0.1% Tween (TBST) for 1 h at room temperature and then incubated with anti-Rfx3 (Rabbit mAb, Proteintech^TM^, 14784-1-AP), anti-Rpl3 (Rabbit mAb, Proteintech^TM^, 11005-1-AP), anti-Shc1 (Rabbit mAb, Proteintech^TM^, 10054-1-AP), anti-Ccrl2 (Rabbit mAb, Proteintech^TM^, 13387-1-AP), anti-Bin1 (Rabbit mAb, Abcam, ab182562), anti-α-tubulin (Rabbit mAb, Proteintech^TM^, 11224-1-AP) overnight at 4°C. Afterward, the membranes were incubated with horseradish peroxidase-conjugated anti-Rabbit IgG (Goat mAb, Abcam, ab6721) for 1 h at room temperature in TBST containing 5% nonfat milk. The bolts were finally performed using the Enhanced Chemiluminescence Kit (Thermo Pierce, Waltham, MA, USA) and were visualized by Image Lab (BIO-RAD, USA).

### In situ hybridization

Brain tissues of CA/CPR and sham mice were dissected and fixed in 4% paraformaldehyde solution for 48 h, followed by embedded in paraffin. Paraformaldehyde brains were sectioned at 10 μm thickness by a microtome. Paraffin slice samples were dewaxed by xylene (3 times, 5 min per time) and ethanol (100%, 85%, and 70% serial rehydration, 3 min), respectively. After being washed by PBS, the samples were processed by 0.2 M HCl for 20 min, 0.5% Triton for 20 min, 20 μg/mL Proteinase K for 20 min, and 3% hydrogen peroxide for 20 min. Each slide was rinsed five times in distilled water. After air-drying, the samples were incubated with biotin-labeled lncRNA NONMMUT113601.1 and digoxigenin-labeled *Shc1* mRNA probes in hybridization buffer (1: 1: 50, v/v/v). After hybridization at 37 °C for 24 h, the unbound and excessive probes were removed by twice washes in 2 × saline sodium citrate (SSC) buffer for 5 min, followed by blocked in 3% BSA for 30 min at room temperature. Then the RNA probes were detected by anti-digoxigenin (sheep Ab, Merck, 11333089001) and anti-avidin (mouse mAb, Santa, sc51760), followed by labeled with FITC labeled anti-mouse IgG (Abcam, ab6724) and Alexa 647 labeled anti-sheep IgG (Abcam, ab150179). The unbound antibody was removed by washing the samples twice in 2 × SSC buffer for 5 min. Nuclei were dyed by DAPI before being observed on a laser confocal microscope (N-SIM S, Nikon, Japan).

The same process was performed as described above to label the lncRNA or mRNA separately. Afterward, the samples were incubated with anti-MAP2 (Rabbit mAb, abcam, ab254264) and Alexa 488 labeled anti-Rab IgG (Goat mAb, Abcam, ab150077) to label the neuron cells. Nuclei were dyed by DAPI before imaging on a laser confocal microscope.

### TUNEL assay

Terminal deoxynucleotidyl transferase dUTP nick end labeling (TUNEL) assay was used to detect neuronal apoptosis [42]. In short, experimental mice were anesthetized and then transcardially perfused with 25 ml 0.9% saline and 20 ml 4% paraformaldehyde, respectively. Subsequently, the brain tissues were harvested and immersed in 10% paraformaldehyde for 48 h, dehydrated, and dissected into 10-μm-thick. To distinguish apoptotic cells, we utilized a Cell Death Detection Kit (Roche, Germany) and 4', 6-diamidino-2-phenylindole (DAPI) staining in line with the manufacturer’s protocol. Neurons co-localized to display TUNEL signal, and DAPI were identified as apoptotic cells. Apoptotic cells counting were conducted, as we have previously reported [43].

### RNA sequencing (RNA-seq) and data analysis

The transcriptome analysis based on RNA-seq was carried out by the Beijing Genomics Institute (Shenzhen, China) using the BGISEQ-500 platform (BGI-Shenzhen, China). Three biological replicates of CA/CPR and sham hippocampal samples were randomly selected for lncRNA and mRNA sequencing. The rRNA-depleted RNA was applied according to the manufacturer’s recommendations in order to obtain the sequencing libraries of lncRNAs and mRNAs. By utilizing FASTX-Toolkit (v 0.0.13), raw data of the fastq format were first processed. Clean data were acquired by removing reads, including reads containing poly-N from raw data, an adapter, and low-quality reads. Additionally, GC content of the clean data, Q30, and Q20 were analyzed. Between the two groups, lncRNA and mRNA with differentially expressed, was identified through fold change and *P*-value, which was calculated utilizing a student's t-test. To further classify lncRNAs and mRNAs, the analysis of hierarchical clustering was employed based on their expression levels by the program MeV.

### Functional enrichment analysis

Functional enrichment analysis is a method for identifying enriched genes for molecular functions, biological processes, and pathways in the datasets of interest. Gene ontology (GO) database analysis (http://www.geneontology.org) was carried out to annotate genes in terms of biological processes, molecular functions, and cell components [44]. KEGG database (http://www.genome.jp/kegg/) analysis of the coding genes related to differentially expressed lncRNAs was also conducted by KOBAS software using a hypergeometric test [45]. GO terms or KEGG pathways with *P*-value<0.05 were regarded as significantly enriched. Benjamini-Hochberg correction with a false discovery rate was utilized for correcting the *P*-value.

### Co-expression network construction

Pearson correlation coefficient (PCC) between the differentially expressed lncRNAs and mRNAs was calculated. LncRNAs and mRNAs with PCC > 0.90 or < -0.90, *P* < 0.01 were selected for drawing the networks of lncRNA-mRNA using the program Cytoscape [46].

### Statistical analysis

All data were presented as means ± SEM. Two variables of microarray data were analyzed and compared using Student’s t-test. The threshold for lncRNAs and mRNAs with differentially expressed was set as fold change ≥ 2.0, *P*-value < 0.05. For other data analyses, *P* < 0.05 was regarded to have a statistical difference between the two groups utilizing Unpaired 2-tailed Student’s t-test via Graphpad prism 7 software (GraphPad, CA).

## Supplementary Material

Supplementary Table 1
