# Correction for: RNA-seq analysis of the key long noncoding RNAs and mRNAs related to cognitive impairment after cardiac arrest and cardiopulmonary resuscitation

**DOI:** 10.18632/aging.205612

**Published:** 2024-02-15

**Authors:** Chan Chen, Changliang Liu, Zhendong Niu, Ming Li, Yuhan Zhang, Rui Gao, Hai Chen, Qiao Wang, Shu Zhang, Ronghua Zhou, Lu Gan, Zheng Zhang, Tao Zhu, Hai Yu, Jin Liu

**Affiliations:** 1 Department of Anesthesiology, Laboratory of Anesthesia and Critical Care Medicine, Translational Neuroscience Center, West China Hospital, Sichuan University and The Research Units of West China, Chinese Academy of Medical Sciences, Chengdu, Sichuan, China; 2 Department of Emergency Medicine, West China Hospital, Sichuan University, Chengdu, Sichuan, China

**Keywords:** long noncoding RNA, cardiac arrest, cardiopulmonary resuscitation, RNA sequencing, signal pathway

**This article has been corrected:** The authors recently found an error in **Figure 4B**, “In situ hybridization of lncRNA and mRNA in neuron cells.” The image of the hippocampal CA1 region in the Sham group depicting colocalization of lncRNA with MAP-2-labeled neuronal cells was inadvertently substituted with an image from the CA/CPR group from the same experiment. That incorrect image was replaced with the correct Sham group image from the initial set of experiments. The authors stated that this alteration does not affect the results or conclusion of this work and apologize for any inconvenience.

The corrected **Figure 4** is presented below.

**Figure 4:**
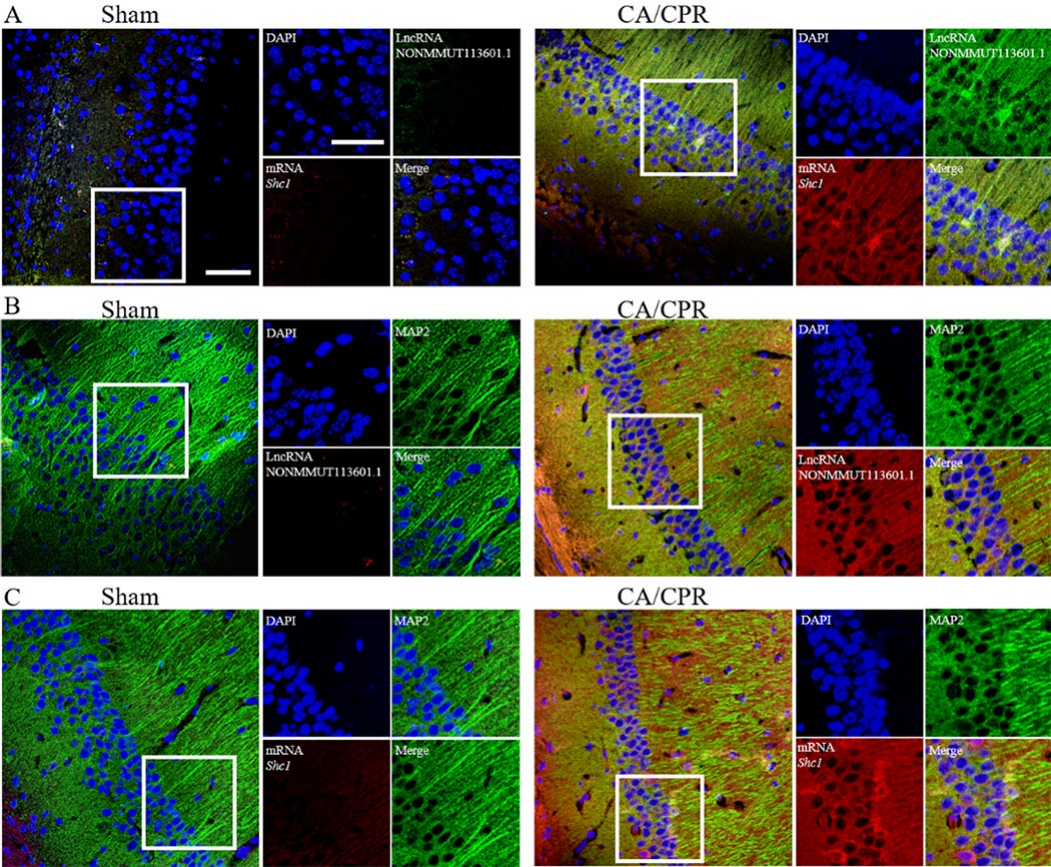
***In situ* hybridization of lncRNA and mRNA in neuron cells. **(**A**) Biotin-labeled lncRNA and digoxigenin-labeled mRNA probes are shown in green and red, respectively. LncRNA and mRNA are co-expressed in CA1 of the hippocampus. (**B, C**) The colocation effect of LncRNA (**B**) or mRNA (**C**) with MAP-2 labeled neuron cells indicated these correlations mainly happened in neuron cells of the hippocampus. LncRNA and mRNA were labeled by red fluorescent probes, and the neuron cells were marked using anti-MAP2 antibody and Alexa 488 conjugated anti-rabbit IgG. Scale bar: 50 μm.

