# Supplementary material for: RNA-seq analysis of the key long noncoding RNAs and mRNAs related to cognitive impairment after cardiac arrest and cardiopulmonary resuscitation
Source: Aging (Albany NY). 2020 Jul 21;12(14):14490–505. doi: 10.18632/aging.103495 (PMC7425488; doi:10.18632/aging.103495)
Supplement: Supplementary Table 1 [file aging-12-103495-s001..docx]

**Supplementary Table 1.** CA/CPR_vs_Sham 2.0 fold up/down regulated lncRNAs and mRNAs (*P* < 0.05)

| 1. **CA/CPR_vs_Sham 2.0 fold up/down regulated lncRNAs (*P* < 0.05)** | | | | | | | | | | |
| --- | --- | --- | --- | --- | --- | --- | --- | --- | --- | --- |
| Gene_ID | Transcriptome_ID | | Length | | NSSm Readnum | | NSCA-CPR Readnum | Fold change | | Up-Down-Regulation |
| LXLOC_014684 | LTCONS_00025353 | | 4091 | | 0 | | 71.7 | 18.09490777 | | Up |
| LXLOC_017555 | LTCONS_00030420 | | 2358 | | 0 | | 23.23 | 15.81379349 | | Up |
| LXLOC_062885 | LTCONS_00109906 | | 1914 | | 0 | | 111.97 | 15.70803967 | | Up |
| LXLOC_041507 | LTCONS_00072709 | | 3190 | | 0 | | 82.61 | 14.83059186 | | Up |
| LXLOC_031985 | LTCONS_00055518 | | 2137 | | 0 | | 57.96 | 13.80807418 | | Up |
| NONMMUG072037.1 | NONMMUT114893.1 | | 1563 | | 0 | | 7 | 13.38181518 | | Up |
| LXLOC_033931 | LTCONS_00058881 | | 1153 | | 0 | | 41.79 | 12.86427451 | | Up |
| NONMMUG039454.2 | NONMMUG072037.1 | | 1799 | | 0 | | 40.64 | 12.7837598 | | Up |
| LXLOC_013645 | LTCONS_00023562 | | 3728 | | 0 | | 40.43 | 12.7688114 | | Up |
| LXLOC_064458 | LTCONS_00112851 | | 609.5 | | 0 | | 64.9 | 12.70700432 | | Up |
| NONMMUG066337.1 | NONMMUT106142.1 | | 2708 | | 7 | | 514 | 12.49064206 | | Up |
| NONMMUG035784.2 | NONMMUT057612.2 | | 1224 | | 0 | | 6.6 | 12.18289104 | | Up |
| LXLOC_007603 | LTCONS_00012889 | | 3367 | | 0 | | 30.49 | 11.95463129 | | Up |
| NONMMUG020828.2 | NONMMUT033905.2 | | 2115 | | 0 | | 28.5 | 11.75988283 | | Up |
| NONMMUG024550.2 | NONMMUT039678.2 | | 1272 | | 0 | | 28.08 | 11.71704487 | | Up |
| NONMMUG056462.1 | NONMMUT090593.1 | | 427 | | 0 | | 5.01 | 11.69158639 | | Up |
| NONMMUG004742.2 | NONMMUT007462.2 | | 2136 | | 0 | | 8.79 | 11.31369151 | | Up |
| NONMMUG021281.2 | NONMMUT034612.2 | | 1737 | | 0 | | 20.52 | 10.81202046 | | Up |
| NONMMUG023674.2 | NONMMUT038330.2 | | 3435 | | 0 | | 19.46 | 10.65898241 | | Up |
| NONMMUG044271.2 | NONMMUT071456.2 | | 4301 | | 0 | | 19 | 10.58995783 | | Up |
| LXLOC_005064 | LTCONS_00008546 | | 1935 | | 0 | | 16.63 | 10.20553533 | | Up |
| NONMMUG000615.2 | NONMMUT000907.2 | | 890 | | 0 | | 5.44 | 10.0941028 | | Up |
| NONMMUG057368.1 | NONMMUT092010.1 | | 1619.83 | | 0 | | 16 | 10.0941028 | | Up |
| NONMMUG085776.1 | NONMMUT135982.1 | | 6968 | | 0 | | 16 | 10.0941028 | | Up |
| NONMMUG022979.2 | NONMMUT037280.2 | | 2788 | | 0 | | 15.53 | 10.00807465 | | Up |
| NONMMUG022742.2 | NONMMUT036945.2 | | 4100 | | 0 | | 15.31 | 9.96690756 | | Up |
| NONMMUG000699.2 | NONMMUT001046.2 | | 2592 | | 0 | | 15.06 | 9.919402534 | | Up |
| NONMMUG032496.2 | NONMMUT118452.1 | | 3967 | | 0 | | 15 | 9.907883996 | | Up |
| NONMMUG002930.2 | NONMMUT004555.2 | | 1996 | | 0 | | 14.53 | 9.8160284 | | Up |
| NONMMUG044596.2 | NONMMUT071966.2 | | 2742 | | 0 | | 14.52 | 9.814041901 | | Up |
| NONMMUG016041.2 | NONMMUT025913.2 | | 2796 | | 0 | | 19.8 | 9.738106201 | | Up |
| NONMMUG024379.2 | NONMMUT039442.2 | | 3079 | | 0 | | 8.7 | 9.623845986 | | Up |
| NONMMUG031545.2 | NONMMUT050886.2 | | 736 | | 1 | | 27 | 9.603877809 | | Up |
| NONMMUG052346.1 | NONMMUT084063.1 | | 1406 | | 0 | | 13 | 9.494982241 | | Up |
| NONMMUG055982.1 | NONMMUT089892.1 | | 628 | | 0 | | 12 | 9.264027806 | | Up |
| NONMMUG038437.2 | NONMMUT062320.2 | | 3902 | | 0 | | 12 | 9.264027806 | | Up |
| NONMMUG058900.1 | NONMMUT094353.1 | | 666 | | 0 | | 12 | 9.264027806 | | Up |
| NONMMUG027963.2 | NONMMUT045312.2 | | 1096 | | 0 | | 12 | 9.264027806 | | Up |
| NONMMUG015689.2 | NONMMUT025347.2 | | 2317 | | 1 | | 23 | 9.141226717 | | Up |
| NONMMUG035869.2 | NONMMUT057744.2 | | 310 | | 0 | | 11.27 | 9.082934025 | | Up |
| NONMMUG066338.1 | NONMMUT106143.1 | | 1476 | | 0 | | 11 | 9.012966042 | | Up |
| NONMMUG008403.2 | NONMMUT013242.2 | | 2910 | | 0 | | 11 | 9.012966042 | | Up |
| NONMMUG050981.1 | NONMMUT081885.1 | | 891 | | 0 | | 11 | 9.012966042 | | Up |
| NONMMUG053916.1 | NONMMUT086579.1 | | 2835 | | 0 | | 11 | 9.012966042 | | Up |
| NONMMUG002814.2 | NONMMUT004353.2 | | 773.84 | | 1 | | 22 | 9.012966042 | | Up |
| NONMMUG017502.2 | NONMMUT028272.2 | | 533 | | 0 | | 6.49 | 8.964470949 | | Up |
| NONMMUG021279.2 | NONMMUT034607.2 | | 997 | | 9.07 | | 193.55 | 8.925022698 | | Up |
| NONMMUG069534.1 | NONMMUT111155.1 | | 635 | | 0 | | 10.51 | 8.881484333 | | Up |
| NONMMUG034214.2 | NONMMUT055113.2 | | 674 | | 0 | | 5.6 | 8.737958994 | | Up |
| NONMMUG022692.2 | NONMMUT036870.2 | | 1621 | | 0 | | 10 | 8.737958994 | | Up |
| LXLOC_027669 | LTCONS_00047954 | | 2755.59 | | 1.46 | | 28.68 | 8.686112304 | | Up |
| NONMMUG016663.2 | NONMMUT026869.2 | | 1816 | | 0 | | 4.75 | 8.589957831 | | Up |
| LXLOC_025008 | LTCONS_00043582 | | 624 | | 0 | | 9.31 | 8.53166514 | | Up |
| NONMMUG038010.2 | NONMMUT061210.2 | | 2069 | | 0 | | 5.78 | 8.443954169 | | Up |
| NONMMUG032055.2 | NONMMUT051685.2 | | 1652 | | 0 | | 9 | 8.433952807 | | Up |
| NONMMUG067473.1 | NONMMUT107912.1 | | 2661 | | 0 | | 9 | 8.433952807 | | Up |
| NONMMUG063392.1 | NONMMUT101317.1 | | 247 | | 0 | | 9 | 8.433952807 | | Up |
| NONMMUG073687.1 | NONMMUT117473.1 | | 788 | | 0 | | 8.99 | 8.430745036 | | Up |
| LXLOC_070719 | LTCONS_00123996 | | 2508 | | 0 | | 8.76 | 8.355964544 | | Up |
| LXLOC_000055 | LTCONS_00000096 | | 2804 | | 0 | | 14.35 | 8.352668838 | | Up |
| NONMMUG017156.2 | NONMMUT027686.2 | | 3606 | | 1 | | 17.34 | 8.326166791 | | Up |
| NONMMUG049396.1 | NONMMUT079375.1 | | 6549 | | 0 | | 8.66 | 8.322836854 | | Up |
| NONMMUG000540.2 | NONMMUT000799.2 | | 4563 | | 0 | | 8.45 | 8.252005487 | | Up |
| LXLOC_053175 | LTCONS_00093453 | | 1161 | | 0 | | 8.41 | 8.238314405 | | Up |
| NONMMUG008676.2 | NONMMUT013725.2 | | 1263 | | 0 | | 8 | 8.094102804 | | Up |
| NONMMUG012469.2 | NONMMUT020106.2 | | 3345 | | 0 | | 8 | 8.094102804 | | Up |
| NONMMUG083351.1 | NONMMUT132380.1 | | 2682 | | 0 | | 8 | 8.094102804 | | Up |
| NONMMUG041952.2 | NONMMUT067757.2 | | 2818.88 | | 0 | | 8 | 8.094102804 | | Up |
| NONMMUG085764.1 | NONMMUT135970.1 | | 4243 | | 0 | | 8 | 8.094102804 | | Up |
| NONMMUG067478.1 | NONMMUT107919.1 | | 1410 | | 0 | | 8 | 8.094102804 | | Up |
| NONMMUG040122.2 | NONMMUT064963.2 | | 2437.38 | | 0 | | 8 | 8.094102804 | | Up |
| NONMMUG024089.2 | NONMMUT039003.2 | | 3949 | | 0 | | 8 | 8.094102804 | | Up |
| NONMMUG047498.1 | NONMMUT076566.1 | | 820 | | 0 | | 8 | 8.094102804 | | Up |
| NONMMUG084005.1 | NONMMUT133326.1 | | 848 | | 0 | | 8 | 8.094102804 | | Up |
| NONMMUG070528.1 | NONMMUT112638.1 | | 975 | | 0 | | 8 | 8.094102804 | | Up |
| NONMMUG011441.2 | NONMMUT018381.2 | | 1876 | | 0 | | 8 | 8.094102804 | | Up |
| NONMMUG002302.2 | NONMMUT003584.2 | | 3911 | | 0 | | 8 | 8.094102804 | | Up |
| NONMMUG085397.1 | NONMMUT135450.1 | | 1033 | | 0 | | 8 | 8.094102804 | | Up |
| NONMMUG020075.2 | NONMMUT032579.2 | | 2990 | | 0 | | 8 | 8.094102804 | | Up |
| NONMMUG020817.2 | NONMMUT033892.2 | | 1380.3 | | 0 | | 8 | 8.094102804 | | Up |
| NONMMUG081654.1 | NONMMUT129965.1 | | 1552 | | 1 | | 16 | 8.094102804 | | Up |
| NONMMUG007389.2 | NONMMUT011672.2 | | 1233 | | 1.32 | | 20.81 | 8.051437065 | | Up |
| NONMMUG019832.2 | NONMMUT032224.2 | | 1357 | | 0 | | 7.73 | 7.995039633 | | Up |
| NONMMUG036499.2 | NONMMUT058712.2 | | 2973 | | 0 | | 7.69 | 7.980070001 | | Up |
| NONMMUG033133.2 | NONMMUT053361.2 | | 2008 | | 0 | | 5.52 | 7.971301715 | | Up |
| NONMMUG031670.2 | NONMMUT051114.2 | | 938 | | 0 | | 7.53 | 7.919402534 | | Up |
| LXLOC_033482 | LTCONS_00058222 | | 882 | | 0 | | 7.47 | 7.89631929 | | Up |
| NONMMUG070640.1 | NONMMUT112755.1 | | 646 | | 0 | | 7.3 | 7.829895732 | | Up |
| NONMMUG004147.2 | NONMMUT006435.2 | | 1143 | | 0 | | 7.09 | 7.745674059 | | Up |
| NONMMUG015661.2 | NONMMUT025303.2 | | 1074 | | 0 | | 7.07 | 7.737523235 | | Up |
| NONMMUG083985.1 | NONMMUT133302.1 | | 995 | | 0 | | 7 | 7.708812649 | | Up |
| NONMMUG014382.2 | NONMMUT023265.2 | | 2766 | | 0 | | 7 | 7.708812649 | | Up |
| NONMMUG021112.2 | NONMMUT034341.2 | | 2285 | | 0 | | 7 | 7.708812649 | | Up |
| NONMMUG003171.2 | NONMMUT004938.2 | | 2184 | | 0 | | 7 | 7.708812649 | | Up |
| NONMMUG051056.1 | NONMMUT082011.1 | | 3170 | | 0 | | 7 | 7.708812649 | | Up |
| NONMMUG047085.1 | NONMMUT075881.1 | | 2536 | | 0 | | 7 | 7.708812649 | | Up |
| NONMMUG038854.2 | NONMMUT062962.2 | | 631 | | 0 | | 7 | 7.708812649 | | Up |
| NONMMUG082828.1 | NONMMUT131577.1 | | 367 | | 0 | | 7 | 7.708812649 | | Up |
| NONMMUG072817.1 | NONMMUT116153.1 | | 963 | | 0 | | 7 | 7.708812649 | | Up |
| NONMMUG017279.2 | NONMMUT027887.2 | | 1620 | | 0 | | 7 | 7.708812649 | | Up |
| NONMMUG046976.1 | NONMMUT075727.1 | | 625 | | 0 | | 7 | 7.708812649 | | Up |
| NONMMUG081638.1 | NONMMUT129942.1 | | 1026 | | 0 | | 7 | 7.708812649 | | Up |
| NONMMUG014170.2 | NONMMUT022916.2 | | 3245 | | 0 | | 7 | 7.708812649 | | Up |
| NONMMUG026756.2 | NONMMUT043333.2 | | 717 | | 0 | | 7 | 7.708812649 | | Up |
| NONMMUG026473.2 | NONMMUT042769.2 | | 1370 | | 0 | | 7 | 7.708812649 | | Up |
| NONMMUG073133.1 | NONMMUT116569.1 | | 1355 | | 0 | | 7 | 7.708812649 | | Up |
| NONMMUG008825.2 | NONMMUT013965.2 | | 2300 | | 0 | | 7 | 7.708812649 | | Up |
| NONMMUG079027.1 | NONMMUT125961.1 | | 946 | | 0 | | 7 | 7.708812649 | | Up |
| NONMMUG003173.2 | NONMMUT004940.2 | | 6368 | | 0 | | 7 | 7.708812649 | | Up |
| NONMMUG032962.2 | NONMMUT053076.2 | | 1460 | | 0 | | 7 | 7.708812649 | | Up |
| NONMMUG058280.1 | NONMMUT093455.1 | | 867 | | 0 | | 7 | 7.708812649 | | Up |
| NONMMUG024478.2 | NONMMUT039579.2 | | 1163 | | 0 | | 7 | 7.708812649 | | Up |
| NONMMUG084218.1 | NONMMUT133690.1 | | 680 | | 0 | | 7 | 7.708812649 | | Up |
| NONMMUG035157.2 | NONMMUT056622.2 | | 1906 | | 0 | | 7 | 7.708812649 | | Up |
| NONMMUG084892.1 | NONMMUT134698.1 | | 551 | | 1 | | 14 | 7.708812649 | | Up |
| NONMMUG008022.2 | NONMMUT012635.2 | | 2711 | | 0 | | 4.89 | 7.70291808 | | Up |
| NONMMUG055404.1 | NONMMUT088910.1 | | 2827.13 | | 0 | | 6.98 | 7.700556877 | | Up |
| NONMMUG065536.1 | NONMMUT104759.1 | | 2066 | | 0 | | 6.79 | 7.620925953 | | Up |
| LXLOC_037167 | LTCONS_00064647 | | 8853 | | 0 | | 6.69 | 7.578115226 | | Up |
| LXLOC_072059 | LTCONS_00126341 | | 860 | | 0 | | 6.54 | 7.512684076 | | Up |
| NONMMUG024320.2 | NONMMUT039355.2 | | 834 | | 0 | | 6.52 | 7.503846733 | | Up |
| NONMMUG026045.2 | NONMMUT042079.2 | | 3761 | | 1 | | 13 | 7.494982241 | | Up |
| NONMMUG000376.2 | NONMMUT000541.2 | | 4340 | | 0 | | 6.42 | 7.459249399 | | Up |
| NONMMUG008386.2 | NONMMUT013219.2 | | 1423 | | 0 | | 6.38 | 7.441215652 | | Up |
| LXLOC_041066 | LTCONS_00071977 | | 3277 | | 0 | | 6.35 | 7.427615988 | | Up |
| NONMMUG008989.2 | NONMMUT014195.2 | | 1945 | | 0 | | 6.32 | 7.413951921 | | Up |
| NONMMUG009486.2 | NONMMUT014958.2 | | 2633 | | 0 | | 6.15 | 7.335275625 | | Up |
| NONMMUG002015.2 | NONMMUT003122.2 | | 1557 | | 0 | | 6.04 | 7.283199904 | | Up |
| NONMMUG053929.1 | NONMMUT086593.1 | | 645 | | 0 | | 6 | 7.264027806 | | Up |
| NONMMUG067534.1 | NONMMUT108016.1 | | 1311 | | 0 | | 6 | 7.264027806 | | Up |
| NONMMUG085568.1 | NONMMUT135707.1 | | 1149 | | 0 | | 6 | 7.264027806 | | Up |
| NONMMUG043587.2 | NONMMUT070419.2 | | 825 | | 0 | | 6 | 7.264027806 | | Up |
| NONMMUG039941.2 | NONMMUT064689.2 | | 514 | | 0 | | 6 | 7.264027806 | | Up |
| NONMMUG063324.1 | NONMMUT101213.1 | | 654 | | 0 | | 6 | 7.264027806 | | Up |
| NONMMUG030386.2 | NONMMUT048926.2 | | 289 | | 0 | | 6 | 7.264027806 | | Up |
| NONMMUG035986.2 | NONMMUT057946.2 | | 1758 | | 0 | | 6 | 7.264027806 | | Up |
| NONMMUG053913.1 | NONMMUT086576.1 | | 2101 | | 0 | | 6 | 7.264027806 | | Up |
| NONMMUG068885.1 | NONMMUT110151.1 | | 1218 | | 0 | | 6 | 7.264027806 | | Up |
| NONMMUG074687.1 | NONMMUT119019.1 | | 3249 | | 0 | | 6 | 7.264027806 | | Up |
| NONMMUG000769.2 | NONMMUT001150.2 | | 1774 | | 0 | | 6 | 7.264027806 | | Up |
| NONMMUG027396.2 | NONMMUT044396.2 | | 551 | | 0 | | 6 | 7.264027806 | | Up |
| NONMMUG075875.1 | NONMMUT120974.1 | | 216 | | 0 | | 6 | 7.264027806 | | Up |
| NONMMUG005601.2 | NONMMUT009032.2 | | 1534 | | 0 | | 6 | 7.264027806 | | Up |
| NONMMUG035312.2 | NONMMUT056885.2 | | 1746 | | 0 | | 6 | 7.264027806 | | Up |
| NONMMUG008803.2 | NONMMUT013925.2 | | 830 | | 0 | | 6 | 7.264027806 | | Up |
| NONMMUG047184.1 | NONMMUT076069.1 | | 630 | | 0 | | 6 | 7.264027806 | | Up |
| NONMMUG081540.1 | NONMMUT129778.1 | | 6897 | | 0 | | 6 | 7.264027806 | | Up |
| NONMMUG022555.2 | NONMMUT036646.2 | | 674 | | 0 | | 6 | 7.264027806 | | Up |
| NONMMUG066224.1 | NONMMUT105941.1 | | 398 | | 0 | | 6 | 7.264027806 | | Up |
| NONMMUG032134.2 | NONMMUT051814.2 | | 1192.33 | | 0 | | 6 | 7.264027806 | | Up |
| NONMMUG042775.2 | NONMMUT069092.2 | | 1100 | | 1 | | 12 | 7.264027806 | | Up |
| LXLOC_078958 | LTCONS_00138362 | | 2422 | | 0 | | 5.99 | 7.25921481 | | Up |
| NONMMUG001096.2 | NONMMUT001635.2 | | 3204 | | 0 | | 5.96 | 7.244727466 | | Up |
| NONMMUG037383.2 | NONMMUT060165.2 | | 1235 | | 1.16 | | 13.5 | 7.175628198 | | Up |
| LXLOC_086385 | LTCONS_00150628 | | 3536 | | 0 | | 5.78 | 7.15624179 | | Up |
| LXLOC_092325 | LTCONS_00160255 | | 12363 | | 0 | | 5.67 | 7.100800275 | | Up |
| NONMMUG051319.1 | NONMMUT082440.1 | | 1572 | | 0 | | 5.64 | 7.08549313 | | Up |
| NONMMUG025784.2 | NONMMUT041689.2 | | 237 | | 0 | | 5.64 | 7.08549313 | | Up |
| NONMMUG002636.2 | NONMMUT004069.2 | | 2743 | | 0 | | 5.55 | 7.039078347 | | Up |
| NONMMUG024403.2 | NONMMUT039476.2 | | 1416 | | 0 | | 5.53 | 7.028661765 | | Up |
| NONMMUG020092.2 | NONMMUT032621.2 | | 2033 | | 1 | | 11 | 7.012966042 | | Up |
| NONMMUG075209.1 | NONMMUT119929.1 | | 2236 | | 1 | | 11 | 7.012966042 | | Up |
| NONMMUG012411.2 | NONMMUT020012.2 | | 846.45 | | 1 | | 11 | 7.012966042 | | Up |
| NONMMUG041768.2 | NONMMUT067440.2 | | 403 | | 1 | | 11 | 7.012966042 | | Up |
| NONMMUG017913.2 | NONMMUT028986.2 | | 2956 | | 1 | | 11 | 7.012966042 | | Up |
| NONMMUG052429.1 | NONMMUT084185.1 | | 341 | | 1 | | 11 | 7.012966042 | | Up |
| NONMMUG067686.1 | NONMMUT108225.1 | | 1473 | | 1 | | 11 | 7.012966042 | | Up |
| NONMMUG068551.1 | NONMMUT109614.1 | | 3288 | | 0 | | 5.46 | 6.991904707 | | Up |
| LXLOC_010567 | LTCONS_00018255 | | 867 | | 0 | | 5.45 | 6.986615264 | | Up |
| NONMMUG037173.2 | NONMMUT059856.2 | | 2647 | | 0 | | 5.41 | 6.965359993 | | Up |
| NONMMUG023747.2 | NONMMUT038425.2 | | 3994 | | 0 | | 5.35 | 6.933180587 | | Up |
| NONMMUG048512.1 | NONMMUT078155.1 | | 642 | | 0 | | 5.29 | 6.900638249 | | Up |
| LXLOC_067477 | LTCONS_00118324 | | 860 | | 0 | | 5.25 | 6.87873765 | | Up |
| NONMMUG023373.2 | NONMMUT037855.2 | | 3196 | | 2 | | 21 | 6.87873765 | | Up |
| NONMMUG016557.2 | NONMMUT026710.2 | | 2517 | | 0 | | 4.68 | 6.851126051 | | Up |
| NONMMUG071131.1 | NONMMUT113481.1 | | 1003 | | 0 | | 5.19 | 6.845571882 | | Up |
| NONMMUG070016.1 | NONMMUT111834.1 | | 500 | | 0 | | 5.18 | 6.840007 | | Up |
| NONMMUG046244.2 | NONMMUT074553.2 | | 1359 | | 0 | | 5.18 | 6.840007 | | Up |
| NONMMUG002855.2 | NONMMUT004419.2 | | 1244 | | 0 | | 5.14 | 6.817639523 | | Up |
| LXLOC_013756 | LTCONS_00023788 | | 7183 | | 0 | | 5.12 | 6.806390425 | | Up |
| LXLOC_054160 | LTCONS_00095096 | | 1977 | | 4.54 | | 45.84 | 6.765788487 | | Up |
| NONMMUG013951.2 | NONMMUT022606.2 | | 1050 | | 0 | | 5.04 | 6.760950272 | | Up |
| NONMMUG044043.2 | NONMMUT133556.1 | | 13991 | | 1 | | 10.04 | 6.749477533 | | Up |
| NONMMUG010779.2 | NONMMUT017156.2 | | 1947 | | 0 | | 5.01 | 6.743724011 | | Up |
| NONMMUG003692.2 | NONMMUT005735.2 | | 934 | | 0 | | 5 | 6.737958994 | | Up |
| NONMMUG085793.1 | NONMMUT135999.1 | | 3054 | | 0 | | 5 | 6.737958994 | | Up |
| NONMMUG023888.2 | NONMMUT038696.2 | | 1192 | | 0 | | 5 | 6.737958994 | | Up |
| NONMMUG046972.1 | NONMMUT075719.1 | | 2147 | | 0 | | 5 | 6.737958994 | | Up |
| NONMMUG016813.2 | NONMMUT027107.2 | | 2289 | | 0 | | 5 | 6.737958994 | | Up |
| NONMMUG049306.1 | NONMMUT079252.1 | | 268 | | 0 | | 5 | 6.737958994 | | Up |
| NONMMUG026112.2 | NONMMUT042169.2 | | 2178 | | 0 | | 5 | 6.737958994 | | Up |
| NONMMUG001806.2 | NONMMUT002673.2 | | 477 | | 0 | | 5 | 6.737958994 | | Up |
| NONMMUG058532.1 | NONMMUT093819.1 | | 1005 | | 0 | | 5 | 6.737958994 | | Up |
| NONMMUG058063.1 | NONMMUT093132.1 | | 1514 | | 0 | | 5 | 6.737958994 | | Up |
| NONMMUG070494.1 | NONMMUT112597.1 | | 1404 | | 0 | | 5 | 6.737958994 | | Up |
| NONMMUG025526.2 | NONMMUT041217.2 | | 1427 | | 0 | | 5 | 6.737958994 | | Up |
| NONMMUG043489.2 | NONMMUT070274.2 | | 959 | | 0 | | 5 | 6.737958994 | | Up |
| NONMMUG054398.1 | NONMMUT087336.1 | | 1663 | | 0 | | 5 | 6.737958994 | | Up |
| NONMMUG083307.1 | NONMMUT132321.1 | | 480 | | 0 | | 5 | 6.737958994 | | Up |
| NONMMUG061934.1 | NONMMUT099001.1 | | 1651 | | 0 | | 5 | 6.737958994 | | Up |
| NONMMUG034783.2 | NONMMUT055998.2 | | 2358 | | 0 | | 5 | 6.737958994 | | Up |
| NONMMUG013202.2 | NONMMUT021276.2 | | 3002 | | 0 | | 5 | 6.737958994 | | Up |
| NONMMUG018149.2 | NONMMUT029347.2 | | 689 | | 0 | | 5 | 6.737958994 | | Up |
| NONMMUG016179.2 | NONMMUT026137.2 | | 1209 | | 0 | | 5 | 6.737958994 | | Up |
| NONMMUG024600.2 | NONMMUT039756.2 | | 3555 | | 0 | | 5 | 6.737958994 | | Up |
| NONMMUG016990.2 | NONMMUT027412.2 | | 1074 | | 0 | | 5 | 6.737958994 | | Up |
| NONMMUG005688.2 | NONMMUT009173.2 | | 781 | | 0 | | 5 | 6.737958994 | | Up |
| NONMMUG069415.1 | NONMMUT110976.1 | | 1181 | | 0 | | 5 | 6.737958994 | | Up |
| NONMMUG021450.2 | NONMMUT034851.2 | | 1582 | | 0 | | 5 | 6.737958994 | | Up |
| NONMMUG012875.2 | NONMMUT020694.2 | | 1926.52 | | 0 | | 5 | 6.737958994 | | Up |
| NONMMUG035021.2 | NONMMUT056391.2 | | 5036 | | 0 | | 5 | 6.737958994 | | Up |
| NONMMUG072322.1 | NONMMUT115314.1 | | 816 | | 0 | | 5 | 6.737958994 | | Up |
| NONMMUG083438.1 | NONMMUT132532.1 | | 221 | | 0 | | 5 | 6.737958994 | | Up |
| NONMMUG048805.1 | NONMMUT078557.1 | | 3389 | | 0 | | 5 | 6.737958994 | | Up |
| NONMMUG017343.2 | NONMMUT027995.2 | | 969 | | 0 | | 5 | 6.737958994 | | Up |
| NONMMUG033221.2 | NONMMUT053525.2 | | 1729 | | 0 | | 5 | 6.737958994 | | Up |
| NONMMUG080949.1 | NONMMUT128938.1 | | 1006 | | 0 | | 5 | 6.737958994 | | Up |
| NONMMUG067474.1 | NONMMUT107915.1 | | 997 | | 0 | | 5 | 6.737958994 | | Up |
| NONMMUG084878.1 | NONMMUT134667.1 | | 1955 | | 0 | | 5 | 6.737958994 | | Up |
| NONMMUG083579.1 | NONMMUT132781.1 | | 721 | | 0 | | 5 | 6.737958994 | | Up |
| NONMMUG016213.2 | NONMMUT026193.2 | | 496 | | 0 | | 5 | 6.737958994 | | Up |
| NONMMUG042366.2 | NONMMUT068459.2 | | 4147 | | 0 | | 5 | 6.737958994 | | Up |
| NONMMUG036457.2 | NONMMUT058653.2 | | 1678 | | 0 | | 5 | 6.737958994 | | Up |
| NONMMUG052685.1 | NONMMUT084593.1 | | 742 | | 0 | | 5 | 6.737958994 | | Up |
| NONMMUG012619.2 | NONMMUT020323.2 | | 1795 | | 0 | | 5 | 6.737958994 | | Up |
| NONMMUG064474.1 | NONMMUT103115.1 | | 766 | | 0 | | 5 | 6.737958994 | | Up |
| NONMMUG011614.2 | NONMMUT018665.2 | | 1519 | | 0 | | 5 | 6.737958994 | | Up |
| NONMMUG080346.1 | NONMMUT128027.1 | | 829 | | 0 | | 5 | 6.737958994 | | Up |
| NONMMUG018764.2 | NONMMUT030354.2 | | 1067 | | 0 | | 5 | 6.737958994 | | Up |
| NONMMUG002983.2 | NONMMUT004632.2 | | 794.21 | | 0 | | 5 | 6.737958994 | | Up |
| NONMMUG081354.1 | NONMMUT129501.1 | | 808 | | 0 | | 5 | 6.737958994 | | Up |
| NONMMUG045466.2 | NONMMUT073314.2 | | 1395 | | 0 | | 5 | 6.737958994 | | Up |
| NONMMUG034248.2 | NONMMUT055164.2 | | 1851 | | 0 | | 5 | 6.737958994 | | Up |
| NONMMUG011240.2 | NONMMUT017962.2 | | 733.47 | | 0 | | 5 | 6.737958994 | | Up |
| NONMMUG018283.2 | NONMMUT029575.2 | | 3516 | | 0 | | 5 | 6.737958994 | | Up |
| NONMMUG039521.2 | NONMMUT064012.2 | | 3050.16 | | 0 | | 5 | 6.737958994 | | Up |
| NONMMUG001916.2 | NONMMUT002957.2 | | 3660 | | 0 | | 5 | 6.737958994 | | Up |
| NONMMUG080216.1 | NONMMUT127822.1 | | 1103 | | 0 | | 5 | 6.737958994 | | Up |
| NONMMUG034829.2 | NONMMUT056060.2 | | 2434 | | 0 | | 5 | 6.737958994 | | Up |
| NONMMUG053867.1 | NONMMUT086497.1 | | 2063 | | 0 | | 5 | 6.737958994 | | Up |
| NONMMUG021916.2 | NONMMUT035539.2 | | 514 | | 1 | | 10 | 6.737958994 | | Up |
| NONMMUG004305.2 | NONMMUT006694.2 | | 421 | | 1 | | 10 | 6.737958994 | | Up |
| NONMMUG016218.2 | NONMMUT026201.2 | | 4060 | | 1 | | 10 | 6.737958994 | | Up |
| NONMMUG019626.2 | NONMMUT031914.2 | | 903.43 | | 1 | | 10 | 6.737958994 | | Up |
| NONMMUG085798.1 | NONMMUT136004.1 | | 5433 | | 1 | | 10 | 6.737958994 | | Up |
| NONMMUG067655.1 | NONMMUT108189.1 | | 3979 | | 1 | | 10 | 6.737958994 | | Up |
| NONMMUG028781.2 | NONMMUT046572.2 | | 2190.56 | | 6 | | 58 | 6.640139793 | | Up |
| NONMMUG009976.2 | NONMMUT015877.2 | | 1726.43 | | 2 | | 19 | 6.589957831 | | Up |
| NONMMUG038588.2 | NONMMUT062558.2 | | 1557 | | 2 | | 19 | 6.589957831 | | Up |
| NONMMUG012868.2 | NONMMUT020680.2 | | 1806 | | 9 | | 83 | 6.504331664 | | Up |
| NONMMUG019557.2 | NONMMUT031807.2 | | 1569 | | 0 | | 4.82 | 6.464040568 | | Up |
| NONMMUG023935.2 | NONMMUT038759.2 | | 573 | | 3.17 | | 28.76 | 6.457120665 | | Up |
| NONMMUG026182.2 | NONMMUT042277.2 | | 476 | | 1 | | 9 | 6.433952807 | | Up |
| NONMMUG067476.1 | NONMMUT107917.1 | | 816 | | 1 | | 9 | 6.433952807 | | Up |
| NONMMUG052617.1 | NONMMUT084488.1 | | 287 | | 1 | | 9 | 6.433952807 | | Up |
| NONMMUG072326.1 | NONMMUT115325.1 | | 1213 | | 1 | | 9 | 6.433952807 | | Up |
| NONMMUG067895.1 | NONMMUT108619.1 | | 1408 | | 1 | | 9 | 6.433952807 | | Up |
| NONMMUG051144.1 | NONMMUT082162.1 | | 514 | | 1 | | 9 | 6.433952807 | | Up |
| NONMMUG085808.1 | NONMMUT136014.1 | | 1259 | | 1 | | 9 | 6.433952807 | | Up |
| NONMMUG039191.2 | NONMMUT063455.2 | | 851 | | 1 | | 9 | 6.433952807 | | Up |
| NONMMUG020945.2 | NONMMUT034078.2 | | 4729 | | 1 | | 9 | 6.433952807 | | Up |
| NONMMUG005661.2 | NONMMUT009122.2 | | 675 | | 1 | | 9 | 6.433952807 | | Up |
| NONMMUG035306.2 | NONMMUT056878.2 | | 482 | | 1 | | 9 | 6.433952807 | | Up |
| NONMMUG032217.2 | NONMMUT051948.2 | | 3744 | | 1 | | 9 | 6.433952807 | | Up |
| NONMMUG038263.2 | NONMMUT061939.2 | | 3581 | | 1 | | 9 | 6.433952807 | | Up |
| NONMMUG073163.1 | NONMMUT116617.1 | | 5514 | | 1 | | 8.76 | 6.355964544 | | Up |
| NONMMUG009975.2 | NONMMUT015846.2 | | 1632.02 | | 3 | | 26 | 6.325057239 | | Up |
| NONMMUG011246.2 | NONMMUT017972.2 | | 3858 | | 2 | | 17 | 6.269028487 | | Up |
| NONMMUG000833.2 | NONMMUT001234.2 | | 549 | | 0 | | 6.57 | 6.206040429 | | Up |
| NONMMUG032065.2 | NONMMUT051698.2 | | 2268 | | 4 | | 33.01 | 6.183765271 | | Up |
| NONMMUG041735.2 | NONMMUT067395.2 | | 3559 | | 4 | | 33 | 6.182891043 | | Up |
| NONMMUG022341.2 | NONMMUT036302.2 | | 3132 | | 5 | | 40.87 | 6.156187291 | | Up |
| NONMMUG076108.1 | NONMMUT121358.1 | | 1344 | | 1.08 | | 8.65 | 6.097440445 | | Up |
| LXLOC_071802 | LTCONS_00125918 | | 15432 | | 1 | | 8 | 6.094102804 | | Up |
| NONMMUG039303.2 | NONMMUT063634.2 | | 550 | | 1 | | 8 | 6.094102804 | | Up |
| NONMMUG033082.2 | NONMMUT053274.2 | | 412 | | 1 | | 8 | 6.094102804 | | Up |
| NONMMUG027654.2 | NONMMUT044785.2 | | 414 | | 1 | | 8 | 6.094102804 | | Up |
| NONMMUG058683.1 | NONMMUT094010.1 | | 1899 | | 1 | | 8 | 6.094102804 | | Up |
| NONMMUG007867.2 | NONMMUT012414.2 | | 490 | | 1 | | 8 | 6.094102804 | | Up |
| NONMMUG025665.2 | NONMMUT041453.2 | | 1760 | | 1 | | 8 | 6.094102804 | | Up |
| NONMMUG048053.1 | NONMMUT077436.1 | | 2036 | | 1 | | 8 | 6.094102804 | | Up |
| NONMMUG001753.2 | NONMMUT002596.2 | | 4150 | | 1 | | 8 | 6.094102804 | | Up |
| NONMMUG048359.1 | NONMMUT077920.1 | | 775 | | 1 | | 8 | 6.094102804 | | Up |
| NONMMUG038288.2 | NONMMUT061971.2 | | 2668 | | 1 | | 8 | 6.094102804 | | Up |
| NONMMUG057887.1 | NONMMUT092871.1 | | 2344.06 | | 1 | | 8 | 6.094102804 | | Up |
| NONMMUG064864.1 | NONMMUT103752.1 | | 1529 | | 1 | | 8 | 6.094102804 | | Up |
| NONMMUG001000.2 | NONMMUT001490.2 | | 2534 | | 1 | | 8 | 6.094102804 | | Up |
| NONMMUG048243.1 | NONMMUT077742.1 | | 1569 | | 1 | | 8 | 6.094102804 | | Up |
| NONMMUG062850.1 | NONMMUT100463.1 | | 897 | | 2 | | 16 | 6.094102804 | | Up |
| NONMMUG039885.2 | NONMMUT064606.2 | | 2352.01 | | 5 | | 40 | 6.094102804 | | Up |
| NONMMUG039196.2 | NONMMUT063462.2 | | 1587 | | 0 | | 6.18 | 6.066224423 | | Up |
| NONMMUG058899.1 | NONMMUT094351.1 | | 1913 | | 5 | | 39 | 6.021051052 | | Up |
| NONMMUG042938.2 | NONMMUT069328.2 | | 1082 | | 1.01 | | 7.68 | 5.94760484 | | Up |
| NONMMUG046111.2 | NONMMUT074345.2 | | 1460 | | 1.14 | | 8.63 | 5.934756274 | | Up |
| NONMMUG067758.1 | NONMMUT108371.1 | | 2312 | | 1 | | 7.51 | 5.91172862 | | Up |
| NONMMUG009383.2 | NONMMUT014795.2 | | 1857 | | 2 | | 15 | 5.907883996 | | Up |
| NONMMUG078752.1 | NONMMUT125638.1 | | 2099 | | 0 | | 6.6 | 5.779623321 | | Up |
| NONMMUG051611.1 | NONMMUT082934.1 | | 690 | | 1.04 | | 7.36 | 5.74034728 | | Up |
| NONMMUG074494.1 | NONMMUT118707.1 | | 1142 | | 1 | | 7.07 | 5.737523235 | | Up |
| NONMMUG079062.1 | NONMMUT126018.1 | | 2562 | | 1 | | 7 | 5.708812649 | | Up |
| NONMMUG075911.1 | NONMMUT121037.1 | | 823.37 | | 1 | | 7 | 5.708812649 | | Up |
| NONMMUG029415.2 | NONMMUT047505.2 | | 1334 | | 1 | | 7 | 5.708812649 | | Up |
| NONMMUG072768.1 | NONMMUT116068.1 | | 420 | | 1 | | 7 | 5.708812649 | | Up |
| NONMMUG068142.1 | NONMMUT108973.1 | | 2112 | | 1 | | 7 | 5.708812649 | | Up |
| NONMMUG013097.2 | NONMMUT021098.2 | | 497 | | 1 | | 7 | 5.708812649 | | Up |
| NONMMUG056903.1 | NONMMUT091322.1 | | 425 | | 1 | | 7 | 5.708812649 | | Up |
| NONMMUG004798.2 | NONMMUT007568.2 | | 2348 | | 1 | | 7 | 5.708812649 | | Up |
| NONMMUG017831.2 | NONMMUT028826.2 | | 605 | | 1 | | 7 | 5.708812649 | | Up |
| NONMMUG038889.2 | NONMMUT063014.2 | | 2390 | | 1 | | 7 | 5.708812649 | | Up |
| NONMMUG026780.2 | NONMMUT043379.2 | | 3681 | | 1 | | 7 | 5.708812649 | | Up |
| NONMMUG040753.2 | NONMMUT065862.2 | | 3402 | | 1 | | 7 | 5.708812649 | | Up |
| NONMMUG022238.2 | NONMMUT036127.2 | | 2710.3 | | 1 | | 7 | 5.708812649 | | Up |
| NONMMUG000955.2 | NONMMUT001409.2 | | 275 | | 1 | | 7 | 5.708812649 | | Up |
| NONMMUG055258.1 | NONMMUT088694.1 | | 1187 | | 1 | | 7 | 5.708812649 | | Up |
| NONMMUG043239.2 | NONMMUT069856.2 | | 1771 | | 1 | | 7 | 5.708812649 | | Up |
| NONMMUG071598.1 | NONMMUT114158.1 | | 722.46 | | 1 | | 7 | 5.708812649 | | Up |
| NONMMUG066730.1 | NONMMUT106772.1 | | 353 | | 1 | | 7 | 5.708812649 | | Up |
| NONMMUG017996.2 | NONMMUT029109.2 | | 1660 | | 1 | | 7 | 5.708812649 | | Up |
| NONMMUG055171.1 | NONMMUT088548.1 | | 1645 | | 1 | | 7 | 5.708812649 | | Up |
| NONMMUG038432.2 | NONMMUT062311.2 | | 1633 | | 1 | | 7 | 5.708812649 | | Up |
| NONMMUG032970.2 | NONMMUT053085.2 | | 513 | | 1 | | 7 | 5.708812649 | | Up |
| NONMMUG017194.2 | NONMMUT027743.2 | | 796 | | 1 | | 7 | 5.708812649 | | Up |
| NONMMUG085777.1 | NONMMUT135983.1 | | 2354 | | 1 | | 7 | 5.708812649 | | Up |
| NONMMUG040432.2 | NONMMUT065390.2 | | 778 | | 1 | | 7 | 5.708812649 | | Up |
| NONMMUG065867.1 | NONMMUT105335.1 | | 221 | | 1 | | 7 | 5.708812649 | | Up |
| NONMMUG018821.2 | NONMMUT030427.2 | | 2348 | | 1 | | 7 | 5.708812649 | | Up |
| NONMMUG010747.2 | NONMMUT017084.2 | | 602 | | 2 | | 14 | 5.708812649 | | Up |
| NONMMUG036388.2 | NONMMUT058540.2 | | 1122.8 | | 2 | | 14 | 5.708812649 | | Up |
| NONMMUG064713.1 | NONMMUT103513.1 | | 1242 | | 2 | | 14 | 5.708812649 | | Up |
| NONMMUG028031.2 | NONMMUT045424.2 | | 2609 | | 11 | | 77 | 5.708812649 | | Up |
| LXLOC_022862 | LTCONS_00039631 | | 2665 | | 2.15 | | 15 | 5.699210676 | | Up |
| NONMMUG018388.2 | NONMMUT029766.2 | | 4393 | | 0 | | 6.49 | 5.669074437 | | Up |
| NONMMUG081494.1 | NONMMUT129706.1 | | 2741 | | 3 | | 20.44 | 5.630824385 | | Up |
| NONMMUG086505.1 | NONMMUT136930.1 | | 511 | | 1 | | 6.63 | 5.552120545 | | Up |
| NONMMUG001894.2 | NONMMUT002905.2 | | 3451 | | 1.06 | | 7 | 5.540684119 | | Up |
| NONMMUG009595.2 | NONMMUT015113.2 | | 1048.03 | | 5.08 | | 33.32 | 5.521078802 | | Up |
| NONMMUG039893.2 | NONMMUT064618.2 | | 3815 | | 2 | | 13 | 5.494982241 | | Up |
| NONMMUG018707.2 | NONMMUT030264.2 | | 1158.19 | | 2 | | 13 | 5.494982241 | | Up |
| NONMMUG048123.1 | NONMMUT077540.1 | | 1262 | | 2 | | 13 | 5.494982241 | | Up |
| NONMMUG032832.2 | NONMMUT052871.2 | | 2639 | | 2 | | 13 | 5.494982241 | | Up |
| NONMMUG013268.2 | NONMMUT021373.2 | | 650 | | 2 | | 13 | 5.494982241 | | Up |
| NONMMUG057182.1 | NONMMUT091792.1 | | 3282 | | 1.01 | | 6.53 | 5.479558202 | | Up |
| NONMMUG042516.2 | NONMMUT068670.2 | | 665 | | 3.17 | | 20.36 | 5.460468436 | | Up |
| NONMMUG053408.1 | NONMMUT085783.1 | | 1046 | | 5 | | 32 | 5.450246615 | | Up |
| NONMMUG079230.1 | NONMMUT126302.1 | | 2758 | | 1.13 | | 7.22 | 5.445454933 | | Up |
| NONMMUG009180.2 | NONMMUT014497.2 | | 3724 | | 2 | | 12.72 | 5.432156335 | | Up |
| NONMMUG033344.2 | NONMMUT053753.2 | | 796 | | 1.01 | | 6.42 | 5.430538813 | | Up |
| NONMMUG085362.1 | NONMMUT135380.1 | | 1154 | | 1.05 | | 6.62 | 5.406986583 | | Up |
| NONMMUG011236.2 | NONMMUT017958.2 | | 957 | | 1.02 | | 6.43 | 5.406601975 | | Up |
| NONMMUG040586.2 | NONMMUT065615.2 | | 2046 | | 1 | | 6.27 | 5.391033691 | | Up |
| NONMMUG027141.2 | NONMMUT113601.1 | | 1002.39 | | 976 | | 1529 | 5.389373512 | | Up |
| NONMMUG054040.1 | NONMMUT086764.1 | | 655 | | 2 | | 12.45 | 5.370250479 | | Up |
| NONMMUG014289.2 | NONMMUT023094.2 | | 2212.23 | | 14 | | 87 | 5.365279952 | | Up |
| NONMMUG058168.1 | NONMMUT093290.1 | | 729 | | 1 | | 6.14 | 5.330580116 | | Up |
| NONMMUG037684.2 | NONMMUT060628.2 | | 2521 | | 1 | | 6.08 | 5.302245452 | | Up |
| NONMMUG061792.1 | NONMMUT098832.1 | | 2378 | | 1.15 | | 6.99 | 5.301419993 | | Up |
| NONMMUG009989.2 | NONMMUT015904.2 | | 3900 | | 1 | | 6.07 | 5.297495837 | | Up |
| NONMMUG060124.1 | NONMMUT096178.1 | | 2065 | | 1 | | 6.02 | 5.273629778 | | Up |
| NONMMUG051850.1 | NONMMUT083287.1 | | 1956 | | 1 | | 6.01 | 5.268832786 | | Up |
| NONMMUG048301.1 | NONMMUT077820.1 | | 1399 | | 1 | | 6 | 5.264027806 | | Up |
| NONMMUG038822.2 | NONMMUT062917.2 | | 7269 | | 1 | | 6 | 5.264027806 | | Up |
| NONMMUG036025.2 | NONMMUT124114.1 | | 352 | | 1 | | 6 | 5.264027806 | | Up |
| NONMMUG073657.1 | NONMMUT117435.1 | | 921 | | 1 | | 6 | 5.264027806 | | Up |
| NONMMUG036377.2 | NONMMUT058522.2 | | 375 | | 1 | | 6 | 5.264027806 | | Up |
| NONMMUG044974.2 | NONMMUT072574.2 | | 392 | | 1 | | 6 | 5.264027806 | | Up |
| NONMMUG081239.1 | NONMMUT129375.1 | | 1220 | | 1 | | 6 | 5.264027806 | | Up |
| NONMMUG018813.2 | NONMMUT030417.2 | | 389 | | 1 | | 6 | 5.264027806 | | Up |
| NONMMUG053927.1 | NONMMUT086591.1 | | 1106 | | 1 | | 6 | 5.264027806 | | Up |
| NONMMUG010090.2 | NONMMUT016051.2 | | 755.51 | | 1 | | 6 | 5.264027806 | | Up |
| NONMMUG011300.2 | NONMMUT018097.2 | | 905 | | 1 | | 6 | 5.264027806 | | Up |
| NONMMUG051437.1 | NONMMUT082644.1 | | 702 | | 1 | | 6 | 5.264027806 | | Up |
| NONMMUG032184.2 | NONMMUT051896.2 | | 489 | | 1 | | 6 | 5.264027806 | | Up |
| NONMMUG003725.2 | NONMMUT005773.2 | | 820 | | 1 | | 6 | 5.264027806 | | Up |
| NONMMUG071278.1 | NONMMUT113708.1 | | 1344 | | 1 | | 6 | 5.264027806 | | Up |
| NONMMUG081350.1 | NONMMUT129497.1 | | 1380 | | 1 | | 6 | 5.264027806 | | Up |
| NONMMUG021559.2 | NONMMUT035000.2 | | 763 | | 1 | | 6 | 5.264027806 | | Up |
| NONMMUG063405.1 | NONMMUT101337.1 | | 1569 | | 1 | | 6 | 5.264027806 | | Up |
| NONMMUG075796.1 | NONMMUT120844.1 | | 2076 | | 1 | | 6 | 5.264027806 | | Up |
| NONMMUG066374.1 | NONMMUT106197.1 | | 543 | | 1 | | 6 | 5.264027806 | | Up |
| NONMMUG057007.1 | NONMMUT091509.1 | | 938 | | 1 | | 6 | 5.264027806 | | Up |
| NONMMUG050581.1 | NONMMUT081344.1 | | 963 | | 1 | | 6 | 5.264027806 | | Up |
| NONMMUG057637.1 | NONMMUT092479.1 | | 621 | | 1 | | 6 | 5.264027806 | | Up |
| NONMMUG052705.1 | NONMMUT084624.1 | | 668 | | 1 | | 6 | 5.264027806 | | Up |
| NONMMUG055657.1 | NONMMUT089345.1 | | 822 | | 1 | | 6 | 5.264027806 | | Up |
| NONMMUG013296.2 | NONMMUT021417.2 | | 572 | | 1 | | 6 | 5.264027806 | | Up |
| NONMMUG080164.1 | NONMMUT127705.1 | | 652 | | 1 | | 6 | 5.264027806 | | Up |
| NONMMUG043800.2 | NONMMUT070748.2 | | 1423.83 | | 1 | | 6 | 5.264027806 | | Up |
| NONMMUG002540.2 | NONMMUT003933.2 | | 455 | | 1 | | 6 | 5.264027806 | | Up |
| NONMMUG061927.1 | NONMMUT098994.1 | | 1047 | | 1 | | 6 | 5.264027806 | | Up |
| NONMMUG000336.2 | NONMMUT000482.2 | | 2311 | | 1 | | 6 | 5.264027806 | | Up |
| NONMMUG079889.1 | NONMMUT127280.1 | | 1322 | | 1 | | 6 | 5.264027806 | | Up |
| NONMMUG082406.1 | NONMMUT130947.1 | | 5851 | | 1 | | 6 | 5.264027806 | | Up |
| NONMMUG039318.2 | NONMMUT063662.2 | | 1009.01 | | 1 | | 6 | 5.264027806 | | Up |
| NONMMUG040110.2 | NONMMUT064947.2 | | 2887 | | 1 | | 6 | 5.264027806 | | Up |
| NONMMUG085795.1 | NONMMUT136001.1 | | 1278 | | 1 | | 6 | 5.264027806 | | Up |
| NONMMUG015132.2 | NONMMUT024468.2 | | 953 | | 1 | | 6 | 5.264027806 | | Up |
| NONMMUG082989.1 | NONMMUT131872.1 | | 205 | | 1 | | 6 | 5.264027806 | | Up |
| NONMMUG063952.1 | NONMMUT102173.1 | | 2358 | | 1 | | 6 | 5.264027806 | | Up |
| NONMMUG036828.2 | NONMMUT059271.2 | | 844 | | 1 | | 6 | 5.264027806 | | Up |
| NONMMUG005565.2 | NONMMUT008969.2 | | 1148 | | 1 | | 6 | 5.264027806 | | Up |
| NONMMUG081195.1 | NONMMUT129321.1 | | 1136 | | 1 | | 6 | 5.264027806 | | Up |
| NONMMUG020645.2 | NONMMUT033544.2 | | 3206 | | 1 | | 6 | 5.264027806 | | Up |
| NONMMUG038565.2 | NONMMUT062518.2 | | 3712 | | 1 | | 6 | 5.264027806 | | Up |
| NONMMUG069657.1 | NONMMUT111313.1 | | 1666 | | 1 | | 6 | 5.264027806 | | Up |
| NONMMUG018916.2 | NONMMUT030684.2 | | 2230 | | 1 | | 6 | 5.264027806 | | Up |
| NONMMUG025019.2 | NONMMUT040382.2 | | 363 | | 1 | | 6 | 5.264027806 | | Up |
| NONMMUG063948.1 | NONMMUT102167.1 | | 245 | | 1 | | 6 | 5.264027806 | | Up |
| NONMMUG060770.1 | NONMMUT097220.1 | | 1666.83 | | 1 | | 6 | 5.264027806 | | Up |
| NONMMUG000652.2 | NONMMUT000967.2 | | 1902 | | 1 | | 6 | 5.264027806 | | Up |
| NONMMUG086398.1 | NONMMUT136776.1 | | 2139 | | 1 | | 6 | 5.264027806 | | Up |
| NONMMUG017698.2 | NONMMUT028601.2 | | 3725.24 | | 1 | | 6 | 5.264027806 | | Up |
| NONMMUG083257.1 | NONMMUT132250.1 | | 652 | | 1 | | 6 | 5.264027806 | | Up |
| NONMMUG026928.2 | NONMMUT043569.2 | | 2325 | | 1 | | 6 | 5.264027806 | | Up |
| NONMMUG010641.2 | NONMMUT016925.2 | | 681 | | 2 | | 12 | 5.264027806 | | Up |
| NONMMUG072482.1 | NONMMUT115563.1 | | 4301 | | 2 | | 12 | 5.264027806 | | Up |
| NONMMUG046942.1 | NONMMUT075675.1 | | 978 | | 2 | | 12 | 5.264027806 | | Up |
| NONMMUG081646.1 | NONMMUT129955.1 | | 1903 | | 2 | | 12 | 5.264027806 | | Up |
| LXLOC_027887 | LTCONS_00048376 | | 6679 | | 1.31 | | 7.84 | 5.25667649 | | Up |
| NONMMUG050588.1 | NONMMUT081351.1 | | 719 | | 1.09 | | 6.51 | 5.250761621 | | Up |
| NONMMUG057496.1 | NONMMUT092261.1 | | 1647 | | 1 | | 5.96 | 5.244727466 | | Up |
| NONMMUG082337.1 | NONMMUT130867.1 | | 1265 | | 1.17 | | 6.97 | 5.243403057 | | Up |
| LXLOC_037758 | LTCONS_00065675 | | 1040 | | 0 | | 5.71 | 5.238871674 | | Up |
| NONMMUG030954.2 | NONMMUT049995.2 | | 1465.1 | | 1.01 | | 6 | 5.23531722 | | Up |
| NONMMUG052275.1 | NONMMUT083952.1 | | 2198 | | 2 | | 11.82 | 5.220419065 | | Up |
| NONMMUG084993.1 | NONMMUT134846.1 | | 882 | | 2.05 | | 12.04 | 5.202381959 | | Up |
| NONMMUG026480.2 | NONMMUT042780.2 | | 6694.43 | | 55.01 | | 322.35 | 5.195827155 | | Up |
| NONMMUG042888.2 | NONMMUT069267.2 | | 865 | | 1 | | 5.85 | 5.190976054 | | Up |
| NONMMUG034787.2 | NONMMUT056004.2 | | 1369 | | 1 | | 5.79 | 5.161229501 | | Up |
| LXLOC_063397 | LTCONS_00110876 | | 1235 | | 3.02 | | 17.2 | 5.113679025 | | Up |
| NONMMUG059197.1 | NONMMUT094818.1 | | 2496 | | 3 | | 17 | 5.099103486 | | Up |
| NONMMUG084004.1 | NONMMUT133324.1 | | 8102 | | 3 | | 17 | 5.099103486 | | Up |
| NONMMUG060347.1 | NONMMUT096553.1 | | 1658 | | 1.46 | | 8.24 | 5.087454741 | | Up |
| NONMMUG031297.2 | NONMMUT050513.2 | | 1399 | | 1.08 | | 5.98 | 5.032331149 | | Up |
| NONMMUG077643.1 | NONMMUT123825.1 | | 243 | | 2 | | 11 | 5.012966042 | | Up |
| NONMMUG031332.2 | NONMMUT050562.2 | | 345 | | 2 | | 11 | 5.012966042 | | Up |
| NONMMUG032625.2 | NONMMUT052557.2 | | 1111 | | 2 | | 11 | 5.012966042 | | Up |
| NONMMUG080854.1 | NONMMUT128797.1 | | 1582 | | 2 | | 11 | 5.012966042 | | Up |
| NONMMUG003998.2 | NONMMUT006207.2 | | 1262 | | 2 | | 11 | 5.012966042 | | Up |
| NONMMUG074209.1 | NONMMUT118234.1 | | 1003 | | 2 | | 11 | 5.012966042 | | Up |
| NONMMUG062770.1 | NONMMUT100336.1 | | 1682 | | 2 | | 11 | 5.012966042 | | Up |
| NONMMUG015445.2 | NONMMUT024972.2 | | 1291 | | 2 | | 11 | 5.012966042 | | Up |
| NONMMUG018971.2 | NONMMUT030753.2 | | 2245 | | 2 | | 11 | 5.012966042 | | Up |
| NONMMUG013657.2 | NONMMUT021984.2 | | 965 | | 2 | | 11 | 5.012966042 | | Up |
| NONMMUG017548.2 | NONMMUT028351.2 | | 676 | | 2 | | 11 | 5.012966042 | | Up |
| NONMMUG066768.1 | NONMMUT106842.1 | | 1278 | | 4 | | 22 | 5.012966042 | | Up |
| NONMMUG085065.1 | NONMMUT134920.1 | | 2844 | | 2.12 | | 11.61 | 5.000566409 | | Up |
| LXLOC_008044 | LTCONS_00013719 | | 1066 | | 3.54 | | 19.27 | 4.983173417 | | Up |
| NONMMUG067675.1 | NONMMUT108213.1 | | 1051 | | 5 | | 27 | 4.960021619 | | Up |
| NONMMUG045595.2 | NONMMUT073529.2 | | 3133 | | 1.22 | | 6.51 | 4.925655596 | | Up |
| NONMMUG077938.1 | NONMMUT124312.1 | | 1365 | | 3 | | 16 | 4.924177803 | | Up |
| NONMMUG065724.1 | NONMMUT105096.1 | | 1812 | | 2.99 | | 15.82 | 4.901167225 | | Up |
| NONMMUG022798.2 | NONMMUT037030.2 | | 1817.78 | | 2.32 | | 12.06 | 4.850169198 | | Up |
| NONMMUG001964.2 | NONMMUT003031.2 | | 2829 | | 1.21 | | 6.24 | 4.827180768 | | Up |
| NONMMUG003659.2 | NONMMUT005647.2 | | 3889 | | 3.02 | | 15.54 | 4.820834903 | | Up |
| NONMMUG012874.2 | NONMMUT020692.2 | | 557 | | 7 | | 36 | 4.819242963 | | Up |
| NONMMUG018331.2 | NONMMUT029673.2 | | 720 | | 769.46 | | 3945.32 | #REF! | | Up |
| NONMMUG063176.1 | NONMMUT100990.1 | | 1031 | | 3.64 | | 18.35 | 4.76164222 | | Up |
| NONMMUG051463.1 | NONMMUT082682.1 | | 338 | | 2 | | 10 | 4.737958994 | | Up |
| NONMMUG059262.1 | NONMMUT094884.1 | | 1573 | | 2 | | 10 | 4.737958994 | | Up |
| NONMMUG052428.1 | NONMMUT084184.1 | | 993 | | 2 | | 10 | 4.737958994 | | Up |
| NONMMUG067353.1 | NONMMUT107721.1 | | 353 | | 2 | | 10 | 4.737958994 | | Up |
| NONMMUG029896.2 | NONMMUT048221.2 | | 808 | | 2 | | 10 | 4.737958994 | | Up |
| NONMMUG003383.2 | NONMMUT005247.2 | | 2990 | | 2 | | 10 | 4.737958994 | | Up |
| NONMMUG079427.1 | NONMMUT126601.1 | | 555 | | 4 | | 20 | 4.737958994 | | Up |
| NONMMUG027743.2 | NONMMUT044925.2 | | 3973 | | 4 | | 20 | 4.737958994 | | Up |
| NONMMUG009737.2 | NONMMUT015354.2 | | 2158 | | 6 | | 30 | 4.737958994 | | Up |
| NONMMUG012873.2 | NONMMUT020687.2 | | 1508 | | 16 | | 80 | 4.737958994 | | Up |
| NONMMUG006023.2 | NONMMUT009624.2 | | 2747 | | 1.28 | | 6.39 | 4.733447046 | | Up |
| NONMMUG067615.1 | NONMMUT108130.1 | | 653 | | 2 | | 9.85 | 4.694350254 | | Up |
| NONMMUG004676.2 | NONMMUT007320.2 | | 4872 | | 5.95 | | 29.15 | 4.679171424 | | Up |
| NONMMUG029803.2 | NONMMUT048035.2 | | 3615 | | 1.89 | | 9.25 | 4.676237067 | | Up |
| NONMMUG021083.2 | NONMMUT034294.2 | | 3573.53 | | 26.64 | | 129.66 | 4.660237785 | | Up |
| NONMMUG040188.2 | NONMMUT065054.2 | | 1658 | | 7 | | 34 | 4.654318643 | | Up |
| NONMMUG024709.2 | NONMMUT039932.2 | | 754 | | 6 | | 29 | 4.640139793 | | Up |
| LXLOC_059336 | LTCONS_00104106 | | 3017 | | 11.74 | | 56.47 | 4.62620725 | | Up |
| NONMMUG010663.2 | NONMMUT016967.2 | | 2555 | | 4.62 | | 22.12 | 4.612876062 | | Up |
| NONMMUG015149.2 | NONMMUT024502.2 | | 1398 | | 3.67 | | 17.53 | 4.60605086 | | Up |
| LXLOC_053176 | LTCONS_00093457 | | 4123 | | 13.09 | | 62.44 | 4.602113685 | | Up |
| NONMMUG040702.2 | NONMMUT065785.2 | | 1692.61 | | 8 | | 38 | 4.589957831 | | Up |
| NONMMUG021218.2 | NONMMUT034488.2 | | 699 | | 1.68 | | 7.97 | 4.586339787 | | Up |
| NONMMUG003131.2 | NONMMUT004885.2 | | 5940 | | 1.98 | | 9.35 | 4.573034674 | | Up |
| NONMMUG000956.2 | NONMMUT001411.2 | | 461 | | 2 | | 9.4 | 4.559424318 | | Up |
| NONMMUG002280.2 | NONMMUT003535.2 | | 1165.22 | | 33 | | 155 | 4.557563376 | | Up |
| NONMMUG031543.2 | NONMMUT050884.2 | | 1045 | | 13 | | 61 | 4.554698043 | | Up |
| NONMMUG045859.2 | NONMMUT073944.2 | | 1613 | | 1.58 | | 7.39 | 4.545402416 | | Up |
| NONMMUG058289.1 | NONMMUT093477.1 | | 380 | | 3 | | 14 | 4.538887647 | | Up |
| NONMMUG012714.2 | NONMMUT020467.2 | | 4404 | | 3 | | 14 | 4.538887647 | | Up |
| NONMMUG076576.1 | NONMMUT122111.1 | | 1257 | | 2 | | 9.32 | 4.534762714 | | Up |
| LXLOC_011970 | LTCONS_00020599 | | 3411 | | 7.94 | | 36.96 | 4.531610493 | | Up |
| NONMMUG039813.2 | NONMMUT064507.2 | | 1022 | | 3 | | 13.94 | 4.526495115 | | Up |
| NONMMUG041536.2 | NONMMUT067082.2 | | 2084.41 | | 5 | | 23 | 4.497370527 | | Up |
| LXLOC_025627 | LTCONS_00044603 | | 2278 | | 4.17 | | 19 | 4.469863064 | | Up |
| NONMMUG039676.2 | NONMMUT064287.2 | | 4778 | | 2 | | 9 | 4.433952807 | | Up |
| NONMMUG028123.2 | NONMMUT045561.2 | | 674 | | 2 | | 9 | 4.433952807 | | Up |
| NONMMUG085075.1 | NONMMUT134935.1 | | 1268 | | 2 | | 9 | 4.433952807 | | Up |
| NONMMUG052476.1 | NONMMUT084257.1 | | 1459 | | 2 | | 9 | 4.433952807 | | Up |
| NONMMUG070979.1 | NONMMUT113243.1 | | 977 | | 2 | | 9 | 4.433952807 | | Up |
| NONMMUG074785.1 | NONMMUT119206.1 | | 2075 | | 2 | | 9 | 4.433952807 | | Up |
| NONMMUG048361.1 | NONMMUT077922.1 | | 2245 | | 2 | | 9 | 4.433952807 | | Up |
| NONMMUG009153.2 | NONMMUT014448.2 | | 1749 | | 2 | | 9 | 4.433952807 | | Up |
| NONMMUG045454.2 | NONMMUT073297.2 | | 738 | | 2 | | 9 | 4.433952807 | | Up |
| NONMMUG073155.1 | NONMMUT116604.1 | | 728 | | 2 | | 9 | 4.433952807 | | Up |
| NONMMUG076703.1 | NONMMUT122295.1 | | 3462 | | 2 | | 9 | 4.433952807 | | Up |
| NONMMUG047850.1 | NONMMUT077163.1 | | 1097 | | 2 | | 9 | 4.433952807 | | Up |
| NONMMUG020605.2 | NONMMUT033483.2 | | 1531 | | 2.01 | | 9.04 | 4.432357347 | | Up |
| NONMMUG000958.2 | NONMMUT001413.2 | | 5837 | | 13 | | 58 | 4.409185358 | | Up |
| NONMMUG002285.2 | NONMMUT003544.2 | | 2282 | | 2.01 | | 8.85 | 4.371066712 | | Up |
| NONMMUG017047.2 | NONMMUT027497.2 | | 3479 | | 2.36 | | 10.38 | 4.367998162 | | Up |
| NONMMUG034201.2 | NONMMUT055100.2 | | 1910 | | 1.69 | | 7.38 | 4.347297944 | | Up |
| NONMMUG006008.2 | NONMMUT009602.2 | | 1072 | | 4.37 | | 19.06 | 4.343788673 | | Up |
| NONMMUG012870.2 | NONMMUT020683.2 | | 1432 | | 25 | | 109 | 4.342759074 | | Up |
| NONMMUG062927.1 | NONMMUT100600.1 | | 1088 | | 3 | | 13 | 4.325057239 | | Up |
| NONMMUG056218.1 | NONMMUT090266.1 | | 1110 | | 3 | | 13 | 4.325057239 | | Up |
| NONMMUG035850.2 | NONMMUT057716.2 | | 903 | | 3 | | 13 | 4.325057239 | | Up |
| NONMMUG002077.2 | NONMMUT003198.2 | | 1300 | | 3 | | 13 | 4.325057239 | | Up |
| NONMMUG038865.2 | NONMMUT062976.2 | | 2977 | | 3 | | 13 | 4.325057239 | | Up |
| NONMMUG002609.2 | NONMMUT004032.2 | | 1955 | | 2.01 | | 8.62 | 4.29508754 | | Up |
| NONMMUG035624.2 | NONMMUT057375.2 | | 5154 | | 7 | | 30 | 4.293174152 | | Up |
| NONMMUG019998.2 | NONMMUT032457.2 | | 2682 | | 13.31 | | 57 | 4.291005501 | | Up |
| LXLOC_062571 | LTCONS_00109453 | | 3072 | | 2.43 | | 10.4 | 4.289213423 | | Up |
| NONMMUG025262.2 | NONMMUT040754.2 | | 4917 | | 18.8 | | 80.39 | 4.286669582 | | Up |
| NONMMUG013359.2 | NONMMUT021524.2 | | 776 | | 15 | | 64 | 4.280321613 | | Up |
| NONMMUG007420.2 | NONMMUT011729.2 | | 3747 | | 3.01 | | 12.84 | 4.279722425 | | Up |
| NONMMUG034945.2 | NONMMUT056271.2 | | 305 | | 4 | | 17 | 4.269028487 | | Up |
| LXLOC_029540 | LTCONS_00051066 | | 3110 | | 10.74 | | 45.26 | 4.244587987 | | Up |
| NONMMUG072766.1 | NONMMUT116065.1 | | 724 | | 5 | | 21 | 4.23488146 | | Up |
| NONMMUG065255.1 | NONMMUT104328.1 | | 557 | | 5 | | 21 | 4.23488146 | | Up |
| NONMMUG032495.2 | NONMMUT052331.2 | | 4052 | | 5 | | 21 | 4.23488146 | | Up |
| NONMMUG016640.2 | NONMMUT026841.2 | | 4012 | | 2.11 | | 8.83 | 4.224443682 | | Up |
| NONMMUG064246.1 | NONMMUT102771.1 | | 2090 | | 4.69 | | 19.59 | 4.219017944 | | Up |
| NONMMUG067617.1 | NONMMUT108132.1 | | 1444 | | 8 | | 33 | 4.182891043 | | Up |
| NONMMUG066240.1 | NONMMUT105977.1 | | 372 | | 6.32 | | 26.03 | 4.178460501 | | Up |
| NONMMUG072443.1 | NONMMUT115504.1 | | 1564 | | 1.9 | | 7.82 | 4.176441182 | | Up |
| NONMMUG004873.2 | NONMMUT007699.2 | | 2358 | | 3.83 | | 15.61 | 4.148211285 | | Up |
| NONMMUG039868.2 | NONMMUT064587.2 | | 1856 | | 15 | | 61 | 4.141796288 | | Up |
| NONMMUG006151.2 | NONMMUT009813.2 | | 1905 | | 4.43 | | 18 | 4.13933941 | | Up |
| NONMMUG002161.2 | NONMMUT003319.2 | | 3325 | | 2.05 | | 8.28 | 4.12211652 | | Up |
| NONMMUG048348.1 | NONMMUT077900.1 | | 1452 | | 205.45 | | 829.77 | 4.12195114 | | Up |
| NONMMUG060134.1 | NONMMUT096193.1 | | 1221 | | 1.97 | | 7.95 | 4.119621266 | | Up |
| NONMMUG000155.2 | NONMMUT000222.2 | | 1089 | | 3.82 | | 15.32 | 4.101646312 | | Up |
| NONMMUG072074.1 | NONMMUT114954.1 | | 553 | | 2 | | 8 | 4.094102804 | | Up |
| NONMMUG010515.2 | NONMMUT016705.2 | | 2675 | | 2 | | 8 | 4.094102804 | | Up |
| NONMMUG042256.2 | NONMMUT068314.2 | | 3090 | | 2 | | 8 | 4.094102804 | | Up |
| NONMMUG032837.2 | NONMMUT052881.2 | | 233 | | 2 | | 8 | 4.094102804 | | Up |
| NONMMUG052940.1 | NONMMUT084985.1 | | 754 | | 2 | | 8 | 4.094102804 | | Up |
| NONMMUG075828.1 | NONMMUT120911.1 | | 1106 | | 2 | | 8 | 4.094102804 | | Up |
| NONMMUG069244.1 | NONMMUT110683.1 | | 929 | | 2 | | 8 | 4.094102804 | | Up |
| NONMMUG015081.2 | NONMMUT024398.2 | | 577 | | 2 | | 8 | 4.094102804 | | Up |
| NONMMUG009049.2 | NONMMUT014288.2 | | 1114 | | 2 | | 8 | 4.094102804 | | Up |
| NONMMUG016998.2 | NONMMUT027423.2 | | 2798 | | 2 | | 8 | 4.094102804 | | Up |
| NONMMUG005686.2 | NONMMUT009168.2 | | 1139 | | 2 | | 8 | 4.094102804 | | Up |
| NONMMUG071874.1 | NONMMUT114641.1 | | 717 | | 2 | | 8 | 4.094102804 | | Up |
| NONMMUG046982.1 | NONMMUT075738.1 | | 1018 | | 2 | | 8 | 4.094102804 | | Up |
| NONMMUG079292.1 | NONMMUT126390.1 | | 882 | | 2 | | 8 | 4.094102804 | | Up |
| NONMMUG041448.2 | NONMMUT066953.2 | | 989 | | 2 | | 8 | 4.094102804 | | Up |
| NONMMUG076705.1 | NONMMUT122297.1 | | 3569.51 | | 2 | | 8 | 4.094102804 | | Up |
| NONMMUG033397.2 | NONMMUT053834.2 | | 3542 | | 2 | | 8 | 4.094102804 | | Up |
| NONMMUG020820.2 | NONMMUT033896.2 | | 1803 | | 2 | | 8 | 4.094102804 | | Up |
| NONMMUG056385.1 | NONMMUT090487.1 | | 555 | | 2 | | 8 | 4.094102804 | | Up |
| NONMMUG009483.2 | NONMMUT014954.2 | | 3906 | | 2 | | 8 | 4.094102804 | | Up |
| NONMMUG063721.1 | NONMMUT101831.1 | | 1588 | | 2 | | 8 | 4.094102804 | | Up |
| NONMMUG080797.1 | NONMMUT128719.1 | | 1032 | | 2 | | 8 | 4.094102804 | | Up |
| NONMMUG020166.2 | NONMMUT032786.2 | | 1013 | | 2 | | 8 | 4.094102804 | | Up |
| NONMMUG052464.1 | NONMMUT084243.1 | | 700.08 | | 2 | | 8 | 4.094102804 | | Up |
| NONMMUG051063.1 | NONMMUT082024.1 | | 571 | | 2 | | 8 | 4.094102804 | | Up |
| NONMMUG059202.1 | NONMMUT094824.1 | | 1758 | | 2 | | 8 | 4.094102804 | | Up |
| NONMMUG021615.2 | NONMMUT035091.2 | | 1396 | | 2 | | 8 | 4.094102804 | | Up |
| NONMMUG059863.1 | NONMMUT095750.1 | | 542 | | 2 | | 8 | 4.094102804 | | Up |
| NONMMUG054239.1 | NONMMUT087076.1 | | 1202 | | 2 | | 8 | 4.094102804 | | Up |
| NONMMUG084909.1 | NONMMUT134740.1 | | 1563 | | 2 | | 8 | 4.094102804 | | Up |
| NONMMUG010057.2 | NONMMUT016003.2 | | 3021.66 | | 2 | | 8 | 4.094102804 | | Up |
| NONMMUG021605.2 | NONMMUT035077.2 | | 1485 | | 2 | | 8 | 4.094102804 | | Up |
| NONMMUG000150.2 | NONMMUT000215.2 | | 1543 | | 3 | | 12 | 4.094102804 | | Up |
| NONMMUG040513.2 | NONMMUT065505.2 | | 1034 | | 3 | | 12 | 4.094102804 | | Up |
| NONMMUG075829.1 | NONMMUT120913.1 | | 384 | | 3 | | 12 | 4.094102804 | | Up |
| NONMMUG035623.2 | NONMMUT057374.2 | | 1458 | | 3 | | 12 | 4.094102804 | | Up |
| NONMMUG071062.1 | NONMMUT113374.1 | | 531 | | 3 | | 12 | 4.094102804 | | Up |
| NONMMUG063299.1 | NONMMUT101169.1 | | 505 | | 3 | | 12 | 4.094102804 | | Up |
| NONMMUG076186.1 | NONMMUT121496.1 | | 594 | | 3 | | 12 | 4.094102804 | | Up |
| NONMMUG063815.1 | NONMMUT101971.1 | | 287 | | 3 | | 12 | 4.094102804 | | Up |
| NONMMUG035691.2 | NONMMUT057460.2 | | 528 | | 3 | | 12 | 4.094102804 | | Up |
| NONMMUG010007.2 | NONMMUT015933.2 | | 1813 | | 4 | | 16 | 4.094102804 | | Up |
| NONMMUG043580.2 | NONMMUT070412.2 | | 1032 | | 4 | | 16 | 4.094102804 | | Up |
| NONMMUG031039.2 | NONMMUT050132.2 | | 301 | | 4 | | 16 | 4.094102804 | | Up |
| NONMMUG008285.2 | NONMMUT013066.2 | | 2139.9 | | 5 | | 20 | 4.094102804 | | Up |
| NONMMUG009966.2 | NONMMUT015745.2 | | 1400.08 | | 5 | | 20 | 4.094102804 | | Up |
| NONMMUG063763.1 | NONMMUT101889.1 | | 856 | | 5 | | 20 | 4.094102804 | | Up |
| NONMMUG062851.1 | NONMMUT100464.1 | | 1739 | | 6 | | 24 | 4.094102804 | | Up |
| NONMMUG071559.1 | NONMMUT114097.1 | | 576 | | 7 | | 28 | 4.094102804 | | Up |
| NONMMUG015274.2 | NONMMUT024679.2 | | 2553 | | 7 | | 28 | 4.094102804 | | Up |
| NONMMUG060048.1 | NONMMUT096040.1 | | 972 | | 7 | | 28 | 4.094102804 | | Up |
| NONMMUG065810.1 | NONMMUT105252.1 | | 829 | | 2.03 | | 8.11 | 4.090547179 | | Up |
| NONMMUG068151.1 | NONMMUT108986.1 | | 1478 | | 2 | | 7.96 | 4.079639666 | | Up |
| NONMMUG069403.1 | NONMMUT110945.1 | | 705 | | 3.57 | | 14.18 | 4.073825911 | | Up |
| NONMMUG058108.1 | NONMMUT093199.1 | | 427 | | 2.16 | | 8.54 | 4.060512319 | | Up |
| NONMMUG079178.1 | NONMMUT126182.1 | | 2858 | | 2.26 | | 8.92 | 4.055544679 | | Up |
| NONMMUG031460.2 | NONMMUT050754.2 | | 2274 | | 2.27 | | 8.94 | 4.049267872 | | Up |
| NONMMUG027506.2 | NONMMUT044573.2 | | 437 | | 2.03 | | 7.97 | 4.040302798 | | Up |
| NONMMUG026364.2 | NONMMUT042564.2 | | 2206.11 | | 12 | | 47 | 4.033355506 | | Up |
| NONMMUG013671.2 | NONMMUT022007.2 | | 1786 | | 9 | | 35 | 4.012818835 | | Up |
| NONMMUG079422.1 | NONMMUT126593.1 | | 636 | | 2.92 | | 11.35 | 4.011406851 | | Up |
| NONMMUG043667.2 | NONMMUT070542.2 | | 1385 | | 7 | | 27 | 3.989167965 | | Up |
| NONMMUG029338.2 | NONMMUT047390.2 | | 3637 | | 29.13 | | 112.27 | 3.986892536 | | Up |
| NONMMUG007965.2 | NONMMUT012546.2 | | 1209 | | 2.73 | | 10.5 | 3.980935748 | | Up |
| NONMMUG021014.2 | NONMMUT034201.2 | | 1869.1 | | 71 | | 271.81 | 3.967518014 | | Up |
| NONMMUG001406.2 | NONMMUT002092.2 | | 3174 | | 3.26 | | 12.39 | 3.94656744 | | Up |
| NONMMUG005657.2 | NONMMUT009117.2 | | 1492.35 | | 3.24 | | 12.29 | 3.940941199 | | Up |
| NONMMUG034212.2 | NONMMUT055111.2 | | 3766 | | 24.82 | | 93.87 | 3.932424892 | | Up |
| NONMMUG008260.2 | NONMMUT013010.2 | | 3382.93 | | 109.85 | | 413.45 | 3.918458804 | | Up |
| NONMMUG074828.1 | NONMMUT119289.1 | | 748 | | 4.02 | | 15.09 | 3.910753603 | | Up |
| NONMMUG023007.2 | NONMMUT037310.2 | | 1687 | | 4.83 | | 18.13 | 3.910670466 | | Up |
| NONMMUG012425.2 | NONMMUT020032.2 | | 343 | | 4 | | 15 | 3.907883996 | | Up |
| NONMMUG066770.1 | NONMMUT106844.1 | | 2428 | | 4 | | 15 | 3.907883996 | | Up |
| NONMMUG041537.2 | NONMMUT067084.2 | | 2959 | | 15 | | 56 | 3.895031457 | | Up |
| NONMMUG030993.2 | NONMMUT050053.2 | | 1886 | | 6.33 | | 23.53 | 3.882550636 | | Up |
| NONMMUG011550.2 | NONMMUT018582.2 | | 3742 | | 2.7 | | 10 | 3.87204018 | | Up |
| NONMMUG078099.1 | NONMMUT124598.1 | | 631 | | 2.48 | | 9.14 | 3.857810894 | | Up |
| NONMMUG009788.2 | NONMMUT015433.2 | | 347 | | 3 | | 11 | 3.84304104 | | Up |
| NONMMUG069506.1 | NONMMUT111113.1 | | 2753 | | 3 | | 11 | 3.84304104 | | Up |
| NONMMUG049722.1 | NONMMUT079936.1 | | 718 | | 3 | | 11 | 3.84304104 | | Up |
| NONMMUG050875.1 | NONMMUT081729.1 | | 713 | | 3 | | 11 | 3.84304104 | | Up |
| NONMMUG002773.2 | NONMMUT004285.2 | | 1539 | | 3 | | 11 | 3.84304104 | | Up |
| NONMMUG028103.2 | NONMMUT045536.2 | | 2249 | | 3 | | 11 | 3.84304104 | | Up |
| NONMMUG079104.1 | NONMMUT126076.1 | | 1061 | | 3 | | 11 | 3.84304104 | | Up |
| NONMMUG028847.2 | NONMMUT046655.2 | | 1286 | | 3 | | 11 | 3.84304104 | | Up |
| NONMMUG036709.2 | NONMMUT059076.2 | | 749 | | 3 | | 11 | 3.84304104 | | Up |
| NONMMUG066677.1 | NONMMUT106691.1 | | 636 | | 6 | | 22 | 3.84304104 | | Up |
| NONMMUG075724.1 | NONMMUT120720.1 | | 368 | | 7.68 | | 27.96 | 3.822475094 | | Up |
| NONMMUG077273.1 | NONMMUT123202.1 | | 897 | | 11 | | 40 | 3.819095757 | | Up |
| NONMMUG023597.2 | NONMMUT038207.2 | | 301 | | 8.29 | | 30.07 | 3.811864527 | | Up |
| NONMMUG060133.1 | NONMMUT096188.1 | | 738.56 | | 8 | | 29 | 3.810064795 | | Up |
| LXLOC_008374 | LTCONS_00014584 | | 2863 | | 36.6 | | 132.54 | 3.807127346 | | Up |
| NONMMUG077125.1 | NONMMUT122964.1 | | 1949 | | 5 | | 18 | 3.790096618 | | Up |
| NONMMUG034527.2 | NONMMUT055625.2 | | 3070 | | 5 | | 18 | 3.790096618 | | Up |
| NONMMUG047094.1 | NONMMUT075898.1 | | 1234 | | 5 | | 18 | 3.790096618 | | Up |
| NONMMUG041331.2 | NONMMUT066780.2 | | 2352 | | 12 | | 43 | 3.776707312 | | Up |
| NONMMUG031205.2 | NONMMUT050382.2 | | 1534.23 | | 32.26 | | 115.53 | 3.775001175 | | Up |
| NONMMUG001288.2 | NONMMUT001914.2 | | 538.93 | | 26 | | 93 | 3.77154099 | | Up |
| NONMMUG066001.1 | NONMMUT105561.1 | | 1505.15 | | 3 | | 10.7 | 3.763255586 | | Up |
| NONMMUG004303.2 | NONMMUT006683.2 | | 3762.31 | | 38.36 | | 136.76 | 3.762046209 | | Up |
| NONMMUG035399.2 | NONMMUT057041.2 | | 1996.02 | | 18 | | 64 | 3.754252802 | | Up |
| NONMMUG002514.2 | NONMMUT003902.2 | | 2435 | | 29 | | 103 | 3.751141869 | | Up |
| NONMMUG044058.2 | NONMMUT071133.2 | | 5315 | | 4.64 | | 16.42 | 3.740617638 | | Up |
| NONMMUG046007.2 | NONMMUT074178.2 | | 2158 | | 2.47 | | 8.73 | 3.737044029 | | Up |
| NONMMUG037723.2 | NONMMUT060691.2 | | 2580 | | 4 | | 14 | 3.708812649 | | Up |
| NONMMUG013682.2 | NONMMUT022030.2 | | 397 | | 4 | | 14 | 3.708812649 | | Up |
| NONMMUG007648.2 | NONMMUT012094.2 | | 719.11 | | 4 | | 14 | 3.708812649 | | Up |
| NONMMUG025922.2 | NONMMUT041875.2 | | 2478 | | 6 | | 21 | 3.708812649 | | Up |
| NONMMUG073971.1 | NONMMUT117896.1 | | 714 | | 14 | | 49 | 3.708812649 | | Up |
| NONMMUG040945.2 | NONMMUT066160.2 | | 3220 | | 4 | | 13.99 | 3.706750919 | | Up |
| NONMMUG060573.1 | NONMMUT096920.1 | | 7616 | | 27 | | 94 | 3.693505503 | | Up |
| NONMMUG074343.1 | NONMMUT118453.1 | | 1764 | | 3.11 | | 10.79 | 3.683519563 | | Up |
| NONMMUG040419.2 | NONMMUT065368.2 | | 1693 | | 10.32 | | 35.73 | 3.677488691 | | Up |
| NONMMUG043366.2 | NONMMUT070057.2 | | 898.21 | | 6.07 | | 21 | 3.675344617 | | Up |
| NONMMUG023085.2 | NONMMUT037434.2 | | 2576 | | 9 | | 31 | 3.662645422 | | Up |
| NONMMUG071149.1 | NONMMUT113509.1 | | 747 | | 3.06 | | 10.53 | 3.659906561 | | Up |
| NONMMUG032724.2 | NONMMUT052698.2 | | 3378 | | 3.9 | | 13.39 | 3.653322668 | | Up |
| NONMMUG038669.2 | NONMMUT062675.2 | | 1244 | | 6.47 | | 22.11 | 3.63982562 | | Up |
| NONMMUG076779.1 | NONMMUT122408.1 | | 1903 | | 6.18 | | 21.06 | 3.631756191 | | Up |
| NONMMUG084345.1 | NONMMUT133866.1 | | 1072 | | 5.09 | | 17.32 | 3.627505542 | | Up |
| NONMMUG001397.2 | NONMMUT002081.2 | | 1662 | | 5 | | 17 | 3.625172297 | | Up |
| NONMMUG065517.1 | NONMMUT104720.1 | | 4946 | | 5 | | 17 | 3.625172297 | | Up |
| NONMMUG001142.2 | NONMMUT001695.2 | | 1685 | | 5 | | 17 | 3.625172297 | | Up |
| NONMMUG026120.2 | NONMMUT112676.1 | | 4539 | | 5 | | 17 | 3.625172297 | | Up |
| NONMMUG025304.2 | NONMMUT040834.2 | | 417 | | 15 | | 51 | 3.625172297 | | Up |
| NONMMUG030988.2 | NONMMUT050040.2 | | 922 | | 4 | | 13.55 | 3.614544697 | | Up |
| NONMMUG065096.1 | NONMMUT104101.1 | | 2215 | | 3.56 | | 12 | 3.600273324 | | Up |
| NONMMUG086992.1 | NONMMUT137556.1 | | 960 | | 3 | | 10.11 | 3.599599987 | | Up |
| NONMMUG000470.2 | NONMMUT000677.2 | | 950 | | 5.36 | | 18.05 | 3.597490667 | | Up |
| LXLOC_009358 | LTCONS_00016068 | | 2030 | | 13.63 | | 45.85 | 3.594375148 | | Up |
| NONMMUG015581.2 | NONMMUT025179.2 | | 3331 | | 37 | | 124 | 3.583588694 | | Up |
| NONMMUG026457.2 | NONMMUT042743.2 | | 4401 | | 2.99 | | 10 | 3.577668025 | | Up |
| LXLOC_037823 | LTCONS_00065817 | | 4626 | | 187.39 | | 626.12 | 3.574893243 | | Up |
| LXLOC_034054 | LTCONS_00059095 | | 1256 | | 10.06 | | 33.61 | 3.574623279 | | Up |
| NONMMUG008261.2 | NONMMUT013012.2 | | 4381 | | 124 | | 414 | 3.572684099 | | Up |
| NONMMUG027706.2 | NONMMUT044861.2 | | 1139 | | 3.03 | | 10.11 | 3.570889401 | | Up |
| NONMMUG010136.2 | NONMMUT016143.2 | | 3192 | | 3 | | 10 | 3.568033993 | | Up |
| NONMMUG073979.1 | NONMMUT117907.1 | | 727 | | 3 | | 10 | 3.568033993 | | Up |
| NONMMUG040548.2 | NONMMUT065567.2 | | 1497 | | 3 | | 10 | 3.568033993 | | Up |
| NONMMUG018492.2 | NONMMUT029931.2 | | 2579 | | 3 | | 10 | 3.568033993 | | Up |
| NONMMUG074880.1 | NONMMUT119375.1 | | 1849 | | 3 | | 10 | 3.568033993 | | Up |
| NONMMUG077272.1 | NONMMUT123197.1 | | 1096 | | 3 | | 10 | 3.568033993 | | Up |
| NONMMUG013396.2 | NONMMUT021579.2 | | 698 | | 3 | | 10 | 3.568033993 | | Up |
| NONMMUG022564.2 | NONMMUT036665.2 | | 663 | | 3 | | 10 | 3.568033993 | | Up |
| NONMMUG069645.1 | NONMMUT111301.1 | | 811 | | 3 | | 10 | 3.568033993 | | Up |
| NONMMUG019981.2 | NONMMUT032438.2 | | 3347 | | 3 | | 10 | 3.568033993 | | Up |
| NONMMUG017139.2 | NONMMUT027659.2 | | 827 | | 3 | | 10 | 3.568033993 | | Up |
| NONMMUG056477.1 | NONMMUT090620.1 | | 1200 | | 3 | | 10 | 3.568033993 | | Up |
| NONMMUG062310.1 | NONMMUT099603.1 | | 807 | | 3 | | 10 | 3.568033993 | | Up |
| NONMMUG007185.2 | NONMMUT011371.2 | | 309.18 | | 3 | | 10 | 3.568033993 | | Up |
| NONMMUG054403.1 | NONMMUT087343.1 | | 1281 | | 3 | | 10 | 3.568033993 | | Up |
| NONMMUG071648.1 | NONMMUT114229.1 | | 299 | | 6 | | 20 | 3.568033993 | | Up |
| NONMMUG051249.1 | NONMMUT082338.1 | | 867 | | 6 | | 20 | 3.568033993 | | Up |
| NONMMUG071246.1 | NONMMUT113664.1 | | 2853 | | 6 | | 20 | 3.568033993 | | Up |
| NONMMUG067943.1 | NONMMUT108692.1 | | 324 | | 12 | | 40 | 3.568033993 | | Up |
| NONMMUG002896.2 | NONMMUT004491.2 | | 1026 | | 3.08 | | 10.25 | 3.563346112 | | Up |
| LXLOC_048132 | LTCONS_00084570 | | 1636 | | 3.89 | | 12.93 | 3.559863234 | | Up |
| NONMMUG004004.2 | NONMMUT006217.2 | | 1141 | | 13 | | 43 | 3.545752878 | | Up |
| NONMMUG023274.2 | NONMMUT037701.2 | | 1280 | | 3.23 | | 10.65 | 3.536597525 | | Up |
| NONMMUG031410.2 | NONMMUT050683.2 | | 1989 | | 7 | | 23 | 3.526516872 | | Up |
| NONMMUG019142.2 | NONMMUT031076.2 | | 1799 | | 7 | | 23 | 3.526516872 | | Up |
| NONMMUG039531.2 | NONMMUT064032.2 | | 4907 | | 123 | | 404 | 3.525496759 | | Up |
| NONMMUG020366.2 | NONMMUT033108.2 | | 1016 | | 11 | | 36 | 3.51508957 | | Up |
| NONMMUG041053.2 | NONMMUT066322.2 | | 2516 | | 2.95 | | 9.64 | 3.510739188 | | Up |
| NONMMUG043670.2 | NONMMUT070549.2 | | 3436.4 | | 78.07 | | 254.15 | 3.499782916 | | Up |
| LXLOC_025438 | LTCONS_00044295 | | 5317 | | 3.21 | | 10.44 | 3.497055823 | | Up |
| NONMMUG063515.1 | NONMMUT101506.1 | | 1451 | | 4 | | 13 | 3.494982241 | | Up |
| NONMMUG013509.2 | NONMMUT021735.2 | | 764 | | 4 | | 13 | 3.494982241 | | Up |
| NONMMUG001493.2 | NONMMUT002223.2 | | 554.2 | | 4 | | 13 | 3.494982241 | | Up |
| NONMMUG085213.1 | NONMMUT135116.1 | | 846 | | 4 | | 13 | 3.494982241 | | Up |
| NONMMUG058783.1 | NONMMUT094154.1 | | 441 | | 4 | | 13 | 3.494982241 | | Up |
| NONMMUG039678.2 | NONMMUT064291.2 | | 518 | | 4 | | 13 | 3.494982241 | | Up |
| NONMMUG077260.1 | NONMMUT123179.1 | | 1024 | | 4 | | 13 | 3.494982241 | | Up |
| NONMMUG061936.1 | NONMMUT099004.1 | | 1246 | | 4 | | 13 | 3.494982241 | | Up |
| NONMMUG043631.2 | NONMMUT070487.2 | | 2759 | | 4 | | 13 | 3.494982241 | | Up |
| NONMMUG021734.2 | NONMMUT035271.2 | | 1699 | | 4 | | 13 | 3.494982241 | | Up |
| NONMMUG040315.2 | NONMMUT065220.2 | | 2949 | | 4 | | 13 | 3.494982241 | | Up |
| NONMMUG037251.2 | NONMMUT059966.2 | | 2753 | | 4 | | 13 | 3.494982241 | | Up |
| NONMMUG065141.1 | NONMMUT104157.1 | | 1560 | | 8 | | 26 | 3.494982241 | | Up |
| NONMMUG037731.2 | NONMMUT060703.2 | | 1666 | | 8 | | 26 | 3.494982241 | | Up |
| NONMMUG062375.1 | NONMMUT099706.1 | | 623.68 | | 5.2 | | 16.88 | 3.491565556 | | Up |
| NONMMUG040369.2 | NONMMUT065295.2 | | 2093 | | 4 | | 12.97 | 3.488315953 | | Up |
| NONMMUG034701.2 | NONMMUT055881.2 | | 1707 | | 6.73 | | 21.82 | 3.488048188 | | Up |
| NONMMUG043859.2 | NONMMUT070831.2 | | 2272 | | 3.75 | | 12.15 | 3.486090431 | | Up |
| NONMMUG023059.2 | NONMMUT037396.2 | | 482 | | 7 | | 22.67 | 3.484817932 | | Up |
| NONMMUG018191.2 | NONMMUT029440.2 | | 3122 | | 238.11 | | 767.5 | 3.471179883 | | Up |
| NONMMUG080904.1 | NONMMUT128866.1 | | 852 | | 9.27 | | 29.81 | 3.464413067 | | Up |
| NONMMUG059095.1 | NONMMUT094655.1 | | 4109 | | 9 | | 28.9 | 3.460247977 | | Up |
| NONMMUG029858.2 | NONMMUT048144.2 | | 2705 | | 13.16 | | 42.14 | 3.452164455 | | Up |
| NONMMUG064773.1 | NONMMUT103605.1 | | 2038 | | 5 | | 16 | 3.450246615 | | Up |
| NONMMUG043902.2 | NONMMUT070888.2 | | 813 | | 5 | | 16 | 3.450246615 | | Up |
| NONMMUG078206.1 | NONMMUT124763.1 | | 1286 | | 5 | | 16 | 3.450246615 | | Up |
| NONMMUG074179.1 | NONMMUT118189.1 | | 1249 | | 5 | | 16 | 3.450246615 | | Up |
| NONMMUG005913.2 | NONMMUT009473.2 | | 3057.15 | | 5 | | 16 | 3.450246615 | | Up |
| NONMMUG050465.1 | NONMMUT081143.1 | | 1345 | | 5 | | 15.94 | 3.439406063 | | Up |
| NONMMUG042683.2 | NONMMUT068951.2 | | 1528.01 | | 5 | | 15.94 | 3.439406063 | | Up |
| NONMMUG082557.1 | NONMMUT131155.1 | | 945 | | 3.54 | | 11.28 | 3.437994409 | | Up |
| NONMMUG072767.1 | NONMMUT116067.1 | | 1146 | | 12 | | 38 | 3.42003283 | | Up |
| LXLOC_046484 | LTCONS_00081627 | | 1553 | | 14.24 | | 44.82 | 3.40248981 | | Up |
| NONMMUG023569.2 | NONMMUT038165.2 | | 688.84 | | 7 | | 22 | 3.398256198 | | Up |
| NONMMUG010981.2 | NONMMUT017501.2 | | 229 | | 3.46 | | 10.86 | 3.394463125 | | Up |
| NONMMUG061529.1 | NONMMUT098442.1 | | 810 | | 4.97 | | 15.59 | 3.392709147 | | Up |
| NONMMUG000744.2 | NONMMUT001118.2 | | 1783 | | 9.83 | | 30.73 | 3.382871695 | | Up |
| NONMMUG020352.2 | NONMMUT033091.2 | | 2331 | | 8 | | 25 | 3.381815184 | | Up |
| NONMMUG043002.2 | NONMMUT069425.2 | | 1844 | | 8 | | 25 | 3.381815184 | | Up |
| NONMMUG007254.2 | NONMMUT011477.2 | | 1288 | | 9 | | 28 | 3.368962646 | | Up |
| NONMMUG015910.2 | NONMMUT025683.2 | | 649 | | 9 | | 28 | 3.368962646 | | Up |
| NONMMUG023862.2 | NONMMUT038645.2 | | 1295 | | 9 | | 28 | 3.368962646 | | Up |
| NONMMUG059208.1 | NONMMUT094830.1 | | 1789 | | 10.44 | | 32.4 | 3.361847007 | | Up |
| NONMMUG076697.1 | NONMMUT122288.1 | | 543 | | 10 | | 31 | 3.358639235 | | Up |
| NONMMUG012533.2 | NONMMUT020199.2 | | 1313.7 | | 65 | | 201 | 3.351470561 | | Up |
| NONMMUG004693.2 | NONMMUT007343.2 | | 3382.33 | | 4 | | 12.34 | 3.344643783 | | Up |
| NONMMUG032160.2 | NONMMUT051852.2 | | 1666 | | 3.87 | | 11.92 | 3.340060333 | | Up |
| NONMMUG054735.1 | NONMMUT087892.1 | | 1708 | | 13 | | 40 | 3.337079558 | | Up |
| NONMMUG021052.2 | NONMMUT034251.2 | | 666 | | 7.04 | | 21.58 | 3.326197866 | | Up |
| NONMMUG002604.2 | NONMMUT076887.1 | | 7350 | | 17 | | 52 | 3.320056558 | | Up |
| NONMMUG035690.2 | NONMMUT057459.2 | | 3515 | | 7.5 | | 22.91 | 3.31613272 | | Up |
| NONMMUG036964.2 | NONMMUT059478.2 | | 1588 | | 3.6 | | 10.97 | 3.309092233 | | Up |
| NONMMUG001083.2 | NONMMUT001621.2 | | 3522 | | 6.08 | | 18.52 | 3.307984544 | | Up |
| LXLOC_003365 | LTCONS_00005857 | | 1924 | | 14.38 | | 43.76 | 3.305200929 | | Up |
| NONMMUG078280.1 | NONMMUT124884.1 | | 1559 | | 7.05 | | 21.45 | 3.304667775 | | Up |
| NONMMUG042223.2 | NONMMUT068261.2 | | 520 | | 7.53 | | 22.78 | 3.288194759 | | Up |
| NONMMUG012801.2 | NONMMUT020592.2 | | 699.64 | | 8.38 | | 25.22 | 3.273195058 | | Up |
| LXLOC_070804 | LTCONS_00124163 | | 3859 | | 4 | | 12 | 3.264027806 | | Up |
| NONMMUG014047.2 | NONMMUT022727.2 | | 1561 | | 4 | | 12 | 3.264027806 | | Up |
| NONMMUG005538.2 | NONMMUT008927.2 | | 2666.6 | | 4 | | 12 | 3.264027806 | | Up |
| NONMMUG076833.1 | NONMMUT122493.1 | | 1413 | | 4 | | 12 | 3.264027806 | | Up |
| NONMMUG080743.1 | NONMMUT128641.1 | | 345 | | 4 | | 12 | 3.264027806 | | Up |
| NONMMUG077146.1 | NONMMUT122998.1 | | 753 | | 4 | | 12 | 3.264027806 | | Up |
| NONMMUG013640.2 | NONMMUT021962.2 | | 498 | | 4 | | 12 | 3.264027806 | | Up |
| NONMMUG038357.2 | NONMMUT062169.2 | | 1020 | | 5 | | 15 | 3.264027806 | | Up |
| NONMMUG025502.2 | NONMMUT041152.2 | | 1750 | | 5 | | 15 | 3.264027806 | | Up |
| NONMMUG010592.2 | NONMMUT016836.2 | | 2873 | | 5 | | 15 | 3.264027806 | | Up |
| NONMMUG063143.1 | NONMMUT100938.1 | | 1252 | | 5 | | 15 | 3.264027806 | | Up |
| NONMMUG068141.1 | NONMMUT108972.1 | | 3007 | | 5 | | 15 | 3.264027806 | | Up |
| NONMMUG080481.1 | NONMMUT128199.1 | | 705 | | 6 | | 18 | 3.264027806 | | Up |
| NONMMUG010978.2 | NONMMUT017496.2 | | 2194 | | 6 | | 18 | 3.264027806 | | Up |
| NONMMUG084867.1 | NONMMUT134642.1 | | 399.16 | | 6 | | 18 | 3.264027806 | | Up |
| NONMMUG026816.2 | NONMMUT043422.2 | | 315 | | 6.97 | | 20.91 | 3.264027806 | | Up |
| NONMMUG021955.2 | NONMMUT035593.2 | | 1140.14 | | 7 | | 21 | 3.264027806 | | Up |
| NONMMUG078482.1 | NONMMUT125215.1 | | 3300 | | 7 | | 21 | 3.264027806 | | Up |
| NONMMUG014975.2 | NONMMUT024228.2 | | 3607 | | 10 | | 30 | 3.264027806 | | Up |
| NONMMUG052326.1 | NONMMUT084037.1 | | 1007.17 | | 10 | | 30 | 3.264027806 | | Up |
| NONMMUG046940.1 | NONMMUT075672.1 | | 1504.09 | | 14 | | 42 | 3.264027806 | | Up |
| NONMMUG018089.2 | NONMMUT029258.2 | | 474 | | 179.84 | | 538.91 | 3.260763638 | | Up |
| NONMMUG005543.2 | NONMMUT008934.2 | | 1493 | | 4.76 | | 14.21 | 3.249848956 | | Up |
| NONMMUG005961.2 | NONMMUT009534.2 | | 270.74 | | 42.67 | | 126.97 | 3.240490098 | | Up |
| NONMMUG018245.2 | NONMMUT029522.2 | | 3107 | | 80.16 | | 238.09 | 3.235208032 | | Up |
| NONMMUG021735.2 | NONMMUT035274.2 | | 3701 | | 77 | | 228.69 | 3.235028667 | | Up |
| NONMMUG018187.2 | NONMMUT029433.2 | | 1336 | | 267.95 | | 795.6 | 3.234261725 | | Up |
| LXLOC_074420 | LTCONS_00130537 | | 6565 | | 9.92 | | 29.45 | 3.233814021 | | Up |
| NONMMUG002354.2 | NONMMUT003664.2 | | 2800 | | 16.49 | | 48.86 | 3.228227734 | | Up |
| LXLOC_018992 | LTCONS_00032988 | | 1444 | | 26.45 | | 78.29 | 3.225223447 | | Up |
| NONMMUG081071.1 | NONMMUT129132.1 | | 975 | | 292 | | 864 | 3.224228691 | | Up |
| LXLOC_070360 | LTCONS_00123342 | | 5574.34 | | 20.75 | | 61.34 | 3.221536465 | | Up |
| NONMMUG080138.1 | NONMMUT127661.1 | | 812 | | 6 | | 17.72 | 3.218791201 | | Up |
| NONMMUG038712.2 | NONMMUT062731.2 | | 592 | | 7.06 | | 20.78 | 3.209013936 | | Up |
| NONMMUG046905.1 | NONMMUT075610.1 | | 501 | | 13.86 | | 40.69 | 3.20159685 | | Up |
| NONMMUG050912.1 | NONMMUT081791.1 | | 499 | | 3.99 | | 11.62 | 3.178401639 | | Up |
| LXLOC_044540 | LTCONS_00078280 | | 30211.97 | | 4.93 | | 14.35 | 3.176885175 | | Up |
| NONMMUG079748.1 | NONMMUT127078.1 | | 2576 | | 11 | | 32 | 3.175239567 | | Up |
| NONMMUG034044.2 | NONMMUT054866.2 | | 1284.17 | | 52.53 | | 152.67 | 3.17250902 | | Up |
| NONMMUG010903.2 | NONMMUT017369.2 | | 3502 | | 10 | | 29 | 3.166208605 | | Up |
| NONMMUG084799.1 | NONMMUT134514.1 | | 2996 | | 10 | | 29 | 3.166208605 | | Up |
| NONMMUG031546.2 | NONMMUT050887.2 | | 657.07 | | 60 | | 174 | 3.166208605 | | Up |
| NONMMUG012464.2 | NONMMUT020099.2 | | 2878 | | 10.77 | | 31.13 | 3.156677458 | | Up |
| NONMMUG012111.2 | NONMMUT019512.2 | | 547 | | 9 | | 26 | 3.155132238 | | Up |
| NONMMUG069737.1 | NONMMUT111427.1 | | 1311 | | 17 | | 49 | 3.14859681 | | Up |
| LXLOC_017405 | LTCONS_00030173 | | 1236 | | 3.86 | | 11.1 | 3.141876652 | | Up |
| NONMMUG066709.1 | NONMMUT106733.1 | | 1021 | | 8 | | 23 | 3.141226717 | | Up |
| NONMMUG043363.2 | NONMMUT070054.2 | | 585.98 | | 8 | | 23 | 3.141226717 | | Up |
| NONMMUG081205.1 | NONMMUT129335.1 | | 1532 | | 10.96 | | 31.37 | 3.128378266 | | Up |
| NONMMUG039184.2 | NONMMUT063446.2 | | 1294 | | 29 | | 83 | 3.128219677 | | Up |
| NONMMUG004302.2 | NONMMUT006672.2 | | 3864 | | 36.58 | | 104.61 | 3.125890384 | | Up |
| NONMMUG040198.2 | NONMMUT065069.2 | | 340 | | 13 | | 37 | 3.112130099 | | Up |
| NONMMUG025018.2 | NONMMUT040378.2 | | 2181.59 | | 51.91 | | 147.72 | 3.111664354 | | Up |
| NONMMUG083090.1 | NONMMUT132015.1 | | 8038 | | 5.65 | | 16.02 | 3.101205555 | | Up |
| NONMMUG035622.2 | NONMMUT057373.2 | | 1299 | | 6 | | 17 | 3.099103486 | | Up |
| NONMMUG040854.2 | NONMMUT066018.2 | | 2333 | | 40 | | 113 | 3.09060454 | | Up |
| NONMMUG003232.2 | NONMMUT005038.2 | | 910.76 | | 11 | | 31 | 3.083632188 | | Up |
| NONMMUG050556.1 | NONMMUT081318.1 | | 10646 | | 5.89 | | 16.59 | 3.082051499 | | Up |
| NONMMUG031549.2 | NONMMUT050893.2 | | 5677 | | 21 | | 59 | 3.074754058 | | Up |
| NONMMUG010565.2 | NONMMUT016794.2 | | 1890 | | 6.06 | | 16.98 | 3.066996325 | | Up |
| NONMMUG081639.1 | NONMMUT129943.1 | | 311 | | 5 | | 14 | 3.064956459 | | Up |
| NONMMUG037439.2 | NONMMUT060241.2 | | 1355 | | 5 | | 14 | 3.064956459 | | Up |
| NONMMUG042450.2 | NONMMUT068576.2 | | 825 | | 5 | | 14 | 3.064956459 | | Up |
| NONMMUG059956.1 | NONMMUT095881.1 | | 1840 | | 5 | | 14 | 3.064956459 | | Up |
| NONMMUG063175.1 | NONMMUT100987.1 | | 484 | | 5 | | 14 | 3.064956459 | | Up |
| NONMMUG063111.1 | NONMMUT100882.1 | | 1209 | | 5 | | 14 | 3.064956459 | | Up |
| NONMMUG063040.1 | NONMMUT100769.1 | | 935 | | 5 | | 14 | 3.064956459 | | Up |
| NONMMUG032023.2 | NONMMUT051639.2 | | 759 | | 20 | | 56 | 3.064956459 | | Up |
| NONMMUG070464.1 | NONMMUT112548.1 | | 1957 | | 7.64 | | 21.39 | 3.064686683 | | Up |
| NONMMUG016921.2 | NONMMUT100045.1 | | 6624.03 | | 26.1 | | 72.84 | 3.055465036 | | Up |
| NONMMUG007818.2 | NONMMUT012342.2 | | 1941.6 | | 90 | | 251 | 3.05348372 | | Up |
| NONMMUG027534.2 | NONMMUT044610.2 | | 991 | | 10.66 | | 29.72 | 3.05255616 | | Up |
| NONMMUG022667.2 | NONMMUT036825.2 | | 991 | | 10.66 | | 29.72 | 3.05255616 | | Up |
| NONMMUG011154.2 | NONMMUT017793.2 | | 2840 | | 14 | | 39 | 3.050197398 | | Up |
| NONMMUG009088.2 | NONMMUT014344.2 | | 991 | | 10.67 | | 29.72 | 3.049850684 | | Up |
| NONMMUG019644.2 | NONMMUT031948.2 | | 991 | | 10.67 | | 29.72 | 3.049850684 | | Up |
| NONMMUG036487.2 | NONMMUT058691.2 | | 991 | | 10.67 | | 29.7 | 3.047908314 | | Up |
| NONMMUG069528.1 | NONMMUT111149.1 | | 554 | | 9 | | 25 | 3.041965181 | | Up |
| NONMMUG042060.2 | NONMMUT067944.2 | | 2459 | | 5.63 | | 15.63 | 3.040324706 | | Up |
| NONMMUG003937.2 | NONMMUT006107.2 | | 2628 | | 26 | | 72 | 3.033073371 | | Up |
| NONMMUG046032.2 | NONMMUT074218.2 | | 553 | | 39 | | 108 | 3.033073371 | | Up |
| NONMMUG062132.1 | NONMMUT099309.1 | | 1307 | | 5.06 | | 14.01 | 3.032598136 | | Up |
| NONMMUG047425.1 | NONMMUT076453.1 | | 2883 | | 7.98 | | 22.09 | 3.031968339 | | Up |
| NONMMUG019681.2 | NONMMUT032002.2 | | 1416 | | 6.8 | | 18.78 | 3.025283627 | | Up |
| NONMMUG006090.2 | NONMMUT009730.2 | | 918 | | 4.34 | | 11.98 | 3.023824725 | | Up |
| NONMMUG039397.2 | NONMMUT063796.2 | | 1329 | | 33 | | 91 | 3.020903846 | | Up |
| NONMMUG061594.1 | NONMMUT098528.1 | | 2003 | | 13.39 | | 36.87 | 3.016685716 | | Up |
| LXLOC_059587 | LTCONS_00104513 | | 1236 | | 8.92 | | 24.56 | 3.016492695 | | Up |
| NONMMUG042217.2 | NONMMUT068243.2 | | 903 | | 5.48 | | 15.07 | 3.012966042 | | Up |
| NONMMUG045641.2 | NONMMUT073633.2 | | 1354 | | 8 | | 22 | 3.012966042 | | Up |
| LXLOC_006696 | LTCONS_00011341 | | 1977 | | 8.49 | | 23.32 | 3.009565464 | | Up |
| NONMMUG034874.2 | NONMMUT056128.2 | | 4529.94 | | 32.7 | | 89.4 | 2.996071196 | | Up |
| NONMMUG055637.1 | NONMMUT089321.1 | | 655 | | 8.59 | | 23.48 | 2.995507548 | | Up |
| NONMMUG074874.1 | NONMMUT119364.1 | | 2818 | | 14.97 | | 40.89 | 2.993450487 | | Up |
| NONMMUG013305.2 | NONMMUT021431.2 | | 2703 | | 11 | | 30 | 2.989020758 | | Up |
| NONMMUG034244.2 | NONMMUT055159.2 | | 1269.32 | | 25 | | 68 | 2.981316107 | | Up |
| NONMMUG053588.1 | NONMMUT086120.1 | | 218 | | 7 | | 19 | 2.975247987 | | Up |
| NONMMUG001197.2 | NONMMUT001786.2 | | 2771 | | 14 | | 38 | 2.975247987 | | Up |
| NONMMUG016731.2 | NONMMUT026990.2 | | 1015 | | 8.05 | | 21.81 | 2.969960968 | | Up |
| LXLOC_039090 | LTCONS_00068275 | | 2654 | | 7.16 | | 19.38 | 2.967176961 | | Up |
| NONMMUG031551.2 | NONMMUT050898.2 | | 3137.45 | | 119 | | 322 | 2.966301034 | | Up |
| LXLOC_018343 | LTCONS_00031819 | | 25928.79 | | 90.69 | | 244.94 | 2.960928821 | | Up |
| NONMMUG001992.2 | NONMMUT003082.2 | | 1455 | | 10 | | 27 | 2.960021619 | | Up |
| NONMMUG020461.2 | NONMMUT033250.2 | | 1974 | | 10 | | 27 | 2.960021619 | | Up |
| NONMMUG080929.1 | NONMMUT128907.1 | | 830 | | 8.29 | | 22.34 | 2.954473162 | | Up |
| NONMMUG032199.2 | NONMMUT051922.2 | | 1469 | | 56.71 | | 152.5 | 2.948371167 | | Up |
| NONMMUG043984.2 | NONMMUT071021.2 | | 656 | | 7.01 | | 18.84 | 2.946728036 | | Up |
| NONMMUG009195.2 | NONMMUT014521.2 | | 1606.62 | | 16 | | 43 | 2.946632314 | | Up |
| NONMMUG032672.2 | NONMMUT052618.2 | | 511 | | 4.9 | | 13.15 | 2.942521095 | | Up |
| LXLOC_069299 | LTCONS_00121478 | | 3515 | | 7.5 | | 20.09 | 2.937132931 | | Up |
| NONMMUG025750.2 | NONMMUT041632.2 | | 1083 | | 28 | | 75 | 2.937030341 | | Up |
| NONMMUG033889.2 | NONMMUT054652.2 | | 2409 | | 15.49 | | 41.39 | 2.929993013 | | Up |
| NONMMUG034548.2 | NONMMUT055664.2 | | 1354 | | 7.69 | | 20.51 | 2.924646782 | | Up |
| NONMMUG021904.2 | NONMMUT035523.2 | | 2341 | | 6 | | 16 | 2.924177803 | | Up |
| NONMMUG022793.2 | NONMMUT037021.2 | | 1408 | | 6 | | 16 | 2.924177803 | | Up |
| NONMMUG011998.2 | NONMMUT019359.2 | | 639 | | 6 | | 16 | 2.924177803 | | Up |
| NONMMUG035006.2 | NONMMUT056360.2 | | 635 | | 5.48 | | 14.6 | 2.921543946 | | Up |
| NONMMUG080987.1 | NONMMUT129001.1 | | 1830.28 | | 7.14 | | 19 | 2.918109683 | | Up |
| NONMMUG002488.2 | NONMMUT003867.2 | | 815 | | 6.83 | | 18.15 | 2.914126934 | | Up |
| NONMMUG039587.2 | NONMMUT064127.2 | | 911 | | 14 | | 37 | 2.898299692 | | Up |
| LXLOC_021932 | LTCONS_00038112 | | 3493 | | 8.56 | | 22.6 | 2.895382946 | | Up |
| LXLOC_042857 | LTCONS_00075157 | | 3175 | | 190.88 | | 502.04 | 2.884375539 | | Up |
| NONMMUG016178.2 | NONMMUT026133.2 | | 2213.56 | | 8 | | 21 | 2.87873765 | | Up |
| LXLOC_076556 | LTCONS_00134070 | | 751 | | 9.3 | | 24.37 | 2.873710064 | | Up |
| LXLOC_060238 | LTCONS_00105545 | | 2298 | | 8.85 | | 23.17 | 2.871120172 | | Up |
| NONMMUG009965.2 | NONMMUT015735.2 | | 2301 | | 13.56 | | 35.49 | 2.870213596 | | Up |
| NONMMUG072606.1 | NONMMUT115803.1 | | 490 | | 5.52 | | 14.4 | 2.860760084 | | Up |
| NONMMUG025934.2 | NONMMUT041896.2 | | 2102 | | 5.04 | | 13.14 | 2.859042078 | | Up |
| NONMMUG000730.2 | NONMMUT001091.2 | | 413.06 | | 5 | | 13 | 2.851126051 | | Up |
| NONMMUG033195.2 | NONMMUT053477.2 | | 2385 | | 5 | | 13 | 2.851126051 | | Up |
| NONMMUG077572.1 | NONMMUT123698.1 | | 1244 | | 5 | | 13 | 2.851126051 | | Up |
| NONMMUG039886.2 | NONMMUT064609.2 | | 848 | | 5 | | 13 | 2.851126051 | | Up |
| NONMMUG067199.1 | NONMMUT107490.1 | | 1776.31 | | 5 | | 13 | 2.851126051 | | Up |
| NONMMUG083436.1 | NONMMUT132530.1 | | 566 | | 5 | | 13 | 2.851126051 | | Up |
| NONMMUG045259.2 | NONMMUT073022.2 | | 286 | | 5 | | 13 | 2.851126051 | | Up |
| NONMMUG030162.2 | NONMMUT048597.2 | | 4446 | | 10 | | 26 | 2.851126051 | | Up |
| NONMMUG006564.2 | NONMMUT010451.2 | | 2992 | | 10 | | 26 | 2.851126051 | | Up |
| NONMMUG003832.2 | NONMMUT005932.2 | | 1801 | | 17 | | 44 | 2.838040359 | | Up |
| NONMMUG002493.2 | NONMMUT003876.2 | | 1425.92 | | 29 | | 75 | 2.835778195 | | Up |
| NONMMUG001754.2 | NONMMUT002598.2 | | 1299 | | 12 | | 31 | 2.832570424 | | Up |
| NONMMUG054205.1 | NONMMUT087031.1 | | 1297 | | 9.9 | | 25.46 | 2.819566782 | | Up |
| NONMMUG019921.2 | NONMMUT032357.2 | | 3910 | | 7 | | 18 | 2.819242963 | | Up |
| NONMMUG069658.1 | NONMMUT111314.1 | | 1397 | | 7 | | 18 | 2.819242963 | | Up |
| NONMMUG055712.1 | NONMMUT089424.1 | | 1587 | | 7 | | 18 | 2.819242963 | | Up |
| NONMMUG008431.2 | NONMMUT013288.2 | | 669 | | 7 | | 18 | 2.819242963 | | Up |
| NONMMUG077767.1 | NONMMUT124061.1 | | 1124 | | 7 | | 18 | 2.819242963 | | Up |
| NONMMUG069294.1 | NONMMUT110753.1 | | 791.76 | | 14 | | 36 | 2.819242963 | | Up |
| LXLOC_052011 | LTCONS_00091290 | | 4238 | | 9.07 | | 23.21 | 2.805246938 | | Up |
| NONMMUG012203.2 | NONMMUT019651.2 | | 1511 | | 8.6 | | 22 | 2.804292722 | | Up |
| NONMMUG025658.2 | NONMMUT041439.2 | | 1174 | | 21.19 | | 54.19 | 2.803388923 | | Up |
| NONMMUG035093.2 | NONMMUT056508.2 | | 1217 | | 9 | | 23 | 2.801376714 | | Up |
| NONMMUG084267.1 | NONMMUT133778.1 | | 213 | | 9 | | 23 | 2.801376714 | | Up |
| NONMMUG050874.1 | NONMMUT081727.1 | | 2063 | | 9 | | 23 | 2.801376714 | | Up |
| NONMMUG001763.2 | NONMMUT002608.2 | | 2957 | | 18 | | 46 | 2.801376714 | | Up |
| NONMMUG031677.2 | NONMMUT051122.2 | | 1715 | | 5.15 | | 13.13 | 2.794547962 | | Up |
| NONMMUG002230.2 | NONMMUT003467.2 | | 3053 | | 7.58 | | 19.31 | 2.792259626 | | Up |
| LXLOC_075395 | LTCONS_00132006 | | 3173 | | 24.67 | | 62.56 | 2.779068398 | | Up |
| NONMMUG024065.2 | NONMMUT038965.2 | | 3019 | | 15 | | 38 | 2.77617664 | | Up |
| NONMMUG034241.2 | NONMMUT055156.2 | | 2762 | | 15 | | 38 | 2.77617664 | | Up |
| NONMMUG013889.2 | NONMMUT022519.2 | | 585 | | 15 | | 38 | 2.77617664 | | Up |
| NONMMUG008259.2 | NONMMUT013009.2 | | 218 | | 32 | | 81 | 2.77380281 | | Up |
| NONMMUG083716.1 | NONMMUT132978.1 | | 3520 | | 10.69 | | 26.95 | 2.762149644 | | Up |
| NONMMUG019380.2 | NONMMUT031540.2 | | 1388 | | 25 | | 63 | 2.760950272 | | Up |
| NONMMUG041534.2 | NONMMUT067079.2 | | 3388 | | 26.84 | | 67.63 | 2.760660169 | | Up |
| NONMMUG013119.2 | NONMMUT021126.2 | | 2037 | | 296 | | 745 | 2.757389304 | | Up |
| NONMMUG049208.1 | NONMMUT079096.1 | | 1252 | | 5.57 | | 13.99 | 2.751396264 | | Up |
| NONMMUG057675.1 | NONMMUT092533.1 | | 277 | | 6 | | 15 | 2.737958994 | | Up |
| NONMMUG040766.2 | NONMMUT065888.2 | | 300 | | 6 | | 15 | 2.737958994 | | Up |
| NONMMUG040108.2 | NONMMUT064944.2 | | 1655 | | 22 | | 55 | 2.737958994 | | Up |
| NONMMUG038924.2 | NONMMUT063066.2 | | 609 | | 38 | | 95 | 2.737958994 | | Up |
| NONMMUG053509.1 | NONMMUT085973.1 | | 2754 | | 9.18 | | 22.94 | 2.73670147 | | Up |
| LXLOC_068837 | LTCONS_00120676 | | 12711 | | 8.18 | | 20.4 | 2.730895612 | | Up |
| NONMMUG086517.1 | NONMMUT136949.1 | | 3496 | | 15.01 | | 37.4 | 2.728331391 | | Up |
| NONMMUG033689.2 | NONMMUT054313.2 | | 3039.68 | | 19.35 | | 48.21 | 2.728100531 | | Up |
| NONMMUG032058.2 | NONMMUT051690.2 | | 2050 | | 29 | | 72 | 2.717990817 | | Up |
| NONMMUG047581.1 | NONMMUT076710.1 | | 11978 | | 96 | | 238 | 2.71381333 | | Up |
| NONMMUG042928.2 | NONMMUT069313.2 | | 1710 | | 11.27 | | 27.94 | 2.713791815 | | Up |
| NONMMUG010038.2 | NONMMUT015978.2 | | 4150 | | 7.21 | | 17.87 | 2.713039744 | | Up |
| NONMMUG018970.2 | NONMMUT030750.2 | | 3041.27 | | 8.01 | | 19.83 | 2.709723864 | | Up |
| NONMMUG017701.2 | NONMMUT028608.2 | | 712 | | 17 | | 42 | 2.703811968 | | Up |
| NONMMUG034408.2 | NONMMUT055411.2 | | 4230 | | 17 | | 42 | 2.703811968 | | Up |
| NONMMUG031547.2 | NONMMUT050889.2 | | 3665 | | 193.92 | | 477.61 | 2.694845684 | | Up |
| NONMMUG042533.2 | NONMMUT132339.1 | | 5326 | | 10.88 | | 26.73 | 2.687665366 | | Up |
| NONMMUG038483.2 | NONMMUT062393.2 | | 1191.22 | | 16.57 | | 40.65 | 2.683466304 | | Up |
| NONMMUG076818.1 | NONMMUT122464.1 | | 453 | | 20 | | 49 | 2.679666303 | | Up |
| NONMMUG067942.1 | NONMMUT108691.1 | | 377 | | 169 | | 414 | 2.679317847 | | Up |
| NONMMUG026031.2 | NONMMUT042049.2 | | 3331 | | 9 | | 22 | 2.673116039 | | Up |
| NONMMUG080717.1 | NONMMUT128589.1 | | 1229 | | 16.05 | | 39.23 | 2.672870881 | | Up |
| NONMMUG000390.2 | NONMMUT000562.2 | | 1418.24 | | 7.03 | | 17.17 | 2.670689694 | | Up |
| NONMMUG081240.1 | NONMMUT129376.1 | | 3414 | | 9.03 | | 22 | 2.663514066 | | Up |
| NONMMUG038699.2 | NONMMUT062711.2 | | 1357.38 | | 6.89 | | 16.74 | 2.655550084 | | Up |
| NONMMUG016976.2 | NONMMUT098986.1 | | 5810.97 | | 7.71 | | 18.73 | 2.655199073 | | Up |
| NONMMUG008296.2 | NONMMUT013083.2 | | 2324 | | 6.25 | | 15.18 | 2.654590196 | | Up |
| NONMMUG079201.1 | NONMMUT126216.1 | | 866 | | 7 | | 17 | 2.654318643 | | Up |
| NONMMUG024501.2 | NONMMUT039611.2 | | 1364 | | 7 | | 17 | 2.654318643 | | Up |
| NONMMUG028699.2 | NONMMUT046457.2 | | 619 | | 7 | | 17 | 2.654318643 | | Up |
| NONMMUG000413.2 | NONMMUT000601.2 | | 1505 | | 7 | | 17 | 2.654318643 | | Up |
| NONMMUG012381.2 | NONMMUT019944.2 | | 2171 | | 7 | | 17 | 2.654318643 | | Up |
| NONMMUG032603.2 | NONMMUT052516.2 | | 3706 | | 7 | | 17 | 2.654318643 | | Up |
| NONMMUG032993.2 | NONMMUT053121.2 | | 3306 | | 38 | | 92 | 2.64537169 | | Up |
| NONMMUG074389.1 | NONMMUT118541.1 | | 354 | | 12 | | 29 | 2.640139793 | | Up |
| NONMMUG005279.2 | NONMMUT084793.1 | | 1003 | | 12 | | 29 | 2.640139793 | | Up |
| NONMMUG041535.2 | NONMMUT067081.2 | | 2982.67 | | 65 | | 157 | 2.638608676 | | Up |
| NONMMUG036832.2 | NONMMUT059279.2 | | 2345 | | 34 | | 82 | 2.634281131 | | Up |
| NONMMUG038290.2 | NONMMUT061973.2 | | 1804.47 | | 20.61 | | 49.66 | 2.631582318 | | Up |
| NONMMUG046944.1 | NONMMUT075679.1 | | 810 | | 10 | | 24 | 2.620171616 | | Up |
| NONMMUG084007.1 | NONMMUT133329.1 | | 2732 | | 10 | | 24 | 2.620171616 | | Up |
| NONMMUG006920.2 | NONMMUT010963.2 | | 3975 | | 50 | | 120 | 2.620171616 | | Up |
| NONMMUG042198.2 | NONMMUT068220.2 | | 2619 | | 6.25 | | 14.93 | 2.606674946 | | Up |
| NONMMUG085255.1 | NONMMUT135205.1 | | 1097 | | 13 | | 31 | 2.601615989 | | Up |
| NONMMUG037334.2 | NONMMUT060084.2 | | 905 | | 26 | | 62 | 2.601615989 | | Up |
| NONMMUG039635.2 | NONMMUT064205.2 | | 2860.19 | | 78 | | 186 | 2.601615989 | | Up |
| NONMMUG058676.1 | NONMMUT094000.1 | | 2097 | | 6.6 | | 15.73 | 2.600064287 | | Up |
| NONMMUG028397.2 | NONMMUT046001.2 | | 676 | | 12.71 | | 30.28 | 2.598905154 | | Up |
| NONMMUG013126.2 | NONMMUT021137.2 | | 1975 | | 19.53 | | 46.47 | 2.595318196 | | Up |
| NONMMUG063140.1 | NONMMUT100934.1 | | 1795 | | 29 | | 69 | 2.595189728 | | Up |
| NONMMUG080136.1 | NONMMUT127659.1 | | 342 | | 8 | | 19 | 2.589957831 | | Up |
| NONMMUG042592.2 | NONMMUT068821.2 | | 1401 | | 14.53 | | 34.44 | 2.584203684 | | Up |
| NONMMUG035274.2 | NONMMUT056833.2 | | 1877.51 | | 11 | | 26 | 2.576119003 | | Up |
| NONMMUG086059.1 | NONMMUT136353.1 | | 759 | | 9.12 | | 21.51 | 2.569906395 | | Up |
| NONMMUG048907.1 | NONMMUT078667.1 | | 870 | | 20.39 | | 48.08 | 2.569253046 | | Up |
| NONMMUG023057.2 | NONMMUT037394.2 | | 2940 | | 59 | | 139 | 2.566698851 | | Up |
| LXLOC_002239 | LTCONS_00004014 | | 5461 | | 97.8 | | 230.32 | 2.565569452 | | Up |
| NONMMUG017224.2 | NONMMUT027791.2 | | 547.11 | | 57 | | 134 | 2.560501157 | | Up |
| NONMMUG021011.2 | NONMMUT034191.2 | | 2131 | | 463 | | 1084 | 2.548664121 | | Up |
| NONMMUG027293.2 | NONMMUT044223.2 | | 1496 | | 47 | | 110 | 2.547644528 | | Up |
| LXLOC_047371 | LTCONS_00083178 | | 3638 | | 78.53 | | 183.73 | 2.546645627 | | Up |
| LXLOC_042055 | LTCONS_00073629 | | 2314 | | 36.48 | | 85.28 | 2.544306222 | | Up |
| NONMMUG048326.1 | NONMMUT077866.1 | | 817 | | 9 | | 21 | 2.538887647 | | Up |
| NONMMUG055325.1 | NONMMUT088811.1 | | 334 | | 9 | | 21 | 2.538887647 | | Up |
| NONMMUG007894.2 | NONMMUT012454.2 | | 367 | | 9 | | 21 | 2.538887647 | | Up |
| NONMMUG077026.1 | NONMMUT122790.1 | | 620 | | 9 | | 21 | 2.538887647 | | Up |
| NONMMUG001331.2 | NONMMUT001993.2 | | 1355 | | 12 | | 28 | 2.538887647 | | Up |
| NONMMUG018239.2 | NONMMUT029513.2 | | 2164.52 | | 241.48 | | 561.76 | 2.530203195 | | Up |
| NONMMUG001201.2 | NONMMUT001794.2 | | 2405 | | 12 | | 27.85 | 2.523388648 | | Up |
| NONMMUG039708.2 | NONMMUT064352.2 | | 1493 | | 13 | | 30 | 2.507004559 | | Up |
| NONMMUG036770.2 | NONMMUT059176.2 | | 700 | | 13 | | 30 | 2.507004559 | | Up |
| NONMMUG050068.1 | NONMMUT080473.1 | | 1291.04 | | 16.81 | | 38.75 | 2.503855976 | | Up |
| NONMMUG012441.2 | NONMMUT020061.2 | | 1051 | | 23 | | 53 | 2.502819801 | | Up |
| NONMMUG000156.2 | NONMMUT000223.2 | | 3169 | | 33 | | 76 | 2.501169593 | | Up |
| LXLOC_018411 | LTCONS_00032014 | | 2692 | | 223.56 | | 514.66 | 2.500017962 | | Up |
| LXLOC_068516 | LTCONS_00120176 | | 1891 | | 17.12 | | 39.39 | 2.498396235 | | Up |
| NONMMUG059769.1 | NONMMUT095596.1 | | 789 | | 7.53 | | 17.32 | 2.497537125 | | Up |
| NONMMUG027429.2 | NONMMUT044448.2 | | 2527 | | 10 | | 23 | 2.497370527 | | Up |
| NONMMUG024332.2 | NONMMUT039372.2 | | 3393 | | 20 | | 46 | 2.497370527 | | Up |
| NONMMUG047976.1 | NONMMUT077337.1 | | 496 | | 20 | | 46 | 2.497370527 | | Up |
| NONMMUG016241.2 | NONMMUT026245.2 | | 2129 | | 17.66 | | 40.61 | 2.496802173 | | Up |
| NONMMUG043584.2 | NONMMUT070416.2 | | 884 | | 36.01 | | 82.79 | 2.496220643 | | Up |
| NONMMUG043160.2 | NONMMUT069683.2 | | 2471 | | 7.54 | | 17.33 | 2.495373256 | | Up |
| NONMMUG043632.2 | NONMMUT070490.2 | | 880 | | 7.74 | | 17.78 | 2.49380251 | | Up |
| NONMMUG043668.2 | NONMMUT070544.2 | | 1568 | | 13.82 | | 31.72 | 2.491373115 | | Up |
| NONMMUG076162.1 | NONMMUT121449.1 | | 1247 | | 8.86 | | 20.33 | 2.490566027 | | Up |
| NONMMUG002078.2 | NONMMUT003200.2 | | 2558.73 | | 45 | | 103 | 2.483397666 | | Up |
| NONMMUG055270.1 | NONMMUT088715.1 | | 2157 | | 18.78 | | 42.98 | 2.483039644 | | Up |
| NONMMUG050164.1 | NONMMUT080620.1 | | 887 | | 7 | | 16 | 2.47939296 | | Up |
| NONMMUG051667.1 | NONMMUT083015.1 | | 375 | | 7 | | 16 | 2.47939296 | | Up |
| NONMMUG028747.2 | NONMMUT046527.2 | | 358 | | 7 | | 16 | 2.47939296 | | Up |
| NONMMUG055401.1 | NONMMUT088905.1 | | 709 | | 21 | | 48 | 2.47939296 | | Up |
| NONMMUG063882.1 | NONMMUT102059.1 | | 754 | | 71 | | 162 | 2.474308571 | | Up |
| LXLOC_082082 | LTCONS_00143542 | | 743 | | 14.25 | | 32.46 | 2.469504966 | | Up |
| NONMMUG011109.2 | NONMMUT017717.2 | | 488 | | 11 | | 25 | 2.462951947 | | Up |
| LXLOC_055382 | LTCONS_00097178 | | 1963 | | 10.22 | | 23.19 | 2.458318052 | | Up |
| NONMMUG080550.1 | NONMMUT128304.1 | | 712 | | 9.86 | | 22.37 | 2.457914214 | | Up |
| NONMMUG059199.1 | NONMMUT094820.1 | | 8720 | | 19 | | 43 | 2.450777287 | | Up |
| NONMMUG018030.2 | NONMMUT029155.2 | | 3897 | | 9.9 | | 22.36 | 2.44494232 | | Up |
| NONMMUG021282.2 | NONMMUT034613.2 | | 889 | | 8 | | 18 | 2.433952807 | | Up |
| NONMMUG062081.1 | NONMMUT099218.1 | | 659 | | 8 | | 18 | 2.433952807 | | Up |
| NONMMUG078653.1 | NONMMUT125499.1 | | 599 | | 8 | | 18 | 2.433952807 | | Up |
| NONMMUG025094.2 | NONMMUT040510.2 | | 822 | | 8 | | 18 | 2.433952807 | | Up |
| NONMMUG070091.1 | NONMMUT111945.1 | | 311.76 | | 8 | | 18 | 2.433952807 | | Up |
| NONMMUG005393.2 | NONMMUT008718.2 | | 920.11 | | 12 | | 27 | 2.433952807 | | Up |
| NONMMUG019975.2 | NONMMUT032430.2 | | 517.88 | | 12 | | 27 | 2.433952807 | | Up |
| NONMMUG071063.1 | NONMMUT113376.1 | | 517 | | 12 | | 27 | 2.433952807 | | Up |
| NONMMUG073403.1 | NONMMUT117020.1 | | 1121 | | 12 | | 27 | 2.433952807 | | Up |
| NONMMUG003541.2 | NONMMUT005495.2 | | 4129 | | 20 | | 45 | 2.433952807 | | Up |
| LXLOC_087531 | LTCONS_00152501 | | 1655 | | 7.81 | | 17.56 | 2.431899587 | | Up |
| NONMMUG002678.2 | NONMMUT004139.2 | | 3042 | | 16.97 | | 38.12 | 2.429225913 | | Up |
| NONMMUG053577.1 | NONMMUT086101.1 | | 1034 | | 13.27 | | 29.79 | 2.42742231 | | Up |
| NONMMUG041656.2 | NONMMUT067284.2 | | 1821 | | 37.05 | | 83.14 | 2.426241215 | | Up |
| NONMMUG036396.2 | NONMMUT058552.2 | | 2095 | | 15.12 | | 33.89 | 2.422905799 | | Up |
| NONMMUG041545.2 | NONMMUT067095.2 | | 728.58 | | 29 | | 65 | 2.42287644 | | Up |
| NONMMUG030204.2 | NONMMUT048665.2 | | 2727 | | 9.56 | | 21.39 | 2.417810723 | | Up |
| NONMMUG061434.1 | NONMMUT098301.1 | | 1151 | | 11.98 | | 26.79 | 2.416236151 | | Up |
| NONMMUG052568.1 | NONMMUT084410.1 | | 772 | | 123 | | 275 | 2.415649411 | | Up |
| NONMMUG047417.1 | NONMMUT076445.1 | | 1713 | | 16.33 | | 36.49 | 2.414055525 | | Up |
| NONMMUG014417.2 | NONMMUT023313.2 | | 3945 | | 13 | | 29 | 2.409185358 | | Up |
| NONMMUG026293.2 | NONMMUT042423.2 | | 510.09 | | 26 | | 58 | 2.409185358 | | Up |
| NONMMUG013428.2 | NONMMUT021619.2 | | 2322.15 | | 9.01 | | 20.09 | 2.40785991 | | Up |
| NONMMUG023321.2 | NONMMUT037767.2 | | 2788 | | 35 | | 78 | 2.406341208 | | Up |
| NONMMUG066481.1 | NONMMUT106398.1 | | 1362 | | 11.26 | | 25.08 | 2.404763846 | | Up |
| NONMMUG046739.1 | NONMMUT075373.1 | | 1044.15 | | 16.01 | | 35.62 | 2.401531222 | | Up |
| NONMMUG068251.1 | NONMMUT109139.1 | | 377 | | 9 | | 20 | 2.398108991 | | Up |
| NONMMUG042733.2 | NONMMUT069024.2 | | 968 | | 18 | | 40 | 2.398108991 | | Up |
| NONMMUG020322.2 | NONMMUT033012.2 | | 2145 | | 145 | | 322 | 2.396118381 | | Up |
| NONMMUG074032.1 | NONMMUT117981.1 | | 762 | | 19.45 | | 43.11 | 2.390607619 | | Up |
| NONMMUG002922.2 | NONMMUT004534.2 | | 2263 | | 19 | | 42 | 2.382882623 | | Up |
| NONMMUG070658.1 | NONMMUT112786.1 | | 889 | | 40.05 | | 88.37 | 2.377611687 | | Up |
| NONMMUG006334.2 | NONMMUT010096.2 | | 1134 | | 7.74 | | 17.07 | 2.376217978 | | Up |
| NONMMUG026471.2 | NONMMUT042767.2 | | 1198.29 | | 10 | | 22 | 2.369109852 | | Up |
| NONMMUG030519.2 | NONMMUT049291.2 | | 1466 | | 10 | | 22 | 2.369109852 | | Up |
| NONMMUG059203.1 | NONMMUT094825.1 | | 2118 | | 10 | | 22 | 2.369109852 | | Up |
| NONMMUG026363.2 | NONMMUT042562.2 | | 3390 | | 15 | | 33 | 2.369109852 | | Up |
| NONMMUG042728.2 | NONMMUT069019.2 | | 2948 | | 8.8 | | 19.26 | 2.354167353 | | Up |
| NONMMUG080063.1 | NONMMUT127554.1 | | 1303 | | 11 | | 24 | 2.345164569 | | Up |
| NONMMUG074589.1 | NONMMUT118881.1 | | 2712 | | 10.43 | | 22.75 | 2.344357579 | | Up |
| NONMMUG038403.2 | NONMMUT062265.2 | | 4408.58 | | 335 | | 730 | 2.341573541 | | Up |
| NONMMUG027751.2 | NONMMUT044937.2 | | 846 | | 10.42 | | 22.66 | 2.335687972 | | Up |
| NONMMUG055908.1 | NONMMUT089751.1 | | 386.23 | | 13.94 | | 30.28 | 2.332372093 | | Up |
| NONMMUG017134.2 | NONMMUT027654.2 | | 1252 | | 65 | | 141 | 2.328469883 | | Up |
| NONMMUG026865.2 | NONMMUT043487.2 | | 1180 | | 12 | | 26 | 2.325057239 | | Up |
| NONMMUG027055.2 | NONMMUT043769.2 | | 4512 | | 12 | | 26 | 2.325057239 | | Up |
| NONMMUG073854.1 | NONMMUT117704.1 | | 1287 | | 67 | | 145 | 2.321742604 | | Up |
| LXLOC_013503 | LTCONS_00023324 | | 2713 | | 79.46 | | 171.81 | 2.319129426 | | Up |
| NONMMUG036835.2 | NONMMUT059284.2 | | 1744 | | 31 | | 67 | 2.317888565 | | Up |
| NONMMUG030146.2 | NONMMUT048575.2 | | 2224 | | 9.63 | | 20.78 | 2.313278707 | | Up |
| NONMMUG006004.2 | NONMMUT009598.2 | | 1868 | | 41.03 | | 88.41 | 2.309163618 | | Up |
| NONMMUG016431.2 | NONMMUT026537.2 | | 662.5 | | 13 | | 28 | 2.307933212 | | Up |
| NONMMUG011501.2 | NONMMUT018494.2 | | 1806 | | 22.38 | | 48.17 | 2.30595258 | | Up |
| NONMMUG055984.1 | NONMMUT089894.1 | | 9887 | | 141.61 | | 304.69 | 2.304940813 | | Up |
| NONMMUG033258.2 | NONMMUT053587.2 | | 537 | | 13.98 | | 30 | 2.297299084 | | Up |
| NONMMUG051813.1 | NONMMUT083239.1 | | 267 | | 14 | | 30 | 2.293174152 | | Up |
| NONMMUG034242.2 | NONMMUT055157.2 | | 1287 | | 21 | | 45 | 2.293174152 | | Up |
| NONMMUG035913.2 | NONMMUT057831.2 | | 2694 | | 28 | | 60 | 2.293174152 | | Up |
| NONMMUG031284.2 | NONMMUT050494.2 | | 1359.89 | | 36 | | 77 | 2.287825883 | | Up |
| NONMMUG004053.2 | NONMMUT006299.2 | | 1098 | | 36.47 | | 77.65 | 2.274654254 | | Up |
| LXLOC_021819 | LTCONS_00037915 | | 3720 | | 14.45 | | 30.75 | 2.27313664 | | Up |
| NONMMUG077879.1 | NONMMUT124218.1 | | 3439 | | 9.58 | | 20.37 | 2.270799643 | | Up |
| NONMMUG039393.2 | NONMMUT063787.2 | | 3330 | | 58 | | 123 | 2.263169825 | | Up |
| NONMMUG012377.2 | NONMMUT019937.2 | | 1535.08 | | 25 | | 53 | 2.262231334 | | Up |
| LXLOC_062538 | LTCONS_00109367 | | 3482 | | 48.26 | | 102 | 2.253441468 | | Up |
| NONMMUG027579.2 | NONMMUT044669.2 | | 1652.76 | | 9 | | 19 | 2.250107828 | | Up |
| NONMMUG019658.2 | NONMMUT031971.2 | | 968 | | 9 | | 19 | 2.250107828 | | Up |
| NONMMUG017370.2 | NONMMUT028036.2 | | 298 | | 9 | | 19 | 2.250107828 | | Up |
| NONMMUG020579.2 | NONMMUT033444.2 | | 613 | | 217 | | 458 | 2.249407916 | | Up |
| NONMMUG030449.2 | NONMMUT049194.2 | | 1057 | | 9.15 | | 19.28 | 2.244625611 | | Up |
| LXLOC_065585 | LTCONS_00114911 | | 1251 | | 44.21 | | 93.13 | 2.243849293 | | Up |
| NONMMUG026079.2 | NONMMUT042126.2 | | 2551 | | 187.38 | | 394.6 | 2.242950766 | | Up |
| NONMMUG002187.2 | NONMMUT003370.2 | | 758 | | 19 | | 40 | 2.242103967 | | Up |
| NONMMUG030650.2 | NONMMUT049505.2 | | 3233 | | 10 | | 21 | 2.23488146 | | Up |
| NONMMUG037029.2 | NONMMUT059584.2 | | 2062 | | 10 | | 21 | 2.23488146 | | Up |
| NONMMUG075770.1 | NONMMUT120799.1 | | 390 | | 10 | | 21 | 2.23488146 | | Up |
| NONMMUG039558.2 | NONMMUT064077.2 | | 2876 | | 20 | | 42 | 2.23488146 | | Up |
| NONMMUG040120.2 | NONMMUT064961.2 | | 993 | | 40 | | 84 | 2.23488146 | | Up |
| NONMMUG023946.2 | NONMMUT038773.2 | | 511 | | 21 | | 44 | 2.228331196 | | Up |
| NONMMUG084755.1 | NONMMUT134443.1 | | 1305 | | 93 | | 194 | 2.215610867 | | Up |
| NONMMUG027333.2 | NONMMUT044295.2 | | 1673 | | 303 | | 632 | 2.215316334 | | Up |
| NONMMUG043905.2 | NONMMUT070893.2 | | 3148 | | 35 | | 73 | 2.215185888 | | Up |
| NONMMUG036196.2 | NONMMUT124214.1 | | 3715 | | 23.28 | | 48.55 | 2.214863279 | | Up |
| NONMMUG066563.1 | NONMMUT106523.1 | | 735 | | 17.12 | | 35.69 | 2.213777204 | | Up |
| NONMMUG065699.1 | NONMMUT105060.1 | | 663 | | 12 | | 25 | 2.211890183 | | Up |
| NONMMUG021966.2 | NONMMUT035727.2 | | 2671 | | 62 | | 129 | 2.208164695 | | Up |
| NONMMUG075391.1 | NONMMUT120204.1 | | 1998 | | 12.54 | | 26.08 | 2.206915847 | | Up |
| NONMMUG041520.2 | NONMMUT067061.2 | | 3567 | | 165.11 | | 342 | 2.195240453 | | Up |
| NONMMUG045742.2 | NONMMUT073774.2 | | 1424 | | 58 | | 120 | 2.191922005 | | Up |
| NONMMUG079059.1 | NONMMUT126014.1 | | 1153 | | 44 | | 91 | 2.190828848 | | Up |
| NONMMUG051712.1 | NONMMUT083084.1 | | 824 | | 15 | | 31 | 2.188714234 | | Up |
| NONMMUG052300.1 | NONMMUT083993.1 | | 749 | | 16.77 | | 34.65 | 2.188048131 | | Up |
| NONMMUG027336.2 | NONMMUT044301.2 | | 1291 | | 1030 | | 2128 | 2.187810431 | | Up |
| NONMMUG026762.2 | NONMMUT043346.2 | | 2102 | | 25.27 | | 52.19 | 2.18679828 | | Up |
| NONMMUG036531.2 | NONMMUT058766.2 | | 1820 | | 31 | | 64 | 2.185710184 | | Up |
| NONMMUG035681.2 | NONMMUT057449.2 | | 681.06 | | 16 | | 33 | 2.182891043 | | Up |
| NONMMUG075299.1 | NONMMUT120086.1 | | 1247 | | 37 | | 76 | 2.1710511 | | Up |
| NONMMUG085140.1 | NONMMUT135022.1 | | 789 | | 10.41 | | 21.38 | 2.170686373 | | Up |
| NONMMUG069052.1 | NONMMUT110435.1 | | 1114 | | 13.22 | | 27.15 | 2.170562847 | | Up |
| NONMMUG010755.2 | NONMMUT017094.2 | | 4242.26 | | 9.73 | | 19.98 | 2.17019255 | | Up |
| NONMMUG023052.2 | NONMMUT037383.2 | | 944 | | 19 | | 39 | 2.169052215 | | Up |
| NONMMUG066842.1 | NONMMUT106955.1 | | 562 | | 10.77 | | 22.1 | 2.168159044 | | Up |
| NONMMUG067919.1 | NONMMUT108652.1 | | 2672 | | 20 | | 41 | 2.165350624 | | Up |
| NONMMUG011497.2 | NONMMUT018490.2 | | 3452 | | 82 | | 168 | 2.163633641 | | Up |
| NONMMUG005321.2 | NONMMUT008594.2 | | 1231 | | 21 | | 43 | 2.161997468 | | Up |
| NONMMUG077259.1 | NONMMUT123178.1 | | 466 | | 21 | | 43 | 2.161997468 | | Up |
| NONMMUG066331.1 | NONMMUT106129.1 | | 708 | | 17.07 | | 34.92 | 2.159283806 | | Up |
| NONMMUG067729.1 | NONMMUT108312.1 | | 2165 | | 10.43 | | 21.3 | 2.15433135 | | Up |
| NONMMUG031541.2 | NONMMUT050882.2 | | 831 | | 24 | | 49 | 2.153597491 | | Up |
| LXLOC_069743 | LTCONS_00122274 | | 1163 | | 10.3 | | 21.01 | 2.150966454 | | Up |
| LXLOC_059658 | LTCONS_00104629 | | 11576.4 | | 1150.08 | | 2344.41 | 2.149084159 | | Up |
| NONMMUG038883.2 | NONMMUT063001.2 | | 586.01 | | 56 | | 114 | 2.145172989 | | Up |
| NONMMUG019665.2 | NONMMUT031983.2 | | 1435 | | 129.97 | | 264 | 2.138821353 | | Up |
| NONMMUG014787.2 | NONMMUT023953.2 | | 4034 | | 60.85 | | 123.47 | 2.135765562 | | Up |
| NONMMUG072812.1 | NONMMUT116138.1 | | 3473 | | 17.39 | | 35.14 | 2.123815322 | | Up |
| NONMMUG034518.2 | NONMMUT055608.2 | | 1826 | | 51 | | 103 | 2.122253175 | | Up |
| NONMMUG041231.2 | NONMMUT066632.2 | | 255 | | 63 | | 127 | 2.116912331 | | Up |
| NONMMUG022107.2 | NONMMUT035935.2 | | 414 | | 22.34 | | 45 | 2.114694436 | | Up |
| NONMMUG042119.2 | NONMMUT068028.2 | | 659.3 | | 74 | | 149 | 2.113533114 | | Up |
| NONMMUG077939.1 | NONMMUT124313.1 | | 3549 | | 23.01 | | 46.07 | 2.097236033 | | Up |
| LXLOC_021739 | LTCONS_00037782 | | 2210 | | 48.9 | | 97.81 | 2.094397819 | | Up |
| NONMMUG066711.1 | NONMMUT106735.1 | | 1313 | | 11 | | 22 | 2.094102804 | | Up |
| NONMMUG077367.1 | NONMMUT123349.1 | | 1808 | | 11 | | 22 | 2.094102804 | | Up |
| NONMMUG027180.2 | NONMMUT044053.2 | | 743.98 | | 11 | | 22 | 2.094102804 | | Up |
| NONMMUG044162.2 | NONMMUT071294.2 | | 745 | | 12 | | 24 | 2.094102804 | | Up |
| NONMMUG056344.1 | NONMMUT090428.1 | | 355 | | 12 | | 24 | 2.094102804 | | Up |
| LXLOC_086542 | LTCONS_00150928 | | 348 | | 13 | | 26 | 2.094102804 | | Up |
| NONMMUG046655.1 | NONMMUT075227.1 | | 1470 | | 14 | | 28 | 2.094102804 | | Up |
| NONMMUG017193.2 | NONMMUT027742.2 | | 3521 | | 15 | | 30 | 2.094102804 | | Up |
| NONMMUG060522.1 | NONMMUT096837.1 | | 533 | | 15 | | 30 | 2.094102804 | | Up |
| NONMMUG034991.2 | NONMMUT056340.2 | | 2069 | | 15 | | 30 | 2.094102804 | | Up |
| NONMMUG086137.1 | NONMMUT136447.1 | | 1196 | | 15 | | 30 | 2.094102804 | | Up |
| NONMMUG071814.1 | NONMMUT114526.1 | | 673.64 | | 15 | | 30 | 2.094102804 | | Up |
| NONMMUG035566.2 | NONMMUT057278.2 | | 788.18 | | 16 | | 32 | 2.094102804 | | Up |
| NONMMUG026759.2 | NONMMUT043337.2 | | 2846 | | 17 | | 34 | 2.094102804 | | Up |
| NONMMUG010517.2 | NONMMUT016710.2 | | 3837 | | 19 | | 38 | 2.094102804 | | Up |
| NONMMUG045301.2 | NONMMUT073076.2 | | 3053 | | 19 | | 38 | 2.094102804 | | Up |
| NONMMUG006782.2 | NONMMUT010766.2 | | 351 | | 19 | | 38 | 2.094102804 | | Up |
| NONMMUG000946.2 | NONMMUT001395.2 | | 3029 | | 22 | | 44 | 2.094102804 | | Up |
| NONMMUG027315.2 | NONMMUT044256.2 | | 1602 | | 33 | | 66 | 2.094102804 | | Up |
| NONMMUG002080.2 | NONMMUT003207.2 | | 1140 | | 35 | | 70 | 2.094102804 | | Up |
| NONMMUG013306.2 | NONMMUT021432.2 | | 2337 | | 52 | | 104 | 2.094102804 | | Up |
| NONMMUG072769.1 | NONMMUT116071.1 | | 1110 | | 81.58 | | 162.05 | 2.074406021 | | Up |
| NONMMUG017463.2 | NONMMUT028201.2 | | 1821.13 | | 74.9 | | 148.74 | 2.073612911 | | Up |
| NONMMUG040121.2 | NONMMUT064962.2 | | 1180 | | 60 | | 119 | 2.06995714 | | Up |
| NONMMUG052609.1 | NONMMUT084474.1 | | 1323 | | 110.95 | | 220 | 2.069290517 | | Up |
| NONMMUG064201.1 | NONMMUT102696.1 | | 1360 | | 17.12 | | 33.9 | 2.065307948 | | Up |
| NONMMUG010026.2 | NONMMUT015957.2 | | 3364 | | 22.52 | | 44.57 | 2.063835072 | | Up |
| NONMMUG020844.2 | NONMMUT033925.2 | | 503 | | 11.62 | | 22.97 | 2.06038438 | | Up |
| NONMMUG000732.2 | NONMMUT001096.2 | | 948 | | 15.8 | | 31.05 | 2.043440225 | | Up |
| NONMMUG015252.2 | NONMMUT024650.2 | | 1773 | | 12.83 | | 25.2 | 2.041907931 | | Up |
| NONMMUG000727.2 | NONMMUT001087.2 | | 3587 | | 27 | | 53 | 2.040168709 | | Up |
| NONMMUG003136.2 | NONMMUT004893.2 | | 1131.92 | | 17.31 | | 33.97 | 2.039413791 | | Up |
| NONMMUG026669.2 | NONMMUT043182.2 | | 550 | | 25 | | 49 | 2.035810113 | | Up |
| NONMMUG058247.1 | NONMMUT093409.1 | | 1090 | | 24 | | 47 | 2.033355506 | | Up |
| NONMMUG079513.1 | NONMMUT126752.1 | | 1725 | | 22 | | 43.06 | 2.031792396 | | Up |
| NONMMUG019438.2 | NONMMUT031628.2 | | 1706 | | 23 | | 45 | 2.030685085 | | Up |
| NONMMUG035888.2 | NONMMUT057800.2 | | 914 | | 64 | | 125 | 2.025671374 | | Up |
| NONMMUG043757.2 | NONMMUT070687.2 | | 2457 | | 11.44 | | 22.33 | 2.023895203 | | Up |
| LXLOC_015245 | LTCONS_00026322 | | 1043 | | 88.63 | | 172.85 | 2.021409975 | | Up |
| NONMMUG031654.2 | NONMMUT051088.2 | | 1230 | | 20 | | 39 | 2.021051052 | | Up |
| LXLOC_043869 | LTCONS_00077003 | | 2475 | | 26.63 | | 51.92 | 2.020578714 | | Up |
| NONMMUG044338.2 | NONMMUT071550.2 | | 662 | | 78 | | 152 | 2.019153394 | | Up |
| NONMMUG038455.2 | NONMMUT062354.2 | | 1671 | | 19 | | 37 | 2.017154509 | | Up |
| NONMMUG031411.2 | NONMMUT050685.2 | | 859.62 | | 38 | | 74 | 2.017154509 | | Up |
| NONMMUG035809.2 | NONMMUT057665.2 | | 3757 | | 53 | | 103 | 2.01126295 | | Up |
| LXLOC_073277 | LTCONS_00128447 | | 1522 | | 48.61 | | 94.41 | 2.009475895 | | Up |
| NONMMUG067613.1 | NONMMUT108127.1 | | 4434 | | 38.79 | | 75.31 | 2.008416143 | | Up |
| NONMMUG033302.2 | NONMMUT053654.2 | | 3274 | | 17 | | 33 | 2.007965361 | | Up |
| NONMMUG049490.1 | NONMMUT079547.1 | | 828 | | 16 | | 31 | 2.002495425 | | Up |
| NONMMUG072437.1 | NONMMUT115498.1 | | 524 | | 16 | | 31 | 2.002495425 | | Up |
| NONMMUG003107.2 | NONMMUT004829.2 | | 238 | | 32 | | 62 | 2.002495425 | | Up |
| NONMMUG029420.2 | NONMMUT047510.2 | | 1807 | | 18.41 | | 35.66 | 2.001736957 | | Up |
| NONMMUG017797.2 | NONMMUT028776.2 | | 2277.84 | | 2785.05 | | 1344.65 | -2.00684225 | | Down |
| NONMMUG078500.1 | NONMMUT125236.1 | | 1514 | | 29 | | 14 | -2.007149342 | | Down |
| NONMMUG048808.1 | NONMMUT078562.1 | | 1042 | | 29 | | 14 | -2.007149342 | | Down |
| LXLOC_032797 | LTCONS_00057075 | | 970 | | 25.41 | | 12.26 | -2.008771988 | | Down |
| LXLOC_040480 | LTCONS_00070859 | | 1840 | | 195.78 | | 94.44 | -2.009424089 | | Down |
| NONMMUG075150.1 | NONMMUT119862.1 | | 734 | | 56 | | 27 | -2.010832035 | | Down |
| NONMMUG030132.2 | NONMMUT048556.2 | | 607.23 | | 59.75 | | 28.77 | -2.01464418 | | Down |
| NONMMUG039188.2 | NONMMUT063452.2 | | 900 | | 27 | | 13 | -2.014792764 | | Down |
| NONMMUG077021.1 | NONMMUT122778.1 | | 3676 | | 27 | | 13 | -2.014792764 | | Down |
| NONMMUG015089.2 | NONMMUT024409.2 | | 1319 | | 51.16 | | 24.62 | -2.0162682 | | Down |
| NONMMUG009065.2 | NONMMUT014316.2 | | 1148 | | 28.18 | | 13.56 | -2.016526062 | | Down |
| NONMMUG023242.2 | NONMMUT037652.2 | | 3889 | | 112.35 | | 54 | -2.01983482 | | Down |
| NONMMUG086442.1 | NONMMUT136843.1 | | 212 | | 25 | | 12 | -2.023684574 | | Down |
| NONMMUG076184.1 | NONMMUT121488.1 | | 698.55 | | 25 | | 12 | -2.023684574 | | Down |
| NONMMUG049537.1 | NONMMUT079648.1 | | 286 | | 25 | | 12 | -2.023684574 | | Down |
| NONMMUG029529.2 | NONMMUT047670.2 | | 338 | | 25 | | 12 | -2.023684574 | | Down |
| NONMMUG021984.2 | NONMMUT035754.2 | | 1761 | | 25.99 | | 12.44 | -2.031837492 | | Down |
| NONMMUG038830.2 | NONMMUT062929.2 | | 1278 | | 23 | | 11 | -2.03415787 | | Down |
| NONMMUG035324.2 | NONMMUT056906.2 | | 1672 | | 23 | | 11 | -2.03415787 | | Down |
| LXLOC_021791 | LTCONS_00037862 | | 4801 | | 24.14 | | 11.52 | -2.040467114 | | Down |
| NONMMUG012442.2 | NONMMUT020062.2 | | 1485 | | 108.38 | | 51.59 | -2.047767597 | | Down |
| NONMMUG083273.1 | NONMMUT132270.1 | | 5320 | | 39.42 | | 18.73 | -2.053050949 | | Down |
| NONMMUG074405.1 | NONMMUT118560.1 | | 745 | | 40 | | 19 | -2.053898358 | | Down |
| NONMMUG055577.1 | NONMMUT089245.1 | | 1153 | | 40 | | 19 | -2.053898358 | | Down |
| NONMMUG040941.2 | NONMMUT066155.2 | | 2135 | | 124.31 | | 59 | -2.056208191 | | Down |
| NONMMUG012576.2 | NONMMUT020260.2 | | 3612 | | 61.68 | | 29.27 | -2.056657235 | | Down |
| NONMMUG028254.2 | NONMMUT045766.2 | | 724 | | 27 | | 12.81 | -2.057275059 | | Down |
| NONMMUG018598.2 | NONMMUT030102.2 | | 262 | | 38 | | 18 | -2.06190222 | | Down |
| NONMMUG017422.2 | NONMMUT028126.2 | | 2291 | | 21.94 | | 10.39 | -2.062632939 | | Down |
| NONMMUG051282.1 | NONMMUT082382.1 | | 1261 | | 24.33 | | 11.51 | -2.065594169 | | Down |
| NONMMUG062134.1 | NONMMUT099311.1 | | 1712 | | 127 | | 60 | -2.069485378 | | Down |
| NONMMUG083293.1 | NONMMUT132301.1 | | 1366 | | 28.08 | | 13.25 | -2.072998347 | | Down |
| NONMMUG044004.2 | NONMMUT071061.2 | | 2123 | | 35.41 | | 16.66 | -2.081434082 | | Down |
| NONMMUG071156.1 | NONMMUT113521.1 | | 865 | | 53.63 | | 25.19 | -2.086275458 | | Down |
| NONMMUG042448.2 | NONMMUT068574.2 | | 2514 | | 37.22 | | 17.48 | -2.086642937 | | Down |
| NONMMUG038027.2 | NONMMUT061239.2 | | 491 | | 49 | | 23 | -2.088192972 | | Down |
| NONMMUG002343.2 | NONMMUT003649.2 | | 1598.45 | | 32 | | 15 | -2.092116004 | | Down |
| NONMMUG052318.1 | NONMMUT084026.1 | | 769 | | 47 | | 22 | -2.096211662 | | Down |
| NONMMUG046440.2 | NONMMUT074875.2 | | 1397 | | 24.31 | | 11.37 | -2.098532474 | | Down |
| NONMMUG005108.2 | NONMMUT008274.2 | | 2605 | | 26.91 | | 12.52 | -2.113712853 | | Down |
| LXLOC_025877 | LTCONS_00045021 | | 2388 | | 21.27 | | 9.89 | -2.11545241 | | Down |
| NONMMUG041733.2 | NONMMUT067391.2 | | 540 | | 28 | | 13 | -2.119727603 | | Down |
| NONMMUG068826.1 | NONMMUT110030.1 | | 394 | | 33.42 | | 15.51 | -2.12092329 | | Down |
| NONMMUG064042.1 | NONMMUT102356.1 | | 1359 | | 26.45 | | 12.25 | -2.126869142 | | Down |
| NONMMUG056306.1 | NONMMUT090384.1 | | 1039.03 | | 35.07 | | 16.21 | -2.132603875 | | Down |
| NONMMUG037681.2 | NONMMUT060624.2 | | 279 | | 26 | | 12 | -2.13685163 | | Down |
| NONMMUG007428.2 | NONMMUT011737.2 | | 1958.91 | | 26 | | 12 | -2.13685163 | | Down |
| NONMMUG078369.1 | NONMMUT125038.1 | | 1570 | | 36.07 | | 16.64 | -2.138185175 | | Down |
| LXLOC_086425 | LTCONS_00150698 | | 2345 | | 37.94 | | 17.49 | -2.140275975 | | Down |
| NONMMUG084330.1 | NONMMUT133850.1 | | 1441 | | 49.96 | | 22.88 | -2.159270045 | | Down |
| NONMMUG058916.1 | NONMMUT094373.1 | | 938 | | 178.25 | | 81.52 | -2.163249436 | | Down |
| LXLOC_041116 | LTCONS_00072060 | | 1995 | | 44.45 | | 20.28 | -2.170148729 | | Down |
| NONMMUG039185.2 | NONMMUT063449.2 | | 1595 | | 57 | | 26 | -2.170797788 | | Down |
| LXLOC_072172 | LTCONS_00126558 | | 24126 | | 108.41 | | 49.44 | -2.17139158 | | Down |
| NONMMUG006736.2 | NONMMUT010701.2 | | 2730 | | 83.25 | | 37.96 | -2.171837755 | | Down |
| NONMMUG032169.2 | NONMMUT051872.2 | | 2655 | | 35.14 | | 16.01 | -2.174178963 | | Down |
| NONMMUG084142.1 | NONMMUT133549.1 | | 1041 | | 30.36 | | 13.82 | -2.176725546 | | Down |
| NONMMUG060034.1 | NONMMUT096009.1 | | 559 | | 26.48 | | 12.05 | -2.177637147 | | Down |
| LXLOC_031996 | LTCONS_00055536 | | 26489 | | 38.43 | | 17.47 | -2.180603932 | | Down |
| NONMMUG059812.1 | NONMMUT095673.1 | | 471 | | 22 | | 10 | -2.180904243 | | Down |
| NONMMUG047995.1 | NONMMUT077357.1 | | 482 | | 22 | | 10 | -2.180904243 | | Down |
| NONMMUG041816.2 | NONMMUT067502.2 | | 1231.03 | | 22 | | 10 | -2.180904243 | | Down |
| NONMMUG052721.1 | NONMMUT084645.1 | | 680.15 | | 29.25 | | 13.29 | -2.182088236 | | Down |
| NONMMUG015284.2 | NONMMUT024696.2 | | 1761.25 | | 128.44 | | 58.23 | -2.188417288 | | Down |
| NONMMUG052497.1 | NONMMUT084291.1 | | 1384 | | 49.1 | | 22.26 | -2.188436059 | | Down |
| NONMMUG005412.2 | NONMMUT008747.2 | | 2267 | | 25.5 | | 11.56 | -2.188608894 | | Down |
| NONMMUG044072.2 | NONMMUT071168.2 | | 1316.69 | | 64 | | 29 | -2.189935205 | | Down |
| NONMMUG062420.1 | NONMMUT099776.1 | | 1194 | | 43.77 | | 19.83 | -2.190412608 | | Down |
| NONMMUG024119.2 | NONMMUT039051.2 | | 579 | | 31 | | 14 | -2.199579972 | | Down |
| NONMMUG006918.2 | NONMMUT010961.2 | | 1042 | | 133.37 | | 60.15 | -2.203492282 | | Down |
| NONMMUG070945.1 | NONMMUT113172.1 | | 1345 | | 20 | | 9 | -2.209903382 | | Down |
| NONMMUG023217.2 | NONMMUT037622.2 | | 778 | | 20 | | 9 | -2.209903382 | | Down |
| NONMMUG053060.1 | NONMMUT085186.1 | | 2195 | | 60 | | 27 | -2.209903382 | | Down |
| NONMMUG001254.2 | NONMMUT001871.2 | | 2158 | | 41.63 | | 18.72 | -2.211983443 | | Down |
| NONMMUG017437.2 | NONMMUT028155.2 | | 1922.07 | | 38 | | 17 | -2.22682654 | | Down |
| NONMMUG017289.2 | NONMMUT027899.2 | | 627.86 | | 109.98 | | 49.05 | -2.235729496 | | Down |
| NONMMUG066187.1 | NONMMUT105885.1 | | 807 | | 51.13 | | 22.78 | -2.238701753 | | Down |
| NONMMUG064051.1 | NONMMUT102368.1 | | 1507 | | 27 | | 12 | -2.245747198 | | Down |
| NONMMUG063694.1 | NONMMUT101782.1 | | 381 | | 27 | | 12 | -2.245747198 | | Down |
| NONMMUG043854.2 | NONMMUT070819.2 | | 2980 | | 61 | | 27 | -2.257596866 | | Down |
| NONMMUG071393.1 | NONMMUT113882.1 | | 897 | | 43 | | 19 | -2.262571678 | | Down |
| LXLOC_040193 | LTCONS_00070378 | | 1916 | | 215.16 | | 94.92 | -2.267148972 | | Down |
| NONMMUG037685.2 | NONMMUT060630.2 | | 1322 | | 50 | | 22 | -2.274746338 | | Down |
| NONMMUG063327.1 | NONMMUT101216.1 | | 813 | | 19.64 | | 8.62 | -2.281967506 | | Down |
| NONMMUG079199.1 | NONMMUT126212.1 | | 1213 | | 65.02 | | 28.5 | -2.285740469 | | Down |
| LXLOC_000626 | LTCONS_00001076 | | 1042 | | 37.83 | | 16.56 | -2.289553403 | | Down |
| NONMMUG054930.1 | NONMMUT088177.1 | | 678 | | 32 | | 14 | -2.291187351 | | Down |
| LXLOC_000800 | LTCONS_00001378 | | 2161 | | 99.83 | | 43.63 | -2.294203101 | | Down |
| NONMMUG081146.1 | NONMMUT129238.1 | | 1910.08 | | 28.9 | | 12.63 | -2.294326903 | | Down |
| NONMMUG026517.2 | NONMMUT042948.2 | | 1505 | | 80.18 | | 35.01 | -2.296847907 | | Down |
| NONMMUG081630.1 | NONMMUT129921.1 | | 732 | | 39 | | 17 | -2.301775951 | | Down |
| NONMMUG007379.2 | NONMMUT011656.2 | | 1717 | | 28.17 | | 12.27 | -2.303945825 | | Down |
| LXLOC_073274 | LTCONS_00128439 | | 3525 | | 74.12 | | 32.27 | -2.305235803 | | Down |
| NONMMUG013958.2 | NONMMUT022616.2 | | 921 | | 23 | | 10 | -2.309164918 | | Down |
| NONMMUG047193.1 | NONMMUT076085.1 | | 637 | | 23 | | 10 | -2.309164918 | | Down |
| NONMMUG079818.1 | NONMMUT127167.1 | | 779 | | 32.85 | | 14.28 | -2.309691978 | | Down |
| NONMMUG086075.1 | NONMMUT136372.1 | | 2668 | | 27.85 | | 12.06 | -2.320792036 | | Down |
| NONMMUG077282.1 | NONMMUT123214.1 | | 888 | | 18.65 | | 8.04 | -2.333713643 | | Down |
| NONMMUG053635.1 | NONMMUT086188.1 | | 1632 | | 25.33 | | 10.9 | -2.33893508 | | Down |
| NONMMUG033103.2 | NONMMUT053306.2 | | 1834 | | 20.98 | | 9.02 | -2.341527874 | | Down |
| NONMMUG008157.2 | NONMMUT012842.2 | | 2552 | | 28 | | 12 | -2.350682038 | | Down |
| NONMMUG084161.1 | NONMMUT133584.1 | | 464 | | 28 | | 12 | -2.350682038 | | Down |
| NONMMUG016864.2 | NONMMUT027177.2 | | 513 | | 18.65 | | 7.99 | -2.35171364 | | Down |
| NONMMUG022417.2 | NONMMUT036443.2 | | 2943.3 | | 35 | | 14.98 | -2.354531792 | | Down |
| NONMMUG061047.1 | NONMMUT097669.1 | | 2196 | | 63.15 | | 27 | -2.357543849 | | Down |
| NONMMUG020184.2 | NONMMUT032816.2 | | 4523 | | 32.93 | | 14.05 | -2.363561959 | | Down |
| NONMMUG019047.2 | NONMMUT030884.2 | | 762 | | 21.18 | | 9 | -2.375308561 | | Down |
| NONMMUG060651.1 | NONMMUT097045.1 | | 1103 | | 31.79 | | 13.49 | -2.379262532 | | Down |
| NONMMUG050911.1 | NONMMUT081788.1 | | 1302.02 | | 33 | | 14 | -2.37997559 | | Down |
| NONMMUG028739.2 | NONMMUT046519.2 | | 2403 | | 36.74 | | 15.58 | -2.381209982 | | Down |
| NONMMUG008090.2 | NONMMUT012743.2 | | 1402.24 | | 236.54 | | 100.19 | -2.384588497 | | Down |
| NONMMUG086494.1 | NONMMUT136908.1 | | 1363 | | 18.61 | | 7.88 | -2.385518237 | | Down |
| NONMMUG041094.2 | NONMMUT066390.2 | | 722 | | 26 | | 11 | -2.387913395 | | Down |
| NONMMUG002138.2 | NONMMUT003290.2 | | 1559 | | 18.32 | | 7.75 | -2.388199771 | | Down |
| NONMMUG052877.1 | NONMMUT084890.1 | | 1394 | | 54.9 | | 23.19 | -2.392503854 | | Down |
| NONMMUG078037.1 | NONMMUT124473.1 | | 1217 | | 19 | | 8.01 | -2.398147737 | | Down |
| NONMMUG005524.2 | NONMMUT008905.2 | | 1411 | | 33.41 | | 14.03 | -2.409427143 | | Down |
| NONMMUG064639.1 | NONMMUT103388.1 | | 666 | | 31 | | 13 | -2.41341038 | | Down |
| NONMMUG075798.1 | NONMMUT120847.1 | | 1379 | | 31 | | 13 | -2.41341038 | | Down |
| NONMMUG009690.2 | NONMMUT015244.2 | | 1250 | | 21.48 | | 9 | -2.415891369 | | Down |
| NONMMUG023789.2 | NONMMUT038488.2 | | 3790 | | 111.71 | | 46.6 | -2.428610152 | | Down |
| NONMMUG017648.2 | NONMMUT028509.2 | | 1868 | | 38.28 | | 15.96 | -2.430157552 | | Down |
| LXLOC_021335 | LTCONS_00037060 | | 1985 | | 34.45 | | 14.36 | -2.430793663 | | Down |
| NONMMUG071285.1 | NONMMUT113731.1 | | 543 | | 24 | | 10 | -2.431966007 | | Down |
| NONMMUG079506.1 | NONMMUT126744.1 | | 1431 | | 19.36 | | 8.02 | -2.448706817 | | Down |
| NONMMUG058138.1 | NONMMUT093252.1 | | 495 | | 29 | | 12 | -2.451934184 | | Down |
| NONMMUG075264.1 | NONMMUT120023.1 | | 3833 | | 29 | | 12 | -2.451934184 | | Down |
| NONMMUG024953.2 | NONMMUT040280.2 | | 325 | | 29 | | 12 | -2.451934184 | | Down |
| NONMMUG034880.2 | NONMMUT056143.2 | | 777 | | 58 | | 24 | -2.451934184 | | Down |
| NONMMUG010836.2 | NONMMUT017254.2 | | 4435 | | 25.1 | | 10.37 | -2.456440136 | | Down |
| NONMMUG018790.2 | NONMMUT030385.2 | | 822 | | 46 | | 19 | -2.457166081 | | Down |
| NONMMUG044076.2 | NONMMUT071174.2 | | 1993 | | 46 | | 19 | -2.457166081 | | Down |
| NONMMUG071918.1 | NONMMUT114710.1 | | 444 | | 22 | | 9.08 | -2.459375838 | | Down |
| NONMMUG013379.2 | NONMMUT021555.2 | | 2483 | | 21.84 | | 9 | -2.463849095 | | Down |
| NONMMUG019877.2 | NONMMUT032297.2 | | 2193 | | 17 | | 7 | -2.466113034 | | Down |
| NONMMUG012245.2 | NONMMUT019711.2 | | 1934 | | 17 | | 7 | -2.466113034 | | Down |
| NONMMUG071003.1 | NONMMUT113286.1 | | 1203 | | 17 | | 7 | -2.466113034 | | Down |
| NONMMUG042262.2 | NONMMUT068321.2 | | 934.26 | | 17 | | 7 | -2.466113034 | | Down |
| NONMMUG081408.1 | NONMMUT129572.1 | | 362 | | 17 | | 7 | -2.466113034 | | Down |
| NONMMUG004130.2 | NONMMUT006415.2 | | 2777 | | 51 | | 21 | -2.466113034 | | Down |
| NONMMUG033787.2 | NONMMUT054478.2 | | 1189.83 | | 225.81 | | 92.81 | -2.47141162 | | Down |
| NONMMUG024964.2 | NONMMUT040299.2 | | 3724 | | 207.72 | | 85.18 | -2.478004187 | | Down |
| NONMMUG074007.1 | NONMMUT117950.1 | | 1094 | | 26.67 | | 10.93 | -2.479746043 | | Down |
| NONMMUG011205.2 | NONMMUT017901.2 | | 3064 | | 19.01 | | 7.79 | -2.480023793 | | Down |
| NONMMUG012508.2 | NONMMUT020166.2 | | 576.95 | | 92 | | 37.69 | -2.480801328 | | Down |
| NONMMUG078732.1 | NONMMUT125615.1 | | 4708 | | 21.97 | | 9 | -2.480973122 | | Down |
| NONMMUG032824.2 | NONMMUT052860.2 | | 462 | | 22 | | 9 | -2.48491043 | | Down |
| NONMMUG006221.2 | NONMMUT009922.2 | | 503 | | 22 | | 9 | -2.48491043 | | Down |
| NONMMUG048115.1 | NONMMUT077524.1 | | 631 | | 27.58 | | 11.28 | -2.485607974 | | Down |
| LXLOC_032587 | LTCONS_00056662 | | 3003 | | 17.93 | | 7.33 | -2.486877965 | | Down |
| NONMMUG074777.1 | NONMMUT119191.1 | | 1308 | | 34.11 | | 13.9 | -2.49611694 | | Down |
| NONMMUG044200.2 | NONMMUT071354.2 | | 569 | | 54 | | 22 | -2.496808963 | | Down |
| NONMMUG022622.2 | NONMMUT108949.1 | | 3789 | | 34.94 | | 14.22 | -2.499813482 | | Down |
| NONMMUG074021.1 | NONMMUT117967.1 | | 1083 | | 33.34 | | 13.55 | -2.503819701 | | Down |
| NONMMUG063178.1 | NONMMUT100992.1 | | 1322 | | 55.03 | | 22.36 | -2.504493477 | | Down |
| NONMMUG000266.2 | NONMMUT000374.2 | | 1159 | | 69 | | 28 | -2.508236265 | | Down |
| NONMMUG061347.1 | NONMMUT098149.1 | | 325 | | 37 | | 15 | -2.511022736 | | Down |
| NONMMUG004826.2 | NONMMUT007615.2 | | 5818 | | 37 | | 15 | -2.511022736 | | Down |
| NONMMUG059300.1 | NONMMUT094933.1 | | 3392 | | 20.22 | | 8.12 | -2.538359925 | | Down |
| NONMMUG033919.2 | NONMMUT121459.1 | | 4903 | | 20 | | 8.02 | -2.542548912 | | Down |
| NONMMUG044222.2 | NONMMUT071381.2 | | 2176 | | 17.08 | | 6.84 | -2.546376685 | | Down |
| NONMMUG016656.2 | NONMMUT026862.2 | | 410 | | 25 | | 10 | -2.549753385 | | Down |
| NONMMUG074281.1 | NONMMUT118357.1 | | 221 | | 25 | | 10 | -2.549753385 | | Down |
| NONMMUG044070.2 | NONMMUT071163.2 | | 2706.14 | | 60 | | 24 | -2.549753385 | | Down |
| NONMMUG046839.1 | NONMMUT075524.1 | | 716 | | 65.21 | | 25.88 | -2.5724084 | | Down |
| LXLOC_063136 | LTCONS_00110387 | | 1677 | | 22.94 | | 9.09 | -2.576923579 | | Down |
| NONMMUG067739.1 | NONMMUT108340.1 | | 3257 | | 22.57 | | 8.94 | -2.57801656 | | Down |
| NONMMUG066093.1 | NONMMUT105740.1 | | 1793 | | 38 | | 15 | -2.587971031 | | Down |
| NONMMUG078558.1 | NONMMUT125338.1 | | 3243 | | 20.3 | | 8 | -2.59271284 | | Down |
| NONMMUG057730.1 | NONMMUT092627.1 | | 433 | | 28 | | 11 | -2.601743802 | | Down |
| NONMMUG036669.2 | NONMMUT059016.2 | | 1829 | | 28 | | 11 | -2.601743802 | | Down |
| NONMMUG035830.2 | NONMMUT057689.2 | | 2550 | | 34.56 | | 13.57 | -2.603262189 | | Down |
| NONMMUG021417.2 | NONMMUT034799.2 | | 1993 | | 23 | | 9 | -2.613171105 | | Down |
| NONMMUG064360.1 | NONMMUT102953.1 | | 1050 | | 23 | | 9 | -2.613171105 | | Down |
| LXLOC_032182 | LTCONS_00055908 | | 3344 | | 87 | | 34 | -2.616858505 | | Down |
| NONMMUG004163.2 | NONMMUT006466.2 | | 252 | | 35.24 | | 13.73 | -2.625661793 | | Down |
| NONMMUG083859.1 | NONMMUT133163.1 | | 4115 | | 23.99 | | 9.34 | -2.627774601 | | Down |
| NONMMUG023756.2 | NONMMUT038442.2 | | 1559 | | 18 | | 7 | -2.631037354 | | Down |
| NONMMUG058091.1 | NONMMUT093175.1 | | 302 | | 36 | | 14 | -2.631037354 | | Down |
| NONMMUG038064.2 | NONMMUT061298.2 | | 1070 | | 21.91 | | 8.52 | -2.631225493 | | Down |
| NONMMUG041881.2 | NONMMUT067599.2 | | 284 | | 25.44 | | 9.89 | -2.632009685 | | Down |
| LXLOC_055022 | LTCONS_00096414 | | 2649.63 | | 236.8 | | 92.01 | -2.633510213 | | Down |
| NONMMUG045506.2 | NONMMUT073369.2 | | 1123 | | 14.98 | | 5.81 | -2.638752306 | | Down |
| NONMMUG041846.2 | NONMMUT067542.2 | | 829 | | 49 | | 19 | -2.639461857 | | Down |
| NONMMUG007448.2 | NONMMUT011764.2 | | 3542 | | 37.74 | | 14.61 | -2.644173685 | | Down |
| NONMMUG000551.2 | NONMMUT000812.2 | | 2219 | | 44 | | 17 | -2.64983475 | | Down |
| NONMMUG008397.2 | NONMMUT013233.2 | | 1491 | | 17.16 | | 6.59 | -2.667295561 | | Down |
| NONMMUG052407.1 | NONMMUT084145.1 | | 820 | | 15.95 | | 6.1 | -2.679247748 | | Down |
| LXLOC_050339 | LTCONS_00088335 | | 917 | | 44.42 | | 16.93 | -2.689152035 | | Down |
| NONMMUG046611.1 | NONMMUT075170.1 | | 214 | | 21 | | 8 | -2.690532041 | | Down |
| NONMMUG018834.2 | NONMMUT030448.2 | | 1787 | | 31.55 | | 12.01 | -2.692704904 | | Down |
| LXLOC_064966 | LTCONS_00113798 | | 1213 | | 72.47 | | 27.33 | -2.719694064 | | Down |
| NONMMUG040902.2 | NONMMUT066088.2 | | 2296 | | 24.5 | | 9.21 | -2.728914571 | | Down |
| NONMMUG053834.1 | NONMMUT086459.1 | | 1860 | | 34.89 | | 13.11 | -2.730189019 | | Down |
| NONMMUG068980.1 | NONMMUT110344.1 | | 604 | | 18.44 | | 6.92 | -2.733886621 | | Down |
| NONMMUG051742.1 | NONMMUT083130.1 | | 307 | | 16 | | 6 | -2.735972194 | | Down |
| NONMMUG069684.1 | NONMMUT111345.1 | | 735 | | 16 | | 6 | -2.735972194 | | Down |
| LXLOC_089228 | LTCONS_00155400 | | 3573 | | 159.59 | | 59.43 | -2.756111132 | | Down |
| NONMMUG047001.1 | NONMMUT075767.1 | | 1075 | | 108.9 | | 40.53 | -2.757780943 | | Down |
| NONMMUG016157.2 | NONMMUT026105.2 | | 2431.22 | | 51.5 | | 19.15 | -2.760353276 | | Down |
| NONMMUG070159.1 | NONMMUT112044.1 | | 405 | | 89 | | 33 | -2.768575819 | | Down |
| NONMMUG083420.1 | NONMMUT132510.1 | | 788 | | 46 | | 17 | -2.778095425 | | Down |
| NONMMUG022819.2 | NONMMUT037057.2 | | 353 | | 19 | | 7 | -2.787042378 | | Down |
| NONMMUG063512.1 | NONMMUT101497.1 | | 746 | | 38 | | 14 | -2.787042378 | | Down |
| NONMMUG026870.2 | NONMMUT043494.2 | | 3436 | | 112 | | 41.1 | -2.798474062 | | Down |
| NONMMUG033997.2 | NONMMUT054795.2 | | 1431 | | 30 | | 11 | -2.800815149 | | Down |
| NONMMUG044811.2 | NONMMUT072286.2 | | 2797 | | 22.41 | | 8.21 | -2.803274239 | | Down |
| NONMMUG053504.1 | NONMMUT085960.1 | | 1579 | | 18.52 | | 6.78 | -2.805351036 | | Down |
| NONMMUG026425.2 | NONMMUT042666.2 | | 321 | | 22 | | 8 | -2.824760433 | | Down |
| NONMMUG001338.2 | NONMMUT002001.2 | | 1036 | | 55 | | 20 | -2.824760433 | | Down |
| LXLOC_088813 | LTCONS_00154725 | | 22728 | | 32.37 | | 11.76 | -2.827435806 | | Down |
| NONMMUG077476.1 | NONMMUT123533.1 | | 1431 | | 15.83 | | 5.75 | -2.827951984 | | Down |
| LXLOC_007181 | LTCONS_00012153 | | 14138 | | 22.23 | | 8.05 | -2.836791716 | | Down |
| NONMMUG032240.2 | NONMMUT051981.2 | | 560.88 | | 38.84 | | 14 | -2.850129943 | | Down |
| NONMMUG078775.1 | NONMMUT125662.1 | | 1425 | | 23.45 | | 8.44 | -2.854443233 | | Down |
| LXLOC_054930 | LTCONS_00096249 | | 674 | | 14 | | 5.01 | -2.870985833 | | Down |
| NONMMUG053384.1 | NONMMUT085749.1 | | 271 | | 14 | | 5 | -2.87675085 | | Down |
| NONMMUG084102.1 | NONMMUT133478.1 | | 764 | | 14 | | 5 | -2.87675085 | | Down |
| NONMMUG079193.1 | NONMMUT126206.1 | | 898 | | 14 | | 5 | -2.87675085 | | Down |
| NONMMUG044255.2 | NONMMUT071424.2 | | 741 | | 14 | | 5 | -2.87675085 | | Down |
| NONMMUG030046.2 | NONMMUT048429.2 | | 241 | | 14 | | 5 | -2.87675085 | | Down |
| NONMMUG063225.1 | NONMMUT101068.1 | | 1095 | | 14 | | 5 | -2.87675085 | | Down |
| NONMMUG083401.1 | NONMMUT132466.1 | | 812 | | 28 | | 10 | -2.87675085 | | Down |
| NONMMUG016024.2 | NONMMUT025877.2 | | 997 | | 27.61 | | 9.86 | -2.876959868 | | Down |
| NONMMUG079182.1 | NONMMUT126186.1 | | 1536 | | 24.75 | | 8.82 | -2.883053124 | | Down |
| NONMMUG072983.1 | NONMMUT116370.1 | | 2672 | | 35.64 | | 12.7 | -2.883234875 | | Down |
| NONMMUG046370.2 | NONMMUT074720.2 | | 2201.62 | | 2258.4 | | 803.76 | -2.886825889 | | Down |
| NONMMUG023006.2 | NONMMUT037309.2 | | 1131 | | 54.52 | | 19.33 | -2.897785045 | | Down |
| NONMMUG016479.2 | NONMMUT026601.2 | | 1668.04 | | 17.01 | | 6.03 | -2.898203663 | | Down |
| NONMMUG027267.2 | NONMMUT044190.2 | | 360 | | 17 | | 6 | -2.910897877 | | Down |
| NONMMUG010350.2 | NONMMUT016470.2 | | 724.06 | | 17 | | 6 | -2.910897877 | | Down |
| NONMMUG063680.1 | NONMMUT101761.1 | | 1846 | | 17 | | 6 | -2.910897877 | | Down |
| NONMMUG040009.2 | NONMMUT064786.2 | | 240 | | 17 | | 6 | -2.910897877 | | Down |
| NONMMUG016739.2 | NONMMUT026999.2 | | 1726 | | 17.85 | | 6.3 | -2.910897877 | | Down |
| NONMMUG040457.2 | NONMMUT065426.2 | | 430 | | 34 | | 12 | -2.910897877 | | Down |
| NONMMUG000911.2 | NONMMUT001344.2 | | 6063 | | 13.76 | | 4.85 | -2.914744831 | | Down |
| NONMMUG063879.1 | NONMMUT102054.1 | | 1334 | | 14.52 | | 5.1 | -2.924841798 | | Down |
| NONMMUG008188.2 | NONMMUT012902.2 | | 2969 | | 20 | | 7 | -2.935043541 | | Down |
| LXLOC_015135 | LTCONS_00026132 | | 2874 | | 79.68 | | 27.77 | -2.947278136 | | Down |
| NONMMUG086891.1 | NONMMUT137443.1 | | 1437 | | 28.79 | | 10.02 | -2.951267756 | | Down |
| NONMMUG056662.1 | NONMMUT090913.1 | | 1893 | | 28.14 | | 9.79 | -2.952380323 | | Down |
| LXLOC_056234 | LTCONS_00098634 | | 6374 | | 278.96 | | 97.02 | -2.953305565 | | Down |
| NONMMUG052968.1 | NONMMUT085033.1 | | 952 | | 22.09 | | 7.65 | -2.965620727 | | Down |
| NONMMUG052600.1 | NONMMUT084460.1 | | 853 | | 27.59 | | 9.5 | -2.982189272 | | Down |
| NONMMUG009985.2 | NONMMUT015892.2 | | 2143 | | 96 | | 33 | -2.987033958 | | Down |
| NONMMUG069768.1 | NONMMUT111459.1 | | 625 | | 39.55 | | 13.4 | -3.028786584 | | Down |
| NONMMUG049276.1 | NONMMUT079201.1 | | 887 | | 39.73 | | 13.43 | -3.035436173 | | Down |
| NONMMUG024331.2 | NONMMUT039370.2 | | 3072 | | 35.72 | | 12.04 | -3.043690572 | | Down |
| LXLOC_075188 | LTCONS_00131652 | | 1281 | | 32.1 | | 10.73 | -3.067743638 | | Down |
| NONMMUG039109.2 | NONMMUT063323.2 | | 2658 | | 19.07 | | 6.37 | -3.069776328 | | Down |
| NONMMUG042609.2 | NONMMUT068845.2 | | 492 | | 12 | | 4 | -3.075822197 | | Down |
| NONMMUG019095.2 | NONMMUT030998.2 | | 2208.49 | | 12 | | 4 | -3.075822197 | | Down |
| NONMMUG019194.2 | NONMMUT031165.2 | | 3527 | | 15 | | 5 | -3.075822197 | | Down |
| NONMMUG037787.2 | NONMMUT060802.2 | | 279 | | 24 | | 8 | -3.075822197 | | Down |
| NONMMUG070157.1 | NONMMUT112042.1 | | 929 | | 24 | | 8 | -3.075822197 | | Down |
| NONMMUG036923.2 | NONMMUT059413.2 | | 465 | | 24 | | 8 | -3.075822197 | | Down |
| NONMMUG047323.1 | NONMMUT076286.1 | | 1924 | | 27 | | 9 | -3.075822197 | | Down |
| NONMMUG067198.1 | NONMMUT107489.1 | | 700 | | 30 | | 10 | -3.075822197 | | Down |
| NONMMUG016882.2 | NONMMUT098835.1 | | 4283 | | 12.31 | | 4.1 | -3.078167089 | | Down |
| NONMMUG051456.1 | NONMMUT082666.1 | | 3470 | | 29 | | 9.64 | -3.083792893 | | Down |
| NONMMUG057022.1 | NONMMUT091545.1 | | 1447 | | 15.63 | | 5.19 | -3.086919865 | | Down |
| NONMMUG018929.2 | NONMMUT030700.2 | | 1365 | | 15.25 | | 5.06 | -3.089097101 | | Down |
| NONMMUG071145.1 | NONMMUT113503.1 | | 264 | | 34.42 | | 11.36 | -3.104465721 | | Down |
| LXLOC_050972 | LTCONS_00089432 | | 8982.38 | | 16.76 | | 5.49 | -3.126185385 | | Down |
| NONMMUG016702.2 | NONMMUT026930.2 | | 3414 | | 18.4 | | 6 | -3.139239916 | | Down |
| NONMMUG010325.2 | NONMMUT016429.2 | | 612 | | 12.29 | | 4 | -3.144723217 | | Down |
| NONMMUG070339.1 | NONMMUT112327.1 | | 2869 | | 14.81 | | 4.82 | -3.144830374 | | Down |
| NONMMUG050666.1 | NONMMUT081439.1 | | 1570 | | 17.95 | | 5.81 | -3.160644746 | | Down |
| NONMMUG042982.2 | NONMMUT069387.2 | | 277 | | 31 | | 10 | -3.170433627 | | Down |
| NONMMUG062315.1 | NONMMUT099608.1 | | 816 | | 19.47 | | 6.28 | -3.170730035 | | Down |
| NONMMUG030627.2 | NONMMUT049468.2 | | 2424 | | 12.41 | | 4 | -3.172759616 | | Down |
| NONMMUG084240.1 | NONMMUT133730.1 | | 428 | | 28 | | 9 | -3.180757037 | | Down |
| NONMMUG012333.2 | NONMMUT019873.2 | | 3399 | | 30.93 | | 9.91 | -3.189996937 | | Down |
| LXLOC_008984 | LTCONS_00015507 | | 4910 | | 25 | | 8 | -3.193609575 | | Down |
| NONMMUG038509.2 | NONMMUT062430.2 | | 1364 | | 22 | | 7 | -3.210050589 | | Down |
| LXLOC_038071 | LTCONS_00066312 | | 1441 | | 12.58 | | 4 | -3.21201723 | | Down |
| NONMMUG076034.1 | NONMMUT121221.1 | | 346 | | 41 | | 13 | -3.220121768 | | Down |
| NONMMUG059878.1 | NONMMUT095766.1 | | 898 | | 49.41 | | 15.65 | -3.223177993 | | Down |
| NONMMUG074926.1 | NONMMUT119474.1 | | 607 | | 19 | | 6 | -3.231827221 | | Down |
| NONMMUG021154.2 | NONMMUT034400.2 | | 2227 | | 16 | | 5 | -3.262041006 | | Down |
| LXLOC_061623 | LTCONS_00107803 | | 32599.47 | | 26.56 | | 8.25 | -3.27947544 | | Down |
| NONMMUG004045.2 | NONMMUT006290.2 | | 2857 | | 21.44 | | 6.65 | -3.283654516 | | Down |
| NONMMUG075127.1 | NONMMUT119799.1 | | 1263 | | 42 | | 13 | -3.289652605 | | Down |
| NONMMUG013983.2 | NONMMUT022648.2 | | 1646 | | 23.19 | | 7.15 | -3.300872541 | | Down |
| NONMMUG014601.2 | NONMMUT023595.2 | | 2399 | | 13 | | 4 | -3.306776632 | | Down |
| NONMMUG033993.2 | NONMMUT054791.2 | | 1849 | | 13 | | 4 | -3.306776632 | | Down |
| NONMMUG066047.1 | NONMMUT105639.1 | | 637 | | 13 | | 4 | -3.306776632 | | Down |
| NONMMUG010584.2 | NONMMUT016827.2 | | 409 | | 13 | | 4 | -3.306776632 | | Down |
| NONMMUG063934.1 | NONMMUT102141.1 | | 778 | | 13 | | 4 | -3.306776632 | | Down |
| NONMMUG051828.1 | NONMMUT083258.1 | | 1275 | | 13 | | 4 | -3.306776632 | | Down |
| NONMMUG072442.1 | NONMMUT115503.1 | | 515 | | 10.78 | | 3.25 | -3.365588305 | | Down |
| NONMMUG006692.2 | NONMMUT010634.2 | | 557 | | 20 | | 6 | -3.379828384 | | Down |
| NONMMUG083480.1 | NONMMUT132597.1 | | 1323 | | 20 | | 6 | -3.379828384 | | Down |
| NONMMUG017117.2 | NONMMUT027630.2 | | 572 | | 20 | | 6 | -3.379828384 | | Down |
| NONMMUG060970.1 | NONMMUT097508.1 | | 1525 | | 13.4 | | 4.01 | -3.387014913 | | Down |
| NONMMUG044650.2 | NONMMUT072060.2 | | 2589 | | 13.27 | | 3.97 | -3.387812112 | | Down |
| NONMMUG010752.2 | NONMMUT017091.2 | | 4085 | | 15 | | 4.45 | -3.412067715 | | Down |
| NONMMUG081966.1 | NONMMUT130411.1 | | 3164 | | 14 | | 4.15 | -3.414384367 | | Down |
| LXLOC_020286 | LTCONS_00035287 | | 3142 | | 75.7 | | 22.39 | -3.42077474 | | Down |
| NONMMUG008775.2 | NONMMUT013880.2 | | 460 | | 20.66 | | 6.11 | -3.421089134 | | Down |
| NONMMUG086394.1 | NONMMUT136772.1 | | 1807 | | 23.58 | | 6.93 | -3.439170117 | | Down |
| NONMMUG059222.1 | NONMMUT094844.1 | | 1072 | | 10.4 | | 3.05 | -3.445301957 | | Down |
| NONMMUG074563.1 | NONMMUT118837.1 | | 524 | | 28.26 | | 8.28 | -3.448014781 | | Down |
| NONMMUG018686.2 | NONMMUT030231.2 | | 1108.26 | | 13.53 | | 3.95 | -3.458371757 | | Down |
| NONMMUG058245.1 | NONMMUT093407.1 | | 376 | | 24 | | 7 | -3.461112353 | | Down |
| NONMMUG024427.2 | NONMMUT039505.2 | | 3916 | | 10.63 | | 3.09 | -3.470822903 | | Down |
| NONMMUG064250.1 | NONMMUT102775.1 | | 1781 | | 10.77 | | 3.11 | -3.489960724 | | Down |
| NONMMUG009473.2 | NONMMUT014940.2 | | 3203 | | 14 | | 4 | -3.52060704 | | Down |
| NONMMUG064677.1 | NONMMUT103449.1 | | 956 | | 14 | | 4 | -3.52060704 | | Down |
| NONMMUG005549.2 | NONMMUT008945.2 | | 320 | | 14 | | 4 | -3.52060704 | | Down |
| LXLOC_024452 | LTCONS_00042614 | | 5325 | | 23.95 | | 6.79 | -3.542981548 | | Down |
| NONMMUG005486.2 | NONMMUT008848.2 | | 3844 | | 11.84 | | 3.34 | -3.557395342 | | Down |
| NONMMUG060893.1 | NONMMUT097388.1 | | 1598 | | 15.43 | | 4.34 | -3.565839424 | | Down |
| LXLOC_089136 | LTCONS_00155264 | | 402 | | 32 | | 9 | -3.566047193 | | Down |
| NONMMUG029372.2 | NONMMUT047441.2 | | 2781 | | 31.69 | | 8.91 | -3.566957841 | | Down |
| NONMMUG065736.1 | NONMMUT105112.1 | | 4119 | | 15.19 | | 4.27 | -3.567524985 | | Down |
| NONMMUG047543.1 | NONMMUT076641.1 | | 1216 | | 10.3 | | 2.87 | -3.592940586 | | Down |
| NONMMUG061672.1 | NONMMUT098628.1 | | 4200 | | 10.15 | | 2.81 | -3.611572579 | | Down |
| NONMMUG002882.2 | NONMMUT004460.2 | | 2947 | | 11 | | 3.04 | -3.616617785 | | Down |
| NONMMUG012520.2 | NONMMUT020184.2 | | 1317 | | 29 | | 8 | -3.621859186 | | Down |
| NONMMUG080018.1 | NONMMUT127473.1 | | 1880 | | 17.5 | | 4.78 | -3.650441993 | | Down |
| NONMMUG031635.2 | NONMMUT051065.2 | | 890 | | 11 | | 3 | -3.654835431 | | Down |
| NONMMUG082877.1 | NONMMUT131692.1 | | 512 | | 11 | | 3 | -3.654835431 | | Down |
| NONMMUG032466.2 | NONMMUT052292.2 | | 242 | | 11 | | 3 | -3.654835431 | | Down |
| NONMMUG033544.2 | NONMMUT054102.2 | | 1420 | | 11 | | 3 | -3.654835431 | | Down |
| NONMMUG009980.2 | NONMMUT015881.2 | | 1362 | | 11 | | 3 | -3.654835431 | | Down |
| NONMMUG077501.1 | NONMMUT123575.1 | | 303 | | 11 | | 3 | -3.654835431 | | Down |
| NONMMUG053315.1 | NONMMUT085612.1 | | 436 | | 11 | | 3 | -3.654835431 | | Down |
| NONMMUG073746.1 | NONMMUT117574.1 | | 5676 | | 11.01 | | 3 | -3.657457322 | | Down |
| NONMMUG078304.1 | NONMMUT124926.1 | | 1102 | | 31.58 | | 8.58 | -3.665820432 | | Down |
| NONMMUG015169.2 | NONMMUT024530.2 | | 444 | | 11.12 | | 3.01 | -3.676539987 | | Down |
| NONMMUG046010.2 | NONMMUT074181.2 | | 454 | | 12.86 | | 3.45 | -3.702341947 | | Down |
| NONMMUG019800.2 | NONMMUT032166.2 | | 2432 | | 16.16 | | 4.33 | -3.705873732 | | Down |
| NONMMUG052956.1 | NONMMUT085009.1 | | 232 | | 15 | | 4 | -3.719678387 | | Down |
| NONMMUG050210.1 | NONMMUT080696.1 | | 298 | | 15 | | 4 | -3.719678387 | | Down |
| NONMMUG067132.1 | NONMMUT107383.1 | | 235 | | 15 | | 4 | -3.719678387 | | Down |
| NONMMUG039010.2 | NONMMUT063186.2 | | 4159 | | 21.94 | | 5.83 | -3.72988867 | | Down |
| NONMMUG074217.1 | NONMMUT118246.1 | | 792.71 | | 34 | | 9 | -3.740972875 | | Down |
| LXLOC_082739 | LTCONS_00144399 | | 3586.23 | | 21.84 | | 5.77 | -3.74655646 | | Down |
| NONMMUG001963.2 | NONMMUT003030.2 | | 581 | | 15.75 | | 4.15 | -3.75423437 | | Down |
| NONMMUG008419.2 | NONMMUT013269.2 | | 270 | | 19 | | 5 | -3.757896033 | | Down |
| NONMMUG074017.1 | NONMMUT117960.1 | | 1001 | | 16.19 | | 4.26 | -3.758252496 | | Down |
| NONMMUG034098.2 | NONMMUT054952.2 | | 561 | | 9 | | 2.36 | -3.768173479 | | Down |
| NONMMUG033366.2 | NONMMUT053785.2 | | 2644 | | 47 | | 12.3 | -3.773902078 | | Down |
| NONMMUG082747.1 | NONMMUT131458.1 | | 846 | | 15.44 | | 4.04 | -3.774388305 | | Down |
| NONMMUG014305.2 | NONMMUT023135.2 | | 1908 | | 14.43 | | 3.77 | -3.778766938 | | Down |
| NONMMUG059653.1 | NONMMUT095423.1 | | 4244 | | 10.3 | | 2.68 | -3.790576059 | | Down |
| NONMMUG008710.2 | NONMMUT013783.2 | | 1109 | | 35 | | 9 | -3.824613227 | | Down |
| NONMMUG018260.2 | NONMMUT029539.2 | | 1889 | | 23.02 | | 5.89 | -3.838993784 | | Down |
| LXLOC_068908 | LTCONS_00120820 | | 3489 | | 12.67 | | 3.24 | -3.840598808 | | Down |
| NONMMUG053750.1 | NONMMUT086348.1 | | 325 | | 52.64 | | 13.38 | -3.858059942 | | Down |
| LXLOC_047973 | LTCONS_00084271 | | 1209 | | 30.81 | | 7.81 | -3.865905653 | | Down |
| NONMMUG063372.1 | NONMMUT101285.1 | | 2073 | | 12.7 | | 3.2 | -3.883266569 | | Down |
| LXLOC_015616 | LTCONS_00026988 | | 2743 | | 178.88 | | 44.97 | -3.889811804 | | Down |
| NONMMUG020410.2 | NONMMUT033179.2 | | 406 | | 12 | | 3 | -3.905897196 | | Down |
| NONMMUG054659.1 | NONMMUT087772.1 | | 871 | | 12 | | 3 | -3.905897196 | | Down |
| NONMMUG071964.1 | NONMMUT114782.1 | | 404 | | 12 | | 3 | -3.905897196 | | Down |
| NONMMUG051701.1 | NONMMUT083068.1 | | 505 | | 12 | | 3 | -3.905897196 | | Down |
| NONMMUG084163.1 | NONMMUT133586.1 | | 458 | | 12 | | 3 | -3.905897196 | | Down |
| NONMMUG056464.1 | NONMMUT090598.1 | | 314 | | 12 | | 3 | -3.905897196 | | Down |
| NONMMUG035706.2 | NONMMUT057484.2 | | 2563 | | 12 | | 3 | -3.905897196 | | Down |
| NONMMUG013649.2 | NONMMUT021974.2 | | 1487 | | 20 | | 5 | -3.905897196 | | Down |
| NONMMUG064665.1 | NONMMUT103429.1 | | 425 | | 28 | | 7 | -3.905897196 | | Down |
| NONMMUG044522.2 | NONMMUT071856.2 | | 2346 | | 12.15 | | 3.02 | -3.922568914 | | Down |
| NONMMUG017111.2 | NONMMUT027624.2 | | 3387 | | 12.14 | | 3 | -3.939365227 | | Down |
| NONMMUG042491.2 | NONMMUT134095.1 | | 733.86 | | 12.4 | | 3 | -4.000508625 | | Down |
| LXLOC_013349 | LTCONS_00023057 | | 1062 | | 31.17 | | 7.52 | -4.008604371 | | Down |
| NONMMUG031597.2 | NONMMUT050983.2 | | 2638 | | 12.49 | | 3 | -4.021375338 | | Down |
| LXLOC_044738 | LTCONS_00078621 | | 1759 | | 14.77 | | 3.54 | -4.027594317 | | Down |
| NONMMUG002663.2 | NONMMUT004109.2 | | 1621 | | 8.7 | | 2.08 | -4.034760941 | | Down |
| NONMMUG044321.2 | NONMMUT071529.2 | | 388 | | 62.04 | | 14.81 | -4.039151287 | | Down |
| NONMMUG056389.1 | NONMMUT090493.1 | | 1152 | | 21.69 | | 5.17 | -4.04348493 | | Down |
| NONMMUG016043.2 | NONMMUT025915.2 | | 1605 | | 9.81 | | 2.33 | -4.053743559 | | Down |
| NONMMUG014751.2 | NONMMUT023878.2 | | 1093 | | 16.14 | | 3.82 | -4.063889266 | | Down |
| NONMMUG008616.2 | NONMMUT013587.2 | | 229 | | 18.01 | | 4.26 | -4.065642887 | | Down |
| NONMMUG063927.1 | NONMMUT102131.1 | | 388 | | 17 | | 4 | -4.080822878 | | Down |
| LXLOC_078176 | LTCONS_00137024 | | 854 | | 14.42 | | 3.39 | -4.083325168 | | Down |
| NONMMUG073579.1 | NONMMUT117302.1 | | 2023 | | 142.38 | | 33.09 | -4.116455815 | | Down |
| NONMMUG015042.2 | NONMMUT024340.2 | | 2588 | | 16.39 | | 3.8 | -4.123386257 | | Down |
| NONMMUG044075.2 | NONMMUT071173.2 | | 297 | | 13 | | 3 | -4.13685163 | | Down |
| NONMMUG034341.2 | NONMMUT055305.2 | | 1477 | | 13 | | 3 | -4.13685163 | | Down |
| NONMMUG039625.2 | NONMMUT064190.2 | | 562 | | 13 | | 3 | -4.13685163 | | Down |
| LXLOC_089062 | LTCONS_00155126 | | 2035 | | 40.69 | | 9.13 | -4.217872226 | | Down |
| NONMMUG008384.2 | NONMMUT013217.2 | | 2530 | | 8.82 | | 1.97 | -4.231063248 | | Down |
| NONMMUG016935.2 | NONMMUT027277.2 | | 7949 | | 8.99 | | 2 | -4.242539427 | | Down |
| NONMMUG072530.1 | NONMMUT115667.1 | | 1337 | | 9 | | 2 | -4.245747198 | | Down |
| NONMMUG059919.1 | NONMMUT095821.1 | | 826 | | 9 | | 2 | -4.245747198 | | Down |
| NONMMUG029823.2 | NONMMUT048073.2 | | 606 | | 9 | | 2 | -4.245747198 | | Down |
| NONMMUG003734.2 | NONMMUT005784.2 | | 1082 | | 9 | | 2 | -4.245747198 | | Down |
| NONMMUG071055.1 | NONMMUT113366.1 | | 1333 | | 9 | | 2 | -4.245747198 | | Down |
| NONMMUG069715.1 | NONMMUT111400.1 | | 566 | | 9 | | 2 | -4.245747198 | | Down |
| NONMMUG076104.1 | NONMMUT121351.1 | | 231 | | 9 | | 2 | -4.245747198 | | Down |
| NONMMUG039689.2 | NONMMUT064321.2 | | 1402 | | 9 | | 2 | -4.245747198 | | Down |
| NONMMUG036391.2 | NONMMUT058543.2 | | 1970 | | 18 | | 4 | -4.245747198 | | Down |
| NONMMUG012171.2 | NONMMUT019600.2 | | 2575 | | 9.08 | | 2 | -4.271281791 | | Down |
| NONMMUG040990.2 | NONMMUT066238.2 | | 1228 | | 9.6 | | 2.11 | -4.277480009 | | Down |
| NONMMUG013907.2 | NONMMUT022548.2 | | 1344 | | 14.43 | | 3.16 | -4.288046868 | | Down |
| NONMMUG038112.2 | NONMMUT061374.2 | | 1157 | | 9.7 | | 2.11 | -4.307380692 | | Down |
| NONMMUG041385.2 | NONMMUT066860.2 | | 451 | | 13 | | 2.81 | -4.325636371 | | Down |
| NONMMUG023383.2 | NONMMUT037863.2 | | 1419 | | 13.02 | | 2.81 | -4.330072021 | | Down |
| LXLOC_070476 | LTCONS_00123552 | | 1909 | | 51.03 | | 10.99 | -4.336205706 | | Down |
| NONMMUG011009.2 | NONMMUT017566.2 | | 1075 | | 14 | | 3 | -4.350682038 | | Down |
| NONMMUG008523.2 | NONMMUT013433.2 | | 3540 | | 14 | | 3 | -4.350682038 | | Down |
| NONMMUG069683.1 | NONMMUT111344.1 | | 2230 | | 14 | | 3 | -4.350682038 | | Down |
| NONMMUG033568.2 | NONMMUT054135.2 | | 1809 | | 14 | | 3 | -4.350682038 | | Down |
| NONMMUG030344.2 | NONMMUT048864.2 | | 2295 | | 8.23 | | 1.76 | -4.356531199 | | Down |
| NONMMUG028944.2 | NONMMUT046810.2 | | 2575 | | 10.97 | | 2.33 | -4.376220527 | | Down |
| LXLOC_009807 | LTCONS_00016889 | | 519 | | 9.44 | | 2 | -4.383470915 | | Down |
| NONMMUG042615.2 | NONMMUT068858.2 | | 1446 | | 20.05 | | 4.24 | -4.388829329 | | Down |
| NONMMUG012853.2 | NONMMUT020662.2 | | 2338 | | 8.02 | | 1.69 | -4.399055176 | | Down |
| NONMMUG027061.2 | NONMMUT043780.2 | | 2040 | | 22.01 | | 4.59 | -4.429083368 | | Down |
| NONMMUG083489.1 | NONMMUT132612.1 | | 2799 | | 12 | | 2.5 | -4.431966007 | | Down |
| NONMMUG037468.2 | NONMMUT060286.2 | | 1244 | | 16.67 | | 3.44 | -4.459444464 | | Down |
| NONMMUG029802.2 | NONMMUT048034.2 | | 3616 | | 8.72 | | 1.76 | -4.523402608 | | Down |
| NONMMUG062792.1 | NONMMUT100382.1 | | 3690 | | 9.98 | | 2 | -4.543976827 | | Down |
| LXLOC_093669 | LTCONS_00162385 | | 2449 | | 10 | | 2 | -4.549753385 | | Down |
| NONMMUG006445.2 | NONMMUT010260.2 | | 1419 | | 10 | | 2 | -4.549753385 | | Down |
| NONMMUG007250.2 | NONMMUT011473.2 | | 478 | | 10 | | 2 | -4.549753385 | | Down |
| NONMMUG056542.1 | NONMMUT090752.1 | | 1099 | | 10 | | 2 | -4.549753385 | | Down |
| NONMMUG070937.1 | NONMMUT113155.1 | | 373 | | 10 | | 2 | -4.549753385 | | Down |
| NONMMUG024776.2 | NONMMUT040034.2 | | 936 | | 10 | | 2 | -4.549753385 | | Down |
| NONMMUG043537.2 | NONMMUT070351.2 | | 275 | | 10 | | 2 | -4.549753385 | | Down |
| NONMMUG027468.2 | NONMMUT044513.2 | | 446 | | 15 | | 3 | -4.549753385 | | Down |
| LXLOC_071143 | LTCONS_00124774 | | 1009 | | 14.89 | | 2.97 | -4.557515031 | | Down |
| NONMMUG043764.2 | NONMMUT070696.2 | | 1597 | | 10.57 | | 2.1 | -4.568925483 | | Down |
| NONMMUG053092.1 | NONMMUT085255.1 | | 1141 | | 9.13 | | 1.77 | -4.639628195 | | Down |
| NONMMUG050216.1 | NONMMUT080704.1 | | 2192 | | 10.29 | | 1.99 | -4.646702488 | | Down |
| NONMMUG029099.2 | NONMMUT047066.2 | | 2353 | | 14.87 | | 2.85 | -4.672638842 | | Down |
| NONMMUG008148.2 | NONMMUT012830.2 | | 2571 | | 8.23 | | 1.57 | -4.686152939 | | Down |
| NONMMUG085331.1 | NONMMUT135330.1 | | 499 | | 17.38 | | 3.28 | -4.71721792 | | Down |
| NONMMUG027486.2 | NONMMUT044535.2 | | 2236 | | 8.17 | | 1.51 | -4.777472253 | | Down |
| NONMMUG050092.1 | NONMMUT080514.1 | | 1069 | | 7.74 | | 1.41 | -4.819174003 | | Down |
| NONMMUG052376.1 | NONMMUT084097.1 | | 1375 | | 11 | | 2 | -4.824760433 | | Down |
| NONMMUG053292.1 | NONMMUT085569.1 | | 1279 | | 11 | | 2 | -4.824760433 | | Down |
| NONMMUG011267.2 | NONMMUT018011.2 | | 1342 | | 11 | | 2 | -4.824760433 | | Down |
| NONMMUG085705.1 | NONMMUT135899.1 | | 1134 | | 11.58 | | 2.05 | -4.901776073 | | Down |
| LXLOC_092644 | LTCONS_00160728 | | 1741 | | 9.61 | | 1.64 | -5.007578428 | | Down |
| LXLOC_057250 | LTCONS_00100389 | | 2693 | | 16.71 | | 2.83 | -5.029572746 | | Down |
| NONMMUG018383.2 | NONMMUT029760.2 | | 792 | | 39.82 | | 6.69 | -5.052727406 | | Down |
| NONMMUG064161.1 | NONMMUT102583.1 | | 310 | | 12 | | 2 | -5.075822197 | | Down |
| NONMMUG024614.2 | NONMMUT039778.2 | | 1435 | | 12 | | 2 | -5.075822197 | | Down |
| NONMMUG085773.1 | NONMMUT135979.1 | | 956 | | 12.63 | | 2.1 | -5.082684008 | | Down |
| NONMMUG048706.1 | NONMMUT078420.1 | | 3738 | | 6.05 | | 1 | -5.09976748 | | Down |
| NONMMUG033575.2 | NONMMUT054142.2 | | 1319 | | 7.2 | | 1.19 | -5.099967862 | | Down |
| NONMMUG013931.2 | NONMMUT022575.2 | | 2009 | | 6.58 | | 1.07 | -5.14685077 | | Down |
| NONMMUG047555.1 | NONMMUT076660.1 | | 631 | | 6.1 | | 0 | -5.15251482 | | Down |
| LXLOC_082592 | LTCONS_00144154 | | 2049 | | 13.1 | | 2.05 | -5.257639189 | | Down |
| NONMMUG048382.1 | NONMMUT077954.1 | | 1081 | | 6.4 | | 1 | -5.262041006 | | Down |
| NONMMUG043078.2 | NONMMUT069553.2 | | 596 | | 13 | | 2 | -5.306776632 | | Down |
| NONMMUG017315.2 | NONMMUT027940.2 | | 1871 | | 10.63 | | 1.63 | -5.31629265 | | Down |
| NONMMUG052076.1 | NONMMUT083624.1 | | 1891 | | 28.69 | | 4.39 | -5.322407442 | | Down |
| NONMMUG023348.2 | NONMMUT037807.2 | | 3085 | | 7.02 | | 1.07 | -5.333617663 | | Down |
| NONMMUG058945.1 | NONMMUT094408.1 | | 751 | | 6.71 | | 1.01 | -5.369812142 | | Down |
| NONMMUG000354.2 | NONMMUT000508.2 | | 538 | | 40.13 | | 6.02 | -5.379588724 | | Down |
| NONMMUG070976.1 | NONMMUT113235.1 | | 1204 | | 6.76 | | 1.01 | -5.391233103 | | Down |
| NONMMUG040005.2 | NONMMUT064781.2 | | 3948 | | 7.55 | | 1.12 | -5.41185302 | | Down |
| NONMMUG053572.1 | NONMMUT086095.1 | | 5490 | | 6.83 | | 1 | -5.449668353 | | Down |
| NONMMUG014747.2 | NONMMUT023873.2 | | 936 | | 6.84 | | 1 | -5.453889846 | | Down |
| NONMMUG086576.1 | NONMMUT137029.1 | | 669 | | 7.03 | | 1.01 | -5.504235988 | | Down |
| NONMMUG025246.2 | NONMMUT040719.2 | | 4215 | | 7 | | 1 | -5.52060704 | | Down |
| NONMMUG062077.1 | NONMMUT099210.1 | | 1165 | | 7 | | 1 | -5.52060704 | | Down |
| NONMMUG032321.2 | NONMMUT052101.2 | | 858 | | 7 | | 1 | -5.52060704 | | Down |
| NONMMUG073744.1 | NONMMUT117569.1 | | 2805.29 | | 7 | | 1 | -5.52060704 | | Down |
| NONMMUG063919.1 | NONMMUT102110.1 | | 1017 | | 7 | | 1 | -5.52060704 | | Down |
| NONMMUG076837.1 | NONMMUT122498.1 | | 714 | | 7 | | 1 | -5.52060704 | | Down |
| NONMMUG008102.2 | NONMMUT012759.2 | | 1789 | | 7 | | 1 | -5.52060704 | | Down |
| NONMMUG046825.1 | NONMMUT075500.1 | | 2755 | | 7 | | 1 | -5.52060704 | | Down |
| NONMMUG024937.2 | NONMMUT040258.2 | | 2943 | | 7 | | 1 | -5.52060704 | | Down |
| NONMMUG083688.1 | NONMMUT132945.1 | | 266 | | 7 | | 1 | -5.52060704 | | Down |
| NONMMUG007260.2 | NONMMUT011490.2 | | 2420 | | 7 | | 1 | -5.52060704 | | Down |
| NONMMUG052884.1 | NONMMUT084903.1 | | 751 | | 7 | | 1 | -5.52060704 | | Down |
| NONMMUG012297.2 | NONMMUT019785.2 | | 2691 | | 7 | | 1 | -5.52060704 | | Down |
| NONMMUG052734.1 | NONMMUT084666.1 | | 1962 | | 7 | | 1 | -5.52060704 | | Down |
| NONMMUG046844.1 | NONMMUT075532.1 | | 1099 | | 7 | | 1 | -5.52060704 | | Down |
| NONMMUG039067.2 | NONMMUT063260.2 | | 3778 | | 7 | | 1 | -5.52060704 | | Down |
| NONMMUG043057.2 | NONMMUT069515.2 | | 931 | | 7 | | 1 | -5.52060704 | | Down |
| NONMMUG039325.2 | NONMMUT063672.2 | | 2340 | | 7 | | 1 | -5.52060704 | | Down |
| NONMMUG014461.2 | NONMMUT023374.2 | | 2231 | | 7 | | 1 | -5.52060704 | | Down |
| NONMMUG005783.2 | NONMMUT009296.2 | | 3865 | | 7 | | 1 | -5.52060704 | | Down |
| NONMMUG003406.2 | NONMMUT005281.2 | | 1592 | | 7 | | 1 | -5.52060704 | | Down |
| NONMMUG020083.2 | NONMMUT032603.2 | | 1357 | | 7 | | 1 | -5.52060704 | | Down |
| NONMMUG085352.1 | NONMMUT135364.1 | | 705 | | 7 | | 1 | -5.52060704 | | Down |
| NONMMUG061381.1 | NONMMUT098197.1 | | 2275 | | 7 | | 1 | -5.52060704 | | Down |
| NONMMUG077772.1 | NONMMUT124066.1 | | 1255 | | 7 | | 1 | -5.52060704 | | Down |
| NONMMUG036337.2 | NONMMUT058469.2 | | 3420 | | 7 | | 1 | -5.52060704 | | Down |
| NONMMUG085702.1 | NONMMUT135896.1 | | 316 | | 7 | | 1 | -5.52060704 | | Down |
| LXLOC_045412 | LTCONS_00079848 | | 802 | | 25.02 | | 3.53 | -5.556580597 | | Down |
| NONMMUG000755.2 | NONMMUT001131.2 | | 1378 | | 7.11 | | 1 | -5.565596315 | | Down |
| NONMMUG014104.2 | NONMMUT022817.2 | | 4168 | | 6.97 | | 0 | -5.596101203 | | Down |
| NONMMUG045376.2 | NONMMUT073174.2 | | 1517 | | 9 | | 1.25 | -5.601891009 | | Down |
| NONMMUG037546.2 | NONMMUT060399.2 | | 1697 | | 8.34 | | 1.15 | -5.622724241 | | Down |
| NONMMUG083315.1 | NONMMUT132329.1 | | 469 | | 22 | | 3 | -5.654835431 | | Down |
| NONMMUG081216.1 | NONMMUT129351.1 | | 1255 | | 7.35 | | 1 | -5.661385695 | | Down |
| NONMMUG079316.1 | NONMMUT126427.1 | | 587 | | 15 | | 2 | -5.719678387 | | Down |
| NONMMUG004341.2 | NONMMUT006743.2 | | 2869 | | 15 | | 2 | -5.719678387 | | Down |
| NONMMUG005029.2 | NONMMUT008128.2 | | 1515 | | 7.55 | | 1 | -5.738850484 | | Down |
| NONMMUG037258.2 | NONMMUT059981.2 | | 1386 | | 7.64 | | 1 | -5.773042472 | | Down |
| NONMMUG030735.2 | NONMMUT049634.2 | | 1747 | | 7.67 | | 1 | -5.784350351 | | Down |
| LXLOC_008569 | LTCONS_00014870 | | 1558 | | 34.01 | | 4.42 | -5.793578658 | | Down |
| NONMMUG031400.2 | NONMMUT050668.2 | | 866 | | 5.87 | | 0 | -5.804475555 | | Down |
| NONMMUG012116.2 | NONMMUT019520.2 | | 1671 | | 67.56 | | 8.72 | -5.813435772 | | Down |
| NONMMUG063479.1 | NONMMUT101437.1 | | 341 | | 8 | | 1 | -5.905897196 | | Down |
| NONMMUG061131.1 | NONMMUT097822.1 | | 940 | | 8 | | 1 | -5.905897196 | | Down |
| NONMMUG005934.2 | NONMMUT009496.2 | | 882 | | 8 | | 1 | -5.905897196 | | Down |
| NONMMUG083943.1 | NONMMUT133252.1 | | 3579 | | 8 | | 1 | -5.905897196 | | Down |
| NONMMUG005971.2 | NONMMUT009545.2 | | 701 | | 8 | | 1 | -5.905897196 | | Down |
| NONMMUG012669.2 | NONMMUT020404.2 | | 4386 | | 8 | | 1 | -5.905897196 | | Down |
| NONMMUG036511.2 | NONMMUT058728.2 | | 551 | | 8 | | 1 | -5.905897196 | | Down |
| NONMMUG075817.1 | NONMMUT120889.1 | | 799 | | 8 | | 1 | -5.905897196 | | Down |
| NONMMUG022710.2 | NONMMUT036894.2 | | 365 | | 8 | | 1 | -5.905897196 | | Down |
| NONMMUG056913.1 | NONMMUT091341.1 | | 666 | | 8 | | 1 | -5.905897196 | | Down |
| NONMMUG016442.2 | NONMMUT026554.2 | | 1678 | | 8 | | 1 | -5.905897196 | | Down |
| NONMMUG005064.2 | NONMMUT008183.2 | | 1736 | | 8 | | 1 | -5.905897196 | | Down |
| NONMMUG070226.1 | NONMMUT112155.1 | | 460 | | 8 | | 1 | -5.905897196 | | Down |
| NONMMUG059288.1 | NONMMUT094921.1 | | 2942 | | 8 | | 1 | -5.905897196 | | Down |
| NONMMUG047761.1 | NONMMUT076986.1 | | 378 | | 8 | | 1 | -5.905897196 | | Down |
| NONMMUG004856.2 | NONMMUT007669.2 | | 1084 | | 8 | | 1 | -5.905897196 | | Down |
| NONMMUG075560.1 | NONMMUT120473.1 | | 5321 | | 8 | | 1 | -5.905897196 | | Down |
| NONMMUG015287.2 | NONMMUT024702.2 | | 345 | | 16 | | 2 | -5.905897196 | | Down |
| LXLOC_073449 | LTCONS_00128748 | | 2944 | | 18.41 | | 2.3 | -5.907464916 | | Down |
| NONMMUG086024.1 | NONMMUT136305.1 | | 1930 | | 8.36 | | 1.04 | -5.919736023 | | Down |
| NONMMUG015667.2 | NONMMUT025310.2 | | 4865 | | 8.53 | | 1.01 | -6.062278093 | | Down |
| NONMMUG003145.2 | NONMMUT004905.2 | | 1441 | | 6.28 | | 0 | -6.115489576 | | Down |
| NONMMUG074783.1 | NONMMUT119204.1 | | 335 | | 9 | | 1 | -6.245747198 | | Down |
| NONMMUG084555.1 | NONMMUT134153.1 | | 999 | | 9 | | 1 | -6.245747198 | | Down |
| NONMMUG011437.2 | NONMMUT018377.2 | | 548 | | 9 | | 1 | -6.245747198 | | Down |
| NONMMUG069340.1 | NONMMUT110827.1 | | 685 | | 9 | | 1 | -6.245747198 | | Down |
| NONMMUG045925.2 | NONMMUT074067.2 | | 324 | | 9 | | 1 | -6.245747198 | | Down |
| NONMMUG033806.2 | NONMMUT054502.2 | | 249 | | 9.05 | | 1 | -6.26173278 | | Down |
| NONMMUG044089.2 | NONMMUT071192.2 | | 2239 | | 5.64 | | 0 | -6.27660728 | | Down |
| NONMMUG059933.1 | NONMMUT095841.1 | | 3967 | | 9.1 | | 1 | -6.277630286 | | Down |
| NONMMUG018650.2 | NONMMUT030181.2 | | 300 | | 23.12 | | 2.49 | -6.335744696 | | Down |
| NONMMUG077997.1 | NONMMUT124398.1 | | 2409 | | 7.48 | | 0 | -6.392124619 | | Down |
| NONMMUG068193.1 | NONMMUT109052.1 | | 976 | | 8.92 | | 0 | -6.398519292 | | Down |
| NONMMUG016749.2 | NONMMUT027011.2 | | 447 | | 49 | | 5 | -6.491460694 | | Down |
| NONMMUG012637.2 | NONMMUT020346.2 | | 989 | | 44.29 | | 4.45 | -6.536104708 | | Down |
| LXLOC_051973 | LTCONS_00091229 | | 884 | | 5 | | 0 | -6.549753385 | | Down |
| LXLOC_084827 | LTCONS_00148039 | | 4686 | | 5 | | 0 | -6.549753385 | | Down |
| NONMMUG012668.2 | NONMMUT020402.2 | | 3964 | | 5 | | 0 | -6.549753385 | | Down |
| NONMMUG001101.2 | NONMMUT001644.2 | | 3283 | | 5 | | 0 | -6.549753385 | | Down |
| NONMMUG062067.1 | NONMMUT099198.1 | | 2207 | | 5 | | 0 | -6.549753385 | | Down |
| NONMMUG039992.2 | NONMMUT064759.2 | | 3315 | | 5 | | 0 | -6.549753385 | | Down |
| NONMMUG035671.2 | NONMMUT057439.2 | | 420 | | 5 | | 0 | -6.549753385 | | Down |
| NONMMUG061135.1 | NONMMUT097826.1 | | 6733 | | 5 | | 0 | -6.549753385 | | Down |
| NONMMUG069237.1 | NONMMUT110676.1 | | 1182 | | 5 | | 0 | -6.549753385 | | Down |
| NONMMUG059345.1 | NONMMUT095012.1 | | 5631 | | 5 | | 0 | -6.549753385 | | Down |
| NONMMUG003894.2 | NONMMUT006051.2 | | 2378 | | 5 | | 0 | -6.549753385 | | Down |
| NONMMUG005588.2 | NONMMUT009013.2 | | 501 | | 5 | | 0 | -6.549753385 | | Down |
| NONMMUG047656.1 | NONMMUT076818.1 | | 2627 | | 5 | | 0 | -6.549753385 | | Down |
| NONMMUG044226.2 | NONMMUT071389.2 | | 1271 | | 5 | | 0 | -6.549753385 | | Down |
| NONMMUG064451.1 | NONMMUT103079.1 | | 2718 | | 5 | | 0 | -6.549753385 | | Down |
| NONMMUG015967.2 | NONMMUT025780.2 | | 1957.63 | | 5 | | 0 | -6.549753385 | | Down |
| NONMMUG058534.1 | NONMMUT093821.1 | | 913 | | 5 | | 0 | -6.549753385 | | Down |
| NONMMUG001715.2 | NONMMUT002536.2 | | 1177 | | 5 | | 0 | -6.549753385 | | Down |
| NONMMUG076302.1 | NONMMUT121692.1 | | 1608 | | 5 | | 0 | -6.549753385 | | Down |
| NONMMUG004613.2 | NONMMUT007232.2 | | 1513 | | 5 | | 0 | -6.549753385 | | Down |
| NONMMUG012583.2 | NONMMUT020270.2 | | 2639 | | 5 | | 0 | -6.549753385 | | Down |
| NONMMUG005892.2 | NONMMUT009444.2 | | 3894 | | 5 | | 0 | -6.549753385 | | Down |
| NONMMUG007781.2 | NONMMUT012295.2 | | 1225 | | 5 | | 0 | -6.549753385 | | Down |
| NONMMUG023247.2 | NONMMUT037661.2 | | 284 | | 5 | | 0 | -6.549753385 | | Down |
| NONMMUG057600.1 | NONMMUT092425.1 | | 1190 | | 5 | | 0 | -6.549753385 | | Down |
| NONMMUG067668.1 | NONMMUT108202.1 | | 1440 | | 5 | | 0 | -6.549753385 | | Down |
| NONMMUG083508.1 | NONMMUT132641.1 | | 472 | | 5 | | 0 | -6.549753385 | | Down |
| NONMMUG061067.1 | NONMMUT097710.1 | | 220 | | 5 | | 0 | -6.549753385 | | Down |
| NONMMUG056967.1 | NONMMUT091435.1 | | 736.66 | | 5 | | 0 | -6.549753385 | | Down |
| NONMMUG038465.2 | NONMMUT062369.2 | | 522 | | 5 | | 0 | -6.549753385 | | Down |
| NONMMUG083397.1 | NONMMUT132460.1 | | 648 | | 5 | | 0 | -6.549753385 | | Down |
| NONMMUG042695.2 | NONMMUT068974.2 | | 706 | | 5 | | 0 | -6.549753385 | | Down |
| NONMMUG081073.1 | NONMMUT129135.1 | | 719 | | 5 | | 0 | -6.549753385 | | Down |
| NONMMUG079751.1 | NONMMUT127089.1 | | 1223 | | 5 | | 0 | -6.549753385 | | Down |
| NONMMUG039686.2 | NONMMUT064314.2 | | 3920 | | 5 | | 0 | -6.549753385 | | Down |
| NONMMUG059849.1 | NONMMUT095727.1 | | 805 | | 5 | | 0 | -6.549753385 | | Down |
| NONMMUG039798.2 | NONMMUT064488.2 | | 1382 | | 5 | | 0 | -6.549753385 | | Down |
| NONMMUG071687.1 | NONMMUT114324.1 | | 407 | | 5 | | 0 | -6.549753385 | | Down |
| NONMMUG006113.2 | NONMMUT009760.2 | | 2480 | | 5 | | 0 | -6.549753385 | | Down |
| NONMMUG042428.2 | NONMMUT068541.2 | | 1375 | | 5 | | 0 | -6.549753385 | | Down |
| NONMMUG041176.2 | NONMMUT066508.2 | | 3478 | | 5 | | 0 | -6.549753385 | | Down |
| NONMMUG000174.2 | NONMMUT000252.2 | | 736 | | 5 | | 0 | -6.549753385 | | Down |
| NONMMUG021333.2 | NONMMUT034685.2 | | 1676 | | 5 | | 0 | -6.549753385 | | Down |
| NONMMUG031642.2 | NONMMUT051072.2 | | 950 | | 5 | | 0 | -6.549753385 | | Down |
| NONMMUG028250.2 | NONMMUT045758.2 | | 3451 | | 5 | | 0 | -6.549753385 | | Down |
| NONMMUG022036.2 | NONMMUT035838.2 | | 466 | | 5 | | 0 | -6.549753385 | | Down |
| NONMMUG046561.1 | NONMMUT075106.1 | | 1273 | | 5 | | 0 | -6.549753385 | | Down |
| NONMMUG062994.1 | NONMMUT100718.1 | | 843 | | 5 | | 0 | -6.549753385 | | Down |
| NONMMUG035404.2 | NONMMUT057050.2 | | 2298 | | 10 | | 1 | -6.549753385 | | Down |
| NONMMUG034434.2 | NONMMUT055476.2 | | 2868 | | 10 | | 1 | -6.549753385 | | Down |
| NONMMUG001877.2 | NONMMUT002775.2 | | 2097 | | 10 | | 1 | -6.549753385 | | Down |
| NONMMUG083421.1 | NONMMUT132511.1 | | 456 | | 10 | | 1 | -6.549753385 | | Down |
| NONMMUG011005.2 | NONMMUT017555.2 | | 2973 | | 10.24 | | 1.01 | -6.58947423 | | Down |
| NONMMUG085563.1 | NONMMUT135699.1 | | 700 | | 5.21 | | 0 | -6.668463941 | | Down |
| NONMMUG032771.2 | NONMMUT052770.2 | | 2681 | | 5.24 | | 0 | -6.685030819 | | Down |
| NONMMUG016673.2 | NONMMUT026881.2 | | 2828 | | 5.52 | | 0 | -6.722066673 | | Down |
| LXLOC_009914 | LTCONS_00017079 | | 3152 | | 5.34 | | 0 | -6.739576679 | | Down |
| NONMMUG032674.2 | NONMMUT052621.2 | | 1886 | | 5.35 | | 0 | -6.744974979 | | Down |
| NONMMUG018074.2 | NONMMUT029231.2 | | 1354 | | 5.36 | | 0 | -6.750363197 | | Down |
| NONMMUG052486.1 | NONMMUT084268.1 | | 715 | | 5.39 | | 0 | -6.766467741 | | Down |
| NONMMUG028954.2 | NONMMUT046829.2 | | 1158 | | 22.46 | | 2.08 | -6.771302184 | | Down |
| NONMMUG000082.2 | NONMMUT000120.2 | | 1532 | | 10.86 | | 1 | -6.787801592 | | Down |
| NONMMUG084778.1 | NONMMUT134475.1 | | 366 | | 11 | | 1 | -6.824760433 | | Down |
| NONMMUG011630.2 | NONMMUT018691.2 | | 1513 | | 5.55 | | 0 | -6.850872738 | | Down |
| NONMMUG066885.1 | NONMMUT107026.1 | | 1025 | | 10.85 | | 0 | -6.902930849 | | Down |
| NONMMUG046728.1 | NONMMUT075352.1 | | 5502 | | 5.7 | | 0 | -6.927821034 | | Down |
| NONMMUG017080.2 | NONMMUT027581.2 | | 4859 | | 12.89 | | 1.13 | -6.929612367 | | Down |
| NONMMUG086413.1 | NONMMUT136797.1 | | 3129 | | 5.75 | | 0 | -6.953021108 | | Down |
| NONMMUG061308.1 | NONMMUT098093.1 | | 1297 | | 9.05 | | 0 | -6.978640722 | | Down |
| NONMMUG001744.2 | NONMMUT002578.2 | | 3418 | | 5.83 | | 0 | -6.992888962 | | Down |
| NONMMUG063593.1 | NONMMUT101626.1 | | 284 | | 5.83 | | 0 | -6.992888962 | | Down |
| NONMMUG013497.2 | NONMMUT021721.2 | | 3075 | | 5.89 | | 0 | -7.022432464 | | Down |
| NONMMUG001517.2 | NONMMUT002256.2 | | 2140 | | 5.99 | | 0 | -7.071009202 | | Down |
| LXLOC_093375 | LTCONS_00161888 | | 469 | | 6 | | 0 | -7.075822197 | | Down |
| NONMMUG052753.1 | NONMMUT084691.1 | | 727 | | 6 | | 0 | -7.075822197 | | Down |
| NONMMUG003044.2 | NONMMUT004722.2 | | 786 | | 6 | | 0 | -7.075822197 | | Down |
| NONMMUG061626.1 | NONMMUT098571.1 | | 4843 | | 6 | | 0 | -7.075822197 | | Down |
| NONMMUG016879.2 | NONMMUT027202.2 | | 2740 | | 6 | | 0 | -7.075822197 | | Down |
| NONMMUG047611.1 | NONMMUT076760.1 | | 1176 | | 6 | | 0 | -7.075822197 | | Down |
| NONMMUG009482.2 | NONMMUT014953.2 | | 1439 | | 6 | | 0 | -7.075822197 | | Down |
| NONMMUG011963.2 | NONMMUT019306.2 | | 1560 | | 6 | | 0 | -7.075822197 | | Down |
| NONMMUG047112.1 | NONMMUT075917.1 | | 2928 | | 6 | | 0 | -7.075822197 | | Down |
| NONMMUG068033.1 | NONMMUT108809.1 | | 744 | | 6 | | 0 | -7.075822197 | | Down |
| NONMMUG040382.2 | NONMMUT065313.2 | | 964 | | 6 | | 0 | -7.075822197 | | Down |
| NONMMUG071086.1 | NONMMUT113415.1 | | 320 | | 6 | | 0 | -7.075822197 | | Down |
| NONMMUG056743.1 | NONMMUT091043.1 | | 269 | | 6 | | 0 | -7.075822197 | | Down |
| NONMMUG060064.1 | NONMMUT096061.1 | | 723 | | 6 | | 0 | -7.075822197 | | Down |
| NONMMUG018864.2 | NONMMUT030503.2 | | 1514.99 | | 6 | | 0 | -7.075822197 | | Down |
| NONMMUG052539.1 | NONMMUT084367.1 | | 243 | | 6 | | 0 | -7.075822197 | | Down |
| NONMMUG028525.2 | NONMMUT046205.2 | | 2595 | | 6 | | 0 | -7.075822197 | | Down |
| NONMMUG068884.1 | NONMMUT110150.1 | | 568 | | 12 | | 1 | -7.075822197 | | Down |
| NONMMUG070008.1 | NONMMUT111825.1 | | 1541 | | 12 | | 1 | -7.075822197 | | Down |
| NONMMUG032137.2 | NONMMUT051818.2 | | 2729 | | 12 | | 1 | -7.075822197 | | Down |
| LXLOC_012512 | LTCONS_00021543 | | 702 | | 6.02 | | 0 | -7.08542417 | | Down |
| NONMMUG068236.1 | NONMMUT109117.1 | | 1040 | | 6.06 | | 0 | -7.104532783 | | Down |
| NONMMUG063598.1 | NONMMUT101636.1 | | 2297 | | 13.71 | | 1.12 | -7.133213063 | | Down |
| LXLOC_092842 | LTCONS_00161041 | | 2937 | | 6.38 | | 0 | -7.253010044 | | Down |
| LXLOC_072080 | LTCONS_00126382 | | 1094 | | 6.58 | | 0 | -7.342072363 | | Down |
| LXLOC_030708 | LTCONS_00053189 | | 9185 | | 6.62 | | 0 | -7.35955963 | | Down |
| NONMMUG008106.2 | NONMMUT012763.2 | | 1005 | | 6.79 | | 0 | -7.432720344 | | Down |
| LXLOC_037160 | LTCONS_00064635 | | 2071 | | 6.83 | | 0 | -7.449668353 | | Down |
| NONMMUG010697.2 | NONMMUT017013.2 | | 2733 | | 4.88 | | 0 | -7.508805837 | | Down |
| LXLOC_067730 | LTCONS_00118796 | | 1543 | | 7 | | 0 | -7.52060704 | | Down |
| NONMMUG058026.1 | NONMMUT093071.1 | | 3621 | | 7 | | 0 | -7.52060704 | | Down |
| NONMMUG072556.1 | NONMMUT115718.1 | | 318 | | 7 | | 0 | -7.52060704 | | Down |
| NONMMUG067864.1 | NONMMUT108576.1 | | 1444 | | 7 | | 0 | -7.52060704 | | Down |
| NONMMUG050315.1 | NONMMUT080872.1 | | 867.59 | | 7 | | 0 | -7.52060704 | | Down |
| NONMMUG025609.2 | NONMMUT041363.2 | | 1429 | | 7 | | 0 | -7.52060704 | | Down |
| NONMMUG063846.1 | NONMMUT102012.1 | | 2296 | | 7 | | 0 | -7.52060704 | | Down |
| NONMMUG038335.2 | NONMMUT062092.2 | | 1427 | | 7 | | 0 | -7.52060704 | | Down |
| NONMMUG060740.1 | NONMMUT097169.1 | | 1869 | | 7 | | 0 | -7.52060704 | | Down |
| NONMMUG011751.2 | NONMMUT018872.2 | | 2860 | | 7.05 | | 0 | -7.541143711 | | Down |
| NONMMUG022454.2 | NONMMUT036496.2 | | 1739 | | 7.15 | | 0 | -7.581783679 | | Down |
| NONMMUG042355.2 | NONMMUT068446.2 | | 3231 | | 6.74 | | 0 | -7.589929055 | | Down |
| NONMMUG076894.1 | NONMMUT122577.1 | | 963 | | 7.18 | | 0 | -7.593864884 | | Down |
| NONMMUG049258.1 | NONMMUT079167.1 | | 907 | | 7.27 | | 0 | -7.629807924 | | Down |
| LXLOC_046079 | LTCONS_00080962 | | 913 | | 7.37 | | 0 | -7.669226434 | | Down |
| LXLOC_064826 | LTCONS_00113540 | | 636 | | 7.51 | | 0 | -7.723523011 | | Down |
| NONMMUG055029.1 | NONMMUT088332.1 | | 637 | | 7.59 | | 0 | -7.754096967 | | Down |
| NONMMUG044737.2 | NONMMUT072185.2 | | 438 | | 7.63 | | 0 | -7.76926331 | | Down |
| NONMMUG015155.2 | NONMMUT024514.2 | | 2482 | | 4.43 | | 0 | -7.772260982 | | Down |
| LXLOC_040058 | LTCONS_00070097 | | 15711 | | 7.7 | | 0 | -7.795614087 | | Down |
| NONMMUG019458.2 | NONMMUT031662.2 | | 3910 | | 7.75 | | 0 | -7.814289816 | | Down |
| NONMMUG036675.2 | NONMMUT059024.2 | | 3055 | | 7.77 | | 0 | -7.821726393 | | Down |
| NONMMUG066483.1 | NONMMUT106400.1 | | 3841 | | 16.92 | | 1.06 | -7.899083993 | | Down |
| NONMMUG087209.1 | NONMMUT137913.1 | | 2894 | | 7.99 | | 0 | -7.902288202 | | Down |
| NONMMUG007262.2 | NONMMUT011492.2 | | 2617 | | 8 | | 0 | -7.905897196 | | Down |
| NONMMUG087002.1 | NONMMUT137570.1 | | 874 | | 8 | | 0 | -7.905897196 | | Down |
| NONMMUG073822.1 | NONMMUT117662.1 | | 4472 | | 8 | | 0 | -7.905897196 | | Down |
| NONMMUG013999.2 | NONMMUT022670.2 | | 603 | | 8 | | 0 | -7.905897196 | | Down |
| NONMMUG072736.1 | NONMMUT116021.1 | | 699 | | 8 | | 0 | -7.905897196 | | Down |
| NONMMUG080898.1 | NONMMUT128859.1 | | 976 | | 8 | | 0 | -7.905897196 | | Down |
| NONMMUG080298.1 | NONMMUT127946.1 | | 1054 | | 8 | | 0 | -7.905897196 | | Down |
| NONMMUG041112.2 | NONMMUT066416.2 | | 1638 | | 5.37 | | 0 | -8.043453752 | | Down |
| LXLOC_024700 | LTCONS_00043006 | | 1582 | | 8.4 | | 0 | -8.046675851 | | Down |
| LXLOC_050693 | LTCONS_00088972 | | 2185 | | 8.61 | | 0 | -8.117923671 | | Down |
| NONMMUG023705.2 | NONMMUT038368.2 | | 228 | | 8.74 | | 0 | -8.161163755 | | Down |
| LXLOC_013512 | LTCONS_00023343 | | 1093 | | 8.76 | | 0 | -8.167758935 | | Down |
| NONMMUG041289.2 | NONMMUT066722.2 | | 1490 | | 8.97 | | 0 | -8.236113166 | | Down |
| NONMMUG067216.1 | NONMMUT107516.1 | | 478 | | 9 | | 0 | -8.245747198 | | Down |
| NONMMUG040874.2 | NONMMUT128858.1 | | 1513 | | 9 | | 0 | -8.245747198 | | Down |
| NONMMUG008412.2 | NONMMUT013257.2 | | 8220 | | 9.35 | | 0 | -8.355829926 | | Down |
| NONMMUG038154.2 | NONMMUT061512.2 | | 687 | | 9.4 | | 0 | -8.371218709 | | Down |
| LXLOC_064015 | LTCONS_00111932 | | 3497.01 | | 9.47 | | 0 | -8.392626047 | | Down |
| NONMMUG012287.2 | NONMMUT019768.2 | | 257 | | 9.5 | | 0 | -8.401752222 | | Down |
| NONMMUG069576.1 | NONMMUT111211.1 | | 619 | | 4.12 | | 0 | -8.494263404 | | Down |
| NONMMUG051358.1 | NONMMUT082530.1 | | 746 | | 10 | | 0 | -8.549753385 | | Down |
| NONMMUG069438.1 | NONMMUT111007.1 | | 1562 | | 11.01 | | 0 | -8.827382323 | | Down |
| NONMMUG039287.2 | NONMMUT063609.2 | | 1920 | | 11.23 | | 0 | -8.884469241 | | Down |
| LXLOC_053903 | LTCONS_00094655 | | 1943 | | 11.48 | | 0 | -8.947998669 | | Down |
| LXLOC_037187 | LTCONS_00064685 | | 848 | | 11.54 | | 0 | -8.963039833 | | Down |
| NONMMUG018862.2 | NONMMUT030500.2 | | 4353 | | 11.65 | | 0 | -8.990413295 | | Down |
| NONMMUG041927.2 | NONMMUT067707.2 | | 1633 | | 12.35 | | 0 | -9.158775469 | | Down |
| NONMMUG024871.2 | NONMMUT040160.2 | | 1042 | | 4.61 | | 0 | -9.263293074 | | Down |
| LXLOC_016237 | LTCONS_00028184 | | 2692 | | 12.87 | | 0 | -9.277777492 | | Down |
| NONMMUG025822.2 | NONMMUT041741.2 | | 711 | | 13.29 | | 0 | -9.370435595 | | Down |
| LXLOC_019095 | LTCONS_00033138 | | 4226 | | 13.4 | | 0 | -9.394219387 | | Down |
| NONMMUG011127.2 | NONMMUT017751.2 | | 1241 | | 4.45 | | 0 | -9.687439056 | | Down |
| LXLOC_074300 | LTCONS_00130328 | | 8104 | | 15.37 | | 0 | -9.789987715 | | Down |
| LXLOC_087020 | LTCONS_00151657 | | 1035 | | 15.96 | | 0 | -9.898674688 | | Down |
| LXLOC_058335 | LTCONS_00102116 | | 4420 | | 16.23 | | 0 | -9.947079385 | | Down |
| NONMMUG063273.1 | NONMMUT101138.1 | | 1062 | | 5.3 | | 0 | -10.1918131 | | Down |
| LXLOC_037853 | LTCONS_00065874 | | 2539 | | 19.33 | | 0 | -10.45143666 | | Down |
| NONMMUG070577.1 | NONMMUT112692.1 | | 1301 | | 4 | | 0 | -10.54975339 | | Down |
| LXLOC_030284 | LTCONS_00052421 | | 990 | | 21.94 | | 0 | -10.81688044 | | Down |
| LXLOC_077550 | LTCONS_00135904 | | 997 | | 22.5 | | 0 | -10.88960339 | | Down |
| LXLOC_019813 | LTCONS_00034426 | | 1292 | | 22.59 | | 0 | -10.90112193 | | Down |
| NONMMUG028998.2 | NONMMUT046915.2 | | 684 | | 22.63 | | 0 | -10.90622655 | | Down |
| NONMMUG033581.2 | NONMMUT054153.2 | | 2144 | | 7.08 | | 0 | -11.0273271 | | Down |
| NONMMUG022731.2 | NONMMUT036933.2 | | 2547 | | 4.08 | | 0 | -11.25074788 | | Down |
| NONMMUG009536.2 | NONMMUT015027.2 | | 2589 | | 26.11 | | 0 | -11.3189583 | | Down |
| NONMMUG009519.2 | NONMMUT015000.2 | | 1047 | | 4.17 | | 0 | -11.6989945 | | Down |
| LXLOC_008758 | LTCONS_00015085 | | 2025 | | 33.59 | | 0 | -12.04581698 | | Down |
| LXLOC_027892 | LTCONS_00048404 | | 756 | | 37 | | 0 | -12.32480393 | | Down |
| NONMMUG023423.2 | NONMMUT037925.2 | | 2707 | | 38.4 | | 0 | -12.43196601 | | Down |
| LXLOC_001162 | LTCONS_00002013 | | 1474 | | 38.89 | | 0 | -12.46855185 | | Down |
| LXLOC_064029 | LTCONS_00111995 | | 1552 | | 38.92 | | 0 | -12.47077681 | | Down |
| LXLOC_013329 | LTCONS_00023023 | | 1364 | | 64.05 | | 0 | -13.90815053 | | Down |
| NONMMUG008644.2 | NONMMUT013641.2 | | 4304 | | 72.76 | | 0 | -14.27604447 | | Down |
| LXLOC_040051 | LTCONS_00070083 | | 1435 | | 86.03 | | 0 | -14.75943306 | | Down |
| LXLOC_053139 | LTCONS_00093380 | | 957 | | 7.79 | | 0 | -15.11685623 | | Down |
| NONMMUG045930.2 | NONMMUT074073.2 | | 991 | | 123.46 | | 0 | -15.80169697 | | Down |
| LXLOC_066604 | LTCONS_00116609 | | 1915 | | 128.15 | | 0 | -20.02706391 | | Down |
| 1. **CA/CPR_vs_Sham 2.0 fold up/down regulated mRNAs (*P* < 0.05)** | | | | | | | | | | |
| GeneID | Length | Fold change | | NSSm Readnum | | NSCA-CPR Readnum | | | Up-Down-Regulation | |
| Gm1987 | 870 | 16.7709516 | | 0 | | 161.84 | | | Up | |
| Dcbld2 | 6487 | 15.1402857 | | 0 | | 91.97 | | | Up | |
| Zfp85 | 2218 | 12.8441817 | | 0 | | 41.5 | | | Up | |
| MXLOC_038557 | 4671 | 12.5262364 | | 0 | | 37.17 | | | Up | |
| Ndst2 | 3907 | 12.0309933 | | 0 | | 8.14 | | | Up | |
| St3gal2 | 4470 | 12.0145701 | | 0 | | 31.13 | | | Up | |
| MXLOC_055775 | 5338 | 12.0024954 | | 0 | | 31 | | | Up | |
| MXLOC_076352 | 6295.37 | 11.4468633 | | 0 | | 25.57 | | | Up | |
| MXLOC_092825 | 7373 | 11.3818152 | | 0 | | 25 | | | Up | |
| Rpl3 | 1361 | 11.2640278 | | 0 | | 24 | | | Up | |
| Taok1 | 12409 | 11.1349473 | | 0 | | 22.95 | | | Up | |
| MXLOC_004371 | 8948 | 11.0077151 | | 0 | | 21.96 | | | Up | |
| Clock | 9787 | 11.0037706 | | 0 | | 21.93 | | | Up | |
| Arhgap26 | 7899 | 10.9813161 | | 0 | | 21.76 | | | Up | |
| Wwp1 | 6454 | 10.7176902 | | 0 | | 19.86 | | | Up | |
| Mfn2 | 4473.08 | 10.6156597 | | 0 | | 19.17 | | | Up | |
| Tubgcp6 | 6957 | 10.4065724 | | 0 | | 17.83 | | | Up | |
| Rhbdd2 | 4171 | 10.2893244 | | 0 | | 17.12 | | | Up | |
| Tmem44 | 4241 | 10.2262812 | | 0 | | 16.75 | | | Up | |
| Eif5b | 7797 | 10.1933643 | | 0 | | 16.56 | | | Up | |
| Brsk2 | 4506 | 10.0173495 | | 0 | | 15.58 | | | Up | |
| Orc4 | 4029 | 9.90980695 | | 1 | | 30.02 | | | Up | |
| Trim59 | 2920 | 9.90017934 | | 0 | | 14.96 | | | Up | |
| Slc18a3 | 2414 | 9.70881265 | | 0 | | 14 | | | Up | |
| Shc1 | 3554 | 9.582425 | | 0 | | 13.4 | | | Up | |
| Rrm2b | 4512 | 9.46822418 | | 0 | | 12.88 | | | Up | |
| Kcnab1 | 3267 | 9.46149571 | | 0 | | 12.85 | | | Up | |
| Zfp276 | 4877 | 9.30224545 | | 0 | | 12.16 | | | Up | |
| Gcfc2 | 3375 | 9.28558748 | | 0 | | 12.09 | | | Up | |
| Tgtp2 | 2816 | 9.26402781 | | 0 | | 12 | | | Up | |
| Nckap1 | 4405 | 9.26402781 | | 0 | | 12 | | | Up | |
| Knop1 | 5386 | 9.25198023 | | 0 | | 11.95 | | | Up | |
| Aldh3a2 | 3193 | 9.24103653 | | 0 | | 5 | | | Up | |
| Prrc1 | 4801 | 9.22773311 | | 0 | | 11.85 | | | Up | |
| Sertad2 | 5730 | 9.01296604 | | 0 | | 11 | | | Up | |
| Dixdc1 | 5610 | 8.93318059 | | 0 | | 10.7 | | | Up | |
| Myef2 | 2927 | 8.86671745 | | 1.26 | | 26.35 | | | Up | |
| MXLOC_038778 | 7971 | 8.86496488 | | 0 | | 10.45 | | | Up | |
| Ap2m1 | 2931 | 8.85943731 | | 0 | | 10.43 | | | Up | |
| Sfxn5 | 3654 | 8.82884494 | | 0 | | 10.32 | | | Up | |
| E2f4 | 1993 | 8.81763952 | | 0 | | 10.28 | | | Up | |
| MXLOC_030178 | 9775 | 8.73795899 | | 0 | | 10 | | | Up | |
| Golim4 | 4467 | 8.73795899 | | 0 | | 10 | | | Up | |
| Eid2b | 2424 | 8.73795899 | | 0 | | 10 | | | Up | |
| 1700066B19Rik | 2504 | 8.73795899 | | 0 | | 10 | | | Up | |
| Adgrv1 | 19328 | 8.73795899 | | 0 | | 10 | | | Up | |
| Meiob | 1703 | 8.73795899 | | 0 | | 10 | | | Up | |
| Cox8a | 545 | 8.73795899 | | 0 | | 10 | | | Up | |
| Ide | 4964 | 8.59299349 | | 0 | | 9.51 | | | Up | |
| Mlxip | 2649 | 8.52206317 | | 0 | | 7.98 | | | Up | |
| MXLOC_067275 | 5552 | 8.50050512 | | 0 | | 9.21 | | | Up | |
| MXLOC_005691 | 2869 | 8.43395281 | | 0 | | 9 | | | Up | |
| MXLOC_036340 | 2611 | 8.43395281 | | 0 | | 9 | | | Up | |
| Nudt22 | 999 | 8.43395281 | | 0 | | 9 | | | Up | |
| Fastkd3 | 2362 | 8.43395281 | | 0 | | 9 | | | Up | |
| Rwdd4a | 2952 | 8.43395281 | | 0 | | 9 | | | Up | |
| Acvr2b | 10594 | 8.43074504 | | 0 | | 8.99 | | | Up | |
| Plekha2 | 4985 | 8.38219554 | | 0 | | 8.84 | | | Up | |
| Zfp609 | 8585 | 8.35266884 | | 0 | | 8.75 | | | Up | |
| MXLOC_007416 | 3253 | 8.32949289 | | 0 | | 8.68 | | | Up | |
| MXLOC_056238 | 9599 | 8.24516807 | | 0 | | 8.43 | | | Up | |
| Lrrc20 | 2621 | 8.21189018 | | 0 | | 4 | | | Up | |
| MXLOC_024994 | 9872 | 8.15124111 | | 0 | | 8.16 | | | Up | |
| Slco5a1 | 8511 | 8.12994662 | | 1 | | 16.2 | | | Up | |
| MXLOC_025874 | 5714 | 8.0941028 | | 0 | | 8 | | | Up | |
| MXLOC_051608 | 1244 | 8.0941028 | | 0 | | 8 | | | Up | |
| Klhl1 | 4048 | 8.0941028 | | 0 | | 8 | | | Up | |
| Slc10a4 | 2085 | 8.0941028 | | 0 | | 8 | | | Up | |
| Gdpd3 | 1068 | 8.0941028 | | 0 | | 8 | | | Up | |
| Nfatc3 | 6014 | 8.0941028 | | 0 | | 8 | | | Up | |
| Lkaaear1 | 716 | 8.0941028 | | 0 | | 8 | | | Up | |
| Herpud2 | 2744 | 8.0941028 | | 0 | | 8 | | | Up | |
| MXLOC_040079 | 4336 | 8.07601253 | | 0 | | 7.95 | | | Up | |
| MXLOC_075690 | 3335 | 8.05049406 | | 0 | | 7.88 | | | Up | |
| Rfx3 | 9146 | 8.01734946 | | 0 | | 7.79 | | | Up | |
| AI597479 | 3457 | 7.99876994 | | 0 | | 7.74 | | | Up | |
| Col4a1 | 6615 | 7.9536848 | | 0 | | 7.62 | | | Up | |
| Dpy19l1 | 4739 | 7.94610164 | | 0 | | 7.6 | | | Up | |
| Rpl29 | 710 | 7.94610164 | | 0 | | 7.6 | | | Up | |
| Fndc5 | 2702 | 7.907884 | | 0 | | 9 | | | Up | |
| Tas2r125 | 936 | 7.907884 | | 1 | | 15 | | | Up | |
| Aunip | 1270 | 7.907884 | | 1 | | 15 | | | Up | |
| Gm6878 | 2018 | 7.907884 | | 2 | | 30 | | | Up | |
| MXLOC_092926 | 11882 | 7.8495913 | | 0 | | 7.35 | | | Up | |
| Plxna3 | 6943 | 7.78340973 | | 0 | | 4.31 | | | Up | |
| MXLOC_091100 | 7929 | 7.74974085 | | 0 | | 7.1 | | | Up | |
| MXLOC_062084 | 2041 | 7.74567406 | | 0 | | 7.09 | | | Up | |
| Gli1 | 3662 | 7.70881265 | | 0 | | 7 | | | Up | |
| P2ry1 | 3624 | 7.70881265 | | 0 | | 7 | | | Up | |
| Myom1 | 5319 | 7.70881265 | | 0 | | 7 | | | Up | |
| Mlycd | 2100 | 7.70881265 | | 0 | | 7 | | | Up | |
| Tas2r116 | 918 | 7.70881265 | | 0 | | 7 | | | Up | |
| Cypt4 | 644 | 7.70881265 | | 0 | | 7 | | | Up | |
| BC051019 | 1942 | 7.70881265 | | 0 | | 7 | | | Up | |
| Lrrc17 | 2188 | 7.70881265 | | 1 | | 14 | | | Up | |
| Tas2r113 | 930 | 7.70881265 | | 1 | | 14 | | | Up | |
| Ccdc34 | 2079 | 7.70468772 | | 0 | | 6.99 | | | Up | |
| Dnajc28 | 3365 | 7.54776524 | | 0 | | 6.62 | | | Up | |
| MXLOC_062421 | 10528 | 7.53027797 | | 0 | | 6.58 | | | Up | |
| Col8a1 | 5110 | 7.49498224 | | 1 | | 13 | | | Up | |
| MXLOC_078106 | 10237 | 7.46822418 | | 0 | | 6.44 | | | Up | |
| Bbof1 | 2190 | 7.42306849 | | 0 | | 6.34 | | | Up | |
| MXLOC_023295 | 8678 | 7.41395192 | | 0 | | 6.32 | | | Up | |
| Vmn2r42 | 3619 | 7.38181518 | | 0 | | 6.25 | | | Up | |
| Lrch1 | 4626 | 7.37573428 | | 0 | | 4.74 | | | Up | |
| Tbc1d24 | 7767.58 | 7.36793197 | | 0 | | 6.22 | | | Up | |
| MXLOC_073369 | 3408 | 7.26402781 | | 0 | | 6 | | | Up | |
| MXLOC_063741 | 3437 | 7.26402781 | | 0 | | 6 | | | Up | |
| 1600015I10Rik | 2484 | 7.26402781 | | 0 | | 6 | | | Up | |
| Smarcad1 | 4637 | 7.26402781 | | 0 | | 6 | | | Up | |
| Ythdf2 | 4078 | 7.26402781 | | 0 | | 6 | | | Up | |
| Tbc1d13 | 3470 | 7.26402781 | | 0 | | 6 | | | Up | |
| Spata33 | 518 | 7.26402781 | | 0 | | 6 | | | Up | |
| Cyth1 | 3119 | 7.26402781 | | 0 | | 6 | | | Up | |
| Arhgef11 | 5600 | 7.26402781 | | 0 | | 6 | | | Up | |
| Hspbap1 | 4606 | 7.26402781 | | 0 | | 6 | | | Up | |
| Zp2 | 2200 | 7.26402781 | | 0 | | 6 | | | Up | |
| Acvr1c | 8658 | 7.26402781 | | 0 | | 6 | | | Up | |
| Ttc3 | 7295 | 7.26402781 | | 0 | | 6 | | | Up | |
| U2surp | 7604 | 7.26402781 | | 0 | | 6 | | | Up | |
| Sirt2 | 1653 | 7.26402781 | | 0 | | 6 | | | Up | |
| Zdhhc2 | 2498 | 7.26402781 | | 0 | | 6 | | | Up | |
| MXLOC_039681 | 3286 | 7.26402781 | | 1 | | 12 | | | Up | |
| Mrgpra6 | 1016 | 7.26402781 | | 1 | | 12 | | | Up | |
| Dnajb12 | 1774 | 7.23502867 | | 0 | | 5.94 | | | Up | |
| MXLOC_064946 | 3035 | 7.17614111 | | 0 | | 5.82 | | | Up | |
| Abca1 | 10260 | 7.10080027 | | 0 | | 5.67 | | | Up | |
| Cacna1g | 8082 | 7.09060454 | | 0 | | 5.65 | | | Up | |
| Zmym4 | 7009 | 7.08037265 | | 0 | | 5.63 | | | Up | |
| MXLOC_039983 | 5654 | 7.07524307 | | 0 | | 5.62 | | | Up | |
| Arhgef7 | 4929 | 7.07010435 | | 0 | | 5.61 | | | Up | |
| Ctxn3 | 1472 | 7.01296604 | | 1 | | 11 | | | Up | |
| Trmt10c | 1846 | 7.01296604 | | 1 | | 11 | | | Up | |
| Cav1 | 2526 | 7.0077151 | | 0 | | 5.49 | | | Up | |
| MXLOC_062630 | 1051 | 6.91152653 | | 0 | | 5.31 | | | Up | |
| Ahnak | 18100 | 6.88970873 | | 0 | | 5.27 | | | Up | |
| Parp12 | 3229 | 6.8842284 | | 0 | | 5.26 | | | Up | |
| Hnrnpc | 2844 | 6.84557188 | | 0 | | 5.19 | | | Up | |
| Vps13c | 11523 | 6.840007 | | 0 | | 5.18 | | | Up | |
| Rassf1 | 1731 | 6.82884494 | | 0 | | 5.16 | | | Up | |
| Rps13 | 582 | 6.81763952 | | 0 | | 5.14 | | | Up | |
| Dclk1 | 5609.45 | 6.77237757 | | 0 | | 5.06 | | | Up | |
| Trim13 | 1639 | 6.77237757 | | 0 | | 5.06 | | | Up | |
| MXLOC_000694 | 7401 | 6.76095027 | | 0 | | 5.04 | | | Up | |
| MXLOC_051624 | 1511 | 6.73795899 | | 0 | | 5 | | | Up | |
| MXLOC_076257 | 4177 | 6.73795899 | | 0 | | 5 | | | Up | |
| MXLOC_027875 | 4861 | 6.73795899 | | 0 | | 5 | | | Up | |
| MXLOC_012861 | 7415 | 6.73795899 | | 0 | | 5 | | | Up | |
| MXLOC_027537 | 2424 | 6.73795899 | | 0 | | 5 | | | Up | |
| MXLOC_051641 | 4852 | 6.73795899 | | 0 | | 5 | | | Up | |
| MXLOC_064303 | 3040 | 6.73795899 | | 0 | | 5 | | | Up | |
| Cwf19l2 | 3953 | 6.73795899 | | 0 | | 5 | | | Up | |
| Fdx1 | 1212 | 6.73795899 | | 0 | | 5 | | | Up | |
| Nol6 | 4483 | 6.73795899 | | 0 | | 5 | | | Up | |
| Pmaip1 | 2654 | 6.73795899 | | 0 | | 5 | | | Up | |
| Rtp4 | 1573 | 6.73795899 | | 0 | | 5 | | | Up | |
| Eif2a | 2299 | 6.73795899 | | 0 | | 5 | | | Up | |
| Ndc80 | 2201 | 6.73795899 | | 0 | | 5 | | | Up | |
| Krt27 | 1564 | 6.73795899 | | 0 | | 5 | | | Up | |
| Ccr4 | 2787 | 6.73795899 | | 0 | | 5 | | | Up | |
| 1300017J02Rik | 2275 | 6.73795899 | | 0 | | 5 | | | Up | |
| Fn1 | 7522 | 6.73795899 | | 0 | | 5 | | | Up | |
| Hacd1 | 886 | 6.73795899 | | 0 | | 5 | | | Up | |
| Ei24 | 2226 | 6.73795899 | | 0 | | 5 | | | Up | |
| Rhbdf2 | 3644 | 6.73795899 | | 0 | | 5 | | | Up | |
| Tmem167b | 2864 | 6.73795899 | | 0 | | 5 | | | Up | |
| Dnah7a | 12272 | 6.73795899 | | 0 | | 5 | | | Up | |
| Spry2 | 2087 | 6.73795899 | | 0 | | 5 | | | Up | |
| Fcrla | 1658 | 6.73795899 | | 0 | | 5 | | | Up | |
| Inhbe | 2135 | 6.73795899 | | 1 | | 10 | | | Up | |
| Zfp810 | 3238 | 6.73795899 | | 1 | | 10 | | | Up | |
| Tas2r123 | 1002 | 6.73795899 | | 2 | | 20 | | | Up | |
| Brf2 | 1383 | 6.66570266 | | 0 | | 9.07 | | | Up | |
| MXLOC_037223 | 2474 | 6.64699345 | | 0 | | 8.43 | | | Up | |
| Chrac1 | 851 | 6.64185474 | | 1.16 | | 11.22 | | | Up | |
| 1700047I17Rik2 | 3841 | 6.53972806 | | 6.13 | | 57.23 | | | Up | |
| Reep2 | 1920 | 6.43888089 | | 0 | | 5.86 | | | Up | |
| MXLOC_005490 | 3371 | 6.43395281 | | 1 | | 9 | | | Up | |
| Olfr657 | 960 | 6.43395281 | | 1 | | 9 | | | Up | |
| Tmem33 | 6404 | 6.43395281 | | 1 | | 9 | | | Up | |
| Rspry1 | 3849 | 6.43395281 | | 1 | | 9 | | | Up | |
| Mrps22 | 1216 | 6.43395281 | | 1 | | 9 | | | Up | |
| Rgs9 | 7629 | 6.43395281 | | 2 | | 18 | | | Up | |
| 1110059G10Rik | 3361 | 6.18529654 | | 1.09 | | 9 | | | Up | |
| MXLOC_021770 | 13868 | 6.0941028 | | 1 | | 8 | | | Up | |
| Rybp | 4376 | 6.0941028 | | 1 | | 8 | | | Up | |
| Arhgdia | 2732 | 6.0941028 | | 1 | | 8 | | | Up | |
| Cript | 1171 | 6.0941028 | | 1 | | 8 | | | Up | |
| Mnd1 | 874 | 6.0941028 | | 1 | | 8 | | | Up | |
| Mtmr3 | 5674 | 6.0941028 | | 1 | | 8 | | | Up | |
| Parp8 | 3088 | 6.0941028 | | 1 | | 8 | | | Up | |
| Rgs3 | 4001 | 6.0941028 | | 1 | | 8 | | | Up | |
| Sec16a | 8739 | 6.0941028 | | 1 | | 8 | | | Up | |
| Dnajb3 | 1017 | 6.0941028 | | 2 | | 16 | | | Up | |
| Hrh2 | 2789 | 5.94610164 | | 5 | | 38 | | | Up | |
| MXLOC_084713 | 8289 | 5.92990998 | | 1.74 | | 13.15 | | | Up | |
| Zc3h14 | 3088 | 5.84304104 | | 1.89 | | 13.86 | | | Up | |
| Robo4 | 3839 | 5.81006479 | | 4 | | 29 | | | Up | |
| MXLOC_089020 | 5533 | 5.80132383 | | 1.71 | | 12.36 | | | Up | |
| Tiam2 | 6127 | 5.79559784 | | 1.31 | | 9.45 | | | Up | |
| Disp1 | 4785 | 5.7806518 | | 17 | | 122 | | | Up | |
| Pcdhga4 | 4534 | 5.74426499 | | 1.04 | | 7.37 | | | Up | |
| Zdhhc21 | 8751 | 5.74204208 | | 1.48 | | 10.48 | | | Up | |
| Eif4ebp3 | 1151 | 5.7392216 | | 4.18 | | 29.57 | | | Up | |
| MXLOC_079328 | 8841 | 5.71213492 | | 2.48 | | 17.38 | | | Up | |
| MXLOC_092137 | 3595 | 5.70881265 | | 1 | | 7 | | | Up | |
| MXLOC_080308 | 6481 | 5.70881265 | | 1 | | 7 | | | Up | |
| MXLOC_075168 | 4063 | 5.70881265 | | 1 | | 7 | | | Up | |
| MXLOC_042079 | 6139.33 | 5.70881265 | | 1 | | 7 | | | Up | |
| Tmem47 | 4082 | 5.70881265 | | 1 | | 7 | | | Up | |
| Nxpe5 | 2312 | 5.70881265 | | 1 | | 7 | | | Up | |
| C330007P06Rik | 3339 | 5.70881265 | | 1 | | 7 | | | Up | |
| Slc26a2 | 3863 | 5.70881265 | | 1 | | 7 | | | Up | |
| Tmie | 2437 | 5.70881265 | | 1 | | 7 | | | Up | |
| Msx1 | 1931 | 5.70881265 | | 1 | | 7 | | | Up | |
| Pygl | 2821 | 5.70881265 | | 1 | | 7 | | | Up | |
| Wbp2 | 1795 | 5.70881265 | | 1 | | 7 | | | Up | |
| Wnt6 | 2068 | 5.70881265 | | 1 | | 7 | | | Up | |
| Polrmt | 3762 | 5.70881265 | | 1 | | 7 | | | Up | |
| Ighmbp2 | 5562 | 5.70881265 | | 1 | | 7 | | | Up | |
| Slc35d3 | 2629 | 5.70881265 | | 1 | | 7 | | | Up | |
| S100a16 | 1061 | 5.70881265 | | 1 | | 7 | | | Up | |
| Cdsn | 2678 | 5.70881265 | | 1 | | 7 | | | Up | |
| Spry1 | 2163 | 5.70881265 | | 1 | | 7 | | | Up | |
| Olfr652 | 1061 | 5.70881265 | | 1 | | 7 | | | Up | |
| Egf | 4755 | 5.70881265 | | 1 | | 7 | | | Up | |
| Wnt8b | 3376 | 5.70881265 | | 1 | | 7 | | | Up | |
| Dagla | 5634 | 5.70881265 | | 1 | | 7 | | | Up | |
| Olfr456 | 1052 | 5.69642012 | | 1 | | 6.97 | | | Up | |
| Nhsl2 | 12971 | 5.69083508 | | 1.15 | | 8 | | | Up | |
| Nasp | 2037 | 5.63078044 | | 2.57 | | 17.51 | | | Up | |
| MXLOC_051304 | 3045 | 5.49498224 | | 2 | | 13 | | | Up | |
| Ctxn1 | 1226 | 5.49498224 | | 2 | | 13 | | | Up | |
| Plac9b | 998 | 5.38181518 | | 4 | | 25 | | | Up | |
| Abcb9 | 3334 | 5.38181518 | | 4 | | 25 | | | Up | |
| MXLOC_078425 | 6329 | 5.31570782 | | 1.66 | | 10.14 | | | Up | |
| MXLOC_089966 | 4904 | 5.26402781 | | 1 | | 6 | | | Up | |
| MXLOC_078171 | 977 | 5.26402781 | | 1 | | 6 | | | Up | |
| MXLOC_049122 | 1653 | 5.26402781 | | 1 | | 6 | | | Up | |
| Adgrf4 | 2977 | 5.26402781 | | 1 | | 6 | | | Up | |
| Rab9 | 1448 | 5.26402781 | | 1 | | 6 | | | Up | |
| Mrpl44 | 1424 | 5.26402781 | | 1 | | 6 | | | Up | |
| Zfhx3 | 16439 | 5.26402781 | | 1 | | 6 | | | Up | |
| Col9a3 | 2846 | 5.26402781 | | 1 | | 6 | | | Up | |
| Mov10 | 3563 | 5.26402781 | | 1 | | 6 | | | Up | |
| Mthfd2 | 2070 | 5.26402781 | | 1 | | 6 | | | Up | |
| Ndufb8 | 641 | 5.26402781 | | 1 | | 6 | | | Up | |
| Lbr | 3552 | 5.26402781 | | 1 | | 6 | | | Up | |
| Znhit3 | 983 | 5.26402781 | | 1 | | 6 | | | Up | |
| Asb13 | 2410 | 5.26402781 | | 1 | | 6 | | | Up | |
| Zyx | 3399 | 5.26402781 | | 1 | | 6 | | | Up | |
| BC027072 | 5006 | 5.26402781 | | 1 | | 6 | | | Up | |
| Prss54 | 1152 | 5.26402781 | | 1 | | 6 | | | Up | |
| Ubqln4 | 3372 | 5.26402781 | | 1 | | 6 | | | Up | |
| Zbtb48 | 2226 | 5.26402781 | | 1 | | 6 | | | Up | |
| Tmigd3 | 1381.62 | 5.26402781 | | 1 | | 6 | | | Up | |
| Plekhh3 | 3006 | 5.26402781 | | 1 | | 6 | | | Up | |
| F3 | 1876 | 5.26402781 | | 1 | | 6 | | | Up | |
| Tigd4 | 3459 | 5.26402781 | | 1 | | 6 | | | Up | |
| Khdrbs3 | 1893 | 5.26402781 | | 1 | | 6 | | | Up | |
| Snx11 | 2521 | 5.26402781 | | 1 | | 6 | | | Up | |
| Pdlim5 | 2415 | 5.26402781 | | 2 | | 12 | | | Up | |
| Vmn2r112 | 2574 | 5.26402781 | | 2 | | 12 | | | Up | |
| Gltscr1 | 5360 | 5.26402781 | | 2 | | 12 | | | Up | |
| Grhl3 | 2779 | 5.26402781 | | 2 | | 12 | | | Up | |
| Rhbdd1 | 3510 | 5.26402781 | | 2 | | 12 | | | Up | |
| Emilin1 | 3464 | 5.25921481 | | 1 | | 5.99 | | | Up | |
| MXLOC_013320 | 1884 | 5.21997519 | | 2.09 | | 12.35 | | | Up | |
| MXLOC_087821 | 7174 | 5.20035563 | | 2.52 | | 14.79 | | | Up | |
| Emc6 | 1353 | 5.17614111 | | 1 | | 5.82 | | | Up | |
| Tsc22d4 | 2349 | 5.17366119 | | 2 | | 11.63 | | | Up | |
| Cpeb3 | 5901.05 | 5.10398364 | | 1.39 | | 7.89 | | | Up | |
| Creb3l4 | 1476 | 5.09910349 | | 3 | | 17 | | | Up | |
| Gm3417 | 787 | 5.01838408 | | 9.19 | | 50.64 | | | Up | |
| Sdr9c7 | 2849 | 5.01296604 | | 2 | | 11 | | | Up | |
| Golga7b | 2823 | 5.01296604 | | 2 | | 11 | | | Up | |
| Pianp | 2054 | 5.01296604 | | 2 | | 11 | | | Up | |
| Auts2 | 6059 | 5.01296604 | | 2 | | 11 | | | Up | |
| Fxr2 | 2970 | 5.01296604 | | 2 | | 11 | | | Up | |
| Doxl2 | 2288 | 5.01296604 | | 2 | | 11 | | | Up | |
| Galnt10 | 4701 | 4.9933241 | | 2.01 | | 10.98 | | | Up | |
| MXLOC_036317 | 3719.52 | 4.96421574 | | 3.31 | | 17.9 | | | Up | |
| Samd4b | 4391 | 4.95943759 | | 1.83 | | 9.88 | | | Up | |
| MXLOC_040771 | 3155.38 | 4.91824824 | | 8.22 | | 43.75 | | | Up | |
| MXLOC_073382 | 3249 | 4.89808777 | | 3.68 | | 19.45 | | | Up | |
| Iqsec3 | 6574 | 4.86588511 | | 1.5 | | 7.84 | | | Up | |
| MXLOC_020883 | 5427 | 4.86434665 | | 1.34 | | 7 | | | Up | |
| Abcc4 | 5504 | 4.85112605 | | 5 | | 26 | | | Up | |
| MXLOC_001696 | 4111 | 4.73795899 | | 2 | | 10 | | | Up | |
| MXLOC_016234 | 8374 | 4.73795899 | | 2 | | 10 | | | Up | |
| Tagln3 | 1163 | 4.73795899 | | 2 | | 10 | | | Up | |
| A1bg | 1823 | 4.73795899 | | 2 | | 10 | | | Up | |
| Pglyrp2 | 2369 | 4.73795899 | | 2 | | 10 | | | Up | |
| Draxin | 5200 | 4.73795899 | | 2 | | 10 | | | Up | |
| Smc1a | 3970 | 4.73795899 | | 2 | | 10 | | | Up | |
| 1810011O10Rik | 1331 | 4.73795899 | | 2 | | 10 | | | Up | |
| Arhgap12 | 4957 | 4.73795899 | | 3 | | 15 | | | Up | |
| Il1a | 1974 | 4.73795899 | | 4 | | 20 | | | Up | |
| Ifnz | 1462 | 4.73245777 | | 3.15 | | 15.72 | | | Up | |
| Cuta | 658 | 4.69703079 | | 1.42 | | 7 | | | Up | |
| Rbks | 1025 | 4.68859047 | | 2.24 | | 11.01 | | | Up | |
| Haghl | 1129 | 4.68827085 | | 1.64 | | 8.06 | | | Up | |
| Hadhb | 2028 | 4.68651632 | | 2.49 | | 12.23 | | | Up | |
| Papd5 | 4495 | 4.63802208 | | 1.88 | | 9.08 | | | Up | |
| MXLOC_061737 | 4273.81 | 4.61434843 | | 2.48 | | 11.88 | | | Up | |
| Ubald2 | 1482 | 4.60250512 | | 1.57 | | 7.49 | | | Up | |
| Zswim1 | 2701 | 4.59841844 | | 4.66 | | 22.2 | | | Up | |
| Mrpl40 | 1055 | 4.5891946 | | 1.99 | | 9.45 | | | Up | |
| Wtap | 2051 | 4.57023779 | | 1.7 | | 8.02 | | | Up | |
| Stxbp2 | 2703 | 4.55844969 | | 1.89 | | 8.88 | | | Up | |
| 4930555G01Rik | 2065 | 4.55635312 | | 2 | | 9.39 | | | Up | |
| Kbtbd7 | 4528 | 4.55635312 | | 2 | | 9.39 | | | Up | |
| MXLOC_003406 | 5343 | 4.55327865 | | 2 | | 9.38 | | | Up | |
| Aars2 | 3349 | 4.53888765 | | 3 | | 14 | | | Up | |
| Gng2 | 3638 | 4.53888765 | | 3 | | 14 | | | Up | |
| MXLOC_018176 | 2005 | 4.49397795 | | 3.7 | | 17 | | | Up | |
| Ehd1 | 3182 | 4.44317342 | | 4.86 | | 21.94 | | | Up | |
| Gfra1 | 4664 | 4.43846476 | | 2.84 | | 12.8 | | | Up | |
| Gstt2 | 1370 | 4.43395281 | | 2 | | 9 | | | Up | |
| Arsj | 3690 | 4.43395281 | | 2 | | 9 | | | Up | |
| Trim47 | 2192.01 | 4.43395281 | | 2 | | 9 | | | Up | |
| Ptpn4 | 4706 | 4.43395281 | | 2 | | 9 | | | Up | |
| 3110052M02Rik | 4367 | 4.43395281 | | 2 | | 9 | | | Up | |
| Plk3 | 2327 | 4.43395281 | | 2 | | 9 | | | Up | |
| Selp | 3438 | 4.43395281 | | 2 | | 9 | | | Up | |
| Lef1 | 3532 | 4.43395281 | | 2 | | 9 | | | Up | |
| Mef2c | 6325 | 4.41496688 | | 17.45 | | 78.01 | | | Up | |
| Slc29a3 | 5240 | 4.40961512 | | 41.67 | | 185.94 | | | Up | |
| Dact2 | 2809 | 4.39810899 | | 2.25 | | 10 | | | Up | |
| Sdr39u1 | 1227 | 4.39282502 | | 17.9 | | 79.41 | | | Up | |
| MXLOC_038308 | 4552 | 4.3850171 | | 3.7 | | 16.37 | | | Up | |
| MXLOC_016171 | 6215 | 4.32505724 | | 3 | | 13 | | | Up | |
| Sema6d | 6296 | 4.32505724 | | 3 | | 13 | | | Up | |
| Mrgpra9 | 1052 | 4.32505724 | | 3 | | 13 | | | Up | |
| Sri | 2399 | 4.32505724 | | 3 | | 13 | | | Up | |
| Gpatch11 | 4207 | 4.32505724 | | 3 | | 13 | | | Up | |
| Htr2c | 4750 | 4.32505724 | | 3 | | 13 | | | Up | |
| Ercc2 | 3547 | 4.29317415 | | 7 | | 30 | | | Up | |
| Ccnl2 | 2442 | 4.29208099 | | 3.52 | | 15.08 | | | Up | |
| Borcs8 | 1994 | 4.28502806 | | 2.01 | | 8.59 | | | Up | |
| Emilin3 | 3417 | 4.28332566 | | 2.25 | | 9.61 | | | Up | |
| Lrrc3 | 4295 | 4.27794778 | | 3.8 | | 16.2 | | | Up | |
| MXLOC_015267 | 4855 | 4.27096759 | | 7 | | 29.77 | | | Up | |
| Aldh1b1 | 2302 | 4.26902849 | | 20 | | 85 | | | Up | |
| MXLOC_031756 | 8325.48 | 4.26682337 | | 3.08 | | 13.08 | | | Up | |
| Mrpl51 | 1515 | 4.25950798 | | 2.5 | | 10.59 | | | Up | |
| MXLOC_005686 | 3228 | 4.24210397 | | 1.9 | | 8 | | | Up | |
| MXLOC_075284 | 9756.54 | 4.23071483 | | 165 | | 692 | | | Up | |
| Stx1b | 1574 | 4.21511679 | | 2.86 | | 11.93 | | | Up | |
| Tcfl5 | 2207 | 4.21189018 | | 12 | | 50 | | | Up | |
| MXLOC_048794 | 2166 | 4.19684701 | | 6 | | 24.87 | | | Up | |
| MXLOC_048970 | 4577 | 4.1837741 | | 7.92 | | 32.68 | | | Up | |
| Ift140 | 5804 | 4.17451545 | | 2.3 | | 9.46 | | | Up | |
| MXLOC_043075 | 10422 | 4.13602204 | | 2.05 | | 8.32 | | | Up | |
| Ttr | 1221 | 4.11954982 | | 10.16 | | 41 | | | Up | |
| MXLOC_053557 | 7453 | 4.09989631 | | 16.17 | | 64.81 | | | Up | |
| Ccm2 | 1701 | 4.0941028 | | 2 | | 8 | | | Up | |
| Smdt1 | 585 | 4.0941028 | | 2 | | 8 | | | Up | |
| Aup1 | 1492 | 4.0941028 | | 2 | | 8 | | | Up | |
| Mansc1 | 2376 | 4.0941028 | | 2 | | 8 | | | Up | |
| Sdhaf2 | 3097 | 4.0941028 | | 2 | | 8 | | | Up | |
| Runx1 | 6929 | 4.0941028 | | 2 | | 8 | | | Up | |
| Naa25 | 3105 | 4.0941028 | | 2 | | 8 | | | Up | |
| Itgae | 3813 | 4.0941028 | | 2 | | 8 | | | Up | |
| Marveld2 | 2203 | 4.0941028 | | 2 | | 8 | | | Up | |
| Il1b | 1348 | 4.0941028 | | 2 | | 8 | | | Up | |
| Abhd8 | 1985 | 4.0941028 | | 2 | | 8 | | | Up | |
| MXLOC_041745 | 2677 | 4.0941028 | | 3 | | 12 | | | Up | |
| Ubac1 | 3450 | 4.0941028 | | 3 | | 12 | | | Up | |
| Lactb2 | 1887 | 4.0941028 | | 3 | | 12 | | | Up | |
| Foxo4 | 3162 | 4.0941028 | | 3 | | 12 | | | Up | |
| F5 | 7433 | 4.0941028 | | 3 | | 12 | | | Up | |
| Nodal | 2094 | 4.0941028 | | 3 | | 12 | | | Up | |
| Rab1b | 1835 | 4.0941028 | | 4 | | 16 | | | Up | |
| MXLOC_011429 | 8686 | 4.0941028 | | 5 | | 20 | | | Up | |
| Zic4 | 4088 | 4.0941028 | | 5 | | 20 | | | Up | |
| Snapc3 | 1385 | 4.0941028 | | 7 | | 28 | | | Up | |
| Zbtb33 | 4948 | 4.07140096 | | 3.19 | | 12.66 | | | Up | |
| Hdac1 | 1971 | 4.0629572 | | 3.26 | | 12.9 | | | Up | |
| Clns1a | 3395 | 4.05348484 | | 4.65 | | 18.34 | | | Up | |
| Ppp1cc | 2379 | 4.04234554 | | 2.25 | | 8.84 | | | Up | |
| Atg13 | 3573 | 4.02405255 | | 8.13 | | 31.74 | | | Up | |
| MXLOC_000803 | 12518 | 4.00249543 | | 8 | | 31 | | | Up | |
| MXLOC_074341 | 10515 | 3.9680576 | | 5.03 | | 19.26 | | | Up | |
| Vegfb | 1181 | 3.96011889 | | 2.81 | | 10.73 | | | Up | |
| MXLOC_017896 | 9319 | 3.93073521 | | 2.18 | | 8.24 | | | Up | |
| Nek7 | 4095 | 3.92313578 | | 4.78 | | 18.02 | | | Up | |
| Pdgfrl | 1533 | 3.907884 | | 4 | | 15 | | | Up | |
| Sycp3 | 1122 | 3.907884 | | 4 | | 15 | | | Up | |
| Ndrg2 | 2115 | 3.89349299 | | 4.02 | | 15 | | | Up | |
| Arid1a | 8175 | 3.89297487 | | 4.01 | | 14.96 | | | Up | |
| Sptlc2 | 6710 | 3.86833665 | | 3.82 | | 14.13 | | | Up | |
| MXLOC_036794 | 4892 | 3.86394976 | | 3 | | 11.08 | | | Up | |
| MXLOC_035500 | 8981 | 3.85029806 | | 3.61 | | 13.27 | | | Up | |
| MXLOC_025083 | 27889 | 3.84630236 | | 20.9 | | 76.72 | | | Up | |
| Treh | 1943 | 3.84304104 | | 3 | | 11 | | | Up | |
| Ptpre | 5388 | 3.84304104 | | 3 | | 11 | | | Up | |
| Pif1 | 3680 | 3.84304104 | | 3 | | 11 | | | Up | |
| Cyp1b1 | 5128 | 3.84304104 | | 3 | | 11 | | | Up | |
| Gm7030 | 899 | 3.83952977 | | 2.99 | | 10.95 | | | Up | |
| Nudt21 | 1111 | 3.77402198 | | 3 | | 10.74 | | | Up | |
| Lgi1 | 4259 | 3.76710534 | | 7 | | 25 | | | Up | |
| Numbl | 2733 | 3.7602368 | | 5.72 | | 20.38 | | | Up | |
| Hspb7 | 2768 | 3.70881265 | | 6 | | 21 | | | Up | |
| Pifo | 956 | 3.70881265 | | 6 | | 21 | | | Up | |
| Ankrd63 | 4861 | 3.67877976 | | 15.59 | | 54 | | | Up | |
| MXLOC_005403 | 6060 | 3.64771452 | | 3 | | 10.28 | | | Up | |
| Dcakd | 1714 | 3.64005506 | | 2.73 | | 9.33 | | | Up | |
| Zmynd15 | 2801 | 3.6251723 | | 5 | | 17 | | | Up | |
| Sphkap | 6512 | 3.6251723 | | 5 | | 17 | | | Up | |
| MXLOC_042376 | 4361 | 3.621361 | | 7.13 | | 24.21 | | | Up | |
| Xirp1 | 5839 | 3.61099345 | | 60 | | 203 | | | Up | |
| MXLOC_082018 | 5393 | 3.56803399 | | 3 | | 10 | | | Up | |
| Ifi27l2b | 1093 | 3.56803399 | | 3 | | 10 | | | Up | |
| Kpna4 | 3726 | 3.56803399 | | 3 | | 10 | | | Up | |
| Stxbp6 | 4325 | 3.56803399 | | 3 | | 10 | | | Up | |
| Bcap29 | 1791 | 3.56803399 | | 3 | | 10 | | | Up | |
| Usp5 | 3176 | 3.56803399 | | 3 | | 10 | | | Up | |
| Phrf1 | 5374 | 3.56803399 | | 3 | | 10 | | | Up | |
| Il1rap | 4418.26 | 3.56731256 | | 4 | | 13.33 | | | Up | |
| MXLOC_075170 | 8061 | 3.5573671 | | 10.84 | | 36 | | | Up | |
| Chst11 | 5519 | 3.55357085 | | 3 | | 9.95 | | | Up | |
| Eya3 | 4979 | 3.54922414 | | 9.08 | | 30.07 | | | Up | |
| Sult6b1 | 2060 | 3.53903485 | | 10 | | 33 | | | Up | |
| Ckb | 1478 | 3.52356356 | | 10.2 | | 33.48 | | | Up | |
| MXLOC_081495 | 10219.52 | 3.51589185 | | 8.99 | | 29.43 | | | Up | |
| MXLOC_063138 | 10605 | 3.51284398 | | 6.07 | | 19.85 | | | Up | |
| MXLOC_034495 | 3897 | 3.51089569 | | 3.06 | | 10 | | | Up | |
| Arhgef2 | 4291.96 | 3.50000593 | | 3.09 | | 10.06 | | | Up | |
| MXLOC_019396 | 6847 | 3.49498224 | | 4 | | 13 | | | Up | |
| Xk | 5043 | 3.49498224 | | 4 | | 13 | | | Up | |
| Abl1 | 7162 | 3.49498224 | | 4 | | 13 | | | Up | |
| Tmem198b | 2335 | 3.49498224 | | 12 | | 39 | | | Up | |
| Tmem25 | 2354 | 3.49392007 | | 4.18 | | 13.58 | | | Up | |
| MXLOC_072607 | 7257 | 3.49365287 | | 3.34 | | 10.85 | | | Up | |
| MXLOC_086730 | 21388 | 3.48609043 | | 10 | | 32.4 | | | Up | |
| MXLOC_084982 | 15322 | 3.48447079 | | 11 | | 35.62 | | | Up | |
| Pik3c2b | 7929 | 3.47607536 | | 4.24 | | 13.69 | | | Up | |
| Slc39a7 | 2393 | 3.46247656 | | 3.09 | | 9.93 | | | Up | |
| Ube2j2 | 3357 | 3.45486694 | | 3.12 | | 10 | | | Up | |
| Dennd1c | 2521 | 3.45024661 | | 5 | | 16 | | | Up | |
| Cdca7l | 2611 | 3.45024661 | | 15 | | 48 | | | Up | |
| Dock4 | 8065 | 3.44994146 | | 5.91 | | 18.91 | | | Up | |
| Prkcg | 2962 | 3.42003283 | | 6 | | 19 | | | Up | |
| Pld5 | 3571 | 3.42003283 | | 6 | | 19 | | | Up | |
| Kbtbd11 | 6972 | 3.40306812 | | 8.17 | | 25.72 | | | Up | |
| MXLOC_039934 | 2615.31 | 3.40086073 | | 6.04 | | 19 | | | Up | |
| Rb1 | 4625 | 3.3982562 | | 7 | | 22 | | | Up | |
| Adcy3 | 4348 | 3.39195392 | | 11.25 | | 35.28 | | | Up | |
| Ctr9 | 4300 | 3.36896265 | | 9 | | 28 | | | Up | |
| MXLOC_061353 | 17637 | 3.32389282 | | 6.36 | | 19.48 | | | Up | |
| MXLOC_012637 | 8125 | 3.31634683 | | 31.88 | | 97.39 | | | Up | |
| MXLOC_049898 | 8003.14 | 3.30647435 | | 15.52 | | 47.25 | | | Up | |
| MXLOC_025691 | 17174 | 3.30438357 | | 4.26 | | 12.96 | | | Up | |
| Fam179b | 6434 | 3.26402781 | | 4 | | 12 | | | Up | |
| Zfp41 | 3535 | 3.26402781 | | 4 | | 12 | | | Up | |
| Id4 | 1660 | 3.26402781 | | 4 | | 12 | | | Up | |
| Rasd2 | 2810 | 3.26402781 | | 4 | | 12 | | | Up | |
| Homer1 | 4287 | 3.26402781 | | 4 | | 12 | | | Up | |
| Slc5a6 | 3218 | 3.26402781 | | 5 | | 15 | | | Up | |
| Gchfr | 629 | 3.26402781 | | 5 | | 15 | | | Up | |
| Ormdl3 | 2023 | 3.26402781 | | 5 | | 15 | | | Up | |
| Vma21 | 4334.97 | 3.26402781 | | 5 | | 15 | | | Up | |
| Sp9 | 2813 | 3.26402781 | | 5 | | 15 | | | Up | |
| Tpt1 | 894 | 3.26402781 | | 5 | | 15 | | | Up | |
| Tbpl1 | 2875 | 3.26402781 | | 60 | | 180 | | | Up | |
| Galnt1 | 3887 | 3.26249343 | | 6.27 | | 18.8 | | | Up | |
| MXLOC_036213 | 1068 | 3.23726147 | | 3.61 | | 10.73 | | | Up | |
| Furin | 4336 | 3.21770174 | | 11.72 | | 34.6 | | | Up | |
| Mink1 | 4862 | 3.21073639 | | 16.94 | | 49.89 | | | Up | |
| MXLOC_079013 | 6924 | 3.20897709 | | 16.58 | | 48.8 | | | Up | |
| Ralgapa1 | 8231 | 3.2052551 | | 7.77 | | 22.84 | | | Up | |
| Fam222b | 3884 | 3.20389641 | | 5.98 | | 17.57 | | | Up | |
| MXLOC_014769 | 3788 | 3.19754496 | | 6 | | 17.59 | | | Up | |
| Psip1 | 3239 | 3.18333179 | | 7.01 | | 20.45 | | | Up | |
| Sfrp2 | 2001 | 3.17523957 | | 11 | | 32 | | | Up | |
| Hist1h2be | 932.99 | 3.1662086 | | 7 | | 20.3 | | | Up | |
| Olfr536 | 3853 | 3.15909297 | | 56 | | 162 | | | Up | |
| Plcb2 | 5076 | 3.14859681 | | 17 | | 49 | | | Up | |
| MXLOC_066502 | 13895 | 3.12596993 | | 11.13 | | 31.83 | | | Up | |
| Myadml2 | 1530.74 | 3.12324915 | | 7 | | 20 | | | Up | |
| Sgsh | 4324 | 3.12324915 | | 14 | | 40 | | | Up | |
| Hist1h1c | 725 | 3.11552039 | | 20 | | 56.99 | | | Up | |
| MXLOC_060406 | 9885 | 3.09910349 | | 6 | | 17 | | | Up | |
| Lrrc10b | 2077 | 3.09910349 | | 6 | | 17 | | | Up | |
| Zic1 | 3350 | 3.09910349 | | 6 | | 17 | | | Up | |
| Slc7a15 | 2350 | 3.09324482 | | 29 | | 82 | | | Up | |
| Phf14 | 7436 | 3.09060454 | | 4 | | 11.3 | | | Up | |
| Eno2 | 2440 | 3.08940469 | | 5.96 | | 16.83 | | | Up | |
| Aph1b | 4558 | 3.07491734 | | 5.99 | | 16.83 | | | Up | |
| Azin1 | 4620 | 3.07353118 | | 6 | | 16.85 | | | Up | |
| MXLOC_047515 | 10624 | 3.0696659 | | 4.81 | | 13.49 | | | Up | |
| Slc23a2 | 6364 | 3.06495646 | | 5 | | 14 | | | Up | |
| Klhl40 | 2455 | 3.06495646 | | 30 | | 84 | | | Up | |
| Lrch3 | 5660 | 3.06150239 | | 42.39 | | 118.55 | | | Up | |
| Rab5a | 2364 | 3.04196518 | | 9 | | 25 | | | Up | |
| Got2 | 2345 | 3.03307337 | | 13 | | 36 | | | Up | |
| MXLOC_004376 | 4175 | 3.02501362 | | 11.95 | | 33 | | | Up | |
| Ddx6 | 6008 | 3.01296604 | | 4.36 | | 11.99 | | | Up | |
| Tmprss2 | 3161 | 3.01296604 | | 8 | | 22 | | | Up | |
| Kifc3 | 2927 | 2.9921393 | | 17.57 | | 47.97 | | | Up | |
| Nhlrc1 | 2294 | 2.98902076 | | 11 | | 30 | | | Up | |
| 1700019O17Rik | 1721 | 2.97524799 | | 7 | | 19 | | | Up | |
| Zfp518b | 6816 | 2.96630103 | | 17 | | 46 | | | Up | |
| Naa35 | 2614 | 2.96002162 | | 10 | | 27 | | | Up | |
| N4bp2l1 | 1906 | 2.94670613 | | 9.09 | | 24.43 | | | Up | |
| Kif5b | 6030 | 2.93361233 | | 20.61 | | 55.14 | | | Up | |
| Il13ra1 | 3716 | 2.9241778 | | 6 | | 16 | | | Up | |
| Olfr569 | 945 | 2.9241778 | | 6 | | 16 | | | Up | |
| Fam20a | 2541 | 2.9241778 | | 15 | | 40 | | | Up | |
| MXLOC_088487 | 10706 | 2.92380368 | | 163.9 | | 437.01 | | | Up | |
| MXLOC_010306 | 4869.49 | 2.91694171 | | 15.97 | | 42.48 | | | Up | |
| Tmco3 | 4533 | 2.90676141 | | 7.27 | | 19.27 | | | Up | |
| Tmem248 | 3669 | 2.87701245 | | 9.56 | | 25.08 | | | Up | |
| Iffo2 | 5336 | 2.85985414 | | 5.84 | | 15.23 | | | Up | |
| Hdgf | 2245 | 2.85112605 | | 5 | | 13 | | | Up | |
| Emx2 | 2598 | 2.85112605 | | 5 | | 13 | | | Up | |
| Tap1 | 2866 | 2.85112605 | | 5 | | 13 | | | Up | |
| MXLOC_048629 | 23221.79 | 2.82497459 | | 23.19 | | 59.75 | | | Up | |
| MXLOC_061475 | 11917 | 2.81924296 | | 7 | | 18 | | | Up | |
| Rnmtl1 | 1492 | 2.81924296 | | 7 | | 18 | | | Up | |
| Hist2h2aa2 | 596 | 2.81503477 | | 19.06 | | 48.94 | | | Up | |
| Lats2 | 5191 | 2.80920681 | | 16 | | 41 | | | Up | |
| Frmd4a | 5816 | 2.8040528 | | 6.56 | | 16.78 | | | Up | |
| Nr2c1 | 3827 | 2.80137671 | | 9 | | 23 | | | Up | |
| Rnf4 | 3091 | 2.80137671 | | 18 | | 46 | | | Up | |
| Nrxn2 | 6660 | 2.79963765 | | 31.75 | | 81.09 | | | Up | |
| Ptprz1 | 8068 | 2.79361037 | | 6.47 | | 16.49 | | | Up | |
| MXLOC_008360 | 5428 | 2.79058155 | | 6.52 | | 16.6 | | | Up | |
| Pip4k2c | 3374 | 2.77721661 | | 73 | | 185 | | | Up | |
| MXLOC_003701 | 14884 | 2.76757797 | | 14.15 | | 35.74 | | | Up | |
| MXLOC_044291 | 2949 | 2.76095027 | | 9 | | 22.68 | | | Up | |
| Hnrnpk | 2980 | 2.73795899 | | 6 | | 15 | | | Up | |
| Anapc11 | 3229 | 2.73795899 | | 6 | | 15 | | | Up | |
| Tm2d2 | 1281 | 2.73795899 | | 6 | | 15 | | | Up | |
| MXLOC_062461 | 14290.68 | 2.71683341 | | 6.58 | | 16.33 | | | Up | |
| Mycbp2 | 15233 | 2.71401371 | | 6.05 | | 15 | | | Up | |
| Lrrc61 | 2681 | 2.7103474 | | 42 | | 104 | | | Up | |
| MXLOC_013139 | 2846 | 2.68246901 | | 10.92 | | 26.78 | | | Up | |
| MXLOC_019800 | 9019 | 2.68183116 | | 5.71 | | 14 | | | Up | |
| MXLOC_092375 | 16278 | 2.68082069 | | 6.12 | | 15 | | | Up | |
| Fads1 | 3458 | 2.65431864 | | 7 | | 17 | | | Up | |
| Cdkn2b | 1393 | 2.65431864 | | 7 | | 17 | | | Up | |
| Arc | 3056.04 | 2.65431864 | | 7 | | 17 | | | Up | |
| MXLOC_007565 | 7858 | 2.64394777 | | 7.05 | | 17.06 | | | Up | |
| MXLOC_012326 | 15129 | 2.64013979 | | 12 | | 29 | | | Up | |
| Ccdc33 | 2925.89 | 2.64013979 | | 12 | | 29 | | | Up | |
| Rxrb | 2621 | 2.6382619 | | 6.89 | | 16.64 | | | Up | |
| Rpl11 | 594 | 2.63191279 | | 6.54 | | 15.76 | | | Up | |
| Gclm | 2072 | 2.62017162 | | 10 | | 24 | | | Up | |
| MXLOC_038010 | 5677 | 2.61985543 | | 30.42 | | 73 | | | Up | |
| MXLOC_002160 | 15077 | 2.59832584 | | 34.25 | | 81.58 | | | Up | |
| Tmem80 | 1799 | 2.58995783 | | 8 | | 19 | | | Up | |
| Prpf38a | 1519 | 2.58995783 | | 8 | | 19 | | | Up | |
| Mal | 2610 | 2.58995783 | | 8 | | 19 | | | Up | |
| Fgfr3 | 3960 | 2.57605003 | | 32.18 | | 76.06 | | | Up | |
| Iqgap2 | 5771 | 2.5681812 | | 14 | | 33 | | | Up | |
| Loxl1 | 3338 | 2.5681812 | | 14 | | 33 | | | Up | |
| Bach2 | 8493 | 2.5605253 | | 8.92 | | 20.97 | | | Up | |
| Ngfrap1 | 892 | 2.53888765 | | 9 | | 21 | | | Up | |
| Cilp | 4153 | 2.53888765 | | 15 | | 35 | | | Up | |
| Col4a4 | 7808 | 2.53888765 | | 15 | | 35 | | | Up | |
| MXLOC_010759 | 4318.12 | 2.53888765 | | 306 | | 714 | | | Up | |
| MXLOC_044174 | 6542.23 | 2.52846636 | | 19.02 | | 44.22 | | | Up | |
| Tmprss9 | 3454 | 2.51300954 | | 16 | | 37 | | | Up | |
| MXLOC_021063 | 6661 | 2.51210143 | | 7.73 | | 17.87 | | | Up | |
| MXLOC_035642 | 9905 | 2.51204978 | | 36.57 | | 84.54 | | | Up | |
| Mybpc3 | 4150 | 2.50700456 | | 13 | | 30 | | | Up | |
| Pik3r3 | 5004 | 2.49737053 | | 10 | | 23 | | | Up | |
| Phf11d | 2686 | 2.49166253 | | 11 | | 25.25 | | | Up | |
| Fut8 | 2106 | 2.47984414 | | 11.99 | | 27.41 | | | Up | |
| Pik3r2 | 3159 | 2.47939296 | | 7 | | 16 | | | Up | |
| Olfr566 | 951 | 2.47939296 | | 7 | | 16 | | | Up | |
| Trim30b | 2771 | 2.47939296 | | 7 | | 16 | | | Up | |
| Tmem215 | 3218 | 2.47939296 | | 7 | | 16 | | | Up | |
| Acox2 | 2448 | 2.47939296 | | 7 | | 16 | | | Up | |
| Sytl3 | 1504 | 2.47939296 | | 7 | | 16 | | | Up | |
| Phf19 | 3743 | 2.47939296 | | 7 | | 16 | | | Up | |
| Scaf4 | 4177 | 2.46335594 | | 8.57 | | 19.48 | | | Up | |
| MXLOC_072209 | 3100.17 | 2.46295195 | | 44 | | 100 | | | Up | |
| Cttnbp2nl | 4830.01 | 2.46054645 | | 48 | | 109 | | | Up | |
| Pdk4 | 3453 | 2.45381408 | | 13.03 | | 29.52 | | | Up | |
| Nfe2l3 | 2544 | 2.4513647 | | 41.12 | | 93.08 | | | Up | |
| Eif4enif1 | 3679 | 2.4455485 | | 7.45 | | 16.83 | | | Up | |
| MXLOC_006450 | 7403 | 2.43527729 | | 48.4 | | 108.95 | | | Up | |
| Ankrd23 | 2305.05 | 2.43267184 | | 7.51 | | 16.89 | | | Up | |
| MXLOC_013654 | 7722.14 | 2.43180033 | | 11.92 | | 26.8 | | | Up | |
| Capn12 | 2828 | 2.42473549 | | 25.78 | | 57.82 | | | Up | |
| MXLOC_084319 | 13872 | 2.41813441 | | 13.21 | | 29.56 | | | Up | |
| Tmx2 | 2287 | 2.41787817 | | 9.6 | | 21.48 | | | Up | |
| Ubqln1 | 3602 | 2.41050289 | | 7.55 | | 16.85 | | | Up | |
| Tep1 | 8163 | 2.40918536 | | 13 | | 29 | | | Up | |
| Hrnr | 10658 | 2.40918536 | | 13 | | 29 | | | Up | |
| Zfp882 | 5651 | 2.40599547 | | 14.98 | | 33.38 | | | Up | |
| MXLOC_064342 | 7257 | 2.40557995 | | 12.15 | | 27.07 | | | Up | |
| Fam196b | 5371 | 2.40171348 | | 80 | | 178 | | | Up | |
| Mfsd8 | 3051 | 2.40098575 | | 12.03 | | 26.76 | | | Up | |
| Rbm20 | 6592 | 2.4006895 | | 19 | | 42.26 | | | Up | |
| Fpgs | 2263 | 2.39810899 | | 9 | | 20 | | | Up | |
| Npc2 | 3262 | 2.39810899 | | 9 | | 20 | | | Up | |
| Slc6a9 | 3272 | 2.38778558 | | 28 | | 62 | | | Up | |
| Fnip2 | 7095 | 2.38228125 | | 13.71 | | 30.3 | | | Up | |
| Selplg | 2604 | 2.36910985 | | 10 | | 22 | | | Up | |
| Sox1 | 4037 | 2.36910985 | | 10 | | 22 | | | Up | |
| Faah | 3816 | 2.36910985 | | 10 | | 22 | | | Up | |
| Nub1 | 3265 | 2.36910985 | | 20 | | 44 | | | Up | |
| Fam84b | 5563 | 2.36730382 | | 10.17 | | 22.36 | | | Up | |
| Tef | 4244 | 2.36145223 | | 17.15 | | 37.63 | | | Up | |
| Man2a2 | 6554 | 2.34529898 | | 62.61 | | 136.61 | | | Up | |
| Klhdc7a | 5854 | 2.34516457 | | 11 | | 24 | | | Up | |
| Clybl | 1241 | 2.34516457 | | 11 | | 24 | | | Up | |
| Fbxo7 | 1886 | 2.3395984 | | 10.81 | | 23.54 | | | Up | |
| Caprin1 | 4866.67 | 2.33158703 | | 21.39 | | 46.45 | | | Up | |
| MXLOC_013672 | 10088 | 2.31616543 | | 25 | | 54 | | | Up | |
| Wfikkn2 | 3392 | 2.31335179 | | 19 | | 41 | | | Up | |
| Bap1 | 3406 | 2.30793321 | | 13 | | 28 | | | Up | |
| Adam1a | 3092 | 2.30517803 | | 18.71 | | 40.26 | | | Up | |
| Tmem132a | 3489 | 2.30277612 | | 20 | | 43 | | | Up | |
| Sprtn | 2116 | 2.29317415 | | 14 | | 30 | | | Up | |
| Usp31 | 10198 | 2.28307654 | | 9.16 | | 19.56 | | | Up | |
| Ash2l | 3235.23 | 2.27070479 | | 9.11 | | 19.37 | | | Up | |
| MXLOC_079243 | 7161 | 2.26902849 | | 16 | | 34 | | | Up | |
| 1700037H04Rik | 1241 | 2.26902849 | | 24 | | 51 | | | Up | |
| MXLOC_014845 | 4156 | 2.26223133 | | 25 | | 53 | | | Up | |
| Cbx2 | 3753 | 2.25355719 | | 44 | | 93 | | | Up | |
| Onecut1 | 1823 | 2.25010783 | | 9 | | 19 | | | Up | |
| Ccdc80 | 3648 | 2.25010783 | | 9 | | 19 | | | Up | |
| Otud7a | 3461 | 2.2407951 | | 17.64 | | 37.12 | | | Up | |
| MXLOC_000387 | 1434 | 2.23988244 | | 19.49 | | 41 | | | Up | |
| Marcksl1 | 1601 | 2.23488146 | | 10 | | 21 | | | Up | |
| Fam212a | 1061 | 2.23488146 | | 20 | | 42 | | | Up | |
| Wsb2 | 2408 | 2.23488146 | | 20 | | 42 | | | Up | |
| Plpp5 | 1472 | 2.2231057 | | 18.59 | | 38.88 | | | Up | |
| Cd34 | 2529.22 | 2.22236348 | | 11 | | 23 | | | Up | |
| Fxr1 | 2065 | 2.22236348 | | 11 | | 23 | | | Up | |
| Pfn1 | 870 | 2.2209086 | | 10.35 | | 21.63 | | | Up | |
| 2700060E02Rik | 1510 | 2.21690389 | | 23 | | 48 | | | Up | |
| Gm7694 | 3833 | 2.21189018 | | 12 | | 25 | | | Up | |
| Lin7a | 6604 | 2.21189018 | | 12 | | 25 | | | Up | |
| Otud7b | 8052.57 | 2.2100169 | | 11.71 | | 24.38 | | | Up | |
| Hltf | 4956 | 2.20299837 | | 13 | | 27 | | | Up | |
| AU019823 | 3316 | 2.19446854 | | 21.33 | | 44.17 | | | Up | |
| Prune2 | 12512 | 2.18496566 | | 58.14 | | 120 | | | Up | |
| Nol4 | 5028 | 2.18434574 | | 19.83 | | 40.92 | | | Up | |
| MXLOC_031698 | 3418 | 2.18373544 | | 15.53 | | 32.04 | | | Up | |
| Ddx5 | 3520 | 2.1739724 | | 12.47 | | 25.64 | | | Up | |
| Coro1c | 3436 | 2.1710511 | | 37 | | 76 | | | Up | |
| Dnajc14 | 4229 | 2.17045363 | | 62.28 | | 127.9 | | | Up | |
| MXLOC_019753 | 7103.87 | 2.15151618 | | 30.85 | | 62.94 | | | Up | |
| MXLOC_064944 | 9679 | 2.15082022 | | 115.61 | | 235.81 | | | Up | |
| Il7r | 3210 | 2.14342691 | | 29 | | 59 | | | Up | |
| Tctn1 | 4070 | 2.14174198 | | 121.94 | | 247.94 | | | Up | |
| Tenm2 | 9656 | 2.14016218 | | 12.74 | | 25.89 | | | Up | |
| MXLOC_025849 | 3048 | 2.12373492 | | 150.64 | | 304.39 | | | Up | |
| Scai | 10704 | 2.12313213 | | 28.68 | | 57.94 | | | Up | |
| Gspt1 | 6822.4 | 2.11889529 | | 59.1 | | 119.22 | | | Up | |
| MXLOC_082765 | 10553.52 | 2.10415644 | | 22.92 | | 46 | | | Up | |
| MXLOC_069586 | 6595.34 | 2.10235267 | | 13.97 | | 28.02 | | | Up | |
| Elp4 | 6755 | 2.10047132 | | 15.84 | | 31.75 | | | Up | |
| MXLOC_076231 | 3961 | 2.0941028 | | 11 | | 22 | | | Up | |
| Foxh1 | 1366 | 2.0941028 | | 11 | | 22 | | | Up | |
| Sarm1 | 5044 | 2.0941028 | | 12 | | 24 | | | Up | |
| Entpd4 | 3084 | 2.09094984 | | 50.36 | | 100.61 | | | Up | |
| MXLOC_067388 | 5838 | 2.08746718 | | 13.06 | | 26.06 | | | Up | |
| Focad | 5796 | 2.08450083 | | 12.04 | | 24 | | | Up | |
| MXLOC_077671 | 1096 | 2.08433836 | | 13.32 | | 26.55 | | | Up | |
| 4932411N23Rik | 3634 | 2.0809575 | | 11 | | 21.9 | | | Up | |
| MXLOC_059159 | 1885 | 2.07924877 | | 15.58 | | 31 | | | Up | |
| Fam187a | 1563 | 2.07111153 | | 63 | | 125 | | | Up | |
| MXLOC_090891 | 12190 | 2.06719131 | | 72.71 | | 144.07 | | | Up | |
| Rnf225 | 3239 | 2.05780811 | | 40 | | 79 | | | Up | |
| Mfap1b | 1887 | 2.05746749 | | 18.23 | | 36 | | | Up | |
| Gpr85 | 2951 | 2.04658175 | | 25.1 | | 49.38 | | | Up | |
| Slc35e2 | 6291 | 2.04196268 | | 101.63 | | 199.62 | | | Up | |
| Rp2 | 4422 | 2.03842606 | | 11.25 | | 22.07 | | | Up | |
| Slco2a1 | 4033 | 2.03581011 | | 25 | | 49 | | | Up | |
| Gpsm3 | 1343 | 2.02457197 | | 21 | | 41 | | | Up | |
| Apbb1 | 2730 | 2.01441084 | | 20.19 | | 39.28 | | | Up | |
| Supt7l | 2677 | 2.0091911 | | 15.69 | | 30.47 | | | Up | |
| MXLOC_083023 | 3003 | 2.00321686 | | 12.9 | | 25 | | | Up | |
| Hipk2 | 4099 | 2.00296031 | | 40.04 | | 77.59 | | | Up | |
| Eppk1 | 12325 | 2.00249543 | | 16 | | 31 | | | Up | |
| Glud1 | 3158 | -2.0121199 | | 24.9 | | 12 | | | Down | |
| C2 | 2644 | -2.0147928 | | 27 | | 13 | | | Down | |
| Mdk | 785 | -2.0236846 | | 25 | | 12 | | | Down | |
| Lat2 | 1636 | -2.0341579 | | 23 | | 11 | | | Down | |
| MXLOC_022559 | 21308 | -2.0567071 | | 57.34 | | 27.21 | | | Down | |
| MXLOC_093029 | 13393 | -2.0572339 | | 20186.08 | | 9577.31 | | | Down | |
| Mapk8ip3 | 5468 | -2.074742 | | 80.58 | | 38 | | | Down | |
| MXLOC_045891 | 6497 | -2.0806704 | | 23.65 | | 11.13 | | | Down | |
| Rtn1 | 2454.38 | -2.0808229 | | 34 | | 16 | | | Down | |
| Adamts8 | 3632 | -2.0839072 | | 117 | | 55 | | | Down | |
| Ywhaq | 2110 | -2.0927293 | | 28.23 | | 13.23 | | | Down | |
| Rhobtb3 | 4937 | -2.0984183 | | 22 | | 10.29 | | | Down | |
| Tomm70a | 3778 | -2.1049685 | | 30 | | 14 | | | Down | |
| Epha1 | 3273 | -2.1106206 | | 73 | | 34 | | | Down | |
| Serpinf1 | 1494 | -2.1368516 | | 26 | | 12 | | | Down | |
| Slc39a6 | 3882 | -2.1368516 | | 26 | | 12 | | | Down | |
| Plagl2 | 5407 | -2.1368516 | | 39 | | 18 | | | Down | |
| Olfr33 | 957 | -2.1435926 | | 50.71 | | 23.35 | | | Down | |
| Gpr150 | 2146 | -2.1444951 | | 63 | | 29 | | | Down | |
| Ptprs | 6859 | -2.1546789 | | 23 | | 10.55 | | | Down | |
| Cpxm2 | 3506 | -2.156959 | | 24 | | 11 | | | Down | |
| Dcaf12l1 | 3512 | -2.156959 | | 24 | | 11 | | | Down | |
| Ctnnal1 | 3687 | -2.1644632 | | 35 | | 16 | | | Down | |
| Neto1 | 3531 | -2.1683863 | | 46 | | 21 | | | Down | |
| St6galnac5 | 1999 | -2.1809042 | | 22 | | 10 | | | Down | |
| Il17d | 1222 | -2.194677 | | 42 | | 19 | | | Down | |
| Tymp | 2035 | -2.1957467 | | 39.76 | | 17.98 | | | Down | |
| MXLOC_027561 | 3273 | -2.1976858 | | 28.28 | | 12.78 | | | Down | |
| MXLOC_048867 | 4077 | -2.19958 | | 31 | | 14 | | | Down | |
| H2afb3 | 645 | -2.2099034 | | 20 | | 9 | | | Down | |
| Pip5k1c | 4210 | -2.2099034 | | 20 | | 9 | | | Down | |
| MXLOC_048650 | 8452 | -2.2273249 | | 37.47 | | 16.76 | | | Down | |
| MXLOC_059491 | 8959 | -2.2364296 | | 31.69 | | 14.13 | | | Down | |
| MXLOC_001481 | 3340 | -2.2504671 | | 35.18 | | 15.61 | | | Down | |
| Kif1a | 8243 | -2.2724994 | | 45.51 | | 20.04 | | | Down | |
| Chd3 | 7281 | -2.2752852 | | 58.42 | | 25.7 | | | Down | |
| MXLOC_016421 | 6384.11 | -2.297242 | | 49.75 | | 21.72 | | | Down | |
| Vps37b | 2512 | -2.3085046 | | 43.69 | | 19 | | | Down | |
| Cgn | 5014 | -2.3091649 | | 23 | | 10 | | | Down | |
| Rcc2 | 3767 | -2.3091649 | | 23 | | 10 | | | Down | |
| Ccnk | 2632 | -2.3091649 | | 23 | | 10 | | | Down | |
| MXLOC_018318 | 5440 | -2.3190846 | | 23.31 | | 10.1 | | | Down | |
| Ncf1 | 2722 | -2.326545 | | 18.65 | | 8.06 | | | Down | |
| MXLOC_069832 | 18445.77 | -2.345525 | | 27.95 | | 12 | | | Down | |
| Hpcal4 | 4652 | -2.350682 | | 21 | | 9 | | | Down | |
| Ribc1 | 1506 | -2.350682 | | 28 | | 12 | | | Down | |
| MXLOC_072997 | 7075 | -2.350682 | | 35 | | 15 | | | Down | |
| Celf4 | 2258 | -2.3772268 | | 25.48 | | 10.82 | | | Down | |
| Plagl1 | 5261 | -2.3879134 | | 78 | | 33 | | | Down | |
| Lemd2 | 2596 | -2.4017522 | | 19 | | 8 | | | Down | |
| Cacnb3 | 2568 | -2.4017522 | | 19 | | 8 | | | Down | |
| Cldn19 | 4236 | -2.4017522 | | 19 | | 8 | | | Down | |
| Ddx23 | 2814 | -2.4017522 | | 19 | | 8 | | | Down | |
| Mfap4 | 1513 | -2.431966 | | 24 | | 10 | | | Down | |
| MXLOC_069385 | 2985 | -2.4439048 | | 34.39 | | 14.27 | | | Down | |
| Ppm1a | 2789 | -2.466113 | | 17 | | 7 | | | Down | |
| MXLOC_071002 | 6657 | -2.4733629 | | 31.31 | | 12.86 | | | Down | |
| Prkd3 | 5083 | -2.473784 | | 16.68 | | 6.85 | | | Down | |
| Slc22a8 | 3307 | -2.4796595 | | 61 | | 25 | | | Down | |
| MXLOC_064709 | 9604 | -2.4849104 | | 22 | | 9 | | | Down | |
| Tdrd6 | 7059.42 | -2.4849104 | | 22 | | 9 | | | Down | |
| Ccdc60 | 2582 | -2.4849104 | | 22 | | 9 | | | Down | |
| Swt1 | 3668 | -2.4849104 | | 22 | | 9 | | | Down | |
| Polr2b | 3812 | -2.4849104 | | 22 | | 9 | | | Down | |
| Dcc | 10325 | -2.5248639 | | 16.16 | | 6.52 | | | Down | |
| Pcdhga7 | 4659 | -2.5360893 | | 15.8 | | 6.35 | | | Down | |
| Otud4 | 7324.45 | -2.5383911 | | 22.81 | | 9.16 | | | Down | |
| Ecd | 3125 | -2.5497534 | | 20 | | 8 | | | Down | |
| Slc25a1 | 1678 | -2.5497534 | | 20 | | 8 | | | Down | |
| 9430016H08Rik | 1325 | -2.5497534 | | 20 | | 8 | | | Down | |
| Tmem179b | 848 | -2.5592252 | | 35.09 | | 13.99 | | | Down | |
| MXLOC_084391 | 3659 | -2.5850337 | | 43.2 | | 17.07 | | | Down | |
| MXLOC_059060 | 5415 | -2.6127638 | | 31.48 | | 12.32 | | | Down | |
| Coq5 | 2001 | -2.6131711 | | 46 | | 18 | | | Down | |
| Prx | 5260 | -2.617543 | | 107.88 | | 42.15 | | | Down | |
| Erdr1 | 777 | -2.6191714 | | 93.6 | | 36.55 | | | Down | |
| MXLOC_036804 | 1697 | -2.6310374 | | 18 | | 7 | | | Down | |
| Slc4a5 | 5124 | -2.6310374 | | 18 | | 7 | | | Down | |
| Zkscan6 | 2268.02 | -2.6588594 | | 18.46 | | 7.11 | | | Down | |
| Prkaca | 2290 | -2.6666173 | | 15.62 | | 6 | | | Down | |
| Zfp773 | 3025 | -2.6718217 | | 18.83 | | 7.22 | | | Down | |
| Fam208a | 7576 | -2.6917497 | | 32.59 | | 12.41 | | | Down | |
| Efhd2 | 2381 | -2.7359722 | | 16 | | 6 | | | Down | |
| Ncstn | 2855 | -2.7359722 | | 16 | | 6 | | | Down | |
| MXLOC_078159 | 8984 | -2.7511871 | | 26.62 | | 9.93 | | | Down | |
| Dnajc5 | 4325 | -2.7609738 | | 17 | | 6.32 | | | Down | |
| MXLOC_001028 | 2314 | -2.7642626 | | 22.89 | | 8.5 | | | Down | |
| MXLOC_093668 | 7316 | -2.7834632 | | 13309.51 | | 4909.59 | | | Down | |
| Tomm20l | 596 | -2.7870424 | | 19 | | 7 | | | Down | |
| Trcg1 | 2701 | -2.8150289 | | 74 | | 27 | | | Down | |
| MXLOC_012065 | 5214 | -2.8239866 | | 65.24 | | 23.73 | | | Down | |
| Dnm3 | 7519 | -2.8282871 | | 18.42 | | 6.69 | | | Down | |
| MXLOC_013353 | 9088 | -2.8400548 | | 35.94 | | 13 | | | Down | |
| Podnl1 | 2080 | -2.8537596 | | 50 | | 18 | | | Down | |
| Snhg11 | 5864 | -2.858596 | | 21.23 | | 7.63 | | | Down | |
| MXLOC_057262 | 6371 | -2.8752677 | | 19.45 | | 6.95 | | | Down | |
| Eftud2 | 3330.06 | -2.8759313 | | 14.08 | | 5.03 | | | Down | |
| Alkbh2 | 1065 | -2.8767509 | | 14 | | 5 | | | Down | |
| Ankrd34b | 3940 | -2.8767509 | | 14 | | 5 | | | Down | |
| Pgrmc1 | 1870 | -2.8767509 | | 14 | | 5 | | | Down | |
| Ncoa1 | 7329 | -2.8767509 | | 14 | | 5 | | | Down | |
| Fbxo42 | 5925 | -2.8767509 | | 14 | | 5 | | | Down | |
| Wisp1 | 5022 | -2.8767509 | | 14 | | 5 | | | Down | |
| MXLOC_012544 | 29401 | -2.8776059 | | 27 | | 9.64 | | | Down | |
| Tacr1 | 5026 | -2.9016693 | | 19.77 | | 7 | | | Down | |
| Mrpl42 | 666 | -2.9108979 | | 17 | | 6 | | | Down | |
| MXLOC_049079 | 4070 | -2.9116683 | | 137.34 | | 48.46 | | | Down | |
| Pcm1 | 8381 | -2.913148 | | 21.38 | | 7.54 | | | Down | |
| Colec10 | 4587 | -2.9350435 | | 20 | | 7 | | | Down | |
| Ap4m1 | 1716 | -2.9350435 | | 20 | | 7 | | | Down | |
| Sfrp5 | 1900 | -2.9350435 | | 20 | | 7 | | | Down | |
| MXLOC_067550 | 2140 | -2.9633666 | | 23.4 | | 8.11 | | | Down | |
| Chl1 | 7751 | -2.9645802 | | 25.95 | | 8.99 | | | Down | |
| MXLOC_009890 | 4013 | -2.9807276 | | 21.19 | | 7.3 | | | Down | |
| Zdbf2 | 12648 | -2.991021 | | 36.21 | | 12.43 | | | Down | |
| Brd4 | 5948 | -2.9910528 | | 12.41 | | 4.26 | | | Down | |
| Rps28 | 327 | -2.9944216 | | 20.62 | | 7.07 | | | Down | |
| Nol10 | 2986 | -3.016299 | | 20.63 | | 7.02 | | | Down | |
| Phactr1 | 4656.93 | -3.0379457 | | 18.92 | | 6.39 | | | Down | |
| Nolc1 | 3659.18 | -3.0758222 | | 12 | | 4 | | | Down | |
| E230025N22Rik | 2016 | -3.0758222 | | 12 | | 4 | | | Down | |
| Ostc | 1062 | -3.0758222 | | 12 | | 4 | | | Down | |
| Cmtm7 | 1038 | -3.0758222 | | 12 | | 4 | | | Down | |
| Stambpl1 | 1978 | -3.0758222 | | 12 | | 4 | | | Down | |
| Ldlrad3 | 3907.11 | -3.0758222 | | 15 | | 5 | | | Down | |
| Cops3 | 1566 | -3.0758222 | | 15 | | 5 | | | Down | |
| MXLOC_088713 | 18592 | -3.0758222 | | 20.97 | | 6.99 | | | Down | |
| Pi16 | 2245 | -3.0758222 | | 21 | | 7 | | | Down | |
| Lrp1b | 14743 | -3.0758222 | | 27 | | 9 | | | Down | |
| Usp27x | 3240 | -3.0758222 | | 33 | | 11 | | | Down | |
| 1700030K09Rik | 2427 | -3.0963589 | | 21.15 | | 7 | | | Down | |
| MXLOC_026889 | 7944 | -3.1230313 | | 17.87 | | 5.86 | | | Down | |
| Camkk2 | 4846 | -3.1555129 | | 11.38 | | 3.69 | | | Down | |
| Inafm2 | 3053 | -3.1704336 | | 31 | | 10 | | | Down | |
| Atrx | 10244 | -3.1781796 | | 18.65 | | 6 | | | Down | |
| Gpld1 | 4659 | -3.1794499 | | 301.61 | | 96.99 | | | Down | |
| Abi2 | 5773 | -3.1827898 | | 48.91 | | 15.71 | | | Down | |
| MXLOC_073833 | 6563 | -3.1875564 | | 13.69 | | 4.39 | | | Down | |
| MXLOC_008457 | 7416 | -3.2107191 | | 37 | | 11.77 | | | Down | |
| MXLOC_057600 | 15578 | -3.2305842 | | 19.34 | | 6.11 | | | Down | |
| Sgcz | 2389 | -3.2318272 | | 19 | | 6 | | | Down | |
| Brinp1 | 3354 | -3.2318272 | | 19 | | 6 | | | Down | |
| Hmga1-rs1 | 1566 | -3.2614334 | | 56.98 | | 17.81 | | | Down | |
| Uchl1 | 1147 | -3.262041 | | 16 | | 5 | | | Down | |
| Smurf2 | 5331 | -3.2641251 | | 11.08 | | 3.46 | | | Down | |
| 9130019O22Rik | 3878 | -3.2996654 | | 16.21 | | 5 | | | Down | |
| MXLOC_072127 | 9379 | -3.2999819 | | 28.63 | | 8.83 | | | Down | |
| Wipf2 | 7589 | -3.3053626 | | 25.5 | | 7.85 | | | Down | |
| P3h1 | 3013 | -3.3067766 | | 13 | | 4 | | | Down | |
| 4930433I11Rik | 2022 | -3.3067766 | | 13 | | 4 | | | Down | |
| Col6a1 | 3976 | -3.3067766 | | 13 | | 4 | | | Down | |
| Mex3c | 4080 | -3.3067766 | | 13 | | 4 | | | Down | |
| Fv1 | 1380 | -3.3067766 | | 13 | | 4 | | | Down | |
| Tanc2 | 11859 | -3.3173938 | | 40.84 | | 12.52 | | | Down | |
| Fscn1 | 2667 | -3.3508292 | | 33 | | 10 | | | Down | |
| Slc16a10 | 2313 | -3.3873375 | | 10.26 | | 3.07 | | | Down | |
| MXLOC_014499 | 6973 | -3.3925372 | | 19.72 | | 5.89 | | | Down | |
| Csf2rb2 | 4412 | -3.3983425 | | 22.41 | | 6.68 | | | Down | |
| Limk1 | 3110 | -3.4156722 | | 27 | | 8 | | | Down | |
| Dock5 | 10335 | -3.4251352 | | 19.47 | | 5.75 | | | Down | |
| Prom2 | 4224 | -3.4369667 | | 17 | | 5 | | | Down | |
| Phf1 | 2444.03 | -3.4369667 | | 17 | | 5 | | | Down | |
| D17Wsu92e | 3812 | -3.4482598 | | 10.24 | | 3 | | | Down | |
| Nudt16l1 | 1277 | -3.4675804 | | 19.14 | | 5.57 | | | Down | |
| MXLOC_072840 | 5703 | -3.5007577 | | 26.8 | | 7.71 | | | Down | |
| MXLOC_089009 | 7821 | -3.5156957 | | 11.74 | | 3.36 | | | Down | |
| Atp6v1c2 | 1567 | -3.520607 | | 14 | | 4 | | | Down | |
| Piwil1 | 3944 | -3.520607 | | 21 | | 6 | | | Down | |
| MXLOC_063106 | 4975 | -3.5250169 | | 32.74 | | 9.34 | | | Down | |
| MXLOC_081269 | 7090 | -3.539535 | | 26 | | 7.38 | | | Down | |
| MXLOC_077796 | 4605.24 | -3.5522908 | | 29.76 | | 8.41 | | | Down | |
| MXLOC_076201 | 6785 | -3.5547541 | | 18.7 | | 5.28 | | | Down | |
| MXLOC_075614 | 4549 | -3.583902 | | 31.34 | | 8.76 | | | Down | |
| Akap13 | 12543 | -3.6023326 | | 13.07 | | 3.63 | | | Down | |
| Ccl21a | 868 | -3.6112748 | | 202.04 | | 55.94 | | | Down | |
| Eng | 3572 | -3.6121427 | | 12.97 | | 3.59 | | | Down | |
| MXLOC_009257 | 24789 | -3.6218592 | | 19.43 | | 5.36 | | | Down | |
| Clasp1 | 7599 | -3.6286867 | | 9.52 | | 2.62 | | | Down | |
| MXLOC_068033 | 426 | -3.6384586 | | 9.37 | | 2.57 | | | Down | |
| Syncrip | 6773 | -3.6520135 | | 10.22 | | 2.79 | | | Down | |
| MXLOC_091843 | 5847 | -3.6548354 | | 11 | | 3 | | | Down | |
| MXLOC_037355 | 4729 | -3.6548354 | | 11 | | 3 | | | Down | |
| 1110065P20Rik | 762 | -3.6548354 | | 11 | | 3 | | | Down | |
| Prdx4 | 948 | -3.6548354 | | 11 | | 3 | | | Down | |
| Ufm1 | 4867 | -3.6548354 | | 11 | | 3 | | | Down | |
| Idh2 | 1702 | -3.6548354 | | 11 | | 3 | | | Down | |
| Amph | 3219 | -3.6548354 | | 11 | | 3 | | | Down | |
| Slc27a2 | 2301 | -3.6548354 | | 11 | | 3 | | | Down | |
| MXLOC_072511 | 6248 | -3.6684651 | | 10.61 | | 2.88 | | | Down | |
| MXLOC_031629 | 6141 | -3.6781613 | | 20.7 | | 5.6 | | | Down | |
| MXLOC_015627 | 13126 | -3.6782058 | | 23.14 | | 6.26 | | | Down | |
| Wdr37 | 4555 | -3.6920668 | | 26 | | 7 | | | Down | |
| Erc2 | 6083 | -3.6957331 | | 18 | | 4.84 | | | Down | |
| Aff3 | 7251 | -3.6965024 | | 9.3 | | 2.5 | | | Down | |
| MXLOC_063165 | 4191 | -3.7000974 | | 66.82 | | 17.94 | | | Down | |
| MXLOC_038043 | 4692 | -3.7196784 | | 15 | | 4 | | | Down | |
| Gldn | 4649 | -3.7196784 | | 15 | | 4 | | | Down | |
| Wnk3 | 10400 | -3.7423413 | | 35.15 | | 9.3 | | | Down | |
| Fbxo10 | 4634 | -3.757896 | | 19 | | 5 | | | Down | |
| MXLOC_073319 | 4457 | -3.7817958 | | 14.79 | | 3.86 | | | Down | |
| Tmem115 | 2177 | -3.7830961 | | 23 | | 6 | | | Down | |
| MXLOC_023487 | 10410 | -3.7864847 | | 9.94 | | 2.59 | | | Down | |
| Slc31a2 | 1803 | -3.8006735 | | 14.27 | | 3.7 | | | Down | |
| MXLOC_023741 | 2335 | -3.8009624 | | 54 | | 14 | | | Down | |
| MXLOC_034385 | 15287 | -3.8368418 | | 28.9 | | 7.4 | | | Down | |
| Car5b | 3449 | -3.8638762 | | 32.72 | | 8.3 | | | Down | |
| MXLOC_041183 | 7861 | -3.881985 | | 28.84 | | 7.27 | | | Down | |
| MXLOC_007437 | 2443 | -3.9058972 | | 12 | | 3 | | | Down | |
| Chst5 | 1935 | -3.9058972 | | 12 | | 3 | | | Down | |
| Rab11fip4 | 3145 | -3.9058972 | | 12 | | 3 | | | Down | |
| Lrp4 | 8028 | -3.9058972 | | 12 | | 3 | | | Down | |
| Olfr31 | 954 | -3.9058972 | | 12 | | 3 | | | Down | |
| Hes7 | 964 | -3.9058972 | | 16 | | 4 | | | Down | |
| A730008H23Rik | 2217 | -3.9191634 | | 8.72 | | 2.17 | | | Down | |
| Pitpnm3 | 6618 | -4.0121199 | | 8.3 | | 2 | | | Down | |
| Ppp2r3c | 2395 | -4.0425092 | | 13.84 | | 3.3 | | | Down | |
| Atp5g2 | 647 | -4.0808229 | | 17 | | 4 | | | Down | |
| Ccnl1 | 2145 | -4.0821451 | | 49.11 | | 11.55 | | | Down | |
| Yes1 | 2432 | -4.106648 | | 9.82 | | 2.29 | | | Down | |
| Mtcl1 | 7221 | -4.1078525 | | 12.87 | | 3 | | | Down | |
| Rapgef6 | 2827 | -4.113248 | | 17.45 | | 4.06 | | | Down | |
| Ndufs3 | 940 | -4.1368516 | | 13 | | 3 | | | Down | |
| Reep5 | 2897 | -4.1368516 | | 13 | | 3 | | | Down | |
| MXLOC_047016 | 9040 | -4.1751122 | | 14.93 | | 3.4 | | | Down | |
| Ppara | 7207 | -4.2087206 | | 13.55 | | 3.05 | | | Down | |
| Ankzf1 | 1204 | -4.2133267 | | 15.93 | | 3.58 | | | Down | |
| Pcdha4 | 5322 | -4.2275181 | | 22.09 | | 4.94 | | | Down | |
| MXLOC_082697 | 3389 | -4.2457472 | | 9 | | 2 | | | Down | |
| Cpne2 | 2302 | -4.2457472 | | 9 | | 2 | | | Down | |
| Arl4c | 3966 | -4.2457472 | | 9 | | 2 | | | Down | |
| Trim45 | 3973.06 | -4.2457472 | | 9 | | 2 | | | Down | |
| C1qbp | 1165 | -4.2457472 | | 9 | | 2 | | | Down | |
| Gpr156 | 4583 | -4.2457472 | | 9 | | 2 | | | Down | |
| Atp4a | 3470 | -4.2457472 | | 9 | | 2 | | | Down | |
| Cpne7 | 2405 | -4.2457472 | | 9 | | 2 | | | Down | |
| Capn10 | 2877 | -4.2457472 | | 9 | | 2 | | | Down | |
| Slc22a6 | 4026 | -4.2457472 | | 18 | | 4 | | | Down | |
| Vstm5 | 1988 | -4.2457472 | | 18 | | 4 | | | Down | |
| MXLOC_044048 | 12174 | -4.2816315 | | 26.7 | | 5.86 | | | Down | |
| MXLOC_031930 | 13407 | -4.2871269 | | 9.13 | | 2 | | | Down | |
| Nudt2 | 848 | -4.3091649 | | 23 | | 5 | | | Down | |
| Synrg | 7110 | -4.3239523 | | 71.99 | | 15.57 | | | Down | |
| MXLOC_083246 | 3563 | -4.3482519 | | 51.43 | | 11.03 | | | Down | |
| Bcas1 | 2952 | -4.350682 | | 14 | | 3 | | | Down | |
| MXLOC_039418 | 13322.41 | -4.3619953 | | 9.37 | | 2 | | | Down | |
| MXLOC_007257 | 5130 | -4.3758565 | | 33 | | 7.01 | | | Down | |
| Slc22a17 | 2294 | -4.4017522 | | 19 | | 4 | | | Down | |
| Dis3 | 3722 | -4.4017522 | | 19 | | 4 | | | Down | |
| MXLOC_088452 | 3999 | -4.4168987 | | 9.55 | | 2 | | | Down | |
| MXLOC_035911 | 1833 | -4.5497534 | | 10 | | 2 | | | Down | |
| Lrp12 | 4064 | -4.5497534 | | 10 | | 2 | | | Down | |
| Epas1 | 5352 | -4.5497534 | | 10 | | 2 | | | Down | |
| Nlk | 4496 | -4.5497534 | | 10 | | 2 | | | Down | |
| Psd | 3900 | -4.6597479 | | 9.09 | | 1.75 | | | Down | |
| MXLOC_078152 | 2832 | -4.7074848 | | 31.58 | | 5.98 | | | Down | |
| MXLOC_040991 | 6030 | -4.7430356 | | 24.54 | | 4.59 | | | Down | |
| MXLOC_015183 | 12544 | -4.7635158 | | 32.63 | | 6.06 | | | Down | |
| MXLOC_073694 | 5245 | -4.8247604 | | 11 | | 2 | | | Down | |
| Szt2 | 10958 | -4.8247604 | | 11 | | 2 | | | Down | |
| MXLOC_001900 | 2127 | -4.8767509 | | 28 | | 5 | | | Down | |
| Smap1 | 2083 | -4.8944767 | | 30.37 | | 5.39 | | | Down | |
| MXLOC_086876 | 3712 | -4.9292879 | | 7.87 | | 1.38 | | | Down | |
| MXLOC_073022 | 5158 | -4.9651361 | | 10.74 | | 1.86 | | | Down | |
| MXLOC_065661 | 3784.13 | -4.9846075 | | 18.37 | | 3.16 | | | Down | |
| Slc2a6 | 1972 | -4.9895677 | | 14.5 | | 2.49 | | | Down | |
| Epb41l3 | 4045 | -5.0210406 | | 15.13 | | 2.57 | | | Down | |
| MXLOC_071651 | 4042 | -5.0758222 | | 10.08 | | 1.68 | | | Down | |
| Nr2c2ap | 1191 | -5.1369852 | | 60.55 | | 9.88 | | | Down | |
| Gcat | 1883.17 | -5.1501773 | | 10.22 | | 1.66 | | | Down | |
| MXLOC_015918 | 2931 | -5.1517579 | | 12.32 | | 2 | | | Down | |
| Hist1h2ao | 552 | -5.1677857 | | 13.07 | | 2.11 | | | Down | |
| Cnot3 | 2905 | -5.2206392 | | 8.58 | | 1.36 | | | Down | |
| MXLOC_015467 | 10050 | -5.2348629 | | 6.34 | | 1 | | | Down | |
| Rab11b | 6065 | -5.2688494 | | 48.37 | | 7.54 | | | Down | |
| Rbms3 | 7751 | -5.3067766 | | 13 | | 2 | | | Down | |
| Atp5j | 643 | -5.3595596 | | 6.62 | | 1 | | | Down | |
| Pde4d | 7049 | -5.3650693 | | 13 | | 1.96 | | | Down | |
| MXLOC_091904 | 17861 | -5.3826338 | | 17.15 | | 2.57 | | | Down | |
| Agl | 9625 | -5.4183651 | | 16.08 | | 2.38 | | | Down | |
| Elmo2 | 4522 | -5.4477681 | | 20.34 | | 2.98 | | | Down | |
| MXLOC_077534 | 722 | -5.4979136 | | 21.53 | | 3.1 | | | Down | |
| MXLOC_073240 | 5996 | -5.5052999 | | 11.28 | | 1.62 | | | Down | |
| MXLOC_046490 | 4487 | -5.520607 | | 7 | | 1 | | | Down | |
| Map3k9 | 10106.76 | -5.520607 | | 7 | | 1 | | | Down | |
| AI846148 | 1594 | -5.520607 | | 7 | | 1 | | | Down | |
| Syde2 | 5495 | -5.520607 | | 7 | | 1 | | | Down | |
| Retnlb | 696 | -5.520607 | | 7 | | 1 | | | Down | |
| Lgmn | 1874 | -5.520607 | | 7 | | 1 | | | Down | |
| Prdm1 | 5205 | -5.520607 | | 7 | | 1 | | | Down | |
| Pbx2 | 2801 | -5.520607 | | 7 | | 1 | | | Down | |
| Atxn7l2 | 2452 | -5.520607 | | 7 | | 1 | | | Down | |
| Tshr | 4312 | -5.520607 | | 7 | | 1 | | | Down | |
| Wdr75 | 2612 | -5.520607 | | 7 | | 1 | | | Down | |
| Tti1 | 3772 | -5.520607 | | 7 | | 1 | | | Down | |
| Zfp874b | 3772 | -5.5945955 | | 7.11 | | 0 | | | Down | |
| MXLOC_042512 | 3094 | -5.6007815 | | 20.8 | | 2.89 | | | Down | |
| Aip | 2032.4 | -5.6790451 | | 15.16 | | 2.05 | | | Down | |
| Tacc2 | 9103 | -5.700378 | | 7.45 | | 1 | | | Down | |
| 1700123K08Rik | 1047 | -5.7196784 | | 15 | | 2 | | | Down | |
| Cers3 | 3251 | -5.728168 | | 27.23 | | 3.62 | | | Down | |
| Ppp2r5c | 2599 | -5.8183846 | | 12.34 | | 1.59 | | | Down | |
| Hars2 | 2890 | -5.8206085 | | 8 | | 1.03 | | | Down | |
| Svep1 | 11275 | -5.863568 | | 8.12 | | 1.03 | | | Down | |
| MXLOC_007295 | 7922 | -5.8805389 | | 7.93 | | 1 | | | Down | |
| MXLOC_001101 | 8680 | -5.9058972 | | 8 | | 1 | | | Down | |
| Tax1bp3 | 1417 | -5.9058972 | | 24 | | 3 | | | Down | |
| 9930111J21Rik1 | 3863 | -5.9262026 | | 14.26 | | 1.77 | | | Down | |
| Tnpo1 | 5385.09 | -5.962754 | | 16.4 | | 2.01 | | | Down | |
| Fez1 | 1285 | -6.0151856 | | 5.65 | | 0 | | | Down | |
| MXLOC_069839 | 3639.88 | -6.0808229 | | 7.65 | | 0 | | | Down | |
| Scd1 | 4837 | -6.1464857 | | 8 | | 0 | | | Down | |
| MXLOC_070996 | 4102 | -6.1762852 | | 29.96 | | 3.41 | | | Down | |
| MXLOC_015094 | 8246 | -6.2457472 | | 9 | | 1 | | | Down | |
| Nfu1 | 952 | -6.2457472 | | 9 | | 1 | | | Down | |
| Mthfd2l | 2245 | -6.2457472 | | 9 | | 1 | | | Down | |
| Agtpbp1 | 3061 | -6.3298488 | | 12.88 | | 1.39 | | | Down | |
| MXLOC_084288 | 22248.68 | -6.3619953 | | 9.37 | | 1 | | | Down | |
| Dctn3 | 966 | -6.4017522 | | 19 | | 2 | | | Down | |
| Ckap5 | 6514 | -6.4519342 | | 5.51 | | 0 | | | Down | |
| Dicer1 | 9851 | -6.4926151 | | 5 | | 0 | | | Down | |
| MXLOC_000977 | 1531 | -6.5497534 | | 5 | | 0 | | | Down | |
| MXLOC_015771 | 3502 | -6.5497534 | | 5 | | 0 | | | Down | |
| MXLOC_024412 | 4943 | -6.5497534 | | 5 | | 0 | | | Down | |
| MXLOC_045174 | 4287 | -6.5497534 | | 5 | | 0 | | | Down | |
| Snd1 | 3482 | -6.5497534 | | 5 | | 0 | | | Down | |
| Hist1h4m | 405 | -6.5497534 | | 5 | | 0 | | | Down | |
| Trpm1 | 2928 | -6.5497534 | | 5 | | 0 | | | Down | |
| Smyd3 | 3761 | -6.5497534 | | 5 | | 0 | | | Down | |
| Ptp4a1 | 4129 | -6.5497534 | | 5 | | 0 | | | Down | |
| Tsga10 | 2722 | -6.5497534 | | 5 | | 0 | | | Down | |
| Kbtbd8 | 4521 | -6.5497534 | | 5 | | 0 | | | Down | |
| Sh2d4a | 2767 | -6.5497534 | | 5 | | 0 | | | Down | |
| Tmem194 | 3419.04 | -6.5497534 | | 5 | | 0 | | | Down | |
| Ccrl2 | 1763 | -6.5497534 | | 5 | | 0 | | | Down | |
| Fbxo21 | 3909 | -6.5497534 | | 5 | | 0 | | | Down | |
| Ndufb9 | 651 | -6.5497534 | | 5 | | 0 | | | Down | |
| Med15 | 3105 | -6.5497534 | | 5 | | 0 | | | Down | |
| Slc25a15 | 3455 | -6.5497534 | | 5 | | 0 | | | Down | |
| Utp14b | 3702 | -6.5497534 | | 5 | | 0 | | | Down | |
| Tbl2 | 4336 | -6.5497534 | | 5 | | 0 | | | Down | |
| Srp72 | 3605 | -6.5497534 | | 5 | | 0 | | | Down | |
| Adgra3 | 4475 | -6.5497534 | | 5 | | 0 | | | Down | |
| Tln1 | 8560 | -6.5497534 | | 5 | | 0 | | | Down | |
| Cxx1c | 952 | -6.5497534 | | 5 | | 0 | | | Down | |
| MXLOC_034595 | 4698 | -6.5497534 | | 10 | | 1 | | | Down | |
| Ahcyl2 | 5198.32 | -6.5497534 | | 10 | | 1 | | | Down | |
| Pabpc1l | 2580 | -6.5497534 | | 10 | | 1 | | | Down | |
| Mrpl37 | 1496 | -6.5497534 | | 10 | | 1 | | | Down | |
| Slc25a43 | 1243 | -6.5727447 | | 5.04 | | 0 | | | Down | |
| Sort1 | 6831 | -6.6245262 | | 43 | | 4.19 | | | Down | |
| Dusp1 | 1942 | -6.6406393 | | 5.16 | | 0 | | | Down | |
| Notch1 | 9489 | -6.6573663 | | 5.19 | | 0 | | | Down | |
| Pcdhgb1 | 4498 | -6.6684639 | | 5.21 | | 0 | | | Down | |
| Gpr25 | 1960 | -6.7210397 | | 23.77 | | 2.24 | | | Down | |
| Ifngr2 | 2070 | -6.8247604 | | 11 | | 1 | | | Down | |
| Bfsp2 | 1570 | -6.8247604 | | 11 | | 1 | | | Down | |
| MXLOC_027907 | 11395 | -6.8352337 | | 5.52 | | 0 | | | Down | |
| Taf6l | 2203 | -6.8870375 | | 5.62 | | 0 | | | Down | |
| MXLOC_084676 | 7294 | -6.8972875 | | 5.64 | | 0 | | | Down | |
| MXLOC_008109 | 3603 | -6.9479987 | | 5.74 | | 0 | | | Down | |
| MXLOC_007546 | 7978 | -6.9479987 | | 5.74 | | 0 | | | Down | |
| Bend3 | 6049 | -6.9530211 | | 5.75 | | 0 | | | Down | |
| MXLOC_031498 | 1958 | -7.0027704 | | 5.85 | | 0 | | | Down | |
| Pfkm | 2816 | -7.0468231 | | 5.94 | | 0 | | | Down | |
| MXLOC_011956 | 2787 | -7.0565219 | | 5.96 | | 0 | | | Down | |
| MXLOC_028131 | 6003 | -7.0758222 | | 6 | | 0 | | | Down | |
| MXLOC_075533 | 2877 | -7.0758222 | | 6 | | 0 | | | Down | |
| Gm5622 | 1581 | -7.0758222 | | 6 | | 0 | | | Down | |
| Rcan2 | 3107 | -7.0758222 | | 6 | | 0 | | | Down | |
| Smagp | 965 | -7.0758222 | | 6 | | 0 | | | Down | |
| Lrrc63 | 2519 | -7.0758222 | | 6 | | 0 | | | Down | |
| Ggt5 | 4137 | -7.0758222 | | 6 | | 0 | | | Down | |
| Cox14 | 723 | -7.0758222 | | 6 | | 0 | | | Down | |
| Hiatl1 | 3472 | -7.0758222 | | 6 | | 0 | | | Down | |
| Usp46 | 3225 | -7.0758222 | | 6 | | 0 | | | Down | |
| Ypel5 | 2869 | -7.0758222 | | 6 | | 0 | | | Down | |
| Endov | 5112 | -7.0806272 | | 6.01 | | 0 | | | Down | |
| Cog8 | 2104 | -7.0902132 | | 6.03 | | 0 | | | Down | |
| Ccser1 | 5654 | -7.0949943 | | 6.04 | | 0 | | | Down | |
| Mis12 | 2766 | -7.1140398 | | 6.08 | | 0 | | | Down | |
| Smim15 | 1864 | -7.1615082 | | 17.43 | | 1.41 | | | Down | |
| Mest | 2387 | -7.1750837 | | 6.21 | | 0 | | | Down | |
| MXLOC_059253 | 5660 | -7.1797264 | | 6.22 | | 0 | | | Down | |
| Sra1 | 866 | -7.1936096 | | 6.25 | | 0 | | | Down | |
| MXLOC_035968 | 7220 | -7.2120172 | | 6.29 | | 0 | | | Down | |
| Fat3 | 18468 | -7.2120172 | | 6.29 | | 0 | | | Down | |
| Ap1b1 | 4315 | -7.2244218 | | 58.37 | | 4.62 | | | Down | |
| MXLOC_058804 | 4081 | -7.25301 | | 6.38 | | 0 | | | Down | |
| MXLOC_087606 | 4583 | -7.2755347 | | 6.43 | | 0 | | | Down | |
| Bnip2 | 5770 | -7.3067766 | | 6.5 | | 0 | | | Down | |
| Tmem102 | 1941 | -7.3332888 | | 6.56 | | 0 | | | Down | |
| Grin2b | 7505 | -7.3569586 | | 22.62 | | 1.71 | | | Down | |
| Prpf4b | 4466 | -7.3769416 | | 6.66 | | 0 | | | Down | |
| Dennd1a | 4352 | -7.3812707 | | 6.67 | | 0 | | | Down | |
| Kif21b | 9119 | -7.3855934 | | 6.68 | | 0 | | | Down | |
| Ankrd10 | 2898 | -7.4999232 | | 6.95 | | 0 | | | Down | |
| Gm21949 | 2397 | -7.5040718 | | 6.96 | | 0 | | | Down | |
| MXLOC_086981 | 3767 | -7.520607 | | 7 | | 0 | | | Down | |
| MXLOC_012390 | 7976 | -7.520607 | | 7 | | 0 | | | Down | |
| MXLOC_069268 | 7956 | -7.520607 | | 7 | | 0 | | | Down | |
| Csk | 2749 | -7.520607 | | 7 | | 0 | | | Down | |
| Mkrn1 | 2936 | -7.520607 | | 7 | | 0 | | | Down | |
| MXLOC_059706 | 2364 | -7.520607 | | 14 | | 1 | | | Down | |
| MXLOC_038531 | 5713 | -7.5805023 | | 18.01 | | 1.26 | | | Down | |
| MXLOC_075901 | 4280 | -7.6138885 | | 7.23 | | 0 | | | Down | |
| Speg | 4405.48 | -7.6298079 | | 7.27 | | 0 | | | Down | |
| Cyb561d2 | 1782 | -7.6377348 | | 7.29 | | 0 | | | Down | |
| MXLOC_089992 | 608 | -7.6653087 | | 7.36 | | 0 | | | Down | |
| MXLOC_030404 | 4360 | -7.6926216 | | 7.43 | | 0 | | | Down | |
| MXLOC_090315 | 10203 | -7.7464838 | | 7.57 | | 0 | | | Down | |
| Gps1 | 2000 | -7.8105643 | | 7.74 | | 0 | | | Down | |
| Jak1 | 5299 | -7.8217264 | | 7.77 | | 0 | | | Down | |
| Map3k12 | 5352 | -7.8622885 | | 7.88 | | 0 | | | Down | |
| Rbm4b | 1830 | -7.8914341 | | 7.96 | | 0 | | | Down | |
| MXLOC_021426 | 2945 | -7.9058972 | | 8 | | 0 | | | Down | |
| Amz2 | 2816 | -7.9058972 | | 8 | | 0 | | | Down | |
| Ybx3 | 1655 | -7.9058972 | | 8 | | 0 | | | Down | |
| Nsrp1 | 3116 | -7.9058972 | | 8 | | 0 | | | Down | |
| Zswim8 | 6052 | -7.9058972 | | 8 | | 0 | | | Down | |
| Pank2 | 4266 | -7.9524079 | | 8.13 | | 0 | | | Down | |
| MXLOC_045032 | 3220 | -8.025992 | | 8.34 | | 0 | | | Down | |
| Ssbp2 | 6566 | -8.0909887 | | 8.53 | | 0 | | | Down | |
| Kif3a | 5445.5 | -8.097746 | | 8.55 | | 0 | | | Down | |
| Vwa5b2 | 4527 | -8.1907241 | | 8.83 | | 0 | | | Down | |
| Nbea | 10983 | -8.1971456 | | 27.08 | | 1.53 | | | Down | |
| Kcnj3 | 4998 | -8.1972521 | | 8.85 | | 0 | | | Down | |
| MXLOC_034826 | 4032 | -8.2457472 | | 9 | | 0 | | | Down | |
| Sepw1 | 705 | -8.2457472 | | 9 | | 0 | | | Down | |
| Gm3448 | 787 | -8.3060269 | | 9.19 | | 0 | | | Down | |
| MXLOC_081534 | 20537 | -8.3465571 | | 9.32 | | 0 | | | Down | |
| Decr2 | 2051 | -8.3674576 | | 18.4 | | 0 | | | Down | |
| Rbbp9 | 2160 | -8.4199184 | | 9.56 | | 0 | | | Down | |
| Tnks1bp1 | 5747 | -8.5497534 | | 4 | | 0 | | | Down | |
| MXLOC_079620 | 4432 | -8.5497534 | | 10 | | 0 | | | Down | |
| MXLOC_084666 | 4795 | -8.5497534 | | 10 | | 0 | | | Down | |
| Mfhas1 | 6408 | -8.5497534 | | 10 | | 0 | | | Down | |
| Slain1 | 2740 | -8.6040615 | | 10.19 | | 0 | | | Down | |
| Arfrp1 | 2596 | -8.6322394 | | 10.29 | | 0 | | | Down | |
| Tmem55b | 1481 | -8.6629204 | | 10.4 | | 0 | | | Down | |
| Cntnap1 | 5360 | -8.6960228 | | 10.52 | | 0 | | | Down | |
| Cadm2 | 9537 | -8.7341683 | | 10.66 | | 0 | | | Down | |
| Samd10 | 2099 | -8.7395767 | | 10.68 | | 0 | | | Down | |
| MXLOC_014982 | 3974 | -8.7771544 | | 10.82 | | 0 | | | Down | |
| MXLOC_027481 | 2123 | -8.8195095 | | 10.98 | | 0 | | | Down | |
| Grasp | 2014 | -8.8247604 | | 11 | | 0 | | | Down | |
| Sgms2 | 5790 | -8.8247604 | | 11 | | 0 | | | Down | |
| Pgm2l1 | 8626 | -8.8273823 | | 11.01 | | 0 | | | Down | |
| Nsun5 | 2242 | -8.9098474 | | 183.08 | | 8.08 | | | Down | |
| MXLOC_054577 | 3862 | -9.0782257 | | 12.01 | | 0 | | | Down | |
| MXLOC_021448 | 2889 | -9.3067766 | | 13 | | 0 | | | Down | |
| MXLOC_004165 | 7456 | -9.3067766 | | 13 | | 0 | | | Down | |
| MXLOC_003977 | 8210 | -9.3067766 | | 13 | | 0 | | | Down | |
| MXLOC_074719 | 3681 | -9.4135341 | | 13.49 | | 0 | | | Down | |
| 5730455P16Rik | 4536 | -9.4412068 | | 13.62 | | 0 | | | Down | |
| Khdc1a | 1950 | -9.4707143 | | 13.76 | | 0 | | | Down | |
| Arhgap44 | 4022 | -9.520607 | | 14 | | 0 | | | Down | |
| MXLOC_087379 | 8739.01 | -9.6799478 | | 23.08 | | 0 | | | Down | |
| Fbxo22 | 2102 | -9.6928374 | | 5.35 | | 0 | | | Down | |
| Phospho1 | 1910 | -9.7100444 | | 14.95 | | 0 | | | Down | |
| MXLOC_046398 | 2943 | -9.778702 | | 15.31 | | 0 | | | Down | |
| MXLOC_089374 | 1276 | -9.8402344 | | 15.64 | | 0 | | | Down | |
| MXLOC_058640 | 2427 | -10.114571 | | 17.2 | | 0 | | | Down | |
| Bin1 | 2122 | -10.239328 | | 17.96 | | 0 | | | Down | |
| Cadps | 5478 | -10.245747 | | 18 | | 0 | | | Down | |
| 1810010H24Rik | 1099 | -10.306027 | | 18.38 | | 0 | | | Down | |
| Rev1 | 4262 | -10.332592 | | 18.55 | | 0 | | | Down | |
| Tpd52l2 | 3371 | -10.609719 | | 20.42 | | 0 | | | Down | |
| Tro | 7153 | -10.690532 | | 21 | | 0 | | | Down | |
| Golga1 | 4793 | -10.78913 | | 21.73 | | 0 | | | Down | |
| Enpp4 | 4557 | -10.819509 | | 21.96 | | 0 | | | Down | |
| Sec24c | 4218 | -10.911322 | | 22.67 | | 0 | | | Down | |
| Bahcc1 | 10727 | -11.075822 | | 24 | | 0 | | | Down | |
| Pex12 | 2726 | -11.349736 | | 26.39 | | 0 | | | Down | |
| Exosc4 | 1789 | -11.810564 | | 30.96 | | 0 | | | Down | |
| MXLOC_013257 | 4365 | -12.285546 | | 36.5 | | 0 | | | Down | |
| MXLOC_075002 | 4625 | -12.409335 | | 38.1 | | 0 | | | Down | |
| Gpr161 | 6798 | -12.44546 | | 38.58 | | 0 | | | Down | |
| Eps15 | 4233 | -12.585597 | | 10.53 | | 0 | | | Down | |
| Fam53b | 5068 | -12.878039 | | 44.82 | | 0 | | | Down | |
| Dopey1 | 9124 | -13.160527 | | 49.43 | | 0 | | | Down | |
